# Discovery of branching meroterpenoid biosynthetic pathways in *Aspergillus insuetus*: involvement of two terpene cyclases with distinct cyclization modes†

**DOI:** 10.1039/d2sc02994d

**Published:** 2022-08-17

**Authors:** Jia Tang, Yudai Matsuda

**Affiliations:** Department of Chemistry, City University of Hong Kong Tat Chee Avenue Kowloon Hong Kong SAR China ymatsuda@cityu.edu.hk

## Abstract

The aromatic polyketide 3,5-dimethylorsellinic acid (DMOA) serves as a precursor for many fungal meroterpenoids. A large portion of DMOA-derived meroterpenoids are biosynthesized *via* the cyclization of (6*R*,10′*R*)-epoxyfarnesyl-DMOA methyl ester (1). Theoretically, although 1 can be cyclized into many products, only three cyclization modes have been reported. Here, we discovered a meroterpenoid biosynthetic gene cluster in *Aspergillus insuetus* CBS 107.25, which encodes the biosynthetic enzymes for 1 along with a terpene cyclase that is phylogenetically distantly related to the other characterized cyclases of 1. Intriguingly, InsA7, the terpene cyclase, folds 1 in a pre-boat-chair conformation, generating a new meroterpenoid species with an axially oriented hydroxy group at C3. The *A. insuetus* strain also harbors an additional gene cluster encoding another cyclase of 1. The second terpene cyclase–InsB2–also synthesizes a new cyclized product of 1, thereby leading to diverging of the biosynthetic pathway in the fungus. Finally, we characterized the tailoring enzymes encoded by the two clusters, collectively obtained 17 new meroterpenoids, and successfully proposed biosynthetic routes leading to apparent end products of both pathways.

## Abstract 2

Branching meroterpenoid biosynthetic pathways were discovered in the fungus *Aspergillus insuetus* CBS 107.25, in which two terpene cyclases, InsA7 and InsB2, accept the same substrate but generate distinctly cyclized products.

## Graphical abstract

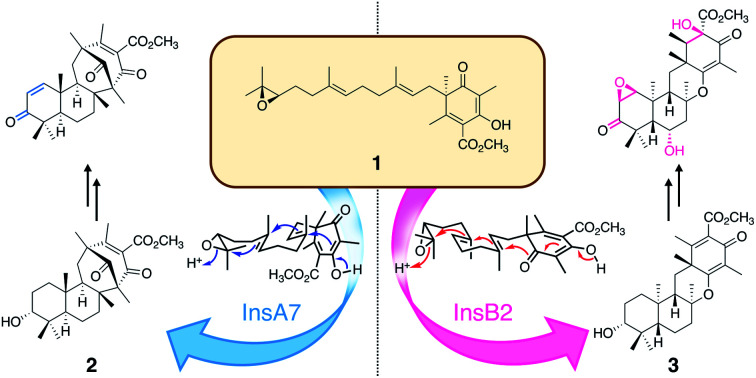

## Introduction

Fungal meroterpenoids derived from the aromatic polyketide 3,5-dimethylorsellinic acid (DMOA) comprise many structurally complicated molecules.^1,2^ Because of the fully substituted nature of DMOA, prenylation reactions in DMOA-derived meroterpenoid biosynthesis occur *via* dearomatization reactions, facilitating the functionalization and rearrangement reactions of the polyketide moiety.^3,4^ In recent years, several enzymes responsible for structural rearrangements, including bridged-ring synthesis in anditomin biosynthesis,^5,6^ ring expansion in terretonin biosynthesis,^7,8^ and orthoester formation in novofumigatonin biosynthesis,^9^ have been identified and characterized. In addition to the many diverse tailoring enzymes, terpene cyclases, which are key enzymes for terpenoid cyclization,^10^ greatly contribute to the diversity of this class of natural products.^11^ Although many terpene cyclases involved in fungal meroterpenoid pathways have been discovered, little is known about how these enzymes catalyze and achieve diverse cyclization reactions.

**Fig. 1:**
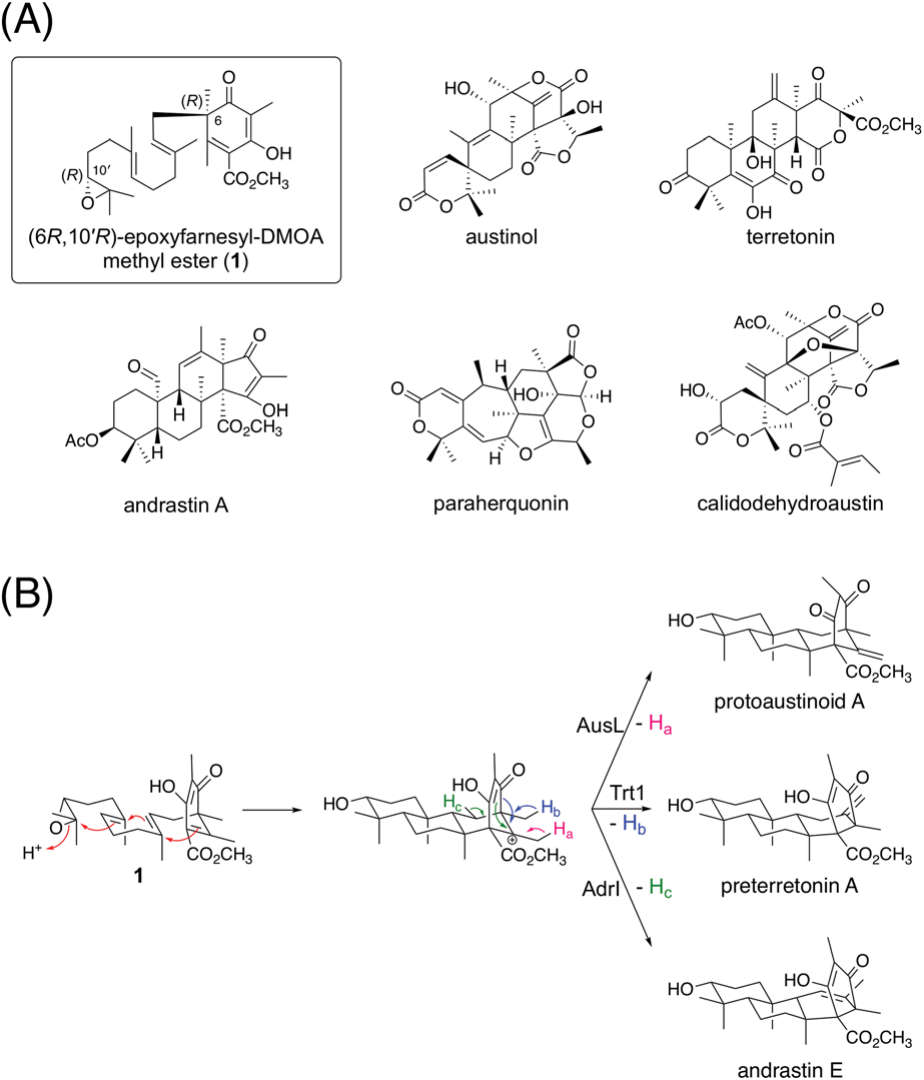
(A) Structures of (6*R*,10′*R*)-epoxyfarnesyl-DMOA methyl ester (1) and selected fungal natural products biosynthesized *via*1. (B) Known cyclization modes of 1.

A considerable number of DMOA-derived meroterpenoids are biosynthesized *via* the cyclization of (6*R*,10′*R*)-epoxyfarnesyl-DMOA methyl ester (1) (Fig. 1A). Currently, 1 is known to be cyclized into protoaustinoid A, preterretonin A, and andrastin E *via* reactions catalyzed by AusL, Trt1, and AdrI, respectively (Fig. 1B).^12,13^ All of these three enzymes cyclize 1 into a common tetracyclic cationic intermediate; however, the final deprotonation occurs from different carbon atoms, thereby leading to the diverging of the reaction pathways. Although 1 can theoretically be cyclized into more diverse products, no such molecules have been reported yet. Interestingly, in a recent study, synthetically prepared 1 and its analogues were subjected to enzymatic reactions with a series of terpene cyclases, some of which yielded products with novel meroterpenoid scaffolds.^14^ Moreover, a biosynthetic study of atlantinone B, a DMOA-derived meroterpenoid, demonstrated that the terpene cyclase AtlC can accept (6*R*,10′*R*)-epoxyfarnesyl-DMOA, an analogue of 1 with a free carboxy group, to yield three meroterpenoids, each containing a monocyclic terpenoid moiety.^15^ These reports suggest that terpene cyclases having new cyclization modes could be mined by investigating a large number of available fungal genome sequences.

In this study, we discovered a meroterpenoid biosynthetic gene cluster in *Aspergillus insuetus* CBS 107.25, which encodes enzymes for the synthesis of 1, as well as a terpene cyclase that is phylogenetically distant from the known cyclases of 1. Intriguingly, InsA7, the terpene cyclase encoded by the cluster, cyclizes 1 into a tetracyclic product with a C3 axial hydroxy group *via* an unusual boat-chair substrate conformation. Furthermore, *A. insuetus* CBS 107.25 harbors a second gene cluster that comprises a meroterpenoid cyclase gene but lacks genes for a precyclized intermediate. InsB2, the second terpene cyclase, also accepts 1 and yields a new cyclized product, thereby providing branching meroterpenoid pathways in the fungus. In the present study, we also characterized tailoring enzymes encoded by the two gene clusters and identified 17 previously undescribed fungal meroterpenoids.

## Results

### Discovery and bioinformatic analysis of the meroterpenoid biosynthetic gene clusters in *Aspergillus insuetus*

**Fig. 2:**
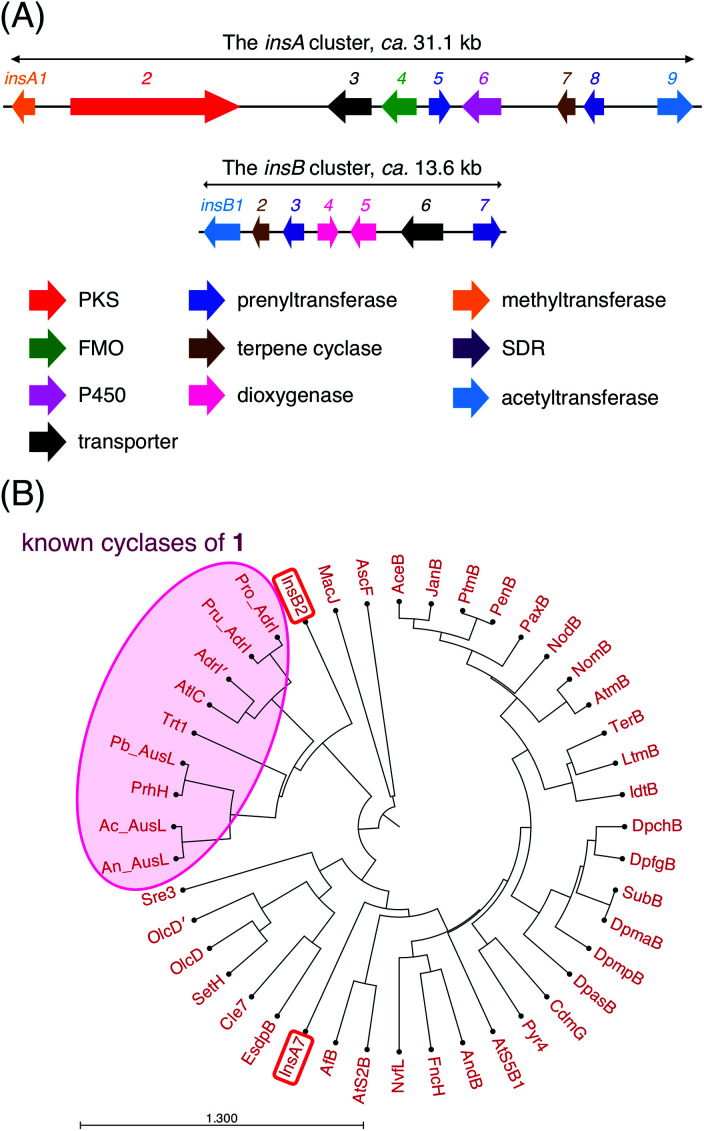
(A) Schematic representation of the *insA* and *insB* clusters in *Aspergillus insuetus* CBS 107.25. (B) Phylogenetic analysis of InsA7, InsB2, and other terpene cyclases involved in the fungal meroterpenoid biosynthesis. An: *Aspergillus nidulans*; Ac: *Aspergillus calidoustus*; Pb: *Penicillium brasilianum*; Pru: *Penicillium rubens*; Pro: *Penicillium roqueforti*. The phylogenetic tree was drawn using CLC Main Workbench 22.0 (QIAGEN) with the Kimura protein distance analysis (UPGMA algorithm).

To discover a biosynthetic gene cluster for a new DMOA-derived meroterpenoid, we searched for DMOA synthase gene homologues in publicly available fungal genome databases and assessed the flanking regions of each identified gene. We found that *A. insuetus* CBS 107.25 contains a genomic region that is somewhat similar to the known gene clusters involved in DMOA-derived meroterpenoid biosynthesis (Fig. 2A). The gene cluster, designated as the *insA* cluster, encodes the non-reducing polyketide synthase InsA2, the prenyltransferase InsA5, the *O*-methyltransferase InsA1, the flavin-dependent monooxygenase InsA4, and the terpene cyclase InsA7, which are required for the generation of the first cyclized intermediate in the biosynthesis. Among these five enzymes, InsA2, InsA5, InsA1, and InsA4 are highly homologous to AusA, AusN, AusM, and AusD, respectively (>80% amino acid sequence identity), which are involved in the synthesis of 1 in *A. calidoustus*.^16,17^ Therefore, the four InsA enzymes could also be involved in the formation of 1 (Fig. S1†). Intriguingly, phylogenetic analysis revealed that the terpene cyclase InsA7 is distantly related to the known cyclases of 1 (Fig. 2B), and accordingly, InsA7 exhibits relatively low similarity with the other cyclization enzymes of 1 (<40% amino acid sequence identity). Thus, InsA7 may be able to cyclize 1 in a manner distinct from that in the preceding cases. The *insA* cluster also encodes the cytochrome P450 monooxygenase InsA6, the short-chain dehydrogenase/reductase (SDR) InsA8, and the acetyltransferase InsA9, which appear to be involved in late-stage biosynthesis.

In addition, analysis of the *A. insuetus* genome revealed the presence of another gene cluster with a meroterpenoid cyclase gene, which was designated as the *insB* cluster (Fig. 2A). Except for the terpene cyclase gene, this cluster lacks the genes required for backbone synthesis; however, it encodes several potential tailoring enzymes, namely, the acetyltransferase InsB1, the two SDRs InsB3 and InsB7, and the two α-ketoglutarate (αKG)-dependent dioxygenases InsB4 and InsB5. Although the terpene cyclase InsB2 belonged to the same clade as the known cyclases of 1 and was most similar to AdrI in the andrastin biosynthetic pathway (Fig. 2B),^13^ it displayed only moderate similarity with the enzymes in the clade (approximately 40% amino acid sequence identity). Since the *insB* cluster was found on a small scaffold (*ca.* 16.6 kb) that only contains the *insB* genes, we were unable to exclude the possibility that the flanking regions of the *insB* cluster encode the enzymes that synthesize the substrate of InsB2. Nevertheless, given the fact that InsB2 is most closely related to the known cyclases of 1, we reasoned that both InsA7 and InsB2 react with 1 and provide differently cyclized products.

Notably, other *A. insuetus* strains reportedly produce two types of DMOA-derived meroterpenoids: terretonins E and F and insuetolides A–C;^18,19^ however, genes that seem to be responsible for the biosynthesis of these molecules were not found in the genome of *A. insuetus* CBS 107.25.

### Functional analysis of the terpene cyclases InsA7 and InsB2

**Fig. 3:**
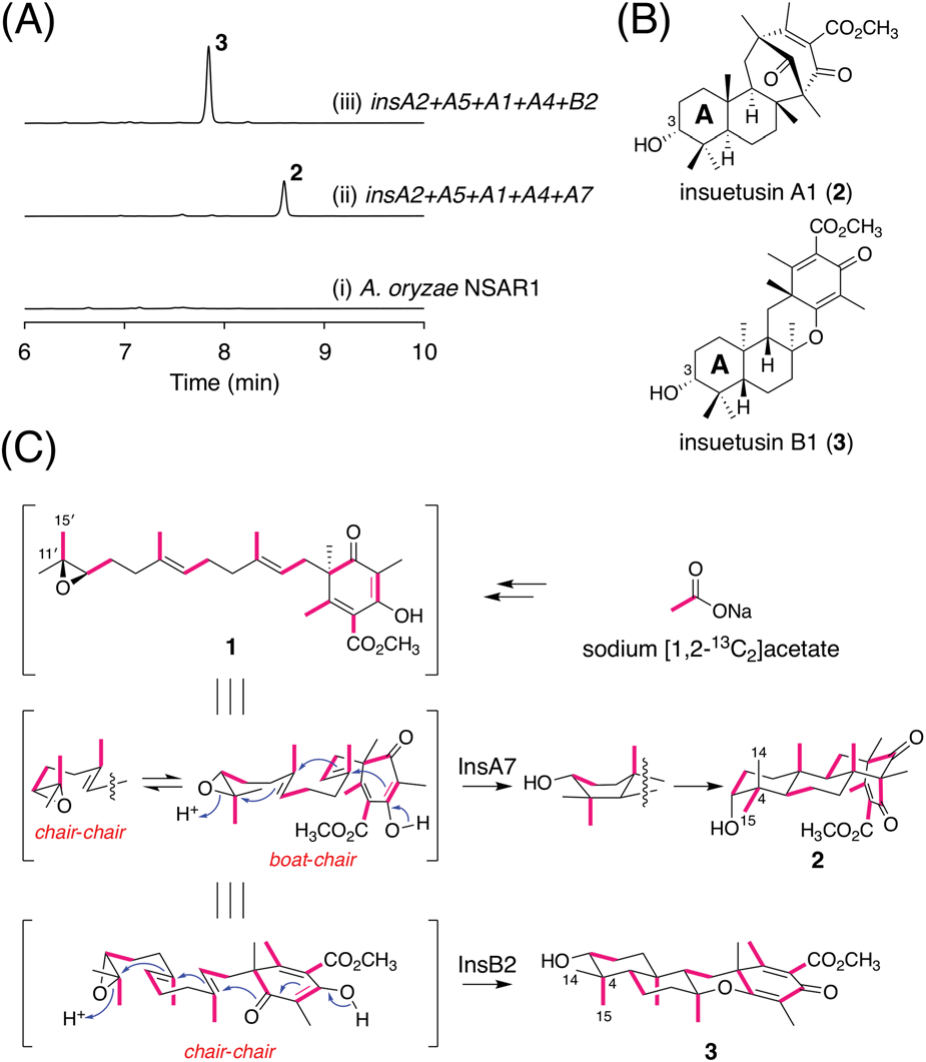
(A) HPLC profiles of the metabolites derived from *A. oryzae* transformants. The chromatograms were monitored at 254 nm. (B) Structures of insuetusins A1 (2) and B1 (3). (C) Results of isotope incorporation experiments performed using sodium [1,2–^13^C_2_]acetate.

To characterize the functions of InsA7 and InsB2, we heterologously expressed *insA7* and *insB2* along with *insA2*, *insA5*, *insA1*, and *insA4*, which are the genes involved in the biosynthesis of the putative substrate of the two cyclases, in *A. oryzae* NSAR1,^20^ a powerful platform for the refactoring of natural product biosynthesis in fungi.^21–24^ The metabolites of the *A. oryzae* transformants were analyzed using high-performance liquid chromatography (HPLC). The HPLC analysis revealed the existence of different major products 2 and 3 in the two strains (Fig. 3A), of which 2 was also detected in *A. insuetus* CBS 107.25 (Fig. S2†). The molecular formulae of both metabolites were established as C_26_H_38_O_5_, which corresponds to the molecular formula of the expected cyclized product. To determine the structures of both products, we cultivated the *A. oryzae* transformants on a large scale and successfully isolated the compounds for nuclear magnetic resonance (NMR) spectroscopy analysis.

The ^1^H NMR spectrum of 2, the product of InsA7, revealed the presence of an oxymethine signal at 3.37 ppm; this signal could be attributable to the existence of a hydroxy group resulting from the epoxide ring-opening and the subsequent cyclization of the farnesyl group. However, remarkably, the coupling pattern of the signal (t, *J* = 2.8 Hz) indicated that the C3 hydroxy group of 2 was axially oriented, which clearly contradicts the fact that the other characterized meroterpenoid cyclases generate an equatorially oriented hydroxy group after cyclization. Further analysis of ^13^C and the 2D NMR spectra confirmed the structure of 2, showcasing its relative stereochemistry, as depicted in Fig. 3B, which features the [3.3.1] bridged bicyclic system and a C3 axial hydroxy group. The absolute configuration of 2 was deduced on the basis of the expectation that it is derived from 1, with (*R*)-epoxide, and that 2 should thus possess the (3*R*)-hydroxy group. Interestingly, except for the C3 axial hydroxy group, the stereocenters in the A- and B-rings of 2 have opposite configurations to those of the other known cyclized products of 1. Compound 2 was hereby designated as insuetusin A1.

In the ^1^H NMR spectrum of 3 (the InsB2 product), at 3.28 ppm, the oxymethine signal was observed as a double doublet (*J* = 11.6 and 4.4 Hz), which is typically observed for products of meroterpenoid cyclases that form the equatorially oriented hydroxy group. Structural analysis of 3 revealed that its NMR spectra match well with those of an unnatural meroterpenoid [compound 26 from the study by Powers *et al.*^25^] that is cyclized from (6*S*,10′*S*)-epoxyfarnesyl-DMOA methyl ester, a synthetically prepared enantiomer of 1. Furthermore, the specific rotation of 3 ([*α*]^22.5^_D_ −100.3) displayed an opposite sign to that of the synthetic meroterpenoid ([*α*]^31.5^_D_ +81.1),^14^ which established the structure of 3 with the 6,6,6,6-fused ring system, as shown in Fig. 3B. Compound 3 was designated as insuetusin B1.

Next, we sought to obtain in-depth insights into the cyclization mechanism to form the unusual terpenoid moiety of 2. The folding mode of the linear substrate of terpene cyclases can be determined by tracing the origin of the two methyl groups at C4.^26,27^ Accordingly, we cultivated the *A. oryzae* transformant synthesizing 2 in the presence of sodium [1,2-^13^C_2_]acetate. An intact acetate unit is incorporated at C11′/C15′ of 1; by checking which of C14 or C15 is coupled with C4 in the ^13^C NMR spectrum of 2, how 1 is folded in the enzyme prior to cyclization was understood (Fig. 3C). Thus, C–C coupling was observed between C4 and C15, indicating that cyclization by InsA7 proceeds *via* a pre-boat-chair conformation (Fig. 3C and Table S6†). For comparison, a similar isotope incorporation experiment was performed using a 3-producing *A. oryzae* strain; an intact acetate unit was incorporated at C4/C15, which is consistent with a common pre-chair-chair conformation (Fig. 3C and Table S7†).

### Investigation of the late-stage biosynthesis of the *insA* pathway

**Fig. 4:**
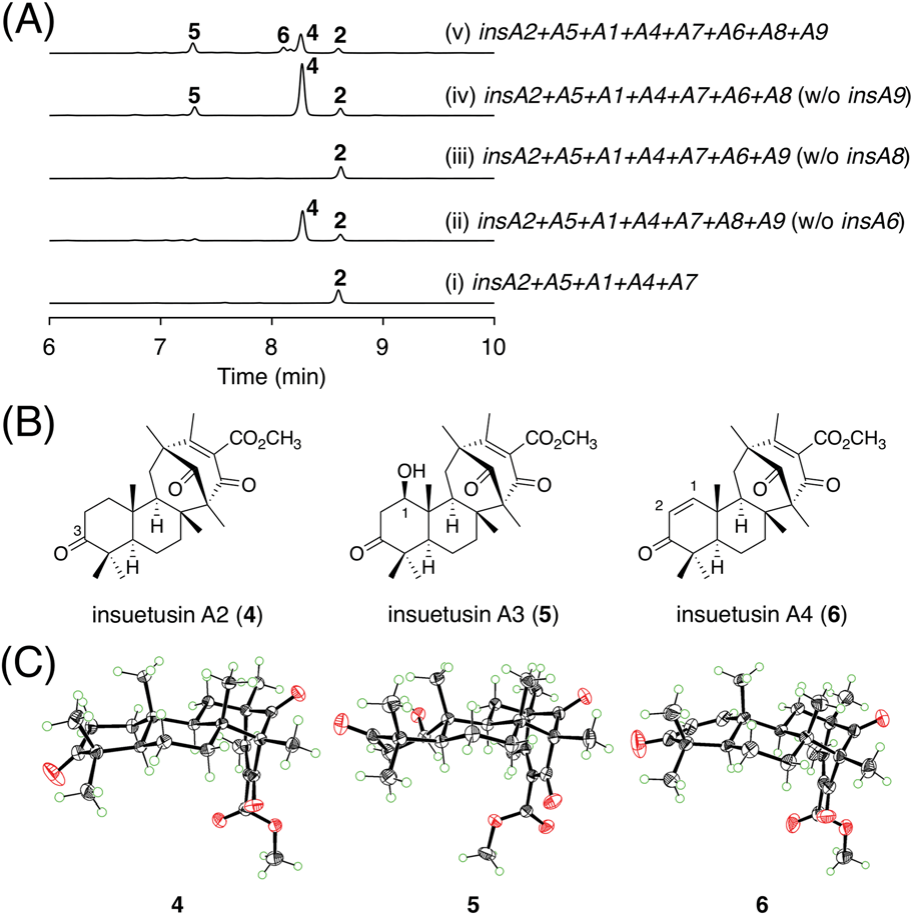
(A) HPLC profiles of the metabolites derived from the *A. oryzae* transformants. The chromatograms were monitored at 254 nm. (B) Structures of 4–6. (C) X-ray crystal structures of 4–6.

Next, we aimed to elucidate the late-stage biosynthesis of the *insA* pathway; this pathway involves the P450 InsA6, the SDR InsA8, and the acetyltransferase InsA9. Because the order of the reactions catalyzed by the three enzymes was not predictable, we created three *A. oryzae* transformants, in which two of the three genes were expressed alongside the five genes involved in the production of 2. The metabolic profile of the strain without the SDR gene *insA8* was almost identical to that of the transformant expressing the five genes (Fig. 4A; traces (i) and (iii)), indicating that InsA8 accepts 2 as its substrate. The transformant lacking the P450 gene *insA6* yielded a new metabolite 4 (Fig. 4A; trace (ii)), which was determined to be the C3 keto form of 2 (Fig. 4B). When the acetyltransferase gene *insA9* was omitted, the transformant generated an additional product 5 (Fig. 4A; trace (iv)). Compound 5 is a hydroxylated analogue of 4 and has a β-hydroxy group at the C1 position (Fig. 4B). Subsequently, we generated another *A. oryzae* transformant harboring all of the three genes to obtain the end product of the *insA* pathway. The transformant provided a specific metabolite 6 (Fig. 4A; trace (v)); this was then characterized to be the dehydrated form of 5 with a double bond at C1/C2 (Fig. 4B). To further confirm the structures of 4–6, single crystals of these metabolites were obtained and subjected to X-ray diffraction analysis with CuKα radiation; consequently, the structures of compounds 4, 5, and 6, including their absolute configurations, were unambiguously determined with Flack parameters of 0.01(3), 0.04(4), and 0.02(11), respectively (Fig. 4C; CCDC: 2174818, 2174817, and 2174819, respectively). The structures of these compounds also substantiate the deduced absolute structure of 2 (Fig. 3B). Compounds 4, 5, and 6 were hereby designated as insuetusins A2, A3, and A4, respectively.

On the basis of the abovementioned experimental data, the functions of the three enzymes are proposed as follows. The SDR InsA8 is responsible for alcohol dehydrogenation at the C3 position. The P450 InsA6 introduces the 1β-hydroxy group, which is acetylated by the acetyltransferase InsA9. The resultant acetoxy group is spontaneously eliminated to produce a C1/C2 double bond. The apparent end product 6 was also detected in the *A. insuetus* strain (Fig. S2†), indicating that the *insA* cluster is active in the fungus.

### Investigation of the late-stage biosynthesis of the *insB* pathway

**Fig. 5:**
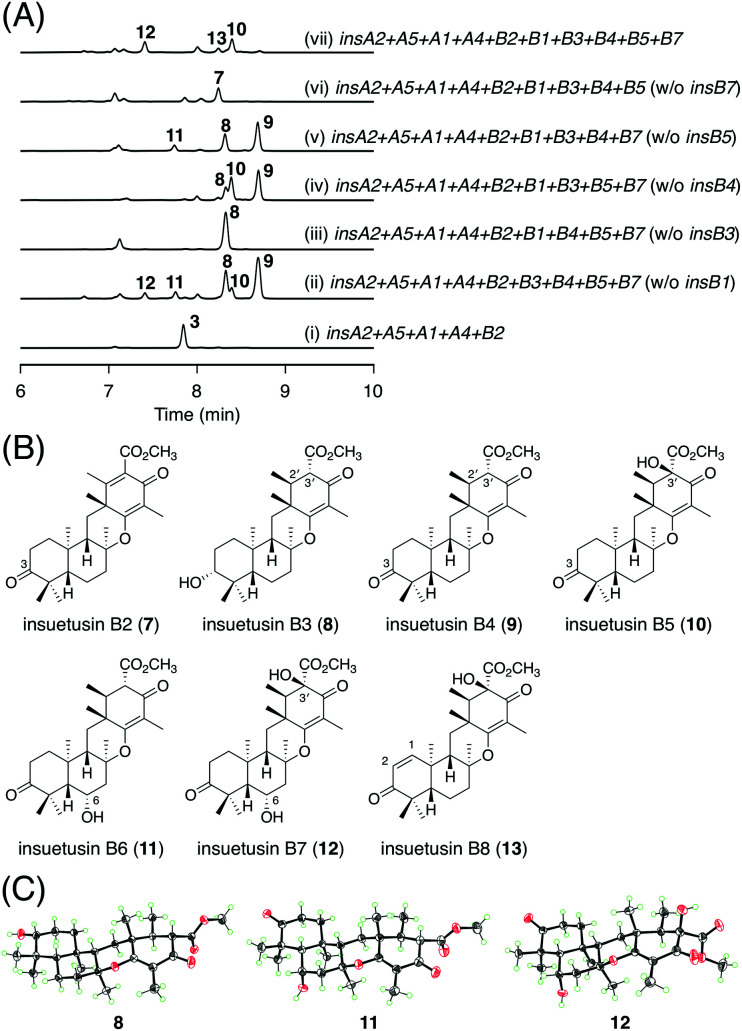
(A) HPLC profiles of the metabolites derived from the *A. oryzae* transformants. The chromatograms were monitored at 270 nm. (B) Structures of 7–13. (C) X-ray crystal structures of 8, 11, and 12.

The late-stage biosynthesis of the *insB* pathway was investigated in a manner similar to that for the *insA* pathway. In brief, a series of five *A. oryzae* transformants, each missing a distinct one of the five tailoring enzyme genes, were created, and their metabolites were isolated and characterized primarily using NMR spectroscopy. All five of the resulting transformants exhibited metabolic profiles different from that of the five-gene expressing strain that synthesizes 3 (Fig. 5A; traces (i to vi)). The transformant lacking the SDR gene *insB7* yielded a major new metabolite 7 (Fig. 5A; trace (vi)), which was determined to be the 3-keto form of 3 (Fig. 5B). Likewise, the omission of *insB3*, the other SDR gene, led to the accumulation of another major product 8 (Fig. 5A; trace (iii)). In compound 8, the double bond at the C2′/C3′ of 3 was reduced to a single bond (Fig. 5B). When the αKG-dependent dioxygenase gene *insB4* was missing, two additional products 9 and 10, as well as the previously determined product 8, were observed (Fig. 5A; trace (iv)). Compound 9 was characterized to harbor the 3-keto group and a single bond at C2′/C3′ (Fig. 5B), whereas 10 was determined to be the 3′-hydroxy form of 9 (Fig. 5B). The strain lacking *insB5*, the other dioxygenase gene, produced a new compound 11 (Fig. 5A; trace (v)), which was characterized as the 6α-hydroxy derivative of 9 (Fig. 5B). Compound 12, the dihydroxy form of 9 with the 6α- and 3′-hydroxy groups (Fig. 5B), was specifically obtained from the transformant lacking the acetyltransferase gene *insB1* (Fig. 5A; trace (ii)). Finally, we generated a transformant expressing all of the five tailoring enzymes. Among the several metabolites produced by this transformant, 13 was found to be unique to the strain (Fig. 5A; trace (vii)) and was determined to be the dehydrogenated form of 10, with a double bond at C1/C2 (Fig. 5B). Compounds 8, 11, and 12 were successfully crystallized and subjected to X-ray diffraction analysis, and their absolute structures were established with Flack parameters of 0.09(4), 0.00(3), and 0.03(3), respectively (Fig. 5C; CCDC: 2174822, 2174820, and 2174821, respectively). Compounds 7–13 were designated as insuetusins B2–B8, respectively.

**Fig. 6:**
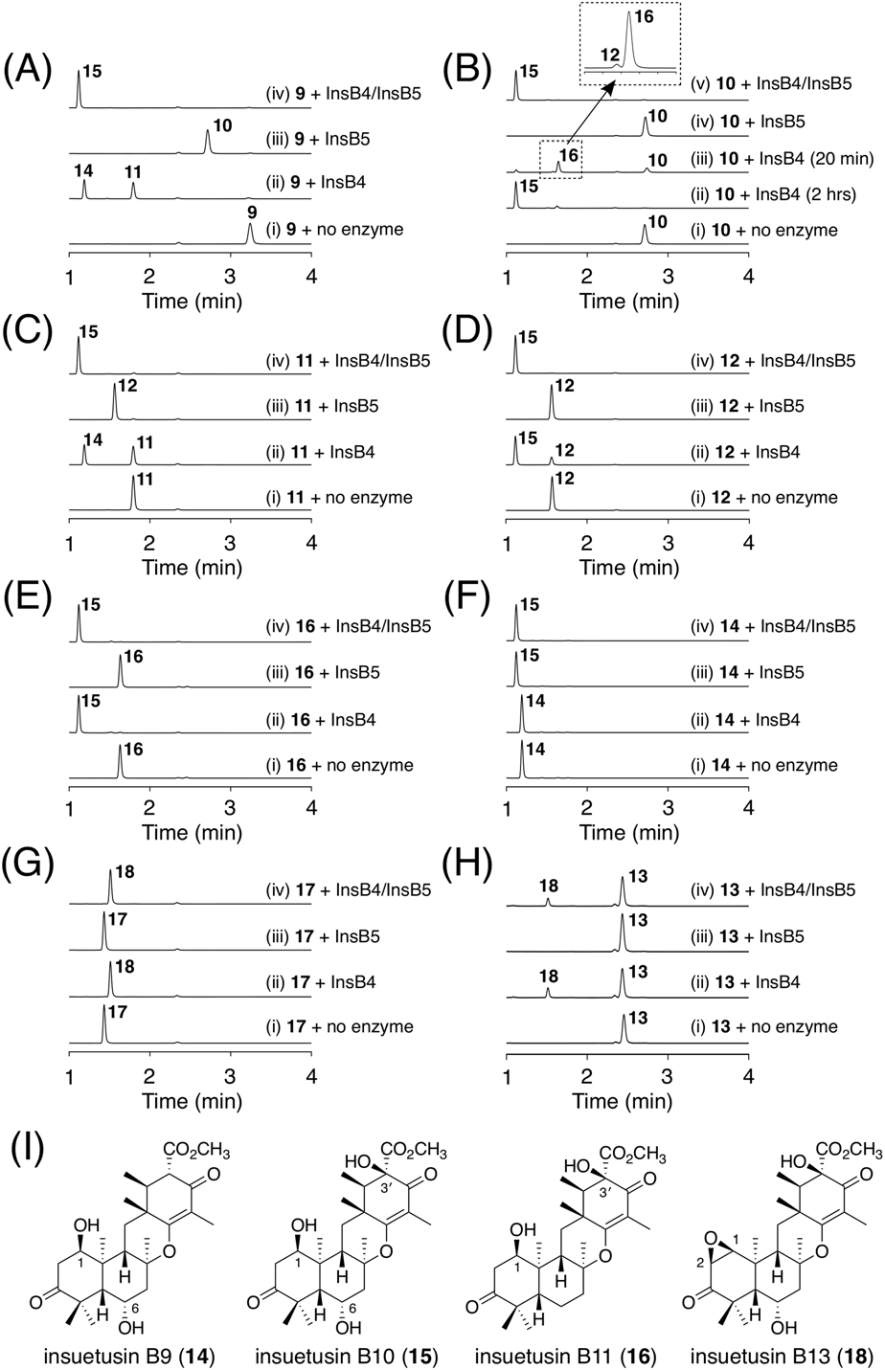
(A–H) HPLC profiles of the products from the *in vitro* enzymatic reactions. The enzymatic reactions were performed for 2 hours, unless otherwise mentioned. The chromatograms were monitored at 270 nm. (I) Structures of 14–16 and 18.

This experiment allowed a rough estimation of the functions of the five tailoring enzymes. The two SDRs InsB3 and InsB7 are involved in C-3 alcohol dehydrogenation and enoylreduction at C2′/C3′, respectively. The two dioxygenases InsB4 and InsB5 are involved in multiple hydroxylation reactions. The acetyltransferase InsB1 has a similar function to InsA9; InsB1 acetylates the C1 hydroxy group, which is followed by the spontaneous elimination of acetate to introduce the C1/C2 unsaturated bond. However, due to the complicated metabolic profiles of the transformants, a complete image of the *insB* pathway was difficult to obtain solely based on the heterologous expression experiment. Because αKG-dependent dioxygenases are often multifunctional and/or play critical roles in the structural complexification of secondary metabolites,^28–30^ we tried to further characterize the functions of the two dioxygenases InsB4 and InsB5; both of these enzymes were expressed as His-tagged proteins in the *Escherichia coli* expression system for *in vitro* enzymatic reactions (Fig. S3†). Based on the results of the *in vivo* experiment, 9 was suggested to be first generated *via* reactions catalyzed by the two SDRs InsB3 and InsB7 and then oxidized by the two dioxygenases. Thus, InsB4 and InsB5 were determined to individually react with 9, according to a standard reaction condition for αKG-dependent dioxygenases.^5,9,31^ The reaction of InsB4 with 9 yielded two products, of which one was identified as the 6α-hydroxy product 11 (Fig. 6A; trace ii). The other product 14 was isolated from a large-scale enzymatic reaction and characterized as the dihydroxylated product of 9 harboring 1β- and 6α-hydroxy groups (Fig. 6I). In contrast, InsB5 hydroxylated 9 at the C3′ position to produce 10 (Fig. 6A; trace (iii)). Subsequently, 9 was incubated with both enzymes, which resulted in the generation of a new product 15 (Fig. 6A; trace (iv)). Compound 15 was successfully isolated and characterized as the trihydroxylated form of 9 containing the 1β-, 6α-, and 3′-hydroxy groups (Fig. 6I).

To obtain further insights into the functions of the two dioxygenases, similar *in vitro* reactions were conducted using a series of compounds obtained in this study. When compound 10 possessing the 3′-hydroxy group was used as the substrate, no further oxidation was observed in the reaction with InsB5 (Fig. 6B; trace (iv)). Meanwhile, InsB4 converted 10 into 15*via* two hydroxylation rounds (Fig. 6B; trace (ii)). The reaction with InsB4 was found to produce a new product 16 when the reaction time was shorter (Fig. 6B; trace iii). Although 16 is a mono-oxygenated form of 10, it was not identical to 12, which was obtained from the heterologous expression experiment. Thus, 16 was isolated from a large-scale enzymatic reaction for structural characterization and was determined to be the 1β-hydroxy form of 10 (Fig. 6I). Interestingly, only a trace amount of 12, an isomer of 16, was detected in this reaction. Next, we investigated the intermediacy of 11. Both InsB4 and InsB5 accepted 11 as a substrate to yield 14 and 12, respectively, and the reactions with both enzymes yielded 15 (Fig. 6C). Furthermore, although 12 and 16 do not serve as substrates of InsB5, both of these compounds were accepted by InsB4 and transformed into 15 (Fig. 6D and E). Finally, 14 was found to be hydroxylated by InsB5 to produce 15 (Fig. 6F). Taken together, InsB4 is responsible for hydroxylation reactions at C1 and C6, whereas InsB5 hydroxylates only at C3′. The three new products 14–16 were designated as insuetusin B9–B11, respectively.

**Fig. 7:**
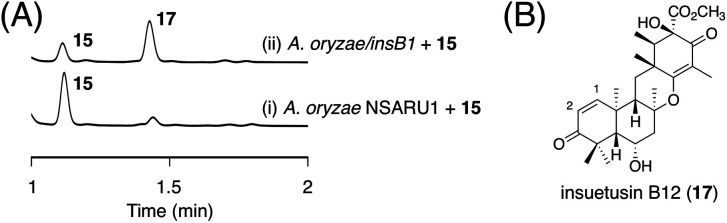
(A) HPLC profiles of bioconversion experiments using the InsB1-expressing *A. oryzae* transformant. The chromatograms were monitored at 270 nm. (B) Structure of 17.

Next, we aimed to obtain the end product of the *insB* pathway. Given the production of 13 with the C1/C2 double bond *in vivo*, we expected that 15 would also undergo a similar dehydration reaction. To investigate this hypothesis, we generated an *A. oryzae* NSARU1 transformant (see ESI† for the method to create this new *A. oryzae* strain) expressing only *insB1*, which was cultivated in the presence of 15. Consequently, 15 was converted to a new metabolite 17 (Fig. 7A), which was, as expected, determined to be the dehydrated form of 15 and named insuetusin B12 (Fig. 7B).

To investigate the possibility that 17 is further oxidized by InsB4 and/or InsB5, *in vitro* enzymatic reactions using 17 as the substrate were performed. Consequently, only InsB4 was found to further oxidize 17 to produce a monooxygenated product 18 (Fig. 6G). Compound 18, designated as insuetusin B13, was found to harbor an epoxide at C1/C2 and was determined to be the apparent end product of the *insB* pathway (Fig. 6I). Likewise, 13 was also converted to 18 by InsB4 (Fig. 6H). Finally, the metabolites of the *A. insuetus* strain were assessed and found to contain 18 (Fig. S2†), thereby confirming that both *insA* and *insB* clusters are actively transcribed in the fungus and that 18 can also be produced *in vivo*.

## Discussion

In this study, we discovered and characterized two new terpene cyclases involved in fungal meroterpenoid biosynthesis. Both cyclases accept (6*R*,10′*R*)-epoxyfarnesyl-DMOA methyl ester (1) as a substrate but produce differently cyclized products (Fig. 3C). Of these two cyclases, the cyclization mode of InsB2 is somewhat similar to those of AusL, Trt1, and AdrI; however, apparently, the polyketide-derived portion is placed in a different manner in InsB2, leading to its unique cyclization mode (Fig. 1B and 3C). In contrast, InsA7 was found to fold 1 in an unusual pre-boat-chair conformation, resulting in the generation of a cyclized product with a C3 axial hydroxy group. Similar cyclization was observed in the reaction of Trt1, the terpene cyclase involved in terretonin biosynthesis, with an unnatural substrate (6*R*,10′*S*)-epoxyfarnesyl-DMOA, which was proposed to proceed *via* pre-boat folding of the A-ring.^14^ Some squalene–hopene cyclases accept unnatural (3*R*)-oxidosqualene to yield a cyclized product with an axially oriented hydroxy group.^26,27,32^ However, these cases used unnatural substrates; therefore, InsA7 represents a rare example of terpene cyclases that fold an epoxyprenyl substrate in the pre-boat-chair conformation, as a natural reaction, to yield a cyclized product with an axial hydroxy group. The results of the phylogenetic analysis showed that InsA7 is more closely related to terpene cyclases that accept an (*S*)-epoxide than to previously characterized enzymes that cyclize 1 (Fig. 2B). The pre-boat folding of the A-ring might be required to enable the protonation of the epoxide by InsA7 and should allow the characteristic cyclization mode of InsA7, which resembles those of the (*S*)-epoxide-accepting cyclases.^5,33,34^ Interestingly, 2, the product of InsA7, was found to be structurally analogous to another DMOA-derived meroterpenoid asperterpene A,^35^ which was isolated from *A. terreus* and is a diastereomer of 4. Asperterpene A and 4 differ at the C8 and C9 positions (Fig. S4†), indicating cyclization in the asperterpene pathway *via* pre-boat folding of the B-ring. The product of the terpene cyclase in the asperterpene biosynthesis has yet to be obtained; however, considering that the *A. terreus* strain also produced a metabolite that appears to be a precursor of terretonin, asperterpene A could also be assumed to be derived from 1. Thus, the cyclase for asperterpene biosynthesis might fold 1 in a pre-boat-boat conformation (Fig. S4†), and the biosynthetic study of asperterpenes would lead to the discovery of a new cyclase of 1.

**Fig. 8:**
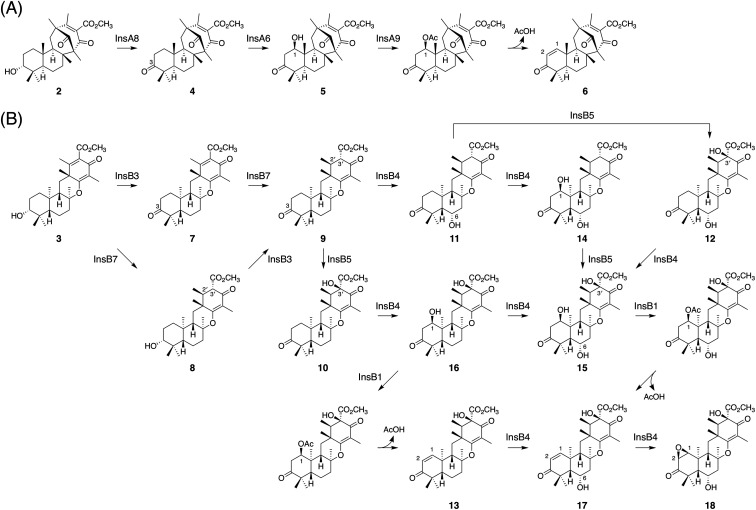
Proposed late-stage biosynthetic pathways of insuetusins (A) A4 (6) and (B) B13 (18).

Another intriguing feature of the insuetusin biosynthesis is the pathway branching to yield two types of meroterpenoid species in a single fungus; metabolic analysis of the *A. insuetus* strain revealed that the pathway branching indeed occurs in the fungus (Fig. S2†). Although 1 serves as the key intermediate of many structurally diverse meroterpenoids, insuetusin pathways are the first example in which 1 is cyclized into two distinct products in a single fungal species. According to the results of our heterologous expression experiments and *in vitro* enzymatic reactions, the complete biosynthetic pathways of insuetusins can be proposed as follows (Fig. 8). In the *insA* pathway (Fig. 8A), the cyclized product 2 is first accepted by the SDR InsA8 and is converted to the 3-keto form 4. Although C3 alcohol dehydrogenation is a common phenomenon in fungal meroterpenoid pathways, InsA8 showed no sequence similarity to known C3 alcohol dehydrogenases such as AdrF,^13^ Trt9,^7^ and OlcF.^36^ This could be because the substrate of InsA8 possesses an axial hydroxy group at C3, whereas all of the other known C3 alcohol dehydrogenases accept equatorially oriented hydroxy groups. Subsequently, 4 undergoes hydroxylation at C1, which is catalyzed by the P450 InsA6 to produce 5. The acetyltransferase InsA9 then acetylates the C1 hydroxy group, and the resultant acetoxy group serves as a good leaving group to install the conjugated olefin at C1/C2, yielding the apparent end product of the *insA* pathway 6. Similar acetyltransferase-assisted dehydration reactions have been observed in the biosynthesis of anditomin and emeridone F.^5,37^

Meanwhile, in the *insB* pathway (Fig. 8B), 3, the product of InsB2, is first converted to 9, *via*7 or 8, by InsB3 and InsB7; InsB3 and InsB7 serve as the C3 alcohol dehydrogenase and the C2′/C3′ enoylreductase, respectively. Subsequently, 9 is accepted by one of the two αKG-dependent dioxygenases InsB4 and InsB5. InsB4 hydroxylates 9 at the C6 position to produce 11, whereas InsB5 performs C3′ hydroxylation to yield 10. Compound 10 is then accepted by InsB4, which performs two rounds of hydroxylation at C1 and C6 to yield 15*via*16. Meanwhile, 11 can also be accepted by InsB4 and InsB5 to be transformed into 15*via*12 or 14. The acetyltransferase InsB1 acetylates the C1 hydroxy group; the resulting acetoxy group then undergoes spontaneous elimination to furnish the C1/C2 double bond, as seen in the *insA* pathway. In the heterologous expression experiment, 16 was accepted by InsB1 and converted to 13; moreover, 15, which was only obtained from the *in vitro* enzymatic reactions, reacted with InsB1 to provide 17. Finally, the αKG-dependent dioxygenase InsB4 accepts 17 and installs an epoxide at C1/C2 to produce 18, which is the apparent end product of the *insB* pathway. Compound 18 can also be derived from 13*via* two rounds of oxidative reactions catalyzed by InsB4, probably *via*17.

Among the tailoring enzymes in the *ins* pathways, the αKG-dependent dioxygenase InsB4 exhibits unusual multifunctionality to install both the 1β- and 6α-hydroxy groups. Although the αKG-dependent enzymes involved in fungal meroterpenoid pathways are often multifunctional and act on more than one carbon atom, they typically perform abstractions of adjacently located hydrogen atoms (Fig. S5†).^5,7,38,39^ In contrast, in InsB4-catalyzed reactions, the two hydrogen atoms abstracted by the enzyme are oriented in opposite directions (Fig. S5†), indicating that InsB4 can accommodate the substrates in two distinct modes. According to the results of the *in vitro* enzymatic reactions, the C3′ hydroxy group is the key determinant for reaction selectivity; when the C3′ hydroxy group is missing, the hydroxylation preferentially occurs at C6 first (*i.e.*, the conversion of 9 to 11), and C-1β hydroxylation is favored after the introduction of the C3′ hydroxyl group (*i.e.*, the conversion of 10 to 16). Currently, it is unclear how the C3′ hydroxy group affects the substrate-binding mode of InsB4. Future structural biology studies are warranted to elucidate the structural basis of the unique reactivity of InsB4.

## Conclusions

Fungal meroterpenoids derived from (6*R*,10′*R*)-epoxyfarnesyl-DMOA methyl ester (1) include many structurally diverse molecules. The first three enzymes that cyclize 1 were identified approximately 10 years ago;^12,13^ since then, no additional terpene cyclases that cyclize 1 in a different cyclization mode had been reported. Our genome mining work reported in this study has led to the discovery of two new cyclases of 1 in a single fungal species. One of these enzymes was found to fold 1 in an unusual pre-boat-chair conformation prior to performing the cyclization reaction. Considering the recently accelerated accumulation of fungal genome sequence data, future genome mining studies will help to identify many more cyclases of 1 with new cyclization modes. Furthermore, because many meroterpenoid cyclases have been characterized in recent years, these cyclases could be rationally engineered according to their sequence comparison, which is underway in our laboratory. In conclusion, our study expands our knowledge of fungal meroterpenoid biosynthesis and demonstrates the usefulness of the genome mining approach in revealing hidden biosynthetic reactions.

## Data availability

Crystallographic data for compounds 4, 5, 6, 8, 11, and 12 have been deposited at the CCDC under 2174818, 2174817, 2174819, 2174822, 2174820, and 2174821, respectively. The other datasets supporting this article have been uploaded as part of the ESI.†

## Author contributions

Y. M. designed the research and conducted the bioinformatic analysis. J. T. performed experiments. Both authors analyzed the data and co-wrote the manuscript.

## Conflicts of interest

There are no conflicts to declare.

## Supplementary Material

SC-013-D2SC02994D-s001

SC-013-D2SC02994D-s002
