# Supplementary material for: Discovery of branching meroterpenoid biosynthetic pathways in Aspergillus insuetus: involvement of two terpene cyclases with distinct cyclization modes
Source: Chem Sci. 2022 Aug 17;13(35):10361–9. doi: 10.1039/d2sc02994d (PMC9473517; doi:10.1039/d2sc02994d)
Supplement: SC-013-D2SC02994D-s001 [file SC-013-D2SC02994D-s001.pdf]

Electronic Supplementary Material (ESI) for Chemical Science.
This journal is © The Royal Society of Chemistry 2022

Supporting Information for

Discovery of branching meroterpenoid biosynthetic pathways in *Aspergillus insuetus*: Involvement of two terpene cyclases with distinct cyclization modes

Jia Tang[a] and Yudai Matsuda*[a]

[a]Department of Chemistry, City University of Hong Kong, Tat Chee Avenue, Kowloon, Hong Kong SAR, China

*Correspondence should be addressed to Yudai Matsuda (ymatsuda@cityu.edu.hk).

Table of Contents

Supplementary Materials and Methods **S2-S11**

Supplementary Tables S1-S7 **S12-S20**

Supplementary Figures S1-S128 **S21-S93**

X-Ray Crystallographic Data **S94-S99**

Supplementary References **S100**

Supplementary Materials and Methods

General experimental procedures

Organic solvents were purchased from Anaqua (Hong Kong) Co. Ltd., and other chemicals were purchased from Wako Chemicals Ltd., Thermo Fisher Scientific, Sigma-Aldrich, or J&K Scientific Ltd., unless noted otherwise. Oligonucleotide primers (Table S3) were purchased from Beijing Genomics Institute or Tech Dragon Limited. PCR was performed using a T100™ Thermal Cycler (Bio-Rad Laboratories, Inc.) with Phanta Max Super-Fidelity DNA Polymerase (Vazyme Biotech Co., Ltd). Analytical HPLC was performed on a Dionex Ultimate 3000 UHPLC system (Thermo Scientific), using a Kinetex 2.6 µm C_{18} 100 Å column (2.1 i.d. x 100 mm; Phenomenex) for the products from in vivo experiments and an Accucore™ C18 column (4.6 i.d. x 100 mm; Thermo Scientific) for the products from in vitro experiments. Preparative HPLC was performed on a Waters 1525 Binary HPLC pump with a 2998 photodiode array detector (Waters Corporation), using an XBridge BEH C18 OBD Prep Column (100 Å, 5 µm, 19 i.d. x 250 mm; Waters Corporation). Flash chromatography was performed using an Isolera Spektra One flash purification system (Biotage). NMR spectra were obtained at 600 MHz (^{1}H)/150 MHz (^{13}C) with a Bruker Ascend Avance III HD spectrometer, and chemical shifts were recorded with reference to solvent signals (^{1}H NMR: $CDCl_3$ 7.26 ppm; ^{13}C NMR: $CDCl_3$ 77.0 ppm). HR-ESI-MS spectra were obtained with micrOTOF-Q II mass spectrometer (Bruker Daltonics) and SCIEX X500R Q-TOF mass spectrometer. Samples for LC-MS analysis were injected into a SCIEX ExionLC AD System with a SCIEX X500R Q-TOF mass spectrometer, using a Luna Omega 1.6 µm C_{18} 100 Å column (2.1 i.d. x 100 mm; Phenomenex). Optical rotations were measured with P-2000 Digital Polarimeter (JASCO Corporation). X-ray diffraction data were collected on a Bruker D8 Venture Photon II diffractometer. UV spectra were measured using Cary 60 UV–Vis Spectrophotometer (Agilent Technologies). CD spectra were obtained with J-1500 Circular Dichroism Spectrophotometer (JASCO Corporation).

Strains

Aspergillus insuetus CBS 107.25 was purchased from the Westerdijk Fungal Biodiversity Institute and used as a source for the cloning of each gene in the *insA* and *insB* clusters. *Aspergillus oryzae* NSAR1 (*niaD*$^{-}$, *sC*$^{-}$, *ΔargB, adeA*$^{-}$)[1] was utilized as the fungal heterologous expression host. Standard DNA engineering was performed with *Escherichia coli* DH5α (Takara Bio Inc).

Generation of *A. oryzae* NSARU1 strain

To generate an *A. oryzae* strain with more selection markers, the *pyrG* gene (AO090011000868) of *A. oryzae* NSAR1 was deactivated by a CRISPR-Cas9-mediated approach. To this end, the components for the dual Cas9-gRNA system, namely Cas9 protein, CRISPR RNAs (crRNAs), and transactivating CRISPR RNA (tracrRNA), were purchased from Integrated DNA Technologies; two separate crRNAs were designed at two different

positions of the *pyrG* gene (protospacer sequences: 5′-CACCAAAGAGCTGCTGGATT-3′; 5′-GAAATTGTGCTTCTCTGCAA-3′). The Cas9 ribonucleoprotein (RNP) complexes were assembled as reported[2] and used for the fungal transformation. The fungal transformation was performed by the previously reported protoplast–polyethylene glycol method.[3] Transformants were selected on M-sorbitol media (0.2% NH_4Cl, 0.1% $(NH_4)_2SO_4$, 0.05% KCl, 0.05% NaCl, 0.1% KH_2PO_4, 0.05% $MgSO_4 \cdot 7H_2O$, 0.002% $FeSO_4 \cdot 7H_2O$, 2% glucose, and 1.2 M sorbitol, pH 5.5) supplemented with 0.1% arginine, 0.15% methionine, 0.01% adenine, 0.2% uracil, 0.5% uridine, and 0.2% of 5-fluoroorotic acid (5-FOA). One of the resultant transformants was selected for further use and hereby named the NSARU1 strain.

Construction of new fungal transformation vectors pPyrG and pPyrG-HR

To construct a fungal expression plasmid containing *pyrG* as a selection marker, the *sC* marker of the pUSA vector[4] was replaced with the *pyrG* gene from *Aspergillus novofumigatus* IBT 16806 (*Aspnov_pyrG*; P174DRAFT_399623) in which the *pyrG* gene is flanked by repeated sequences (Figure S6). The resultant vector was named pPyrG.

Additionally, a variant of pPyrG named pPyrG-HR was created, which is intended for the homologous recombination-based transformation and targets the HS801 locus of *A. oryzae*[5] (Figure S7). Upstream and downstream regions of the target site (~1 kb each) were first amplified from the genomic DNA of *A. oryzae* NSAR1, and then inserted into the pPyrG vector, in which the *pyrG* marker is flanked by the upstream/downstream regions of the HS801 locus.

Primer sequences and a detailed method for the construction of pPyrG and pPyrG-HR are summarized in Table S3 and Table S4. Ligation of DNA fragments to construct the vector was performed using ClonExpress Ultra One Step Cloning Kit (Vazyme Biotech Co., Ltd).

Construction of fungal transformation plasmids

To construct fungal expression plasmids for *A. oryzae*, each gene in *insA* and *insB* clusters was first amplified from the genomic DNA of *A. insuetus* CBS 107.25 with the primers described in Table S3 and Table S4. Upon the constructions of the transformation plasmids, the database sequences of some *insA* and *insB* genes were manually revised (Table S2). Each amplified DNA fragment was then introduced into pTAex3 vector[6] except for *insB1*, while *insB1* was ligated into the pPyrG vector, using a ClonExpress Ultra One Step Cloning Kit (Vazyme Biotech Co., Ltd). For the construction of multigene-containing plasmids, DNA fragments harboring the *amyB* promoter (*PamyB*) and the *amyB* terminator (*TamyB*) were amplified from the pTAex3-based plasmids, and further introduced into the already constructed single gene-containing vector or other vectors, pAdeA,[7] pPTRI,[8] or pPyrG. Detailed methods for the construction of the plasmids used in this study are summarized in Table S4.

Fungal transformation

Transformation of *A. oryzae* was carried out by the previously reported protoplast–polyethylene glycol method,[3] and the transformants created in this study and the plasmids used for the transformation are given in Table S5. For the introduction of *insA1*, *insA2*, *insA4*, *insA5*, and *insA7* or *insB2*, *A. oryzae* NSAR1 was transformed with the two plasmids, pTAex3-insA2+insA5 and pAdeA-insA1+insA4+insA7 or pAdeA-insA1+insA4+insB2, respectively.

The resultant five gene-containing transformant with *insA7* was further transformed using the plasmids, pPTRI-insA8+insA6+insA9, to construct a transformant expressing *insA1*, *insA2*, *insA4*, *insA5*, *insA7*, *insA6*, *insA8*, and *insA9*. To construct the transformants not expressing one of the *insA* genes, plasmids containing only two genes were used upon performing the transformation.

For the introduction of *insB1*, *insB3*, *insB4*, *insB5*, and *insB7*, the *pyrG* gene of the *A. oryzae* transformant harboring *insA1*, *insA2*, *insA4*, *insA5*, and *insB2* was deactivated using the same approach used for the generation of the NSARU1 strain, to increase the number of available selection markers. The resultant strain, designated as *A. oryzae*/*insA1+A2+A4+A5+B2* (*pyrG*$^-$), was further transformed with the two plasmids, pPyrG-insB1+insB5 and pPTRI-insB3+insB4+insB7. To create the transformants lacking one of the *insB* genes, plasmids containing only one gene were used upon performing the transformation.

HPLC analysis of metabolites derived from *A. oryzae* transformants

To analyze the metabolites produced by each *A. oryzae* transformant, the transformants were cultivated on a DPY agar plate [2% dextrin, 1% hipolypepton (Nihon Pharmaceutical Co., Ltd.), 0.5% yeast extract, 0.5% KH_2PO_4, 0.05% $MgSO_4{\bullet}7H_2O$, and 1.5% agar] for seven days at 30 °C. A small piece of fungal mycelia and agar was cut from the plate, soaked in ethyl acetate, and extracted using an ultrasonic bath. The ethyl acetate layer was transferred to a new tube, and the solvent was removed using nitrogen gas flow.

The residue was dissolved in methanol and analyzed by HPLC, with a solvent system of 20 mM formic acid (solvent A) and acetonitrile containing 20 mM formic acid (solvent B), at a flow rate of 0.4 mL/min and a column temperature of 40 °C. Separation was performed using a linear gradient from 10:90 (solvent B/solvent A) to 100:0 for 10 min, 100:0 for the following 3 min, and a linear gradient from 100:0 to 10:90 within the following 2.0 min, and then 10:90 for 2.5 min of equilibrium.

Isolation of each metabolite from *A. oryzae* transformants

To isolate each metabolite, *A. oryzae* transformants were cultivated either on DPY agar plates (the volume of medium in one plate is *ca.* 20 mL) for seven days at 30 °C or in DPY liquid medium at 30 °C/160 rpm for three days. When cultivated on the agar plates, the resulting fungal cultures, including agar medium, were crushed into small pieces, soaked in ethyl acetate, and extracted twice using an ultrasonic bath. After filtration, ethyl acetate was removed in vacuo. When cultivated in the liquid medium, medium and mycelia were first separated

by filtration. The medium was extracted with ethyl acetate, whereas the mycelia were extracted with acetone with sonication for one hour, concentrated, and reextracted with ethyl acetate. Both extracts were then combined. The resultant crude extract was fractionated by flash chromatography, and further purified by preparative HPLC. Purification methods for each compound are described in detail below.

Purification condition for insuetusin A1 (**2**):

The extract of the *A. oryzae* strain with *insA1*, *insA2*, *insA4*, *insA5*, and *insA7* cultivated on 150 DPY agar plates (1090 mg) was subjected to flash chromatography and eluted stepwise using a dichloromethane:ethyl acetate gradient (100:0 to 0:100). Fractions that contained **2** were then purified by reverse-phase preparative HPLC (65% aqueous acetonitrile, 10.0 mL/min) to yield 100.5 mg of **2**.

Purification condition for insuetusin B1 (**3**):

The extract of the *A. oryzae* strain with *insA1*, *insA2*, *insA4*, *insA5*, and *insB2* cultivated in 2 L of DPY liquid medium (1260 mg) was subjected to flash chromatography and eluted stepwise using a dichloromethane:ethyl acetate gradient (100:0 to 0:100). Fractions that contained **3** were then purified by reverse-phase preparative HPLC (55% aqueous acetonitrile, 10.0 mL/min) to yield 108.5 mg of **3**.

Purification conditions for insuetusin A2 (**4**), insuetusin A3 (**5**), and insuetusin A4 (**6**):

The extract of the *A. oryzae* strain with *insA1*, *insA2*, *insA4*, *insA5*, *insA7*, *insA6, insA8*, and *insA9* cultivated on 200 DPY agar plates (1010 mg) was subjected to flash chromatography and eluted stepwise using a dichloromethane:ethyl acetate gradient (100:0 to 0:100). Fractions that contained **4**, **5**, and **6** were then purified by reverse-phase preparative HPLC (55% aqueous acetonitrile, 10.0 mL/min) to yield 50.1 mg of **4**, 13.6 mg of **5**, and 8.3 mg of **6**.

Purification conditions for insuetusin B2 (**7**):

The extract of the *A. oryzae* strain with *insA1*, *insA2*, *insA4*, *insA5*, *insB1*, *insB2*, *insB3*, *insB4*, and *insB5* cultivated on 50 DPY agar plates (305 mg) was subjected to flash chromatography and eluted stepwise using a dichloromethane:ethyl acetate gradient (100:0 to 0:100). Fractions that contained **7** were then purified by reverse-phase preparative HPLC (65% aqueous acetonitrile, 10.0 mL/min) to yield 29.2 mg of **7**.

Purification conditions for insuetusin B3 (**8**), insuetusin B4 (**9**), and insuetusin B6 (**11**):

The extract of the *A. oryzae* strain with *insA1*, *insA2*, *insA4*, *insA5*, *insB1*, *insB2*, *insB3*, *insB4*, and *insB7* cultivated on 100 DPY agar plates (713 mg) was subjected to flash chromatography and eluted stepwise using a dichloromethane:ethyl acetate gradient (100:0 to 0:100). Fractions that contained **8**, **9**, and **11** were then purified by reverse-phase preparative HPLC (65% aqueous acetonitrile, 10.0 mL/min) to yield 10.3 mg of **8**,

30.2 mg of **9**, and 10.1 mg of **11**.

Purification conditions for insuetusin B5 (**10**), insuetusin B7 (**12**), and insuetusin B8 (**13**):

The extract of the *A. oryzae* strain with *insA1*, *insA2*, *insA4*, *insA5*, *insB1*, *insB2*, *insB3*, *insB4*, *insB5*, and *insB7* cultivated on 170 DPY agar plates (1200 mg) was subjected to flash chromatography and eluted stepwise using a dichloromethane:ethyl acetate gradient (100:0 to 0:100). Fractions that contained **10**, **12**, and **13** were then purified by reverse-phase preparative HPLC (60% aqueous acetonitrile, 10.0 mL/min) to yield 70.8 mg of **10**, 24.3 mg of **12**, and 7.2 mg of **13**.

In vivo labeling experiments

For isotope incorporation experiments with sodium [1,2-$^{13}C_2$]acetate, the *A. oryzae* transformants expressing *insA1*, *insA2*, *insA4*, *insA5*, and *insA7* or *insB2* were cultivated on 15 DPY agar plates supplemented with 500 mg/L sodium [1,2-$^{13}C_2$]acetate, at 30 °C for seven days. 9.5 mg labeled insuetusin A1 (**2**) and 13.5 mg labeled insuetusin B1 (**3**) were extracted and purified using the methods described above for these compounds.

LC-MS analysis of metabolites derived from *A. insuetus*

To analyze the metabolites produced by *A. insuetus* CBS 107.25, the fungal strain was cultivated on YES agar plates [2% yeast extract, 15% sucrose, 0.05% $MgSO_4 \cdot 7H_2O$, 2% agar supplemented with 1 mL/L of a trace element solution (10 g/L $ZnSO_4 \cdot 7H_2O$, 5 g/L $CuSO_4 \cdot 5H_2O$), pH 6.5] at 30 °C for seven days. A small piece of fungal mycelia and agar was cut from the plate, soaked in ethyl acetate, and extracted using an ultrasonic bath. The ethyl acetate layer was transferred to a new tube, and the solvent was removed using nitrogen gas flow.
The residue was dissolved in methanol and analyzed by LC-MS, with a solvent system of 20 mM formic acid (solvent A) and acetonitrile containing 20 mM formic acid (solvent B), at a flow rate of 0.4 mL/min and a column temperature of 40 °C. Separation was performed using a linear gradient from 10:90 (solvent B/solvent A) to 100:0 for 10 min, 100:0 for the following 3 min, and a linear gradient from 100:0 to 10:90 within the following 2.0 min, and then 10:90 for 2.5 min of equilibrium.

Expression and purification of InsB4 and InsB5

To express *insB4* and *insB5* in *E. coli*, complementary DNA (cDNA) for each gene was introduced into the pET-28a(+) vector (Novagen), using a ClonExpress Ultra One Step Cloning Kit (Table S3 and Table S4). To obtain the cDNA of *insB4* and *insB5*, total RNA was extracted from the *A. oryzae* strain harboring *insA1*, *insA2*, *insA4*, *insA5*, *insB1*, *insB2*, *insB3*, *insB4*, *insB5*, and *insB7* with *TransZol* Plant (TransGen Biotech Co., Ltd.), and cDNA synthesis was then performed using HiScript III 1st Strand cDNA Synthesis Kit (Vazyme Biotech Co., Ltd).
For the expression of InsB4 and InsB5, *E. coli* Transetta (DE3) was transformed using the above-constructed

pET-28a(+)-based plasmid. The *E. coli* transformants were first cultivated using shaking at 37 °C/160 rpm, in LB medium containing 50 mg/L kanamycin sulfate and 34 mg/L chloramphenicol. When the cultures reached an OD_{600} of 0.55, 0.5 mM IPTG was added to induce the gene expression, followed by further incubation for 20 h at 16 °C/200 rpm. Protein purification was performed as previously described.[3] The purity of each purified protein was examined by sodium dodecyl sulfate polyacrylamide gel electrophoresis (SDS-PAGE) (Figure S3), and the protein concentrations were determined with a NanoDrop™ One^{C} spectrophotometer (Thermo Scientific).

Enzymatic reaction assay of InsB4 and InsB5

Standard enzymatic reactions of InsB4 and/or InsB5 with insuetusin B4 (**9**), insuetusin B5 (**10**), insuetusin B6 (**11**), insuetusin B7 (**12**), insuetusin B8 (**13**), insuetusin B9 (**14**), insuetusin B11 (**16**), or insuetusin B12 (**17**) were performed in reaction mixtures containing 50 mM Tris-HCl buffer (pH 8.0), 0.5 mM of **9**, **10**, **11**, **12**, **13**, **14**, **16**, or **17**, 0.1 mM $FeSO_4$, 2.5 mM α-ketoglutarate, 4 mM ascorbate, 9.9 μM InsB4 and/or 9.8 μM InsB5, in 50 μL reaction mixtures at 30 °C for 2 h. The reactions were terminated by the addition of 50 μL of methanol and vortex mixing. The supernatant obtained after centrifugation was analyzed by HPLC (60% aqueous acetonitrile containing 20 mM formic acid, 1.0 mL/min).

Isolation of each product from in vitro enzymatic reactions

To isolate each product from the in vitro enzymatic reactions, the purified InsB4 (final conc. 10.9 μM) was added to reaction buffer (50 mM Tris-HCl buffer pH 8.0, 0.1 mM $FeSO_4$, 2.5 mM α-ketoglutarate, 4 mM ascorbate) and incubated with 0.5 mM of a substrate at 30 °C. Production and purification methods for each compound are described in detail below.

Production and purification conditions for insuetusin B9 (**14**):

The enzymatic reaction was performed in 23.25 mL of reaction buffer with insuetusin B4 (**9**) overnight. The reaction mixture was loaded onto Sep-Pak C18 plus short cartridge (Waters Corporation) and eluted stepwise using a water:methanol gradient (100:0 to 0:100). The fractions containing **14** were concentrated to yield 5.5 mg of white amorphous solid.

Production and purification conditions for insuetusin B10 (**15**):

The enzymatic reaction was performed in 22.5 mL of reaction buffer with insuetusin B5 (**10**) overnight. The reaction mixture was loaded onto Sep-Pak C18 plus short cartridge (Waters Corporation) and eluted stepwise using a water:methanol gradient (100:0 to 0:100). The fractions containing **15** were concentrated to yield 4.5 mg of white amorphous solid.

Production and purification conditions for insuetusin B11 (**16**):

The enzymatic reaction was performed in 22.5 mL of reaction buffer with insuetusin B5 (**10**) for 1.5 h. The reaction mixture was loaded onto Sep-Pak C18 plus short cartridge (Waters Corporation) and eluted stepwise using a water:methanol gradient (100:0 to 0:100). Fractions that contained **16** were then purified by reverse-phase preparative HPLC (65% aqueous acetonitrile, 10.0 mL/min) to yield 2.1 mg of white amorphous solid.

Production and purification conditions for insuetusin B13 (**18**):

The enzymatic reaction was performed in 13.5 mL of reaction buffer with insuetusin B8 (**13**) overnight. The reaction mixture was loaded onto Sep-Pak C18 plus short cartridge (Waters Corporation) and eluted stepwise using a water:methanol gradient (100:0 to 0:100). Fractions that contained **18** were then purified by reverse-phase preparative HPLC (55% aqueous acetonitrile, 10.0 mL/min) to yield 1.5 mg of white amorphous solid.

Bioconversion experiments

To perform the bioconversion experiment with InsB1, transformation of *A. oryzae* was performed by CRISPR-Cas9-mediated DNA double-strand break and repair by homologous recombination using the pPyrG-HR-insB1 plasmid. To this end, the components for the dual Cas9-gRNA system, namely Cas9 protein, CRISPR RNAs (crRNAs), and transactivating CRISPR RNA (tracrRNA), were purchased from Integrated DNA Technologies; two separate crRNAs were designed for the HS801 locus, respectively (protospacer sequences: 5′-AGTGTTGCAATCCAAGGATA-3′; 5′- AATCAGTCCAAGAACTGCCT-3′). The Cas9 ribonucleoprotein (RNP) complexes were assembled as reported[2] and used for the fungal transformation along with pPyrG-HR-insB1.

The resultant transformant was initially cultivated in 5 mL of DPY medium at 30 ºC and 160 rpm for three days and then transferred to 25 mL of DPY medium containing 16.7 mg/L of **15**. After cultivation at 30 ºC and 160 rpm for three days, medium and mycelium were separated by filtration. The medium was extracted with ethyl acetate, and the extract was analyzed by HPLC with 60% aqueous acetonitrile containing 20 mM formic acid at a flow rate of 1.0 mL/min and a column temperature of 40 °C, using an Accucore™ C18 column (4.6 i.d. x 100 mm; Thermo Scientific).

For the isolation of insuetusin B12 (**17**), the *A. oryzae* transformant with *insB1* was cultivated in 0.36 L of DPY liquid medium containing 22.2 mg/L of **15** at 30 ºC and 160 rpm for seven days. The extract from the transformant (756 mg) was subjected to flash chromatography and eluted stepwise using a dichloromethane:ethyl acetate gradient (100:0 to 0:100). Fractions that contained **17** were then purified by reverse-phase preparative HPLC (50% aqueous acetonitrile, 10.0 mL/min) to yield 2.5 mg of **17**.

Analytical data

Insuetusin A1 (2). Yellowish amorphous solid; $[\alpha]^{22.6}_{D}$ +79.2 (*c* 0.75, $CHCl_3$); UV (MeOH) λ_{max} (log ε) 206 (3.89), 243 (3.80) nm; CD (*c* 0.05, MeOH) λ_{max} (Δε) 195 (−2.8), 209 (+10.5), 228 (−9.8), 268 (+9.7), 315 (+0.5), 338 (+2.0) nm; for NMR data see Figure S8 to Figure S14; HRMS (ESI) *m/z*: $[M + H]^+$ Calcd for $C_{26}H_{39}O_5$ 431.2792; Found 431.2761.

Insuetusin B1 (3). White amorphous solid; $[\alpha]^{22.5}_{D}$ −100.3 (*c* 0.91, $CHCl_3$); UV (MeOH) λ_{max} (log ε) 210 (4.12), 246 (4.04), 287 (3.82) nm; CD (*c* 0.05, MeOH) λ_{max} (Δε) 211 (+13.4), 227 (+7.8), 235 (+8.8), 322 (−7.7) nm; for NMR data see Figure S15 to Figure S21; HRMS (ESI) *m/z*: $[M + H]^+$ Calcd for $C_{26}H_{39}O_5$ 431.2792; Found 431.2756.

Insuetusin A2 (4). Yellowish crystal; $[\alpha]^{22.4}_{D}$ +163.8 (*c* 1.00, $CHCl_3$); UV (MeOH) λ_{max} (log ε) 203 (3.81), 243 (3.73) nm; CD (*c* 0.05, MeOH) λ_{max} (Δε) 196 (−2.7), 209 (+11.7), 228 (−5.2), 269 (+9.9), 316 (+0.4), 339 (+1.8) nm; for NMR data see Figure S22 to Figure S28; HRMS (ESI) *m/z*: $[M + H]^+$ Calcd for $C_{26}H_{37}O_5$ 429.2636; Found 429.2607.

Insuetusin A3 (5). Yellowish crystal; $[\alpha]^{20.6}_{D}$ +87.9 (*c* 0.36, $CHCl_3$); UV (MeOH) λ_{max} (log ε) 203 (3.98), 240 (3.81) nm; CD (*c* 0.05, MeOH) λ_{max} (Δε) 196 (−0.2), 209 (+10.4), 229 (−4.5), 270 (+7.3), 316 (+0.3), 337 (+1.3) nm; for NMR data see Figure S29 to Figure S35; HRMS (ESI) *m/z*: $[M + H]^+$ Calcd for $C_{26}H_{37}O_6$ 445.2585; Found 445.2559.

Insuetusin A4 (6). Yellowish crystal; $[\alpha]^{22.6}_{D}$ +105.1 (*c* 0.59, $CHCl_3$); UV (MeOH) λ_{max} (log ε) 206 (3.87), 229 (3.93) nm; CD (*c* 0.05, MeOH) λ_{max} (Δε) 216 (+7.7), 222 (+7.3), 230 (+8.3), 250 (+1.4), 269 (+4.9), 346 (−0.9) nm; for NMR data see Figure S36 to Figure S42; HRMS (ESI) *m/z*: $[M + H]^+$ Calcd for $C_{26}H_{35}O_5$ 427.2479; Found 427.2450.

Insuetusin B2 (7). White amorphous solid; $[\alpha]^{21.5}_{D}$ −144.8 (*c* 0.96, $CHCl_3$); UV (MeOH) λ_{max} (log ε) 206 (4.05), 246 (3.95), 286 (3.72) nm; CD (*c* 0.05, MeOH) λ_{max} (Δε) 195 (−1.8), 211 (+11.7), 227 (+6.4), 233 (+7.1), 322 (−6.3) nm; for NMR data see Figure S43 to Figure S49; HRMS (ESI) *m/z*: $[M + H]^+$ Calcd for $C_{26}H_{37}O_5$ 429.2636; Found 429.2622.

Insuetusin B3 (8). White crystal; $[\alpha]^{21.1}_{D}$ −232.9 (*c* 0.57, $CHCl_3$); UV (MeOH) λ_{max} (log ε) 199 (3.64), 266 (4.23) nm; CD (*c* 0.05, MeOH) λ_{max} (Δε) 197 (+8.2), 204 (+7.5), 222 (+12.6), 260 (−13.1), 281 (−5.6), 306 (−14.0) nm; for NMR data see Figure S50 to Figure S56; HRMS (ESI) *m/z*: $[M + H]^+$ Calcd for $C_{26}H_{41}O_5$ 433.2949; Found 433.2923.

*Insuetusin B4 (**9**).* White amorphous solid; $[\alpha]^{20.8}_{D}$ −298.4 (*c* 0.81, $CHCl_3$); UV (MeOH) λ_{max} (log ε) 201 (3.68), 267 (4.38) nm; CD (*c* 0.05, MeOH) λ_{max} (Δε) 196 (+10.8), 199 (+10.6), 221 (+17.6), 258 (−18.3), 280 (−8.1), 305 (−19.1) nm; for NMR data see Figure S57 to Figure S63; HRMS (ESI) *m/z*: $[M + H]^+$ Calcd for $C_{26}H_{39}O_5$ 431.2792; Found 431.2772.

*Insuetusin B5 (**10**).* White amorphous solid; $[\alpha]^{21.8}_{D}$ −366.0 (*c* 0.99, $CHCl_3$); UV (MeOH) λ_{max} (log ε) 201 (3.62), 270 (4.23) nm; CD (*c* 0.05, MeOH) λ_{max} (Δε) 222 (+17.4), 264 (−15.2), 290 (−4.2), 319 (−10.5) nm; for NMR data see Figure S64 to Figure S70; HRMS (ESI) *m/z*: $[M + H]^+$ Calcd for $C_{26}H_{39}O_6$ 447.2741; Found 447.2739.

*Insuetusin B6 (**11**).* White crystal; $[\alpha]^{21.3}_{D}$ −198.1 (*c* 0.44, $CHCl_3$); UV (MeOH) λ_{max} (log ε) 202 (3.51), 267 (4.17) nm; CD (*c* 0.05, MeOH) λ_{max} (Δε) 196 (+9.5), 205 (+7.5), 222 (+10.5), 259 (−10.6), 280 (−4.7), 306 (−11.6) nm; for NMR data see Figure S71 to Figure S77; HRMS (ESI) *m/z*: $[M + H]^+$ Calcd for $C_{26}H_{39}O_6$ 447.2741; Found 447.2719.

*Insuetusin B7 (**12**).* White crystal; $[\alpha]^{22.5}_{D}$ −165.8 (*c* 0.79, $CHCl_3$); UV (MeOH) λ_{max} (log ε) 201 (3.58), 271 (4.12) nm; CD (*c* 0.05, MeOH) λ_{max} (Δε) 195 (+8.8), 205 (+7.7), 223 (+12.5), 264 (−10.8), 290 (−2.7), 319 (−8.2) nm; for NMR data see Figure S78 to Figure S84; HRMS (ESI) *m/z*: $[M + H]^+$ Calcd for $C_{26}H_{39}O_7$ 463.2690; Found 463.2674.

*Insuetusin B8 (**13**).* Yellowish amorphous solid; $[\alpha]^{22.0}_{D}$ −358.6 (*c* 0.47, $CHCl_3$); UV (MeOH) λ_{max} (log ε) 201 (3.84), 229 (3.94), 269 (4.18) nm; CD (*c* 0.05, MeOH) λ_{max} (Δε) 213 (+18.3), 259 (−16.8), 291 (−3.4), 318 (−7.7), 365 (+0.9) nm; for NMR data see Figure S85 to Figure S91; HRMS (ESI) *m/z*: $[M + H]^+$ Calcd for $C_{26}H_{37}O_6$ 445.2585; Found 445.2593.

*Insuetusin B9 (**14**).* White amorphous solid; $[\alpha]^{23.2}_{D}$ −138.1 (*c* 0.51, $CHCl_3$); UV (MeOH) λ_{max} (log ε) 201 (3.68), 268 (4.17) nm; CD (*c* 0.05, MeOH) λ_{max} (Δε) 197 (+8.6), 205 (+7.1), 223 (+10.4), 260 (−10.4), 280 (−4.6), 307 (−12.1) nm; for NMR data see Figure S92 to Figure S98; HRMS (ESI) *m/z*: $[M + H]^+$ Calcd for $C_{26}H_{39}O_7$ 463.2690; Found 463.2667.

*Insuetusin B10 (**15**).* White amorphous solid; $[\alpha]^{23.0}_{D}$ −142.4 (*c* 0.41, $CHCl_3$); UV (MeOH) λ_{max} (log ε) 203 (3.98), 273 (4.08) nm; CD (*c* 0.05, MeOH) λ_{max} (Δε) 198 (+7.3), 204 (+6.5), 225 (+8.7), 265 (−9.1), 290 (−2.2), 320 (−7.4) nm; for NMR data see Figure S99 to Figure S105; HRMS (ESI) *m/z*: $[M + H]^+$ Calcd for $C_{26}H_{39}O_8$ 479.2639; Found 479.2619.

*Insuetusin B11 (**16**).* White amorphous solid; $[\alpha]^{22.9}_{D}$ −198.7 (*c* 0.22, $CHCl_3$); UV (MeOH) λ_{max} (log ε) 202 (3.93), 271 (4.28) nm; CD (*c* 0.05, MeOH) λ_{max} (Δε) 200 (+10.5), 203 (+10.1), 223 (+18.7), 262 (−16.5), 290 (−4.0), 319 (−12.1) nm; for NMR data see Figure S106 to Figure S112; HRMS (ESI) *m/z*: $[M + H]^+$ Calcd for $C_{26}H_{39}O_7$ 463.2690; Found 463.2672.

*Insuetusin B12 (**17**).* White amorphous solid; $[\alpha]^{18.8}_{D}$ −170.9 (*c* 0.23, $CHCl_3$); UV (MeOH) λ_{max} (log ε) 202 (3.90), 230 (3.99), 270 (4.20) nm; CD (*c* 0.05, MeOH) λ_{max} (Δε) 216 (+18.0), 263 (−16.5), 291 (−3.6), 318 (−8.2) nm; for NMR data see Figure S113 to Figure S119; HRMS (ESI) *m/z*: $[M + H]^+$ Calcd for $C_{26}H_{37}O_7$ 461.2534; Found 461.2507.

*Insuetusin B13 (**18**).* White amorphous solid; $[\alpha]^{21.0}_{D}$ −89.9 (*c* 0.13, $CHCl_3$); UV (MeOH) λ_{max} (log ε) 203 (3.99), 271 (4.10) nm; CD (*c* 0.05, MeOH) λ_{max} (Δε) 198 (+6.9), 204 (+5.5), 223 (+11.7), 265 (−9.5), 288 (−3.7), 315 (−8.2) nm; for NMR data see Figure S120 to Figure S126; HRMS (ESI) *m/z*: $[M + H]^+$ Calcd for $C_{26}H_{37}O_8$ 477.2483; Found 477.2461.

X-ray crystallographic analysis

Single crystal of compounds **4**, **5**, **6**, **8**, and **11** were grown in CH_3OH/CH_2Cl_2 (2:1, v/v), whereas that of **12** was grown in $CH_3OH/CHCl_3$ (1:3, v/v), by a slow evaporation process at room temperature. Single crystal X-ray diffraction measurements were performed on a Bruker D8 Venture diffractometer using Cu Kα radiation at 173 K (for **4**, **5**, **6**, **8**, and **11**) or 213 K (for **12**). The data collection was performed with the APEX3 program, and cell refinement and data reduction were carried out using the SAINT program. The structures of **4**, **5**, **6**, **8**, **11**, and **12** were solved by direct method with the SHELXT program and refined using the SHELXL program. All non-hydrogen atoms were refined anisotropically, whereas hydrogen atoms were placed by geometrical calculations. Absolute configuration of **4**, **5**, **6**, **8**, **11**, and **12** was determined by the Flack parameters.

Table S1. Annotation of each gene in the *insA* and *insB* clusters from *Aspergillus insuetus* CBS 107.25.

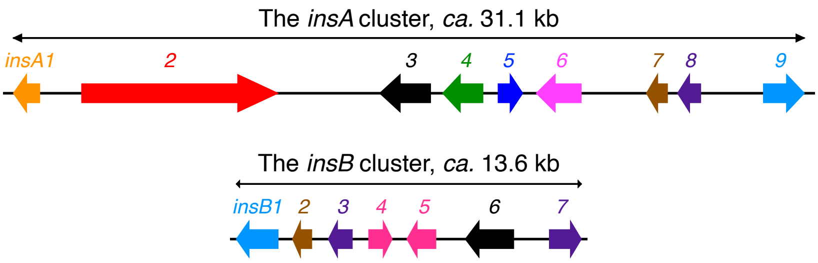

Gene	Protein ID	Amino acids (base pairs)	Protein homologue (origin)	Similarity/Identity (%)	Proposed function
insA1	312027	279 (1019)	AusD (*Aspergillus calidoustus*)	91/86	methyltransferase
insA2	320703	2484 (7687)	AusA (*Aspergillus calidoustus*)	91/84	non-reducing polyketide synthase (SAT-KS-AT-PT-ACP-CMeT-TE)
insA3	320702	619 (1980)	AusY (*Aspergillus calidoustus*)	90/83	major facilitator superfamily transporter
insA4	298416	479 (1564)	AusM (*Aspergillus calidoustus*)	93/88	FAD-dependent monooxygenase
insA5	320701	316 (951)	AusN (*Aspergillus calidoustus*)	93/86	prenyltransferase
insA6	320699	494 (1748)	AusG (*Penicillium brasilianum*)	70/52	cytochrome P450 monooxygenase
insA7	298413	244 (810)	AtmB (*Aspergillus flavus*)	61/44	terpene cyclase
insA8	312020	297 (894)	Bsc3 (*Alternaria brassicicola*)	52/36	short-chain dehydrogenase/reductase
insA9	312019	529 (1590)	EasN (*Aspergillus fumigatus*)	52/34	acetyltransferase
insB1	315383	528 (1655)	AusP (*Penicillium brasilianum*)	54/41	acetyltransferase
insB2	315384	239 (767)	AdrI (*Penicillium roqueforti*)	65/47	terpene cyclase
insB3	315385	257 (955)	AdrF (*Penicillium roqueforti*)	78/66	short-chain dehydrogenase/reductase
insB4	315386	311 (936)	PrhA (*Penicillium brasilianum*)	66/46	Fe(II)/αKG-dependent dioxygenase
insB5	315387	279 (1152)	NvfI (*Aspergillus novofumigatus*)	64/48	Fe(II)/αKG-dependent dioxygenase
insB6	317435	595 (1916)	PrhG (*Penicillium brasilianum*)	73/55	major facilitator superfamily transporter
insB7	300868	338 (1270)	NvfM (*Aspergillus novofumigatus*)	79/66	short-chain dehydrogenase/reductase

Note: Protein IDs are as designated in the JGI database. The sequences of *insA2*, *insA9*, and *insB2* were manually revised in this study (see Table S2).

Table S2. Revised DNA and protein sequences. Highlighted in magenta are the regions predicted for an intron in this study.

Gene	Predicted/revised DNA sequence	Predicted/revised protein sequence
insA2	ATGGGATCGCAGAGTCCATGTTCAGAACATCAGCCTCCTGTGAGCGTGTTCTTTGG<br>ACCGGTCTACCCAGAGTTAACAGAGTCGAGTTCGCATATTCGACAATATCTCTCCGA<br>TGAAGGATCTGCAGGATGGCTCGACGATACCCTACAGGGGCTGCCATCTGTCTGG<br>GAGGATATCGTGAGACAATGGCCAGCTCTTAGGAAAACATCAGGGGAGCCACAGCT<br>CAGACAGCTGACACAATACCTACGCCGCGAGTCATCCAGCCCCGTTAGGGAGAAC<br>CTGAACCTGCTGCTTGTTCCGGTTACGGTGTTGCGGCACATTGTGGAGTTCCGGA<br>AGCTCAAAGATGAGAGAAAAAACCTCGAGATCAAAGATGTCCAAGGGTTTTGCGTT<br>GGAGTGCTTGCTGCAATTACAGTATGCTGGGAGCACGACGATGTGGATTTTGCGAA<br>GGTTGTCAGCACCGTTCTACGGGTGGCTGTGTGTATTGGCGCACTGGTCGACTTA<br>GATGAACTCCACGGAGCCCCTTCCAAGTCCATGGCTGTACGATGGAAGACGAAATG<br>CGAGAACAGACAGCTTGGGGAAGTGTTGGAACGGTACAAGGTGTGGCTATCGACC<br>TTCTAAATTCCTACCGGCTGCTGATAAGAGGAAATAGGGGTATATCGCGTGTATGATC<br>AAGACAAACGGCGCAACAGTTACAGTCCCATCCGAAAACTACAGGTCCGTGACAGA<br>GGATCTTGAGAGCTACGGGATCTCAGTCAAGAGCATCCCTCTCCGTGGGAGGTTC<br>CATACCCCCGATCACATCCCTGCTATGGAGCAGTTGCTGGCGCTCTGCGCGGGAG<br>ACGCTCGTTATCAACTGCCAATCAAGAAAAATCCACATTTATTGCCCAGGTCTAACG<br>TAGACGGCACTCGGATTCCGAGCAATTCGCTGGTTGCAGTTGCGGTCGAGTCCATT<br>TTGGCCAAGCAGGCCAACTGGATGCTCACAGTGGCCGAGGCTCTCAACAGCGAC<br>GGGCCCGCAGACGAGAAACACGCTGTGATCATCGGAGCAGGACAGATCATACCTC<br>AACGATCCTTCTTGGCCAGTGTTGAGCATATAGGAAACCAGATGGCGCCCAATGAC<br>ACTTCTCCTCCTCTGCCAAATGCAGCACTAGATCTACGCTCCACCGCGCAGTGCAA<br>CGGAGCTTTCCCGAAGCCTACGACCCGGGCTTTGTCCACGCCAATCGCCGTTACA<br>GGATTTGCGTGTCGGTACCCGCAGGCAGACTCGGTCGAAGCACTCTGGACGCTCC<br>TTGAGCGCGGCCAGTGCACCGTAAGCCCCATGCCCAACCACCGGCTCAAAGCGG<br>ATAGCCTGCAGCGGCAACCAGCAGGACCGTTCTGGGGCAACTTTCTGCAAAGTCC<br>TGAAAGCTTCGATCATCGCTTCTTTGGCGTCTCGGCCCGCGAAGCAGAGTCTATGG<br>ATCCGCAGCAGAGACTCCTGCTCCAAGTTGCGTACGAAGCCATCGAGTCGGCCAC<br>CTACTGTGGCCTACGAAATACGGAGCTGCCAGATGATGTTGGTTGCTACGTCGGTG<br>TAGGAACGGACGATTACAGCGAGAACGTGGGCTCTCACGACGCGACAGCCTTCTC<br>AGCGACGGGGACGCTGCAGGCCTTCAACAGTGGCCGCATCAGCCATTTTTTTGGG<br>TGGACCGGACCTTCTATCACCGTCGATACGGCTTGTTCTTCTGCGGCGGTGGCTAT<br>TCACCTTGCGTGCCAGGTTAGCACACCTTTTCTTGTTCAGCGAGAGTTAACCGACG<br>AGTACTAATTGCTGGGGGTGTAGGCACTGCACACAAATGATTGCTCGGTTGCCGTT<br>GCTGGCGGAGTCAACGTCATGACAGACCCTCGCTGGTCACAGAACCTCGCCGCC<br>GCCTCCTTTTTGTCTCCAACAGGTGCCTCGAGGGCGTTTGATGCGGCTGCTGATG<br>GGTACTGCCGCGGGGAAGGGGCCGGCCTATTGGTTCTCCGCCCCTTGGACGCTG<br>CGCTTCGGGACGGTGATCCGATCCATGCGGTGATTACAGGAACCTGCGTCAACCA<br>GGGCGCCAACTGCTCTCCAATCACTGTGCCGGATTCCAATTCCCAAAGGAGTCTGT<br>ACATGAAAGCCCTGGCGCAGTCTGGATTGCACCCGGATGCCGTCAGCTATGTGGA<br>AGCGCATGGAACGGGTTCGTACACTGTCTGTTTGCTCGAGCTTGACTGACATCTGG<br>ATAGGAACCCAGGTCGGTGATCCGATCGAGTACGAGAGTATCCGCAGCACGTTTGG<br>CGGGCCACAGCGAACAGAAAAGCTCCACATTGGATCCATCAAAGACAACATCGGG<br>CACACCGAAACCTCATCTGGGGCTGCGGGCATGCTCAAGACGATCTTGATGATTCA<br>GAAGCGACGAATCCCAAAGCAGGCCAACTTCTCGCGCTTGAACCCGCGGATTGTC<br>ACCCACGAGCGAGACCAGATTGCGATTCCGACCCAGTCGCTCGACTGGAAAGCAG<br>CGGAGCGTGTGGCTCTGGTCACCAACTACGGAGCTGCTGGAAGCAATGCTGCCAT<br>TGTTCTAAAACAGCCGGGGCGTGCGTCAAATGAACCAGCCGTTGATCGCTCGCGA<br>TGGCCTGCGCGGGTCCCGTTTATCATCACGGCCAAAACAGAAGAATCTCTGCGCG<br>AATATTGCCGCGAGCTTCAGCACATCCTGCGAGCAGAGCAGCAAGAGAGCCCGGC<br>CGCGACTCACCACCTGGCATACAATCTAGCTGCAAAACAAAACAGGGGCCTGGAAT<br>ACCTGGTCTCGTTCTCTTGTGAGCAAGCAGAGGTCTCAGCTCGGCTGCAAGACAT<br>CGCGGATTGCCGTTCGAAGCCCGTGAGATGCATTCAGCCTCCACCCACAATTGTCC<br>TCTGTTTCGGTGGACAGACCGGGGATATGGCGGGTATTTCACCTAGTCTCGTCGAA<br>AACTGTGATATTCTGCGGTCCCATTTGGTGAGTATCGTCCCTCAGCTGCACTCCAGT<br>CCGCGTGTGGTGTACTTCAACCTGCTGACCGTCTGCAATAGACGGACTGTGATGAA<br>ACTTGTCACACTCTCGGCCTCCCTGGTTTGTTCCCCACAATATTCAGTCCAGAACC<br>GAGGAGGGACCTGGTCAGCCTACATTGTATACTCTTCTCAATCCAGTACGCATCGG<br>CCAAGGCATGGCTCGACTGTGGCCTCGTGGTGGACCGAATGATTGGCCACAGCTT<br>CGGCCAGTTAACCGCCATTTGTGTCGCCGGTGGCCTGTCGCTCATCGATGGCTTG<br>CGTTTGATCTCACAGCGGGCGGCGCTCATCCAGGAGAAATGGGGCAGCGAGCGC	MGSQSPCSEHQPPVSVFFGPVYPELTESSSHIR<br>QYLSDEGSAGWLDDTLQGLPSVWEDIVRQWPA<br>LRKTSGEPQLRQLTQYLRRESSSPVRENLNLLLV<br>PVTVLRHIVEFRKLKDERKNLEIKDVQGFCVGVL<br>AAITVCWEHDDVDFAKVVSTVLRVAVCIGALVDL<br>DELHGAPSKSMAVRWKTKCENRQLGEVLERYK<br>GYIACMIKTNGATVTVPSENYRSVTEDLESYGIS<br>VKSIPLRGRFHTPDHIPAMEQLLALCAGDARYQL<br>PIKKNPHLLPRSNVDGTRIPSNSLVAVAVESILAK<br>QANWMLTVAEALNSDGPADEKHAVIIGAGQIIPQ<br>RSFLASVEHIGNQMAPNDTSPPLPNAALDLRST<br>AQCNGAFPKPTTRALSTPIAVTGFACRYPQADS<br>VEALWTLLERGQCTVSPMPNHRLKADSLQRQP<br>AGPFWGNFLQSPESFDHRFFGVSAREAESMDP<br>QQRLLLQVAYEAIESATYCGLRNTELPDDVGCYV<br>GVGTDDYSENVGSHDATAFSATGTLQAFNSGRI<br>SHFFGWTGPSITVDTACSSAAVAIHLACQALHTN<br>DCSVAVAGGVNVMTDPRWSQNLAAASFLSPTG<br>ASRAFDAAADGYCRGEGAGLLVLRPLDAALRDG<br>DPIHAVITGTCVNQGANCSPITVPDSNSQRSLYM<br>KALAQSGLHPDAVSYVEAHGTGTQVGDPIEYESI<br>RSTFGGPQRTEKLHIGSIKDNIGHTETSSGAAGM<br>LKTILMIQKRRIPKQANFSRLNPRIVTHERDQIAIP<br>TQSLDWKAAERVALVTNYGAAGSNAAIVLKQPG<br>RASNEPAVDRSRWPARVPFIITAKTEESLREYCR<br>ELQHILRAEQQESPAATHHLAYNLAAKQNRGLEY<br>LVSFSCEQAEVSARLQDIADCRSKPVRCIQPPPT<br>IVLCFGGQTGDMAGISPSLVENCDILRSHLTDCD<br>ETCHTLGLPGLFPTIFSPEPRRDLVSLHCILFSIQ<br>YASAKAWLDCGLVVDRMIGHSFGQLTAICVAGGL<br>SLIDGLRLISQRAALIQEKWGSERGVMLSLKASEI<br>QIQELLRAASDTVDVACFNGPQSFVLAGDEKSIA<br>HVETLCVQRGLQHQKKRLRNTHAFHSRLVDPLL<br>PGLSQVAETLDYRPLRIPVEACSEEPDHWARITP<br>FKIVRHSRDPVYFHPAVQRVRRHIPGSCVWLEA<br>GSGSPIVGMVRRVVEAAGPAGEHTYLPMELQD<br>STAEGNLADVAKVLWSKGVPVQFWPFHRSQVG<br>HKWINLPPYQFSKTQHWIDYDPYAFHPTGAVAE<br>EKKNNDGLLRLVKQEANGCLFRINNQDAAYRMC<br>TEGHAVVDQNLCPASLYVEIVVRGAMTLSTSGQ<br>PATMAHIEALNISAPLVVDMPGSVSLRLTRTAKDN<br>DGGWMFSLYSQDGDCPSITHATGKVLLVPQSTG<br>SPASARFHSLNRLLDPGQFDSMAKSPSSNGLKR<br>ATVYQAFRRAVNYADYYRGVVEVYAVGSKAAGR<br>VLLPASPTRMAACDPILIDNFLQVAGIHVNCLSET<br>DADEVFVCSSVGEVSLGDRFLNRDTATPKAWTV<br>YSTYERESEKKVTCDVFALDEDRTLAMTIMSATF<br>TSVSIQSLKRTLSRLNGQTPALSSSSSVGQQPP<br>QPQPKVHEQIAPPAHITISDNDNLRDVQAMLGEL<br>LGVSPGELPSKASLVEIGVDSLMSTEVLAEVDKR<br>FGVKITNSELTDIADVRALAYRIFPSSSSVVHVET<br>FKESTVAIDISIGGQKPIVDSSPIVHQEDSLQFAD<br>SALTAFASTRGSTKHTDQTQFAGFCTSVYPRQM<br>QLVTAYVVEAFQALGAKLESMLPGQAVPSLAILP<br>QHTQVLGQLISVLEHAGLVERKGTDVFRTTKPV<br>DVGPSAVLHQTILADYPQHASEHKLLHTTGARLA<br>ECLTGTADPLSLLFQDAQARALMQDVYSNAPMF<br>KAATMQLAQYLQNLLLGSGCDRDIEILEIGAGTG<br>GTTAFLVSQLAAIPGVKFTYTFTDLSSSLVTLARK<br>RFGSYSFMRYSTLDIEKIPGEELLGKYDIILSSNCI

	GGGGTCATGCTTTCGCTCAAAGCCAGTGAGATCCAGATCCAGGAGCTGTTGCGCG<br>CCGCTTCGGATACTGTGGATGTTGCCTGTTTCAATGGACCGCAGAGCTTTGTGCTC<br>GCCGGGGATGAAAAGTCAATTGCACATGTGGAAACGCTTTGTGTGCAGAGGGGGC<br>TTCAACACCAGAAGAAGCGCCTGAGAAATACACATGCATTTCATTCCCGCCTTGTG<br>GATCCCCTGCTTCCAGGACTGTCCCAGGTAGCAGAGACGCTGGACTATAGGCCAC<br>TTCGCATCCCCGTCGAGGCGTGCTCTGAGGAGCCGGATCATTGGGCGCGTATTAC<br>ACCATTCAAAATCGTCCGACATTCCCGCGACCCAGTGTACTTTCACCCTGCAGTGC<br>AGCGTGTGCGGAGACATATTCCCGGTTCTTGTGTGTGGCTGGAAGCCGGCAGCGG<br>GTCTCCCATTGTGGGGATGGTGCGGCGTGTCGTGGAGGCGGCAGGCCCGGCCG<br>GAGAGCATACCTACTTGCCGATGGAGCTACAGGATTCTACAGCTGAGGGAAACCTG<br>GCCGATGTGGCCAAGGTGCTCTGGTCAAAGGGCGTTCCCGTGCAATTCTGGCCCT<br>TCCATCGGTCGCAGGTGGGACACAAATGGATCAACCTCCCACCCTATCAATTCAGC<br>AAAACGCAGCATTGGATCGACTACGATCCATACGCCTTTCACCCAACAGGTGCAGT<br>TGCAGAGGAGAAAAAGAATAACGACGGCCTCCTGCGACTTGTGAAACAAGAGGCA<br>AATGGTTGTCTGTTCCGTATCAACAATCAAGATGCTGCCTACCGTATGTGTACCGAG<br>GGGCATGCGGTCGTGGACCAGAATCTCTGCCCAGCGTCGCTGTACGTCGAAATCG<br>TAGTCAGAGGCGCCATGACCTTGTCAACCAGTGGGCAGCCAGCGACAATGGCTCA<br>CATTGAAGCGCTCAACATCTCGGCGCCTCTGGTGGTGGACATGCCAGGATCCGTG<br>TCCCTGAGGTTAACGCGGACTGCAAAGGACAATGATGGGGGGTGGATGTTCTCCC<br>TGTACAGCCAGGACGGTGACTGCCCTTCTATCACGCATGCCACAGGCAAGGTTTTG<br>CTTGTGCCCCAATCAACCGGATCTCCGGCATCTGCCCGCTTTCACTCATTGAACCG<br>CCTACTGGACCCCGGTCAATTCGACTCGATGGCAAAATCACCTTCATCCAATGGCC<br>TCAAGAGAGCGACGGTCTACCAGGCCTTTCGTCGAGCTGTCAACTACGCCGACTA<br>CTATCGCGGCGTTGTGGAAGTCTACGCTGTCGGTTCCAAGGCAGCCGGGCGAGTG<br>CTCCTGCCTGCATCCCCCACGCGAATGGCCGCGTGTGACCCTATCCTCATAGACAA<br>CTTTCTCCAGGTCGCGGGGATCCACGTCAACTGCTTGTCTGAAACGGATGCGGAT<br>GAGGTCTTTGTATGTAGCTCCGTCGGTGAAGTGTCATTGGGAGATCGATTTCTCAAC<br>AGGGACACTGCAACCCCCAAGGCGTGGACAGTCTACTCGACCTATGAGCGTGAGT<br>CGGAGAAAAAGGTGACGTGTGATGTATTTGCACTAGATGAGGATCGCACCCTAGCT<br>ATGACCATCATGTCTGCCACGTTTACAAGCGTCTCGATCCAGTCTCTGAAGCGGAC<br>GTTGAGCAGGCTGAATGGCCAAACTCCAGCACTGAGTTCCTCCAGCTCGGTGGGA<br>CAGCAACCGCCTCAGCCTCAGCCAAAAGTTCATGAGCAAATCGCACCGCCTGCCC<br>ATATCACGATTAGCGACAATGACAATCTTCGCGATGTCCAGGCGATGCTAGGTGAGC<br>TGCTGGGCGTTTCGCCTGGGGAGCTTCCCAGCAAGGCATCGCTCGTGGAGATTGG<br>CGTTGACTCGCTGATGAGCACCGAGGTCCTGGCTGAGGTGGACAAGCGCTTTGG<br>GGTCAAGATAACCAACTCGGAGCTGACAGACATTGCAGATGTTCGTGCACTCGCCT<br>ACCGCATCTTCCCAAGTTCATCGTCAGTAGTCCACGTCGAGACCTTCAAAGAATCTA<br>CTGTAGCAATCGACATCAGTATCGGTGGGCAGAAGCCGATCGTCGACTCTTCCCCC<br>ATTGTGCACCAGGAGGATTCGCTCCAGTTTGCGGACAGCGCCCTCACAGCCTTTG<br>CGAGTACTCGAGGAAGCACAAAGCACACCGACCAGACGCAATTTGCTGGCTTCTG<br>CACATCAGTTTATCCTCGGCAGATGCAGCTCGTCACAGCATATGTGGTCGAGGCGT<br>TCCAAGCGCTTGGTGCAAAACTTGAATCGATGTTGCCCGGCCAGGCCGTACCGAG<br>TCTTGCAATCCTCCCGCAACACACTCAGGTGCTGGGCCAGCTGATCAGCGTGCTC<br>GAGCACGCTGGGCTTGTCGAGCGAAAGGGTACAGATGTTTTCCGCACCACCAAAC<br>CAGTTGATGTTGGTCCATCCGCGGTGCTGCACCAGACAATCCTCGCCGACTATCCT<br>CAGCACGCTTCAGAGCACAAGCTGCTGCACACAACTGGCGCACGGTTGGCCGAG<br>TGTCTAACTGGCACGGCTGATCCCTTGTCGCTTCTCTTCCAGGATGCACAGGCTCG<br>CGCTCTCATGCAAGATGTGTACTCAAATGCGCCCATGTTCAAAGCCGCCACGATGC<br>AGCTGGCGCAGTATCTCCAAAACCTGCTGCTTGGCTCTGGATGCGACAGAGATATT<br>GAAATTCTGGAGATCGGTGCGGGAACCGGTGGTACAACTGCTTTTCTTGTGTCGCA<br>GCTTGCAGCGATACCCGGGGTGAAATTCACCTACACTTTCACAGACCTATCCTCATC<br>GCTGGTTACTTTGGCACGCAAAAGGTTCGGCTCATACTCGTTTATGCGCTACTCCAC<br>GCTCGACATCGAAAAGATACCCGGCGAAGAATTGCTTGGGAAGTACGACATCATTC<br>TGTCGTCAAATTGTATCCATGCCACGCGCAGCCTGGCTACCTCGTGTACACACATTC<br>GGAAAATGCTGCGCCCGCACGGTATTCTCTGCCTGATTGAGCTGACCCGAAACCT<br>GCCATGGTTCGACCTCGTCTTTGGTCTCCTGGAAGGATGGTGGCTGTTCAACGAC<br>GGGCGTTCCCATGCCCTTGCCAACGAGAGTCTTTGGCAGGCAAGGCTCCGAGAAG<br>CAGGGTTCAACTGGGTGGACTGGACGGACAACGCGCTGGAAGAGTCTGATATCCT<br>GCGCCTGATTGTGGCGTCAGCTACAAGACCATCTACTGCATTACCCTTGGGACCAT<br>CCGTCGCGCCCGCCCGGGTCGAGACAGTGAAATACGCTGAAAGGGATGGGCTGC<br>AGCTGATGGCCGACATTTACTACCCTCATTCCATTGATCCAAAGGGGACCAAACGG<br>CCCATTGCTCTGCTCATCCACGGCGGCGGCCATATCATGCTCTCTCGCAAGGACAT<br>CCGACCTGCTCAGGTTGACCTTCTTCTCGACGTAGGATTCCTGCCTGTTAGTATTGA<br>CTATCGCTTGTGCCCCGAAGTATCCCTCCTCGAGGGCCCCATGCCGGACGTCCGG<br>GATGCGCTCGCCTGGGCACGCACCGACCTCCCACATCGTCCCCTCAGCCGGAGC<br>GACGTCCAGTCGGATGGGGACCACGTTGTGGCAGTCGGTTGGTCGACGGGAGGT<br>CATCTCGCCATGACCCTGGCCTGGACCGCTCCTGAACACGGCATCCGTCCGCCAC<br>AAGCCATCCTCGCCTTCTACGGTCCTACAGACTATACCGATCCGTTCTGGACGACA<br>CCAAATTTTCCGTACGCCGGGGCCGTCTCGGAAGAACACACCAAGTTGACGCGAC	HATRSLATSCTHIRKMLRPHGILCLIELTRNLPWF<br>DLVFGLLEGWWLFNDGRSHALANESLWQARLR<br>EAGFNWVDWTDNALEESDILRLIVASATRPSTAL<br>PLGPSVAPARVETVKYAERDGLQLMADIYYPHSI<br>DPKGTKRPIALLIHGGGHIMLSRKDIRPAQVDLLL<br>DVGFLPVSIDYRLCPEVSLLEGPMPDVRDALAW<br>ARTDLPHRPLSRSDVQSDGDHVVAVGWSTGGH<br>LAMTLAWTAPEHGIRPPQAILAFYGPTDYTDPFW<br>TTPNFPYAGAVSEEHTKLTRPLDALHDSPITAYNP<br>PPNKQALGGWMAPSDPRSQIALHMNWTGQALS<br>VLFNGCNYKKLAAAKGHSAGEVTLPAPPLADIQ<br>RACPLSQIVAGRYRTPTFLIHGSLDDLIPVEQAQ<br>RTQDALRAAGVESTLRVVEGGLHLFDLGIELETN<br>GTTGSMVDEEGWRAVREGYDFLRQHVAV*

	CTCTCGACGCGCTCCACGATAGCCCCATCACCGCGTACAACCCGCCACCGAACAA<br>GCAAGCCCTGGGCGGGTGGATGGCACCCAGCGATCCACGCAGTCAGATCGCTCT<br>GCACATGAATTGGACGGGCCAGGCGCTCTCCGTGCTGTTCAACGGGTGCAACTAC<br>AAGAAGCTCGCTGCTGCCAAGGGCCATAGCGCCGGTGAGGTGACCCTCCCTGCA<br>CCTCCGCTGGCCGATATCCAGCGCGCCTGTCCGCTCTCGCAGATTGTTGCGGGAC<br>GCTATAGAACGCCTACTTTCCTGATTCATGGTAGCTTGGATGACCTTATCCCTGTGG<br>AACAGGCGCAGCGTACCCAGGATGCCCTGCGCGCTGCAGGGGTGGAATCTACCCT<br>CCGGGTCGTCGAGGGTGGCCTGCATCTGTTTGACCTTGGTATCGAACTGGAGACA<br>AATGGGACAACGGGATCCATGGTGGATGAAGAGGGTTGGAGAGCTGTGCGTGAAG<br>GCTACGACTTTTTGAGGCAGCATGTTGCCGTCTAA	
insA9	ATGAATGCACTATTACTACCGTGGTTAACACCCAATATGCCTGCTCCCCAACCTTTCC<br>CGCCCTTTGTTCTTACTCCTATTGATCACACAACATCCCCTAACACCTATATTTCTTAC<br>CACCTCCTGTTTTCCTTAACGGAGCCGCATACTTCCCTTTGTGATATACAGTCCGGA<br>GTCGAGAGCTTGATACGCAATATTCCATTCTTGGCTGGTGAAATTGGTTATTCAACTA<br>CGACCTTTATTAAAGGGCAACATGCACTAGAAGTCAGACCGTCCTCAGTGCTTATGA<br>AAAGCATACCATTGGTTCAGATAAAATACTTTCCAAAACATATCTTGCCAATTGAGGA<br>TAGAAGCCAGATCAGCACAGAAGGCTTGACGGAGGCTGTCATAAACGAATCTTTGT<br>CGCCACTGCAAATCCAGCTTAGCCTTCCACTGCCCAGACCAGTTTTGCGCTTCCAG<br>GCAAACGTCCTGGCAGATGGAATTATCCTCACCATGGTTTTTGACCACGCTGTTTTC<br>GATGGGACCGGCGGTGGTGTTATTCTTGAGCGTCTGGCTGAATGTTGTCAGAATCC<br>GAACGTCAATCTTACTACGGTCGATAACAGCGAAGCTGAATTAAGATCTACGGTTTG<br>CAGACTTGGGGAAGCTTCGTGTGCGACCCACGAGTTCAACCGATTATTCGCTTCTC<br>GTGAAGTCCCTACGAGCGTCGATACGCAACTAGTGTCGGGAACCTCATTACCAAAG<br>ATTACCACTCGAGCATTTACATTCTCGTCGGACAGAATTCAAACCCTAAAGTCCGCC<br>TGCAATAGGCTTCTCCCTTCGTTATTATATGCTGGAGCAGGCCACCCGACGAGTTTG<br>TTCCTTCCTCCGACGTTTCTTCCAAAATACCTTTCAAGCAGCGACATCGTGACTGCG<br>CTTGTTGCGTTGGGTATCAACCGGGCTAGAGATTGCGGCGATAGGCTCTCAAATCC<br>CACCAAGCTTATGATTGCGGTTAACTTCCGTGAAAGACTTCGGCCTCCCCTACCTAA<br>GGACTATTTTGGAAATGCAGTCACCCAGATCCACAAACAAGTGTCTTCACAAGGTC<br>CCCAAATAGAGCTTGAAAAGGGCTTTTCTGCTGGAGACAAATTTCTAGACAGGAAT<br>GTTGCCCGCGTAGCATGCGTTGCGCAACTTGCAGCTCAGCTTCGTTCCGGATTGAT<br>GTCTATCGATGATAGCTATATCCGGAGTCTCGTGTCCTACATTTCCCAGAATCATGGG<br>GGGGGAGGCTCCCAGGTAGTCCTGACTGATACTATTGTCACTAGCTGGCGTCATCT<br>GAGCGTGTACAACCTGGATTTCGGTGTGAGATTAGGGCGGATTGTTAGATTCCTTC<br>CCCCTATACCCTCCTTTGATGGTGTATGTTGTCTCCTACCTGCACGGTCATTGGAGC<br>GGTTGACCAACAGTCCCATTACAAATGCTTCGAAAGCTCCTTGGGATATGCAAATCT<br>CGCTCGAGTCAAGTGCAATGGCATCTTTCGTGGAAGATGACTTCACTCTATGGGCA<br>TGCGGAAATTCTCTGGAAAGTATAAAGGATAGCATGGGGCCAGATATCGTATGCTAA	MNALLLPWLTPNMPAPQPFPPFVLTPIDHTTSPN<br>TYISYHLLFSLTEPHTSLCDIQSGVESLIRNIPFLA<br>GEIGYSTTTFIKGQHALEVRPSSVLMKSIPLVQIK<br>YFPKHILPIEDRSQISTEGLTEAVINESLSPLQIQL<br>SLPLPRPVLRFQANVLADGIILTMVFDHAVFDGT<br>GGGVILERLAECCQNPNVNLTTVDNSEAELRST<br>VCRLGEASCATHEFNRLFASREVPTSVDTQLVS<br>GTSLPKITTRAFTFSSDRIQTLKSACNRLLPSLLY<br>AGAGHPTSLFLPPTFLPKYLSSSDIVTALVALGIN<br>RARDCGDRLSNPTKLMIAVNFRERLRPPLPKDY<br>FGNAVTQIHKQVSSQGPQIELEKGFSAGDKFLD<br>RNVARVACVAQLAAQLRSGLMSIDDSYIRSLVSYI<br>SQNHGGGGSQVVLTDTIVTSWRHLSVYNLDFG<br>VRLGRIVRFLPPIPSFDGVCCLLPARSLERLTNSP<br>ITNASKAPWDMQISLESSAMASFVEDDFTLWAC<br>GNSLESIKDSMGPDIVC*
insB2	ATGAGTGCATCAGATGTGTTTATTGGCCCTTGGGATCTCCATACAATAGATATCAGTC<br>TCCGATGGGTGGCTTTTATCTGCTGGAGTTTAAACTATATCAGTCTACTAGGCACCG<br>CGATCCGTGATCGCACACCTAGCATGGCACTGCTGGCGCTCTGCTCAGACACGGG<br>CTGGGAGATAGTCTACGGATTCATATTCCCAGAGGCAAGTCGTCATTTTGGCAGCG<br>GCGTCCGCGTCTGGCTGCTACTACATGTGCCTGTGGTCTATGTCATGCTCAAGTTT<br>GGAGCAGACGAATGGGACCACAACCCCTTAGTGAAGAAGAATCTGCCCCTCGTGT<br>ATGTGGCTCTGACCTTTGGGTTCGGAGCGGCCCAGATGGCCCTGGCAAACGAGAT<br>TGGACCGGACCTGGGCTTTTTCTTCGGTGGCGTCTTCTGTCAAACCCTCTTAATATT<br>CAGCCATTTATGTCAGCTCCTCAGCCGGGGGAGCACTCGCGGCGCGTCGTATTCG<br>ATCTGGTGGGTAATCTATGACGTTGGGCTAGACTTCGGCTAACCATCTAAAGGTTCT<br>TTCGCTGTGTTGGCTTCATTGCCGGGTTCTCGAAGCTGATTCTATTGGATCTGCAC<br>GGACATAATGAGGTGCCCTGGCTGGGGAGTCCCATTTGCTGGTTCTATATGGCCGC<br>ATCCGTGGTGTTGGACATCATCTACCCTGTTTGTTTGTACTTCATGCGACGCGAGGA<br>ACAGTCCGGGGAGCAAAGGAAAAACAAAGTCGACTAA	MSASDVFIGPWDLHTIDISLRWVAFICWSLNYISL<br>LGTAIRDRTPSMALLALCSDTGWEIVYGFIFPEAS<br>RHFGSGVRVWLLLHVPVVYVMLKFGADEWDHN<br>PLVKKNLPLVYVALTFGFGAAQMALANEIGPDLG<br>FFFGGVFCQTLLIFSHLCQLLSRGSTRGASYSIW<br>FFRCVGFIAGFSKLILLDLHGHNEVPWLGSPICW<br>FYMAASVVLDIIYPVCLYFMRREEQSGEQRKNK<br>VD*

Table S3. Primers used in this study.

Primer	Sequence (5′ to 3′)
pUSA_pyrG-F	TGTCGGCAACAGATCTGACTCTAGAGGATCCTTTCCTATAATAGAC
pUSA_pyrG-R	TCTTGCCTTGAATCCGACCTGCAGGCATGCAAGCTTG
Aspnov_pyrG-F	GCGACACGCTTCATCGAATGCTGAATCCATGATGCAGGCCAAG
Aspnov_pyrG-R	GGATTCAAGGCAAGAAGAAGGTAGGGAGAC
Aspnov_pyrG_3'-F	GATCTGTTGCCGACAATACCTCCCGAC
Aspnov_pyrG_3'-R	GATGAAGCGTGTCGCAGGTTG
HS801-F1	CAGCAAACTCCTTCTCCGCAGG
HS801-R1	GGAAAGGAAATTTCCACGACATTATCGGTC
HS801-F2	CGACAACCACAGTTCTGATCTCTTGC
HS801-R2	CCACCTAAGGTGCATATAGGACTATACACC
pPyrG_HS801-F1	GTCGTGGAAATTTCCTTTCCGACAGGTTTCCCGACTGGAAAGCG
pPyrG_HS801-R1	GATCAGAACTGTGGTTGTCGGGTAACGCCAGGGTTTTCCCAG
pPyrG_HS801-F2	CCTATATGCACCTTAGGTGGCAACTTAATCGCCTTGCAGCACATC
pPyrG_HS801-R2	TGCGGAGAAGGAGTTTGCTGCGTGCCAGCTGCATTAATGAATCGG
insA1-F	TCGAGCTCGGTACCCATTTCAATATCTGCCCCGGC
insA1-R	CTACTACAGATCCCCATTTTCTACTGCCGCCGAAC
insA2-F	TCGAGCTCGGTACCCTCTAGTCATGGGATCGCAGA
insA2-linker-R	TTCTTCTGGTGTTGAAGCCC
insA2-linker-F	GGGCTTCAACACCAGAAGAA
insA2-R	CTACTACAGATCCCCTGGGATTAGACGGCAACATG
insA4-F	TCGAGCTCGGTACCCATGAGCGATACACCTACCAG
insA4-R	CTACTACAGATCCCCGATCCGAGCTTGTTCTGATC
insA5-F	TCGAGCTCGGTACCCATGGCCGTCATTCCAGAATC
insA5-R	CTACTACAGATCCCCCTAGACACGCACAGCATACT
insA6-F	TCGAGCTCGGTACCCATGGGGTTTAACCCGACACT
insA6-R	CTACTACAGATCCCCGCTTTGCTGCGAAGAAAGC
insA7-F	TCGAGCTCGGTACCCATCCGAAGCCATGAGCTCAA
insA7-R	CTACTACAGATCCCCACTACATCCATTTGCCCCTG
insA8-F	TCGAGCTCGGTACCCATGACTATCTCCAAACCAGTCC
insA8-R	CTACTACAGATCCCCAGCATATGCATGGAAGCTCG
insA9-F	TCGAGCTCGGTACCCATGAATGCACTATTACTACCGTGGT
insA9-R	CTACTACAGATCCCCCACGAGCGTCTGAAACAACT
insB1-F	TCGAGCTCGGTACCCATGGACCAGCTTCGCTGGGTAC
insB1-R	CTACTACAGATCCCCAAGCACTCCAGTCAGTTGTC
insB2-F	TCGAGCTCGGTACCCGGCCAGTAATGAGTGCATCA
insB2-R	CTACTACAGATCCCCATCGCTCCGTTAGTCGACTT
insB3-F	TCGAGCTCGGTACCCATGGCCCTTCTCGATGACTAC
insB3-R	CTACTACAGATCCCCTTGCTTCCATGTCTCAAATTG
insB4-F	TCGAGCTCGGTACCCATGACCATTTCCGCCGCCAC
insB4-R	CTACTACAGATCCCCGTCACTGATTGCAGCTTG
insB5-F	TCGAGCTCGGTACCCATGGAGCAACGCGAAGCCTTG
insB5-R	CTACTACAGATCCCCAGACGGTCTTACTGAGCATC
insB7-F	TCGAGCTCGGTACCCATGACTATTACAAATCACAAGTCAAGAATCAG
insB7-R	CTACTACAGATCCCCTGTATCTAACGAAGGTCGACTCC
InF-pTAex3_BlnI-F	ACGCGCAATTCCTAGCCCATCATGGTGTTTTGATC
InF-pTAex3_BlnI-R	TTGACCGAATCCTAGGTAAGATACATGAGCTTCGG
InF-pAdeA_XbaI-F	GCAGGTCGACTCTAGCCCATCATGGTGTTTTGATC
InF-pAdeA_XbaI-R	TAGTAGATCCTCTAGGTAAGATACATGAGCTTCGG
InF-Linker-F1	GCTCGCGAGCGCGTTCCACTGCATCATCAGTCTAG
InF-Linker-R1	AACGCGCTCGCGAGCAAGTACCATACAGTACCGCG
InF-pAdeA_SpeI-F	TAGAGGATCTACTAGTCAAGAGCAGAATGTGAACG
InF-pAdeA_SpeI-R	AATCCATATGACTAGTGATACATGAGCTTCGGTG
InF-pPTRI_SmaI-F	CTCTAGAGGATCCCCATCATGGTGTTTTGATC
InF-pPTRI_SmaI-R	TCGAGCTCGGTACCCGTAAGATACATGAGCTTCGG
InF-pPTRI_HindIII-F	TGATTACGCCAAGCTCCCATCATGGTGTTTTGATC
InF-pPTRI_HindIII-R	GCAGGCATGCAAGCTGTAAGATACATGAGCTTCGG
InF-pUSA_BamHI-F	TTATAGGAAAGGATCCCCATCATGGTGTTTTGATC
InF-pUSA_BamHI-R	TGACTCTAGAGGATCGTAAGATACATGAGCTTCGG
NdeI_insB4-F	CGCGCGGCAGCCATATGACCATTTCCGCCGCCAC
EcoRI_insB4-R	GACGGAGCTCGAATTCCGTCACTGATTGCAGCTTG
NdeI_insB5-F	CGCGCGGCAGCCATATGGAGCAACGCGAAGCCTTG
EcoRI_insB5-R	GACGGAGCTCGAATTAGACGGTCTTACTGAGCATC

Table S4. Plasmids constructed in this study and PCR conditions for the amplification of the inserts for the plasmid constructions.

Plasmid	Inserts	Primer 1	Primer 2	PCR Template	Vector
pPyrG	part of pUSA<br>*Aspnov_pyrG*<br>3′-flanking region of *Aspnov_pyrG*	pUSA_pyrG-F<br>Aspnov_pyrG-F<br>Aspnov_pyrG_3'-F	pUSA_pyrG-R<br>Aspnov_pyrG-R<br>Aspnov_pyrG_3'-R	pUSA<br>gDNA<br>gDNA	N/A
pPyrG-HR	HS801 upstream region<br>part of pPyrG (1)<br>HS801 downstream region<br>part of pPyrG (2)	HS801-F1<br>pPyrG_HS801-F1<br>HS801-F2<br>pPyrG_HS801-F2	HS801-R1<br>pPyrG_HS801-R1<br>HS801-R2<br>pPyrG_HS801-R2	gDNA<br>pPyrG<br>gDNA<br>pPyrG	N/A
pTAex3-insA1	*insA1*	insA1-F	insA1-R	gDNA	pTAex3 digested with *Sma*I
pTAex3-insA2	1st half of *insA2*<br>2nd half of *insA2*	insA2-F<br>insA2-linker-F	insA2-linker-R<br>insA2-R	gDNA<br>gDNA	pTAex3 digested with *Sma*I
pTAex3-insA4	*insA4*	insA4-F	insA4-R	gDNA	pTAex3 digested with *Sma*I
pTAex3-insA5	*insA5*	insA5-F	insA5-R	gDNA	pTAex3 digested with *Sma*I
pTAex3-insA6	*insA6*	insA6-F	insA6-R	gDNA	pTAex3 digested with *Sma*I
pTAex3-insA7	*insA7*	insA7-F	insA7-R	gDNA	pTAex3 digested with *Sma*I
pTAex3-insA8	*insA8*	insA8-F	insA8-R	gDNA	pTAex3 digested with *Sma*I
pTAex3-insA9	*insA9*	insA9-F	insA9-R	gDNA	pTAex3 digested with *Sma*I
pPyrG-insB1	*insB1*	insB1-F	insB1-R	gDNA	pPyrG digested with *Sma*I
pPyrG-HR-insB1	*insB1*	insB1-F	insB1-R	gDNA	pPyrG-HR digested with *Sma*I
pTAex3-insB2	*insB2*	insB2-F	insB2-R	gDNA	pTAex3 digested with *Sma*I
pTAex3-insB3	*insB3*	insB3-F	insB3-R	gDNA	pTAex3 digested with *Sma*I
pTAex3-insB4	*insB4*	insB4-F	insB4-R	gDNA	pTAex3 digested with *Sma*I
pTAex3-insB5	*insB5*	insB5-F	insB5-R	gDNA	pTAex3 digested with *Sma*I
pPyrG-insB5	*insB5*	insB5-F	insB5-R	gDNA	pPyrG digested with *Sma*I
pTAex3-insB7	*insB7*	insB7-F	insB7-R	gDNA	pTAex3 digested with *Sma*I
pTAex3-insA2+insA5	*PamyB-insA5-TamyB*	InF-pTAex3_BlnI-F	InF-pTAex3_BlnI-R	pTAex3-insA5	pTAex3-insA2 digested with *Bln*I
pAdeA-insA1+insA4	*PamyB-insA1-TamyB*<br>*PamyB-insA4-TamyB*	InF-pAdeA_XbaI-F<br>InF-Linker-F1	InF-Linker-R1<br>InF-pAdeA_XbaI-R	pTAex3-insA1<br>pTAex3-insA4	pAdeA digested with *Xba*I
pAdeA-insA1+insA4+insA7	*PamyB-insA7-TamyB*	InF-pAdeA_SpeI-F	InF-pAdeA_SpeI-R	pTAex3-insA7	pAdeA-insA1+insA4 digested with *Spe*I
pAdeA-insA1+insA4+insB2	*PamyB-insB2-TamyB*	InF-pAdeA_SpeI-F	InF-pAdeA_SpeI-R	pTAex3-insB2	pAdeA-insA1+insA4 digested with *Spe*I
pPTRI-insA8+insA9	*PamyB-insA8-TamyB*<br>*PamyB-insA9-TamyB*	InF-pPTRI_SmaI-F<br>InF-Linker-F1	InF-Linker-R1<br>InF-pPTRI_*SmaI*-R	pTAex3-insA8<br>pTAex3-insA9	pPTRI digested with *Sma*I
pPTRI-insA8+insA6	*PamyB-insA8-TamyB*<br>*PamyB-insA6-TamyB*	InF-pPTRI_SmaI-F<br>InF-Linker-F1	InF-Linker-R1<br>InF-pPTRI_SmaI-R	pTAex3-insA8<br>pTAex3-insA6	pPTRI digested with *Sma*I
pPTRI-insA6+insA9	*PamyB-insA6-TamyB*<br>*PamyB-insA9-TamyB*	InF-pPTRI_SmaI-F<br>InF-Linker-F1	InF-Linker-R1<br>InF-pPTRI_SmaI-R	pTAex3-insA6<br>pTAex3-insA9	pPTRI digested with *Sma*I
pPTRI-insA8+insA6+insA9	*PamyB-insA9-TamyB*	InF-pPTRI_HindIII-F	InF-pPTRI_HindIII-R	pTAex3-insA9	pPTRI-insA8+insA6 digested with *Hind*III
pPTRI-insB3+insB7	*PamyB-insB3-TamyB*<br>*PamyB-insB7-TamyB*	InF-pPTRI_SmaI-F<br>InF-Linker-F1	InF-Linker-R1<br>InF-pPTRI_SmaI-R	pTAex3-insB3<br>pTAex3-insB7	pPTRI digested with *Sma*I
pPTRI-insB3+insB4	*PamyB-insB3-TamyB*<br>*PamyB-insB4-TamyB*	InF-pPTRI_SmaI-F<br>InF-Linker-F1	InF-Linker-R1<br>InF-pPTRI_*SmaI*-R	pTAex3-insB3<br>pTAex3-insB4	pPTRI digested with *Sma*I
pPTRI-insB4+insB7	*PamyB-insB4-TamyB*<br>*PamyB-insB7-TamyB*	InF-pPTRI_SmaI-F<br>InF-Linker-F1	InF-Linker-R1<br>InF-pPTRI_SmaI-R	pTAex3-insB4<br>pTAex3-insB7	pPTRI digested with *Sma*I
pPTRI-insB3+insB4+insB7	*PamyB-insB7-TamyB*	InF-pPTRI_HindIII-F	InF-pPTRI_HindIII-R	pTAex3-insB7	pPTRI-insB3+insB4 digested with *Hind*III
pPyrG-insB1+insB5	*PamyB-insB5-TamyB*	InF-pUSA_BamHI-F	InF-pUSA_BamHI-R	pTAex3-insB5	pPyrG-insB1 digested with *Bam*HI
pET28a(+)-insB4	*insB4*	NdeI_insB4-F	EcoRI-insB4-F	cDNA	pET28a digested with *Nde*I and *Eco*RI
pET28a(+)-insB5	*insB5*	NdeI-insB5-F	EcoRI-insB5-F	cDNA	pET28a digested with *Nde*I and *Eco*RI

Table S5. *Aspergillus oryzae* transformants constructed in this study.

Strain	Host strain	Plasmids used for transformation
A. oryzae/*insA1+A2+A4+A5+A7*	*A. oryzae* NSAR1	pTAex3-insA2+insA5, pAdeA-insA1+insA4+insA7
A. oryzae/*insA1+A2+A4+A5+B2*	*A. oryzae* NSAR1	pTAex3-insA2+insA5, pAdeA-insA1+insA4+insB2
A. oryzae/*insA1+A2+A4+A5+A7+A6+A8*	*A. oryzae*/*insA1+A2+A4+A5+A7*	pPTRI-insA8+insA6
A. oryzae/*insA1+A2+A4+A5+A7+A6+A9*	*A. oryzae*/*insA1+A2+A4+A5+A7*	pPTRI-insA6+insA9
A. oryzae/*insA1+A2+A4+A5+A7+A8+A9*	*A. oryzae*/*insA1+A2+A4+A5+A7*	pPTRI-insA8+insA9
A. oryzae/*insA1+A2+A4+A5+A7+A6+A8+A9*	*A. oryzae*/*insA1+A2+A4+A5+A7*	pPTRI-insA8+insA6+insA9
A. oryzae/*insA1+A2+A4+A5+B2* (*pyrG*$^{-}$)	*A. oryzae*/*insA1+A2+A4+A5+B2*	N/A
A. oryzae/*insA1+A2+A4+A5+B2+B1+B3+B4+B5*	*A. oryzae*/*insA1+A2+A4+A5+B2* (*pyrG*$^{-}$)	pPTRI-insB3+insB4, pPyrG-insB1+insB5
A. oryzae/*insA1+A2+A4+A5+B2+B1+B3+B4+B7*	*A. oryzae*/*insA1+A2+A4+A5+B2* (*pyrG*$^{-}$)	pPTRI-insB3+insB4+insB7, pPyrG-insB1
A. oryzae/*insA1+A2+A4+A5+B2+B1+B3+B5+B7*	*A. oryzae*/*insA1+A2+A4+A5+B2* (*pyrG*$^{-}$)	pPTRI-insB3+insB7, pPyrG-insB1+insB5
A. oryzae/*insA1+A2+A4+A5+B2+B1+B4+B5+B7*	*A. oryzae*/*insA1+A2+A4+A5+B2* (*pyrG*$^{-}$)	pPTRI-insB4+insB7, pPyrG-insB1+insB5
A. oryzae/*insA1+A2+A4+A5+B2+B3+B4+B5+B7*	*A. oryzae*/*insA1+A2+A4+A5+B2* (*pyrG*$^{-}$)	pPTRI-insB3+insB4+insB7, pPyrG-insB5
A. oryzae/*insA1+A2+A4+A5+B2+B1+B3+B4+B5+B7*	*A. oryzae*/*insA1+A2+A4+A5+B2* (*pyrG*$^{-}$)	pPTRI-insB3+insB4+insB7, pPyrG-insB1+insB5
A. oryzae/*insB1*	*A. oryzae* NSARU1	pPyrG-HR-insB1

Table S6. NMR data for insuetusin A1 (**2**) obtained from the isotope incorporation experiments (see Figure S127 for the ^{13}C NMR spectrum).

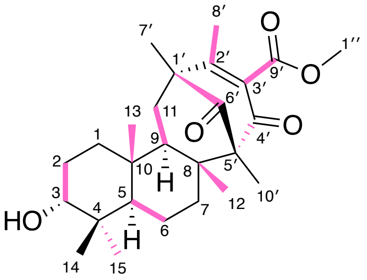

position	δ_C	$^1J_{CC}$ from [1, 2-$^{13}C_2$]-acetate (Hz) / Coupled carbon
1	33.1	-
2	25.0	37.3 / C-3
3	75.6	37.3 / C-2
4	37.5	36.7 / C-15
5	48.3	35.0 / C-6
6	18.0	35.0 / C-5
7	33.2	-
8	46.7	36.3 / C-12
9	50.0	36.0 / C-11
10	37.3	35.6 / C-13
11	34.3	36.0 / C-9
12	15.7	36.3 / C-8
13	17.2	35.6 / C-10
14	22.0	-
15	28.0	36.7 / C-4
1′	51.0	37.4 / C-6′
2′	159.2	41.8 / C-8′
3′	137.3	75.9 / C-9′
4′	196.7	39.0 / C-5′
5′	69.5	39.0 / C-4′
6′	208.5	37.4 / C-1′
7′	19.2	-
8′	16.6	41.8 / C-2′
9′	166.9	75.9 / C-3′
10′	9.7	-
1′′	52.5	-

^{1}H NMR: 600 MHz, ^{13}C NMR: 150 MHz (in $CDCl_3$)

Table S7. NMR data for insuetusin B1 (**3**) obtained from the isotope incorporation experiments (see Figure S128 for the ^{13}C NMR spectrum).

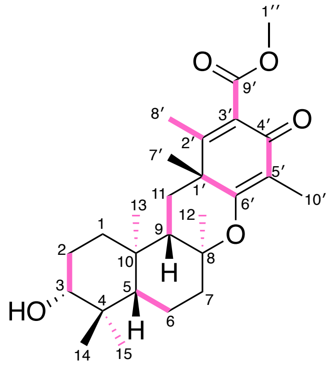

position	δ_C	$^1J_{CC}$ from [1, 2-$^{13}C_2$]-acetate (Hz) / Coupled carbon
1	37.3	-
2	27.0	36.7 / C-3
3	78.5	36.7 / C-2
4	39.0	35.6 / C-15
5	55.3	35.0 / C-6
6	19.6	35.0 / C-5
7	40.7	-
8	84.2	38.9 / C-12
9	51.4	34.5 / C-11
10	36.7	35.6 / C-13
11	28.7	34.5 / C-9
12	21.2	38.9 / C-8
13	15.8	35.6/ C-10
14	28.0	-
15	15.2	35.6 / C-4
1′	41.6	45.0 / C-6′
2′	157.3	42.5 / C-8′
3′	132.0	75.9 / C-9′
4′	183.7	56.0 / C-5′
5′	120.3	56.0 / C-4′
6′	171.6	45.0 / C-1′
7′	27.4	-
8′	15.3	42.5 / C-2′
9′	168.0	75.9 / C-3′
10′	7.9	-
1′′	52.2	-

^{1}H NMR: 600 MHz, ^{13}C NMR: 150 MHz (in $CDCl_3$)

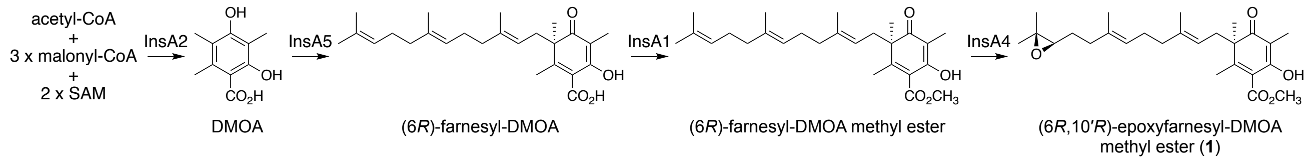

Figure S1. Proposed early-stage biosynthesis of insuetusins.

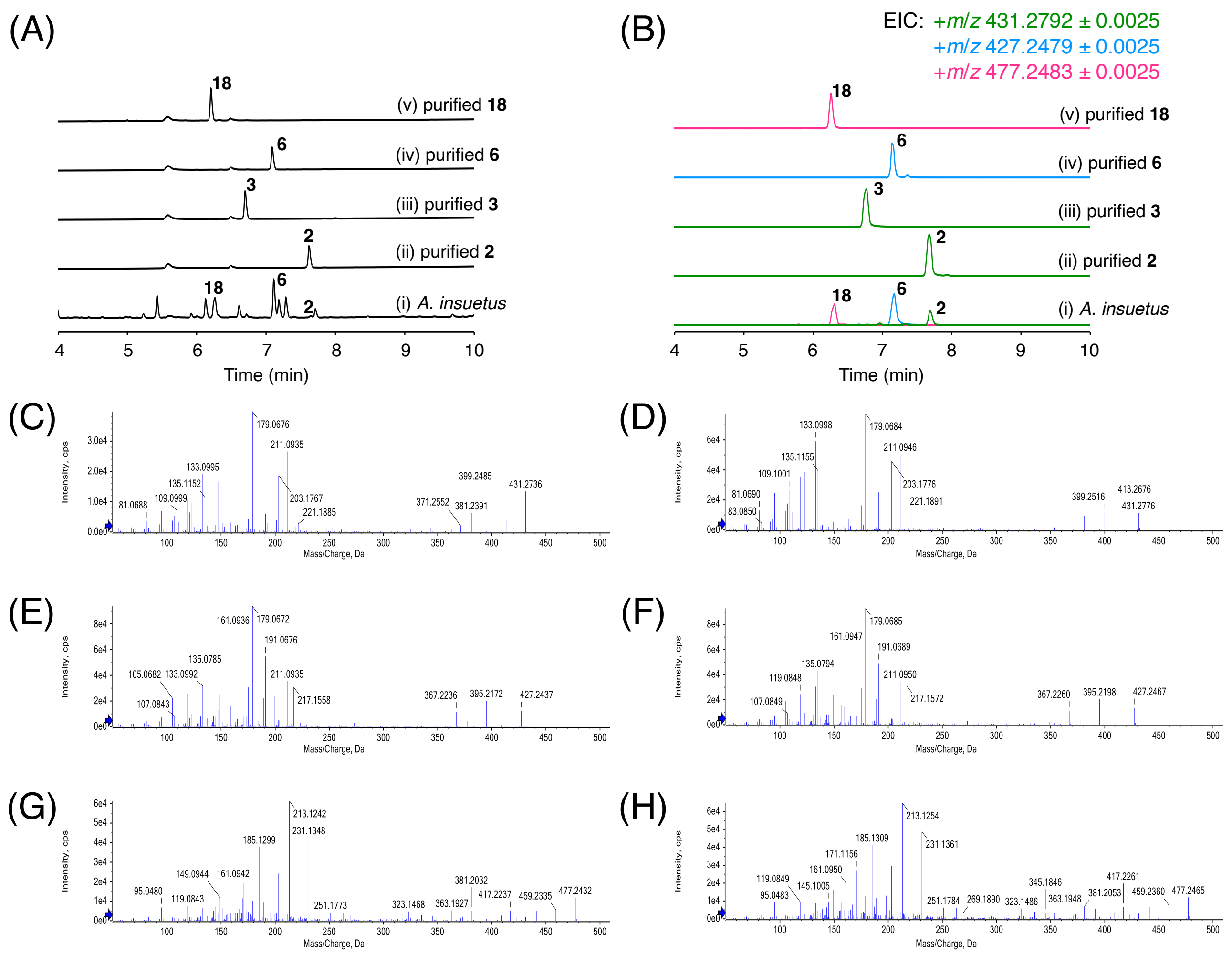

Figure S2. (A) HPLC analysis of the metabolites from *A. insuetus* CBS 107.25 and purified compounds **2**, **3**, **6**, and **18**. Chromatograms were monitored at 254 nm. (B) Extracted ion chromatograms of the metabolites from *A. insuetus* CBS 107.25 and purified compounds **2**, **3**, **6**, and **18**. MS/MS spectra of (C) **2** detected in *A. insuetus*, (D) purified **2**, (E) **6** detected in *A. insuetus*, (F) purified **6**, (G) **18** detected in *A. insuetus*, and (H) purified **18**.

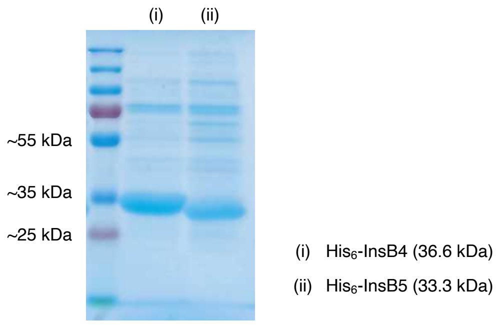

Figure S3. SDS-PAGE analysis of the purified proteins.

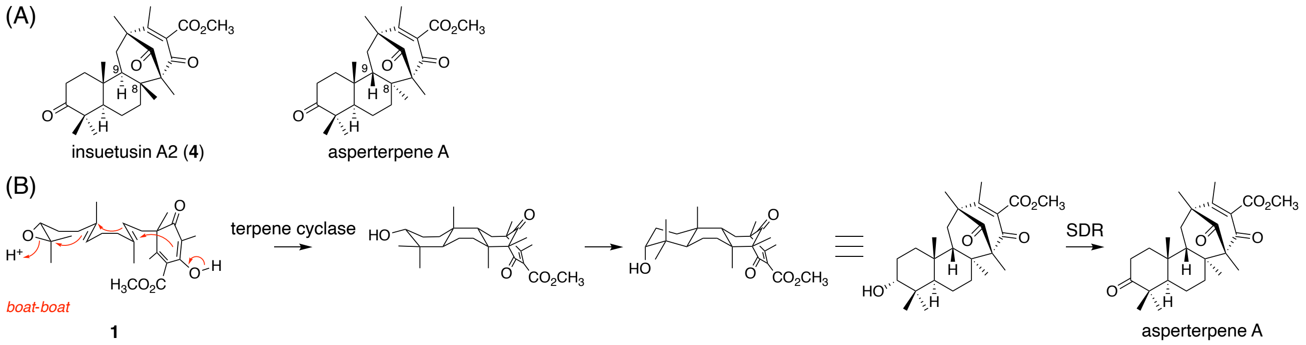

Figure S4. (A) Structures of insuetusin A2 (**4**) and asperterpene A. (B) Proposed biosynthetic mechanism of asperterpene A. SDR: short-chain dehydrogenase/reductase.

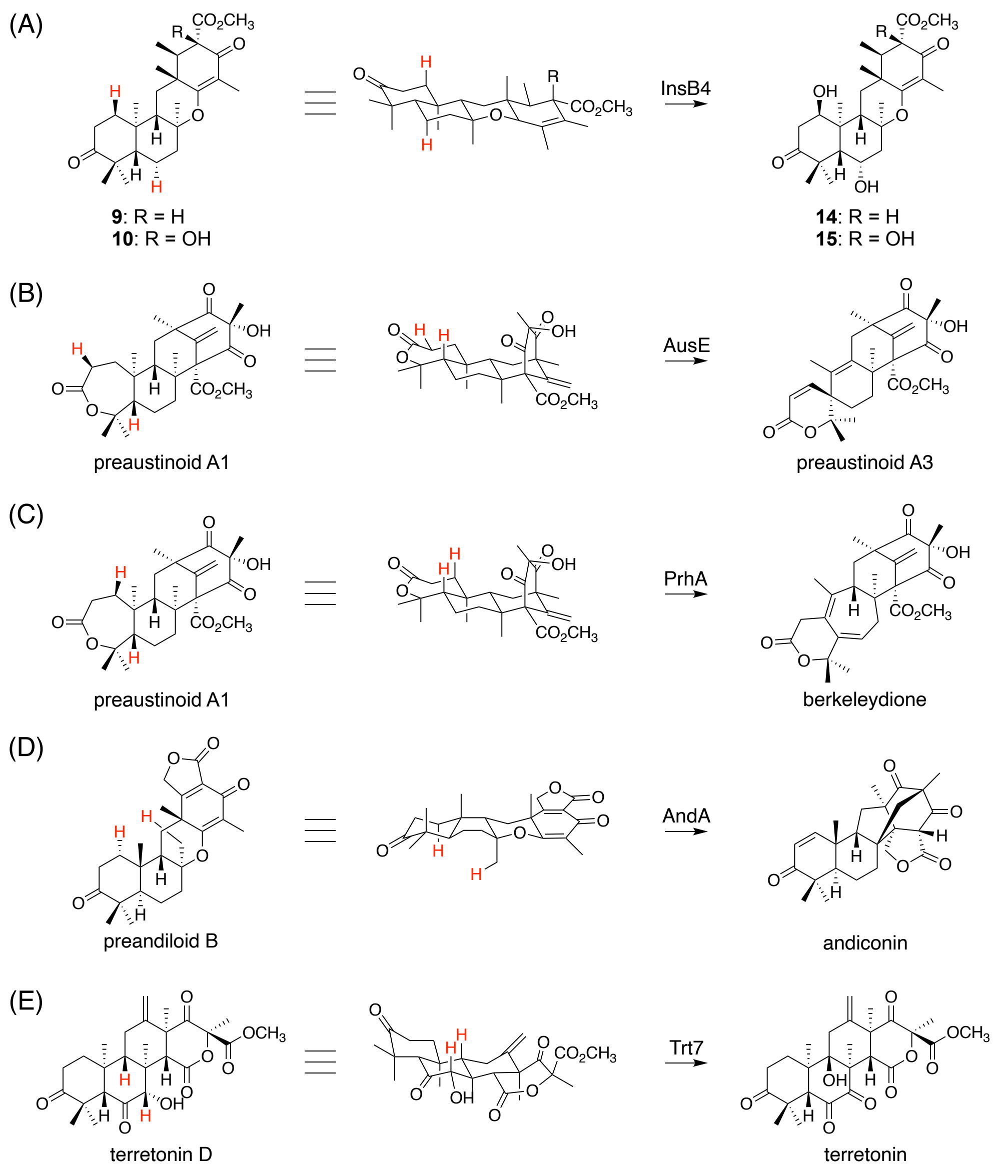

Figure S5. Multifunctional αKG-dependent enzymes involved in fungal meroterpenoid pathways. Reactions catalyzed by (A) InsB4, (B) AusE, (C) PrhA, (D) AndA, and (E) Trt7. Hydrogen atoms abstracted by the ferryl-oxo species of the enzymes are shown in *red*.

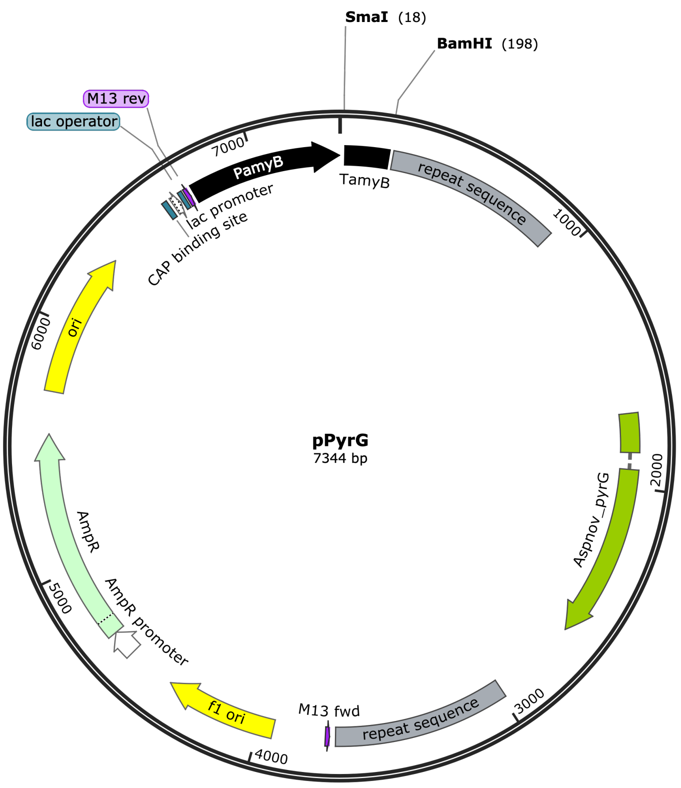

Figure S6. Vector map of pPyrG.

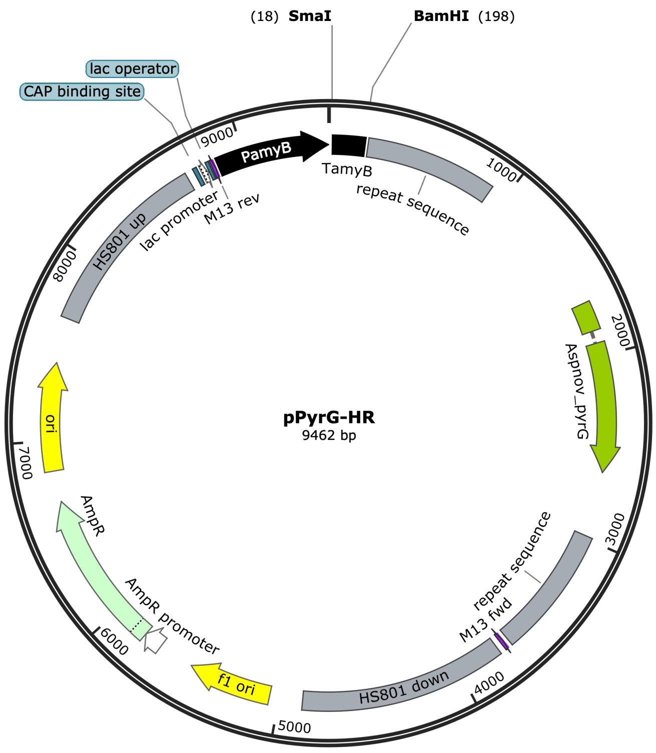

Figure S7. Vector map of pPyrG-HR.

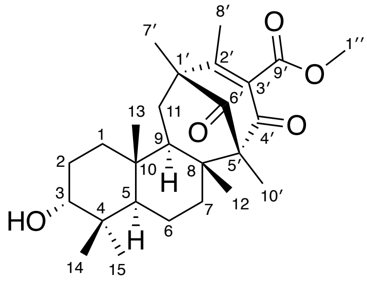

2

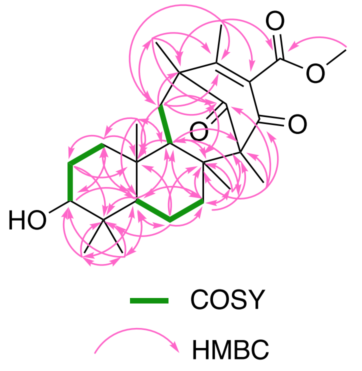

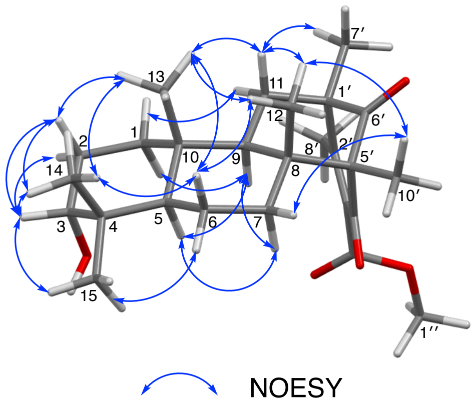

position	δC, type	δH, mult. (*J* in Hz)
1	33.1, C	1.28 (α), td (12.3, 1.4)
		1.34 (β), m
2	25.0, CH_2	1.56, (α), dq (14.8, 3.3)
		1.90 (β), dddd (14.9, 12.1, 4.5, 2.6)
3	75.6, CH	3.37, t (2.8)
4	37.5, C	
5	48.3, CH	1.27, dd (13.2, 4.7)
6	18.0, CH_2	1.49 (α), m
		1.34 (β), m
7	33.2, CH_2	1.64 (α), td (13.0, 3.7)
		1.48 (β), m
8	46.7, C	
9	50.0, CH	1.51, dd (12.7, 3.7)
10	37.3, C	
11	34.3, CH_2	1.80 (α), dd (13.7, 3.7)
		1.66 (β), t (13.4)
12	15.7, CH_3	0.82, s
13	17.2, CH_3	0.90, s
14	22.0, CH_3	0.81, s
15	28.0, CH_3	0.92, s
1′	51.0, C	
2′	159.2, C	
3′	137.3, C	
4′	196.7, C	
5′	69.5, C	
6′	208.5, C	
7′	19.2, CH_3	1.31, s
8′	16.6, CH_3	1.94, s
9′	166.9, C	
10′	9.7, CH_3	1.10, s
1′′	52.5, CH_3	3.84, s

^{1}H NMR: 600 MHz, ^{13}C NMR: 150 MHz (in $CDCl_3$)

Figure S8. NMR data of insuetusin A1 (**2**).

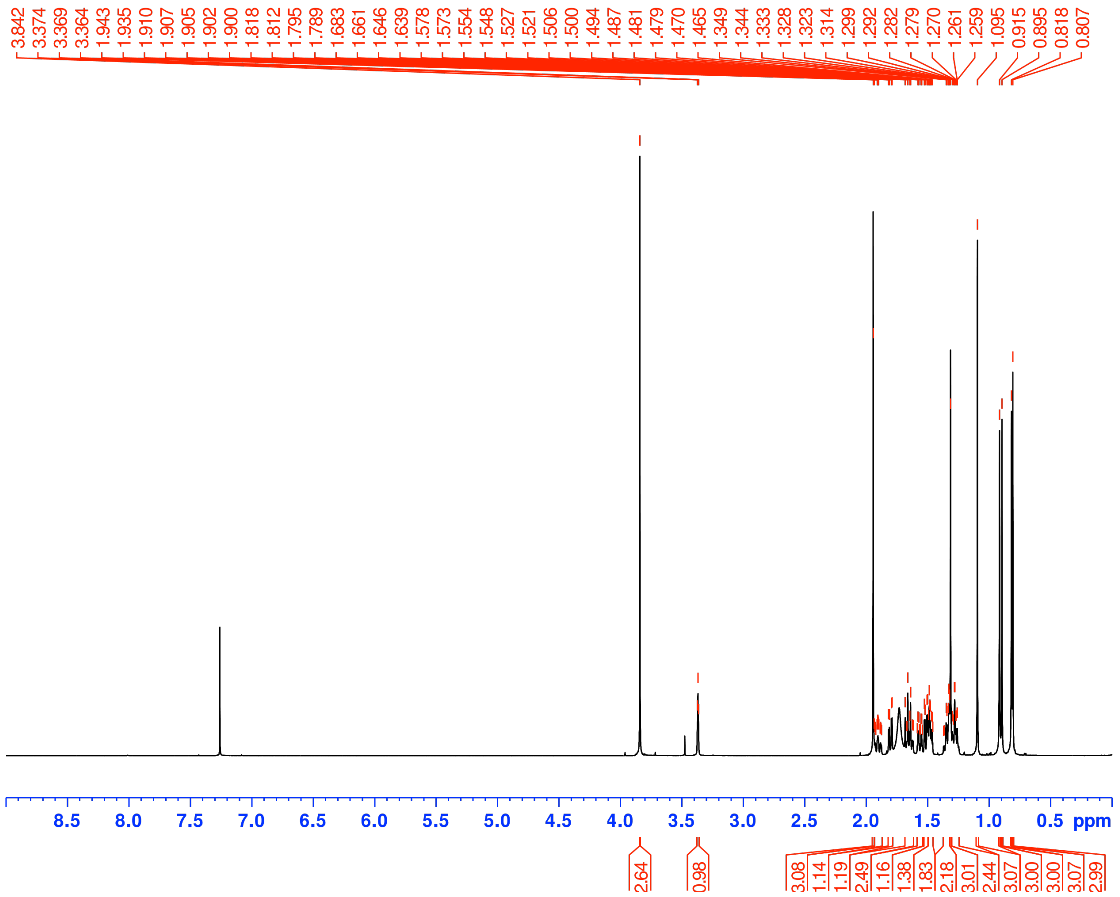

Figure S9. ^{1}H NMR spectrum of insuetusin A1 (**2**) in $CDCl_3$ at 600 MHz.

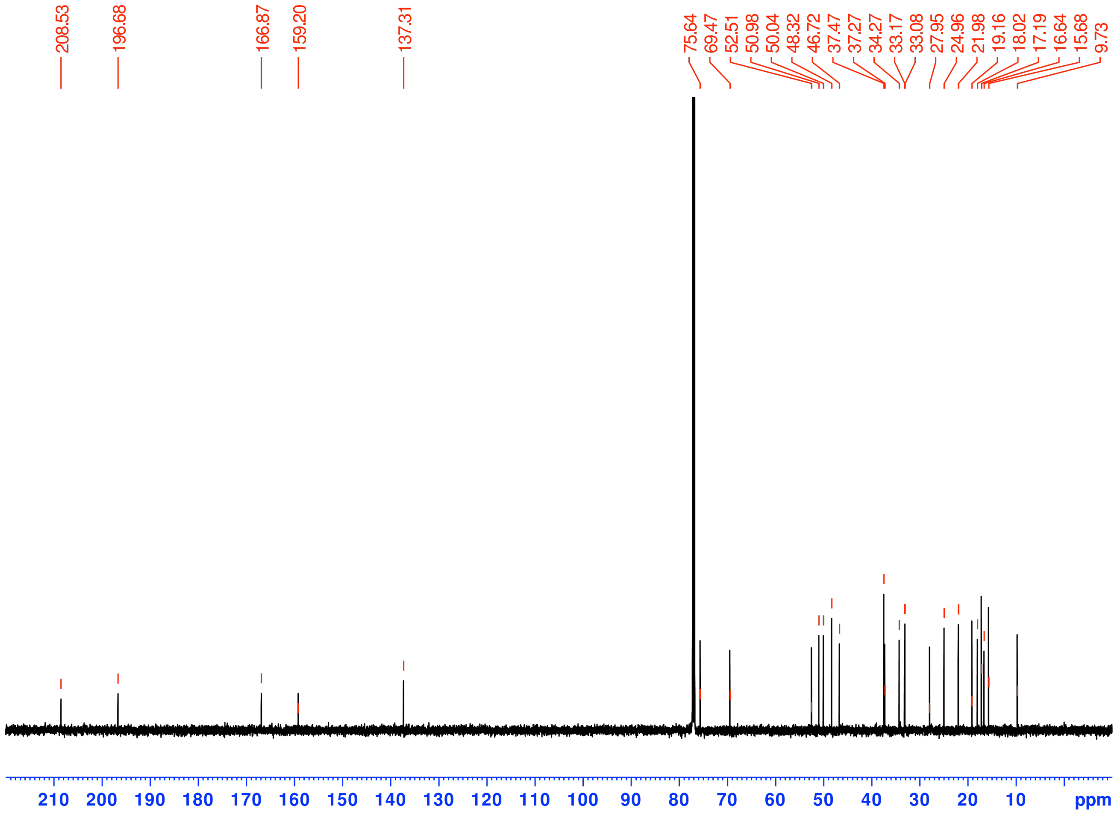

Figure S10. ^{13}C NMR spectrum of insuetusin A1 (**2**) in $CDCl_3$ at 150 MHz.

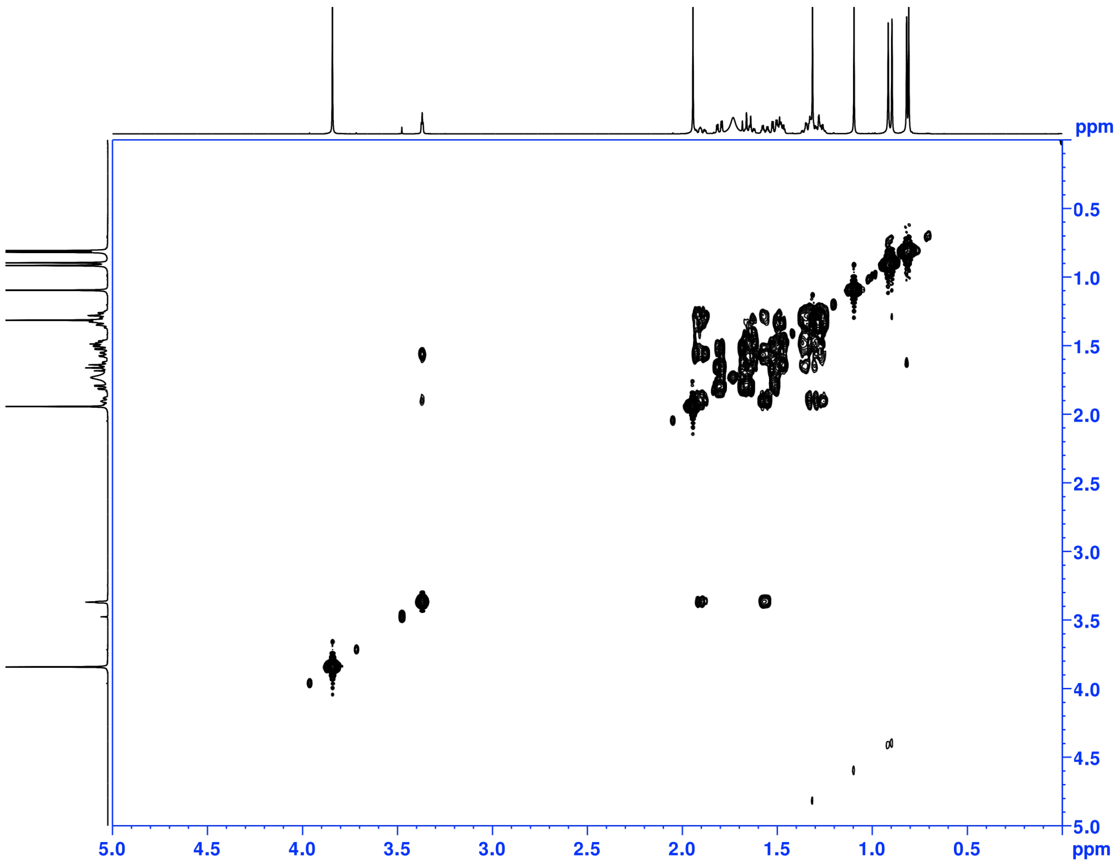

Figure S11. ^{1}H-^{1}H COSY spectrum of insuetusin A1 (**2**) in $CDCl_3$.

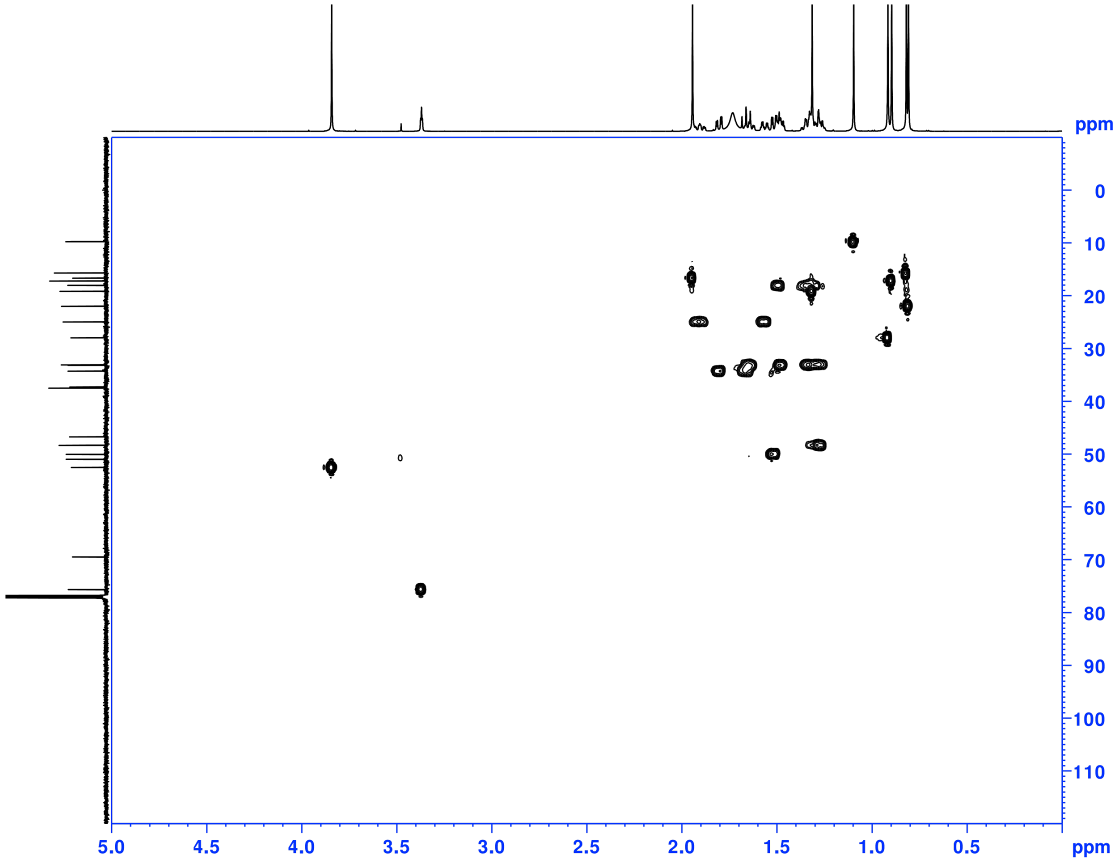

Figure S12. HSQC spectrum of insuetusin A1 (**2**) in $CDCl_3$.

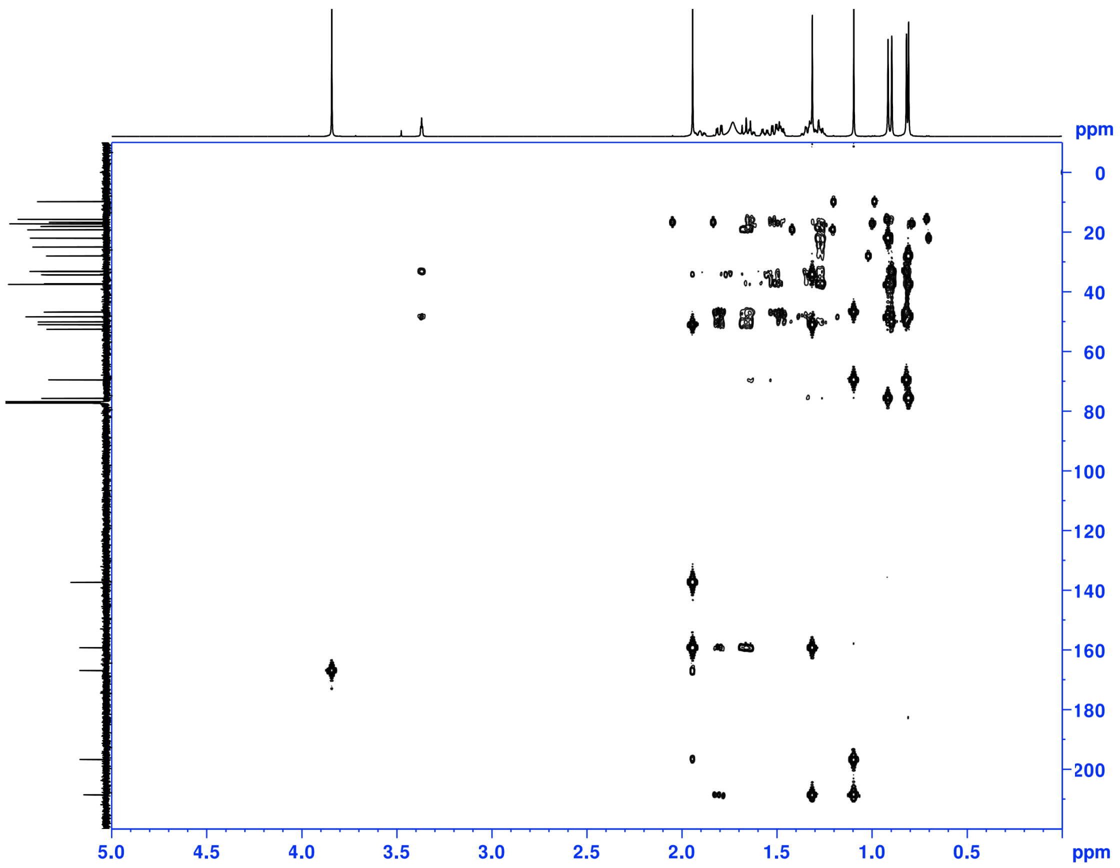

Figure S13. HMBC spectrum of insuetusin A1 (**2**) in $CDCl_3$.

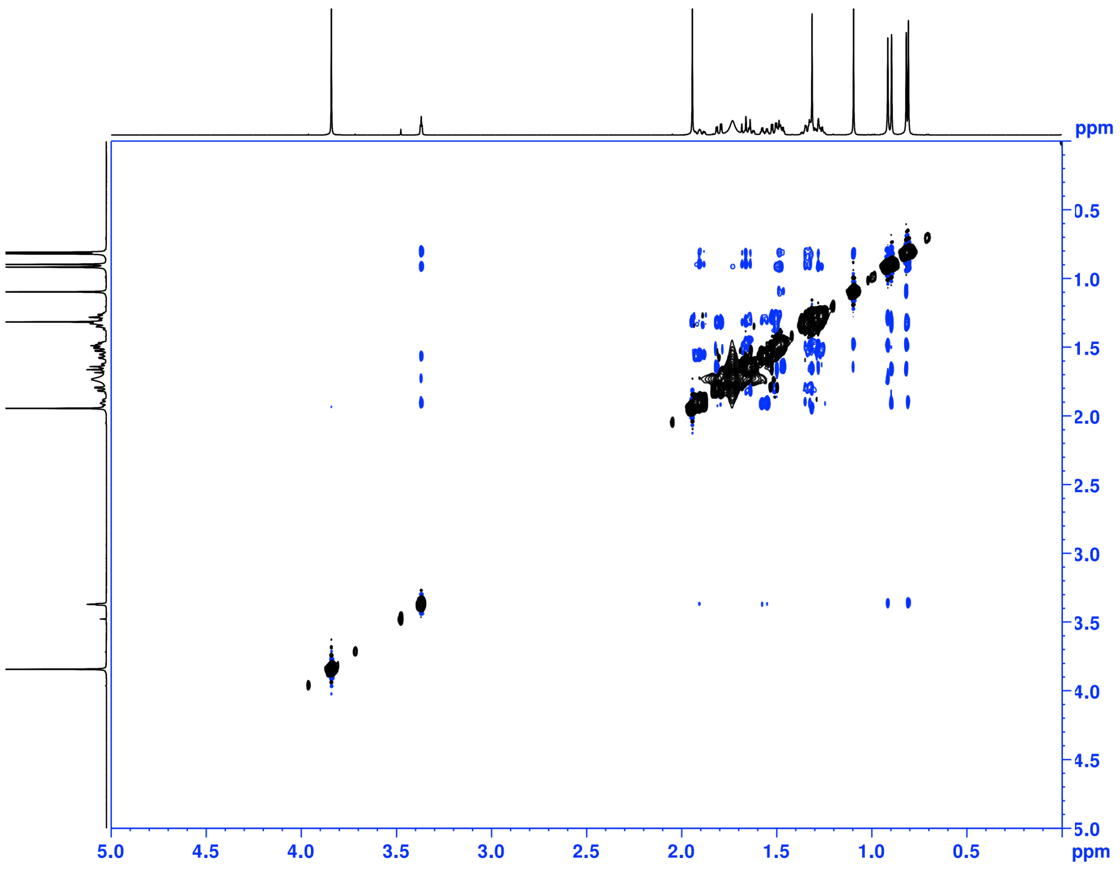

Figure S14. NOESY spectrum of insuetusin A1 (**2**) in $CDCl_3$.

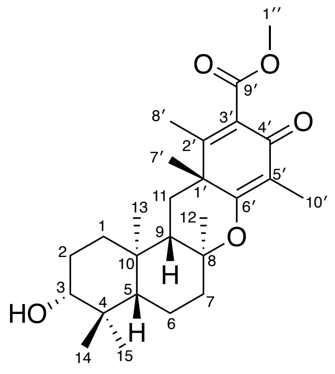

3

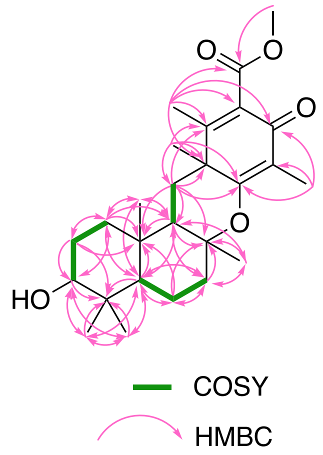

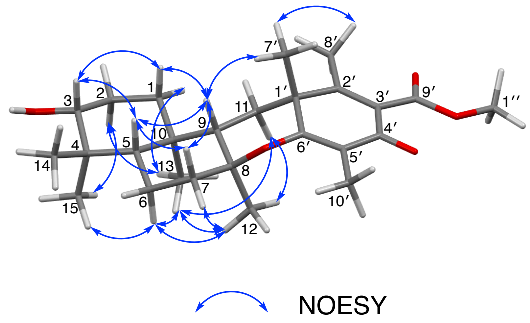

position	δC, type	δH, mult. (*J* in Hz)
1	37.3, CH_2	1.67 (α), dt (13.0, 3.3)
		1.09 (β), td (13.0, 3.5)
2	27.0, CH_2	1.63 (α), qd (13.0, 3.5)
		1.73 (β), m
3	78.5, CH	3.28, dd (11.6, 4.4)
4	39.0, C	
5	55.3, CH	1.04, dd (12.0, 1.8)
6	19.6, CH_2	1.39 (α), qd (13.6, 3.2)
		1.83 (β), dq (14.0, 3.0)
7	40.7, CH_2	2.03 (α), dt (12.2, 3.0)
		1.75 (β), m
8	84.2, C	
9	51.4, CH	1.87, dd (13.1, 2.8)
10	36.7, C	
11	28.7, CH_2	1.51 (α), t (13.0)
		1.76 (β), dd (13.0, 2.8)
12	21.2, CH_3	1.13, s
13	15.8, CH_3	0.75, s
14	28.0, CH_3	1.03, s
15	15.2, CH_3	0.78, s
1′	41.6, CH_3	
2′	157.3, C	
3′	132.0, C	
4′	183.7, C	
5′	120.3, C	
6′	171.6, C	
7′	27.4, CH_3	1.40, s
8′	15.3, CH_3	1.94, s
9′	168.0, C	
10′	7.9, CH_3	1.77, s
1′′	52.2, CH_3	3.86, s

^{1}H NMR: 600 MHz, ^{13}C NMR: 150 MHz (in $CDCl_3$)

Figure S15. NMR data of insuetusin B1 (**3**).

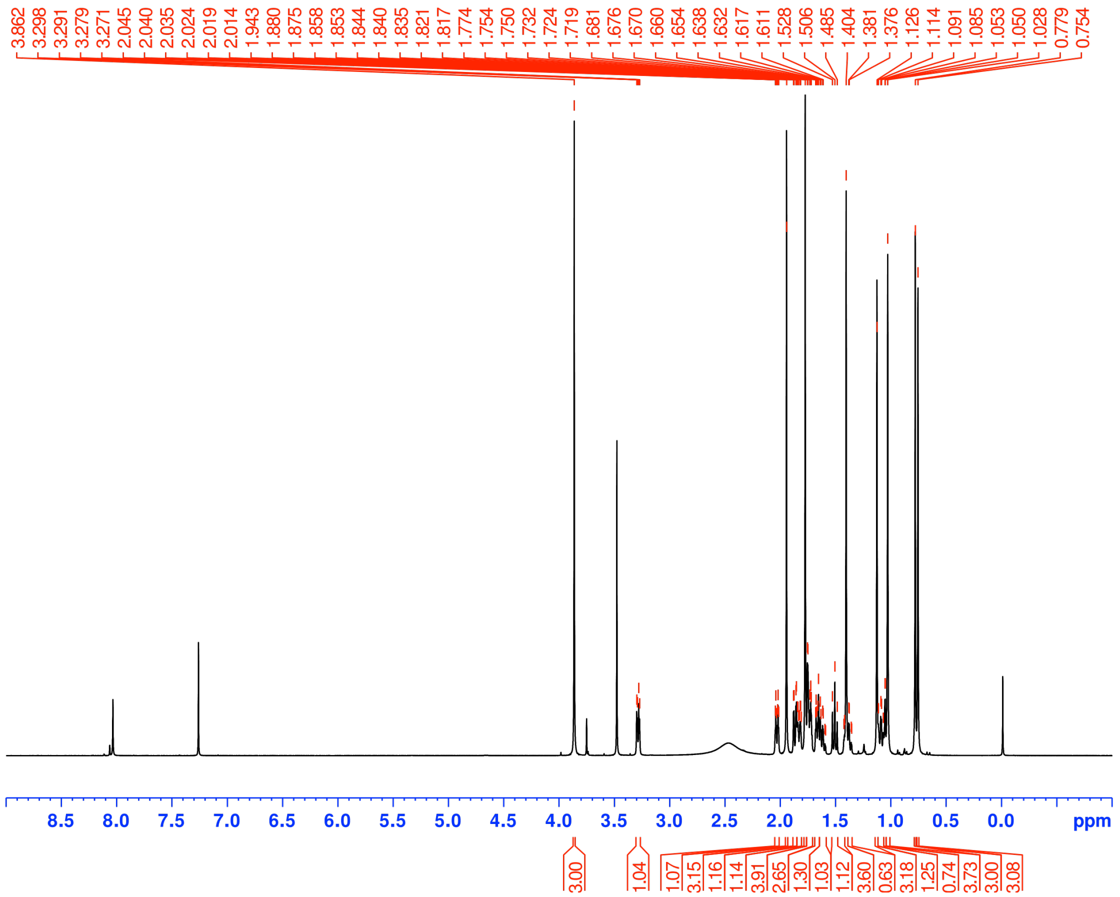

Figure S16. ^{1}H NMR spectrum of insuetusin B1 (**3**) in $CDCl_3$ at 600 MHz.

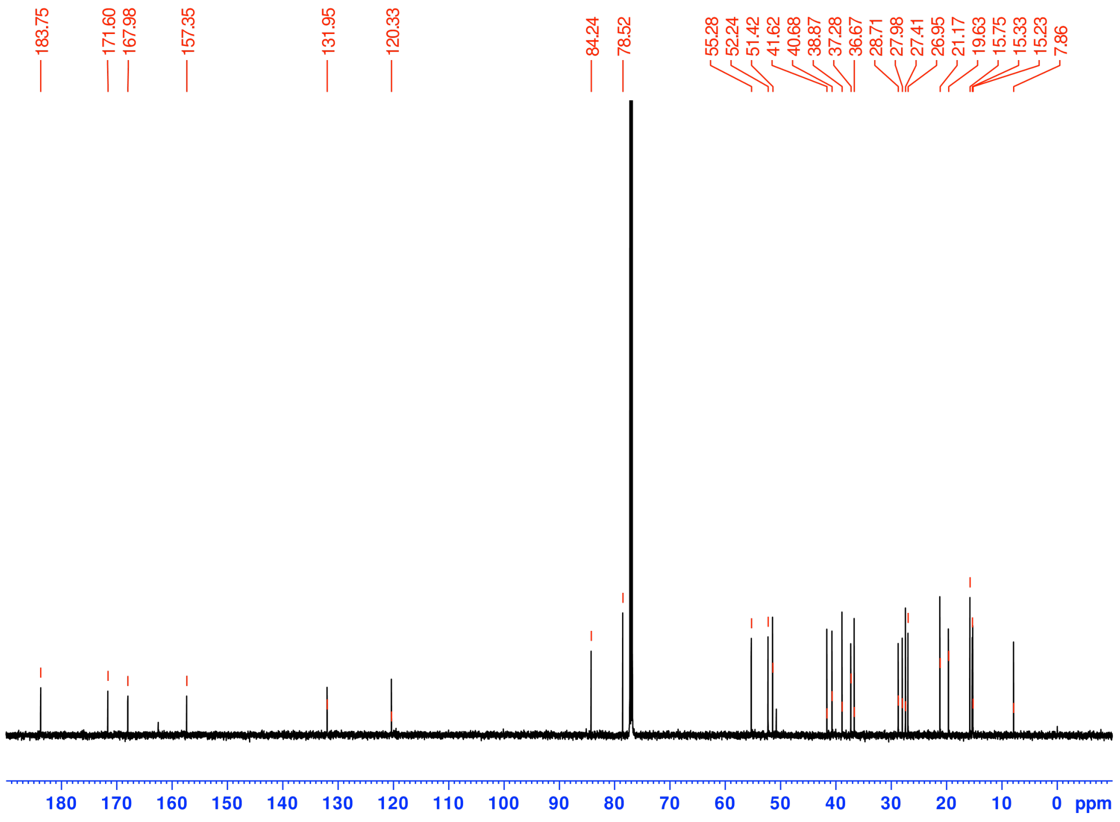

Figure S17. ^{13}C NMR spectrum of insuetusin B1 (**3**) in $CDCl_3$ at 150 MHz.

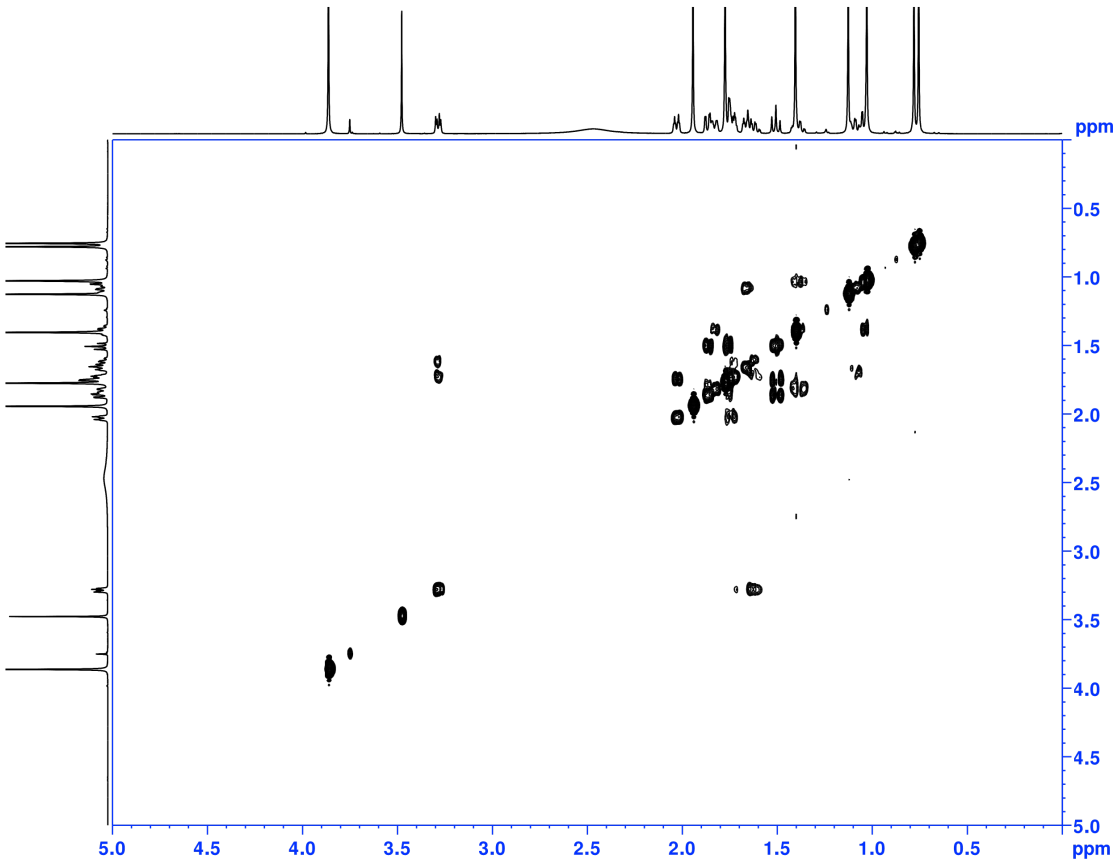

Figure S18. ^{1}H-^{1}H COSY spectrum of insuetusin B1 (**3**) in $CDCl_3$.

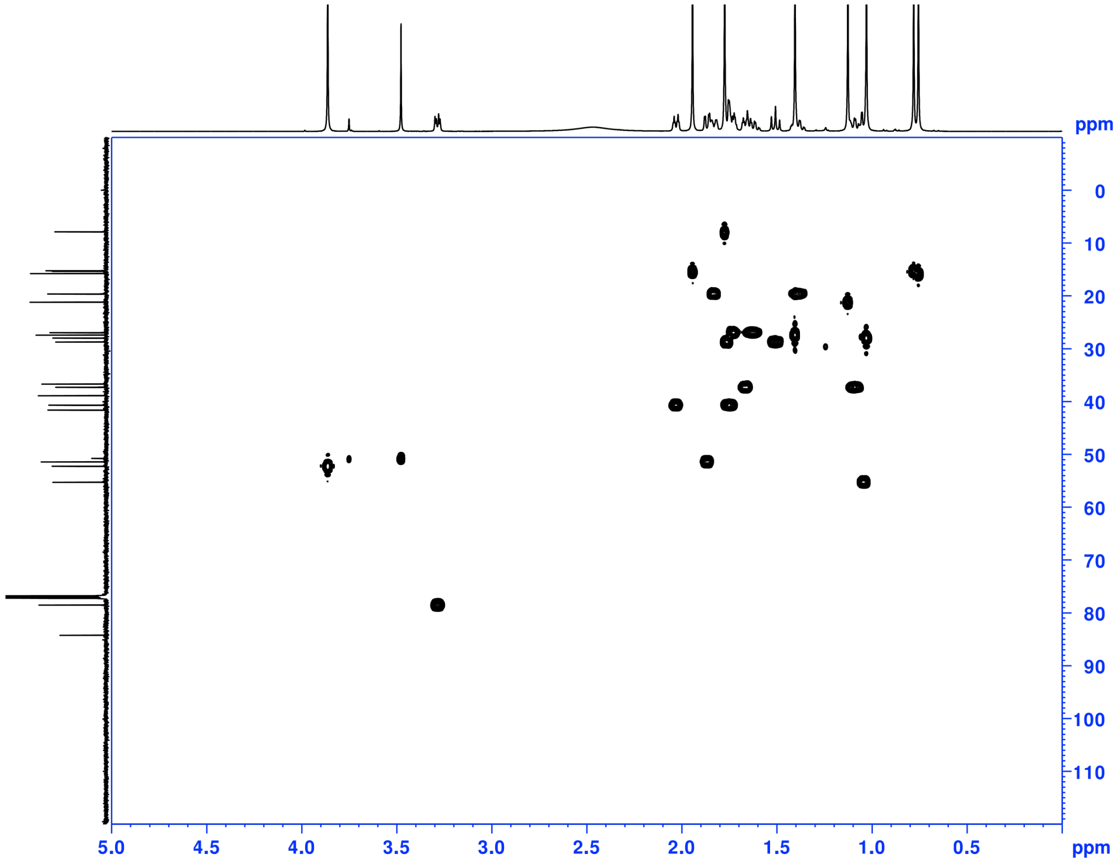

Figure S19. HSQC spectrum of insuetusin B1 (**3**) in $CDCl_3$.

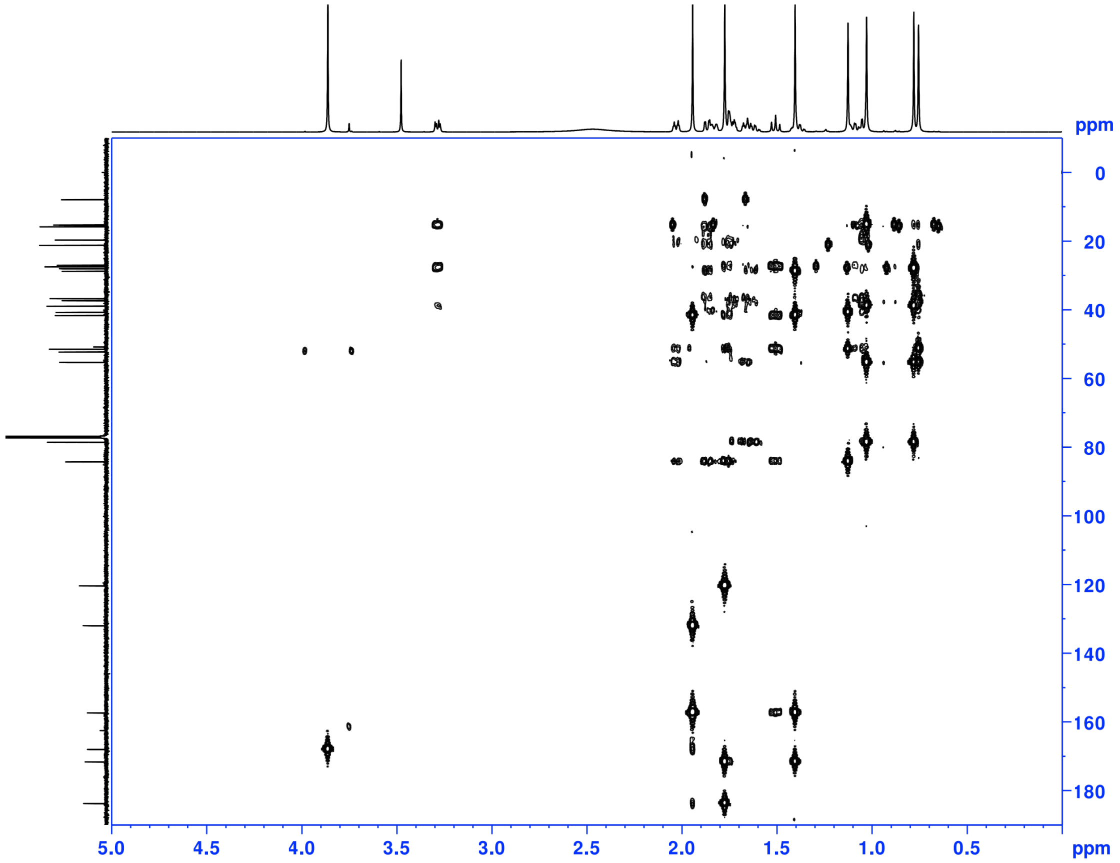

Figure S20. HMBC spectrum of insuetusin B1 (**3**) in $CDCl_3$.

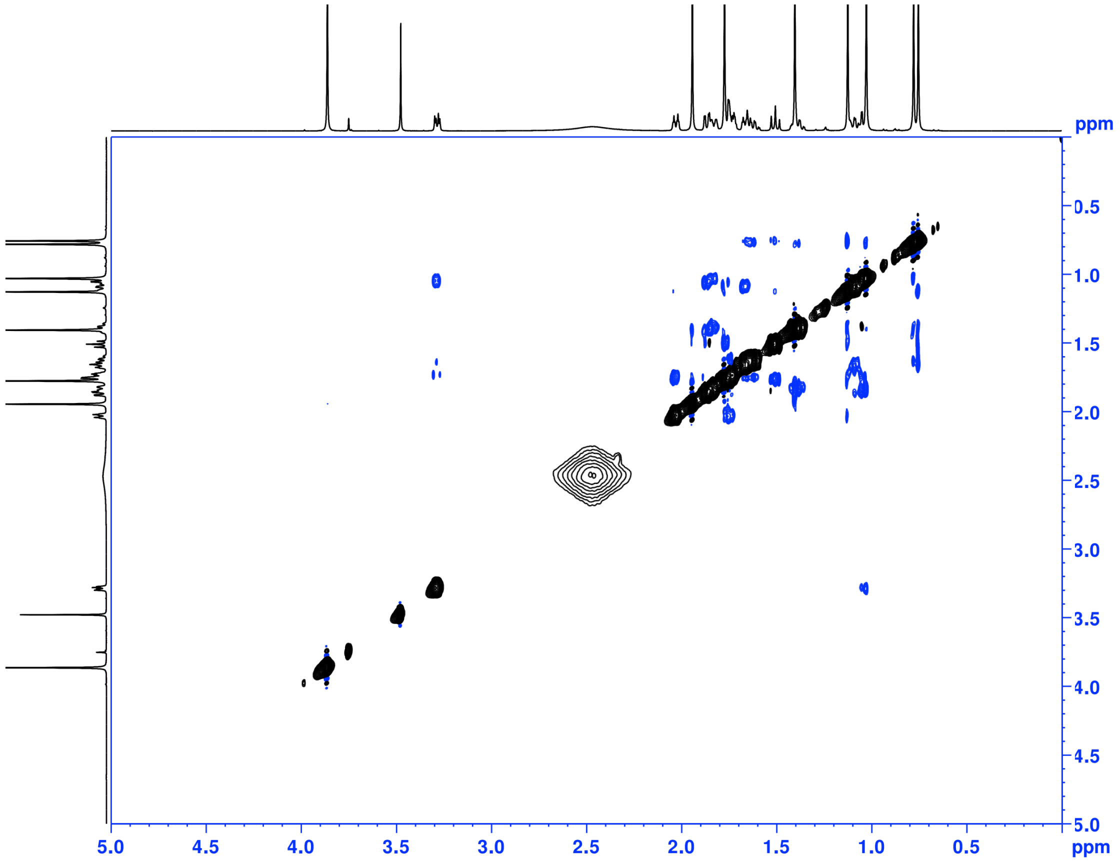

Figure S21. NOESY spectrum of insuetusin B1 (**3**) in $CDCl_3$.

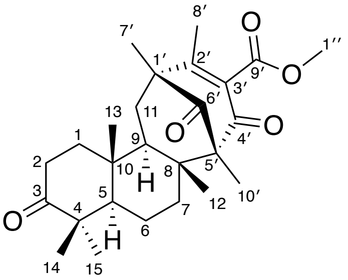

4

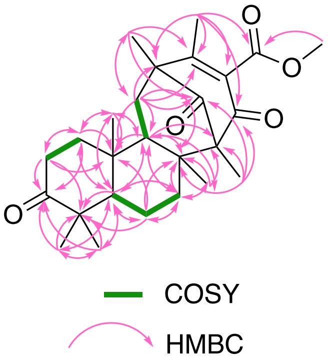

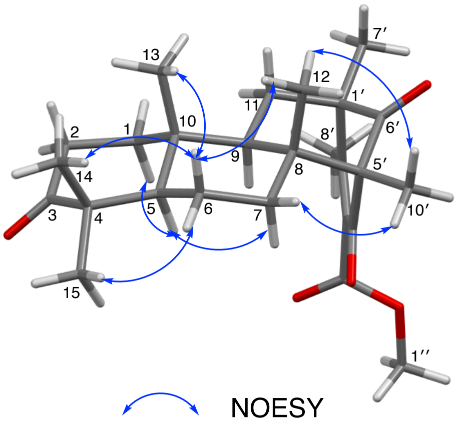

position	δ_C, type	δ_H, mult. (J in Hz)
1	38.9, CH_2	1.86 (α), ddd (13.1, 7.3, 5.3)
		1.37 (β), m
2	33.6, CH_2	2.46, m
3	216.7, C	
4	47.1, C	
5	53.8, CH	1.39, dd (11.9, 1.9)
6	19.5, CH_2	1.55 (α), m
		1.45 (β), m
7	32.7, CH_2	1.62 (α), m
		1.56 (β), m
8	46.2, C	
9	49.5, CH	1.50, dd (11.8, 4.4)
10	36.7, C	
11	34.5, CH_2	1.73, m
12	15.3, CH_3	0.85, s
13	17.3, CH_3	0.96, s
14	20.9, CH_3	1.01, s
15	26.9, CH_3	1.07, s
1′	51.0, C	
2′	158.9, C	
3′	137.5, C	
4′	196.4, C	
5′	69.4, C	
6′	207.9, C	
7′	19.1, CH_3	1.34, s
8′	16.7, CH_3	1.96, s
9′	166.7, C	
10′	9.7, CH_3	1.12, s
1′′	52.5, CH_3	3.85, s

^{1}H NMR: 600 MHz, ^{13}C NMR: 150 MHz (in $CDCl_3$)

Figure S22. NMR data of insuetusin A2 (**4**).

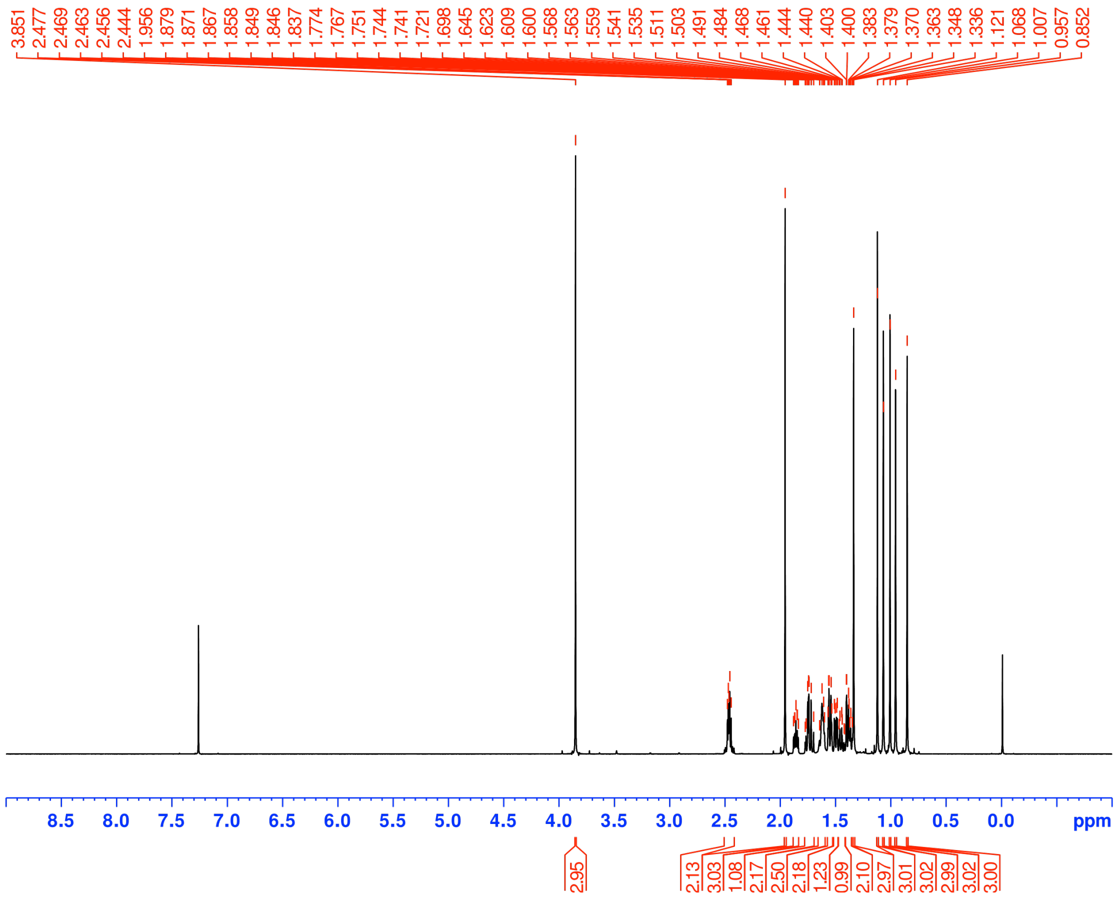

Figure S23. ^{1}H NMR spectrum of insuetusin A2 (**4**) in $CDCl_3$ at 600 MHz.

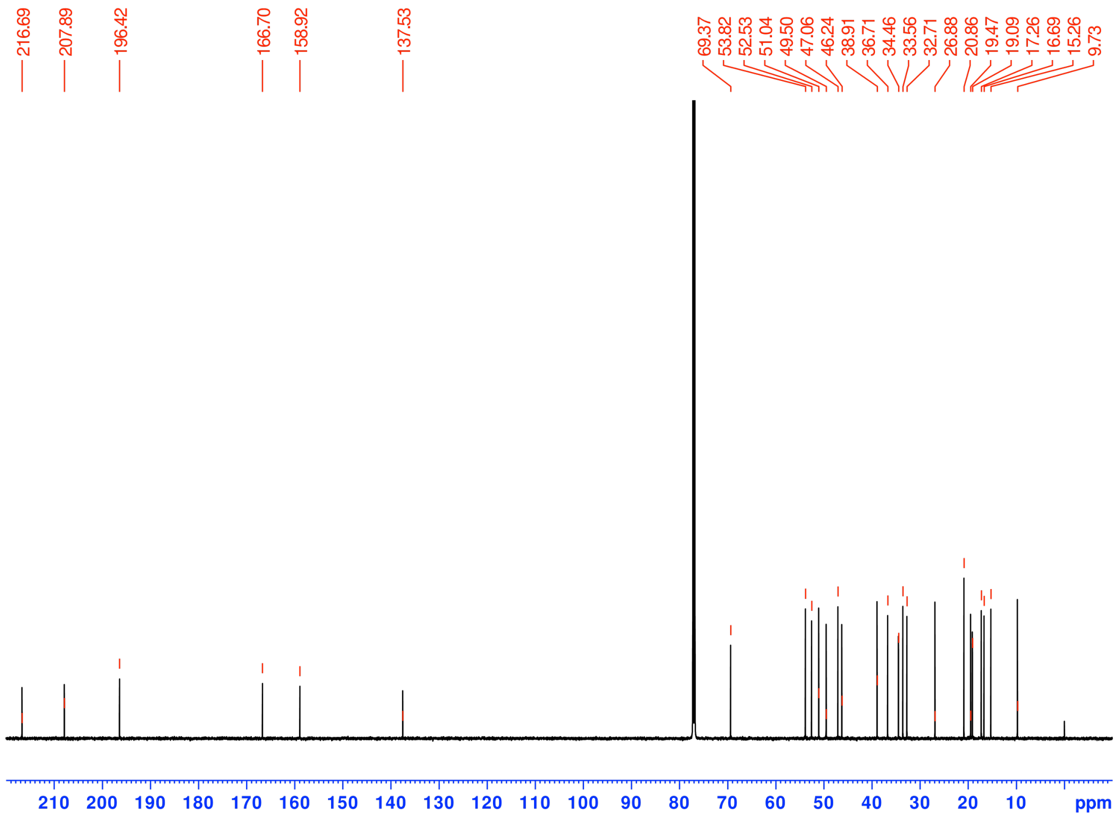

Figure S24. ^{13}C NMR spectrum of insuetusin A2 (**4**) in $CDCl_3$ at 150 MHz.

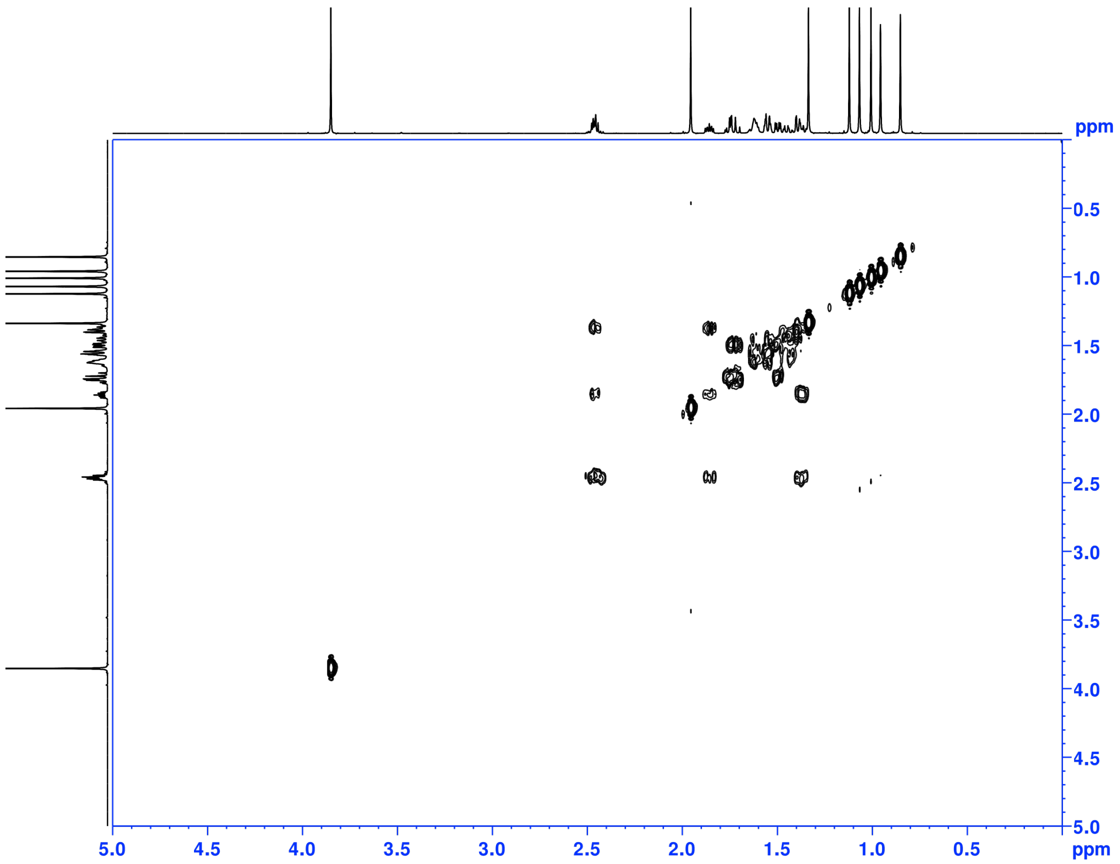

Figure S25. ^{1}H-^{1}H COSY spectrum of insuetusin A2 (**4**) in $CDCl_3$.

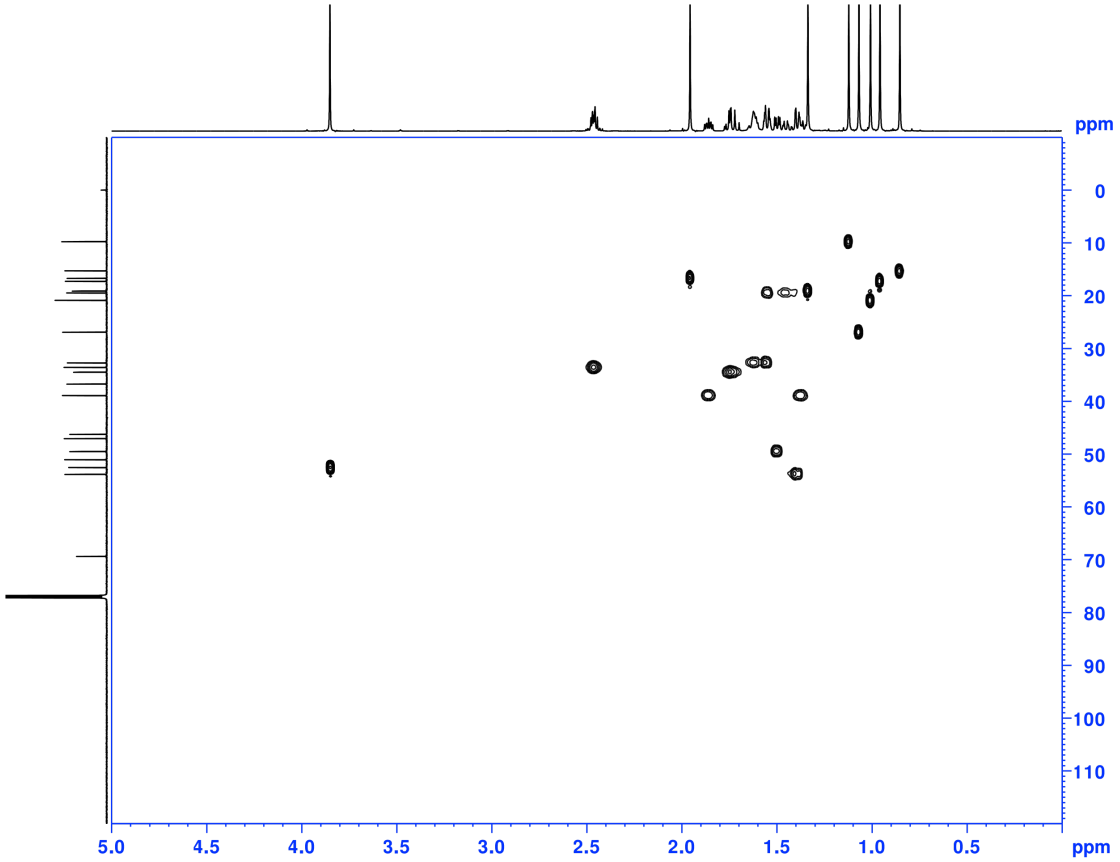

Figure S26. HSQC spectrum of insuetusin A2 (**4**) in $CDCl_3$.

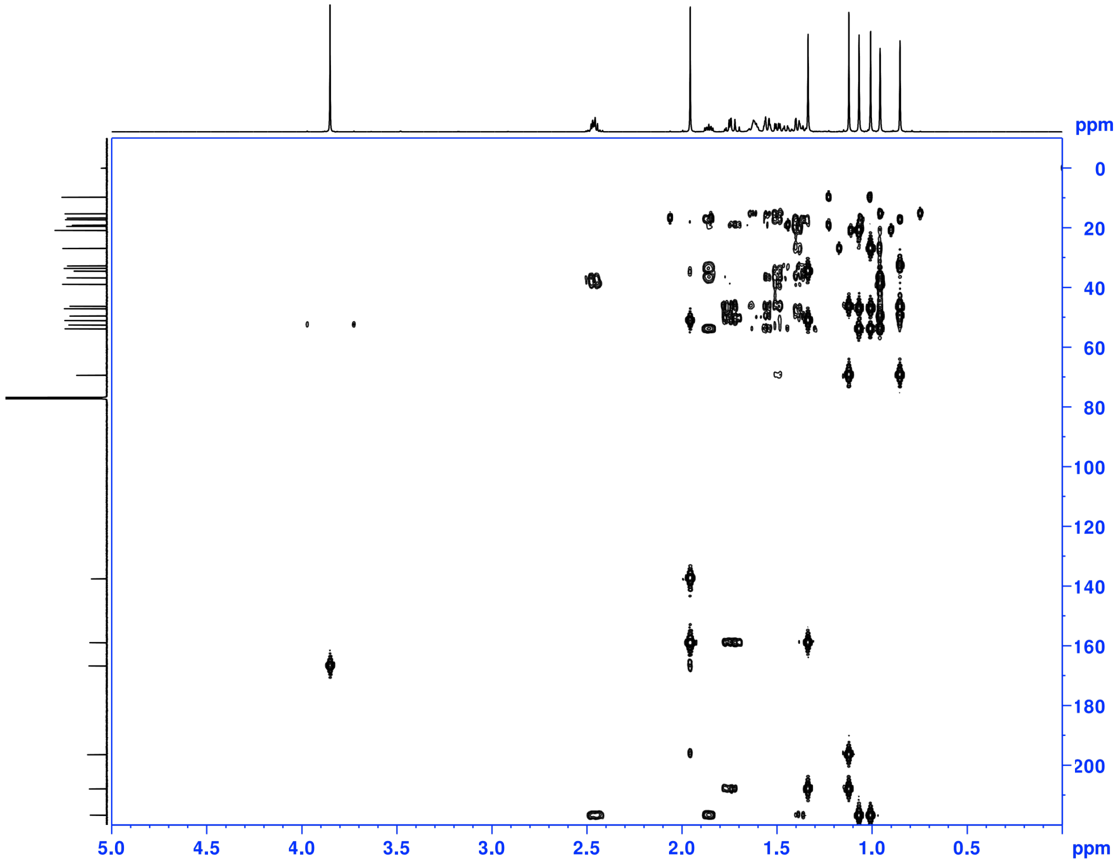

Figure S27. HMBC spectrum of insuetusin A2 (**4**) in $CDCl_3$.

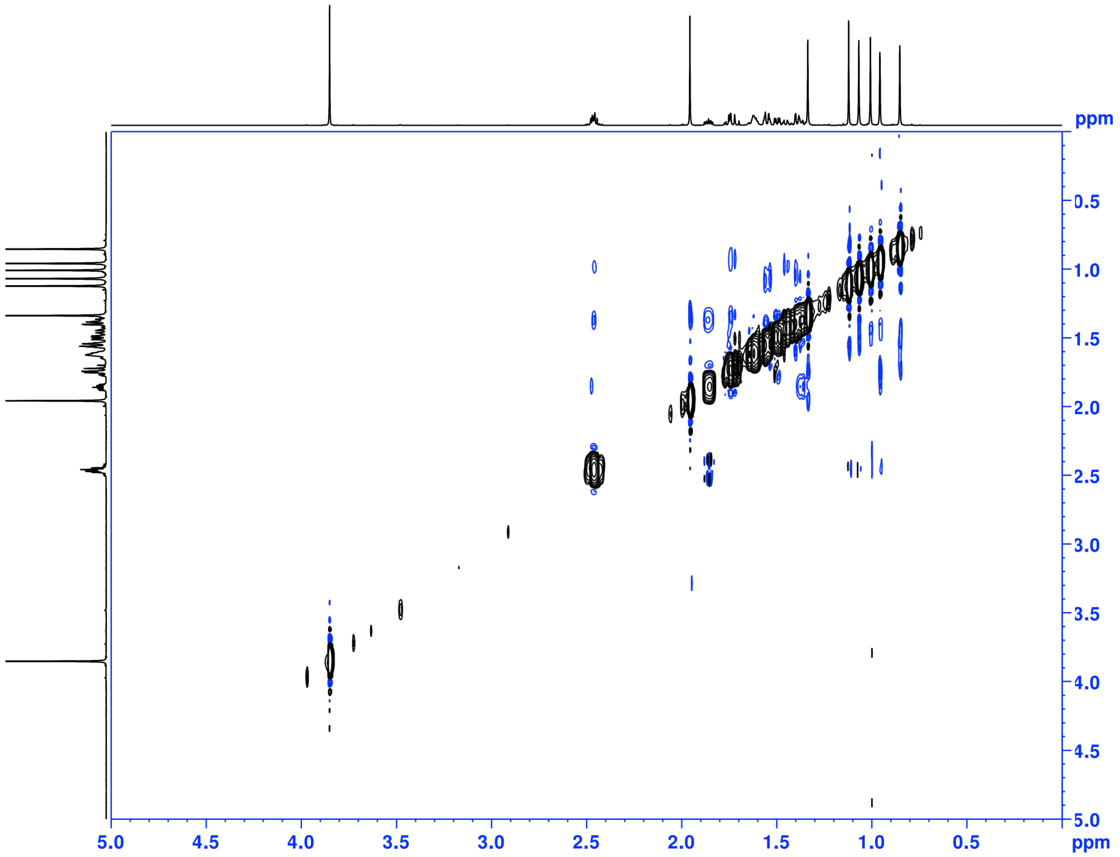

Figure S28. NOESY spectrum of insuetusin A2 (**4**) in $CDCl_3$.

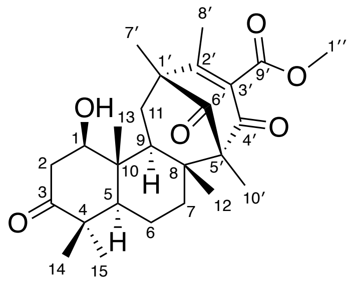

5

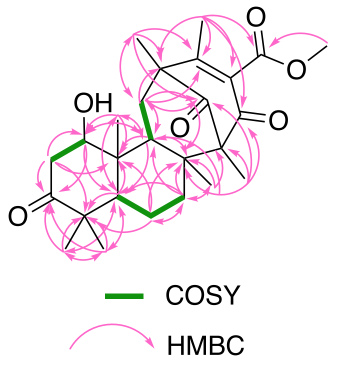

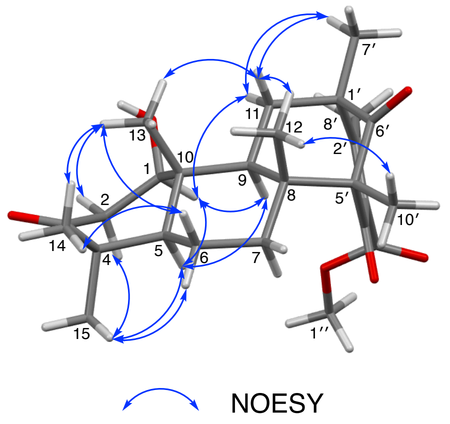

position	δ_C, type	δ_H, mult. (J in Hz)
1	78.9, CH	3.75, dd (7.9, 4.0)
2	45.4, CH_2	2.99 (α), dd (15.0, 8.1)
		2.25 (β), dd (15.0, 4.1)
3	214.6, C	
4	46.9, C	
5	50.4, CH	1.32, dd (12.0, 2.7)
6	18.8, CH_2	1.59 (α), m
		1.47 (β), m
7	32.2, CH_2	1.57, m
8	47.0, C	
9	50.1, CH	1.60, m
10	42.7, C	
11	35.9, CH_2	2.52 (α), dd (14.3, 3.4)
		1.77 (β), dd (14.2, 12.8)
12	15.4, CH_3	0.85, s
13	12.5, CH_3	0.88, s
14	20.1, CH_3	1.02, s
15	27.7, CH_3	1.05, s
1′	51.3, C	
2′	159.9, C	
3′	137.2, C	
4′	196.6, C	
5′	69.4, C	
6′	208.1, C	
7′	19.0, CH_3	1.33, s
8′	16.9, CH_3	1.98, s
9′	166.9, C	
10′	9.8, CH_3	1.12, s
1′′	52.5, CH_3	3.85, s

^{1}H NMR: 600 MHz, ^{13}C NMR: 150 MHz (in $CDCl_3$)

Figure S29. NMR data of insuetusin A3 (**5**).

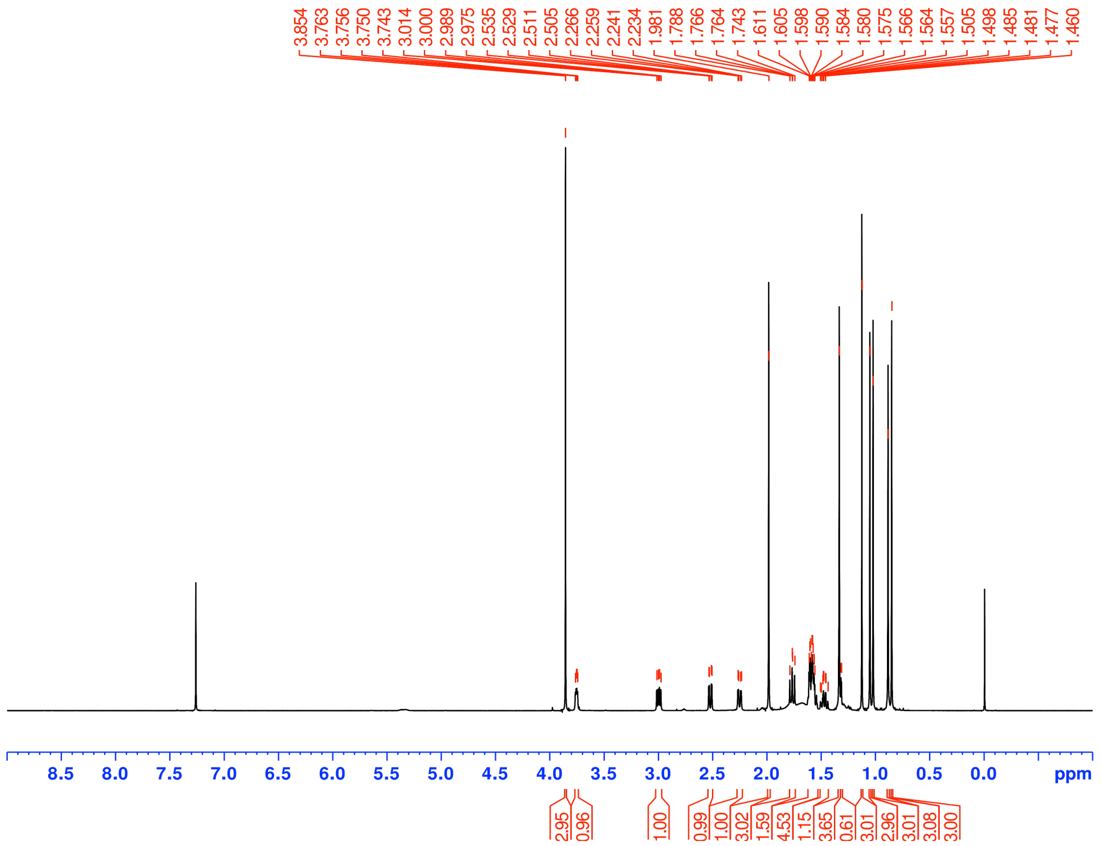

Figure S30. ^{1}H NMR spectrum of insuetusin A3 (**5**) in $CDCl_3$ at 600 MHz.

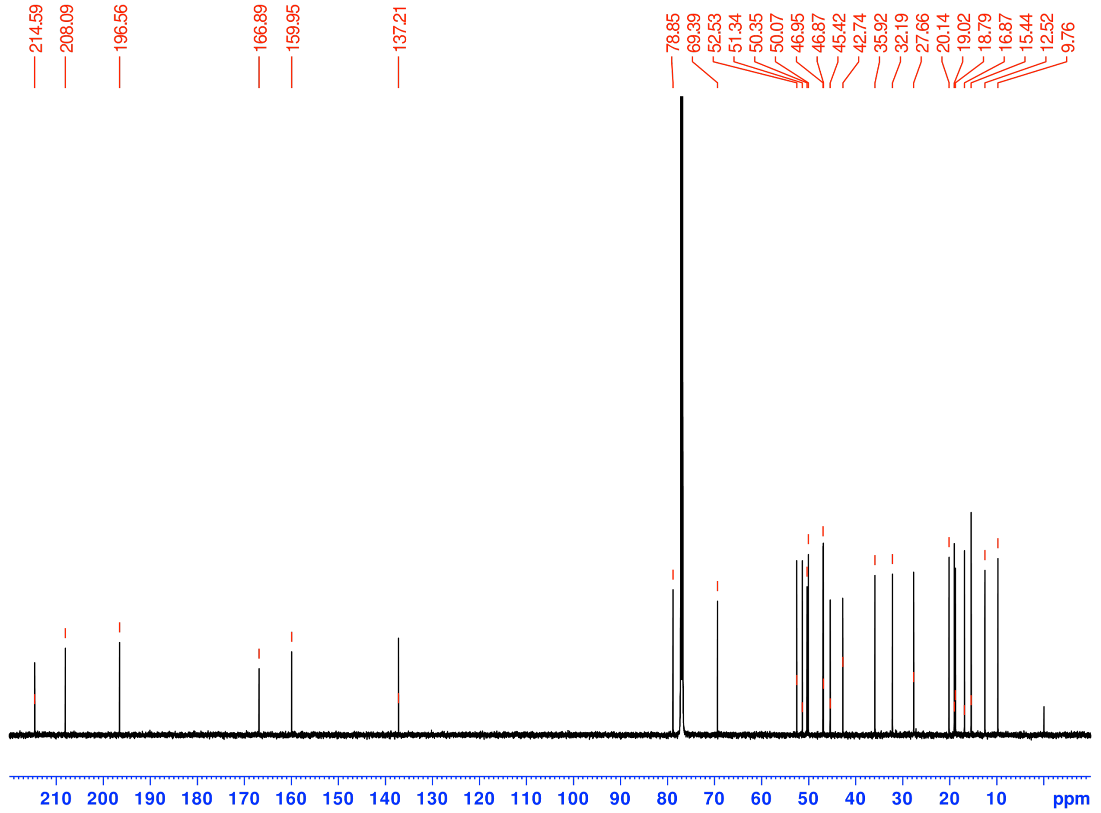

Figure S31. ^{13}C NMR spectrum of insuetusin A3 (**5**) in $CDCl_3$ at 150 MHz.

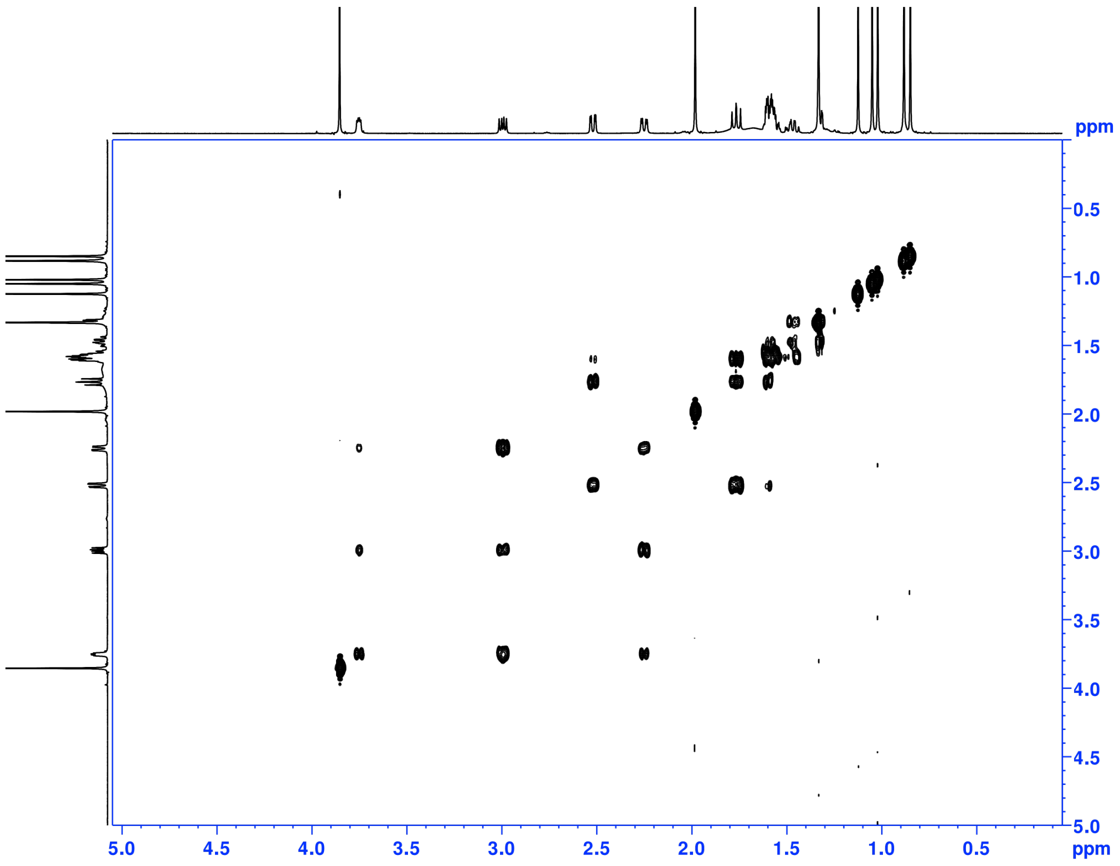

Figure S32. ^{1}H-^{1}H COSY spectrum of insuetusin A3 (**5**) in $CDCl_3$.

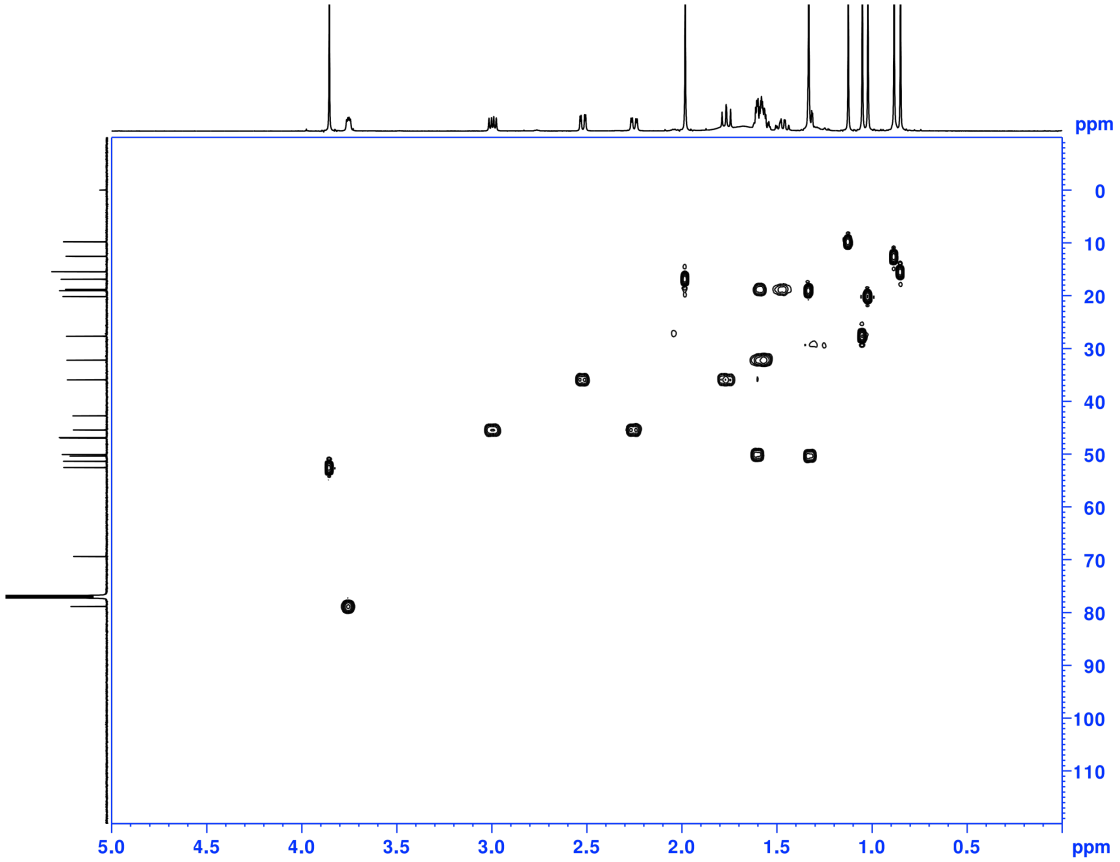

Figure S33. HSQC spectrum of insuetusin A3 (**5**) in $CDCl_3$.

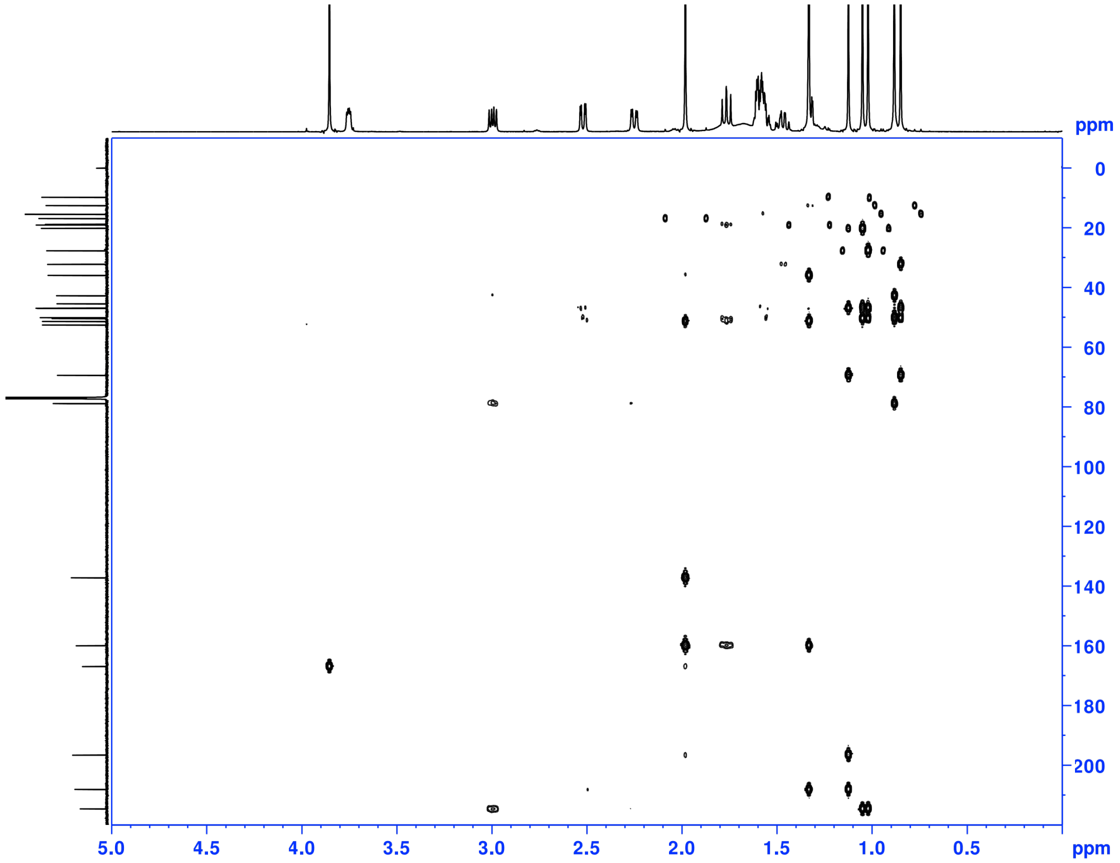

Figure S34. HMBC spectrum of insuetusin A3 (**5**) in $CDCl_3$.

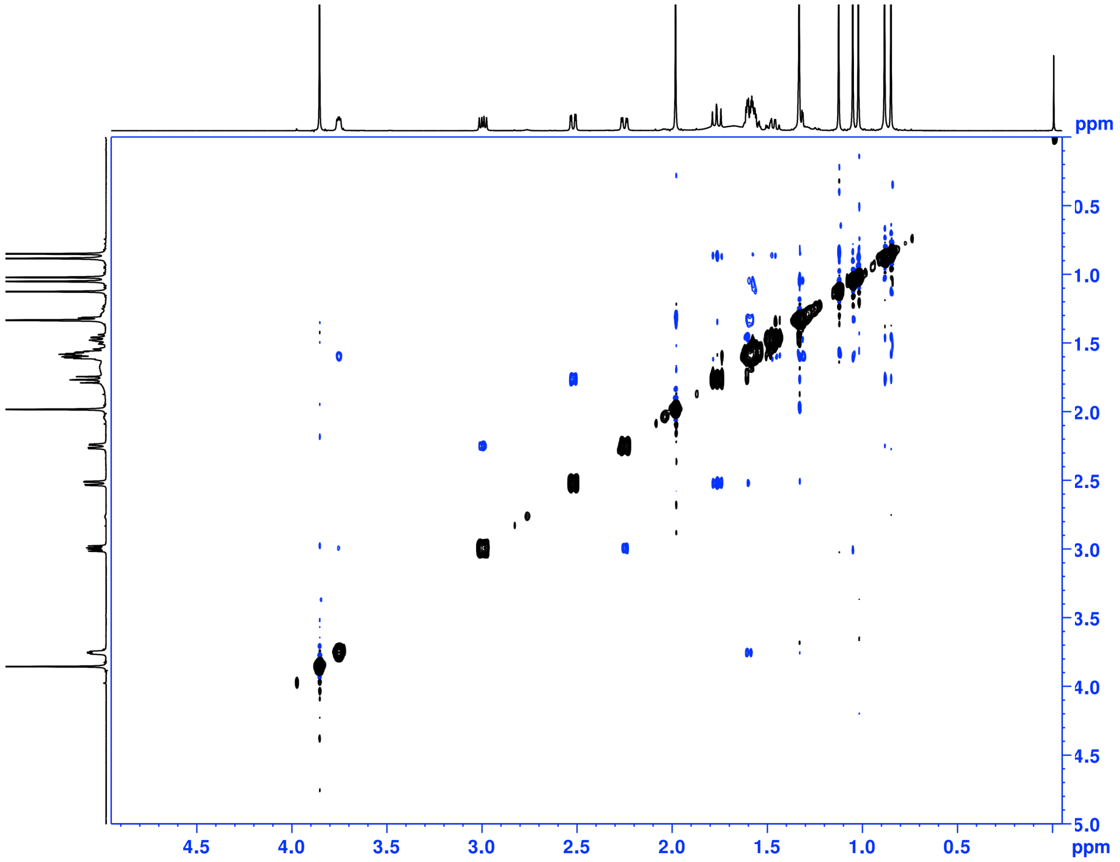

Figure S35. NOESY spectrum of insuetusin A3 (**5**) in $CDCl_3$.

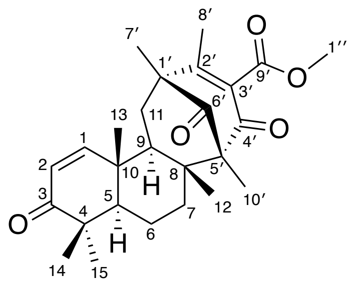

6

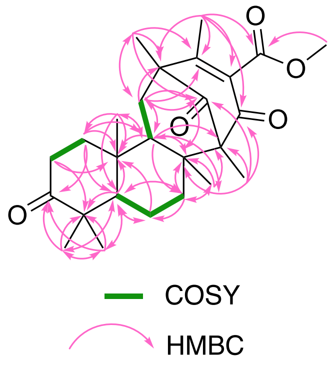

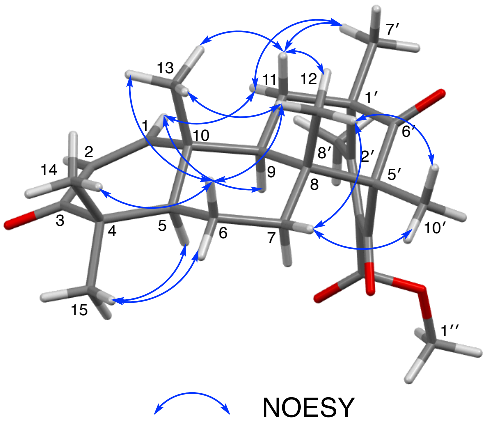

position	δ_C, type	δ_H, mult. (*J* in Hz)
1	156.2, CH	6.91, d (10.1)
2	126.5, CH	5.85, d (10.1)
3	204.6, C	
4	44.7, C	
5	52.8, CH	1.58, dd (12.5, 1.4)
6	18.7, CH_2	1.62 (α), m
		1.51 (β), td (12.5, 2.5)
7	32.7, CH_2	1.68 (α), td (11.5, 2.7)
		1.58 (β), m
8	46.2, C	
9	44.5, CH	1.67, dd (12.6, 2.9)
10	39.3, C	
11	33.9, CH_2	1.96 (α), dd (13.6, 2.9)
		1.82 (β), t (13.0)
12	15.9, CH_3	0.89, s
13	20.7, CH_3	1.12, s
14	21.4, CH_3	1.08, s
15	27.8, CH_3	1.12, s
1′	51.0, C	
2′	158.8, C	
3′	137.7, C	
4′	196.1, C	
5′	69.2, C	
6′	207.4, C	
7′	19.2, CH_3	1.39, s
8′	16.8, CH_3	2.01, s
9′	166.5, C	
10′	9.8, CH_3	1.15, s
1′′	52.5, CH_3	3.83, s

^{1}H NMR: 600 MHz, ^{13}C NMR: 150 MHz (in $CDCl_3$)

Figure S36. NMR data of insuetusin A4 (**6**).

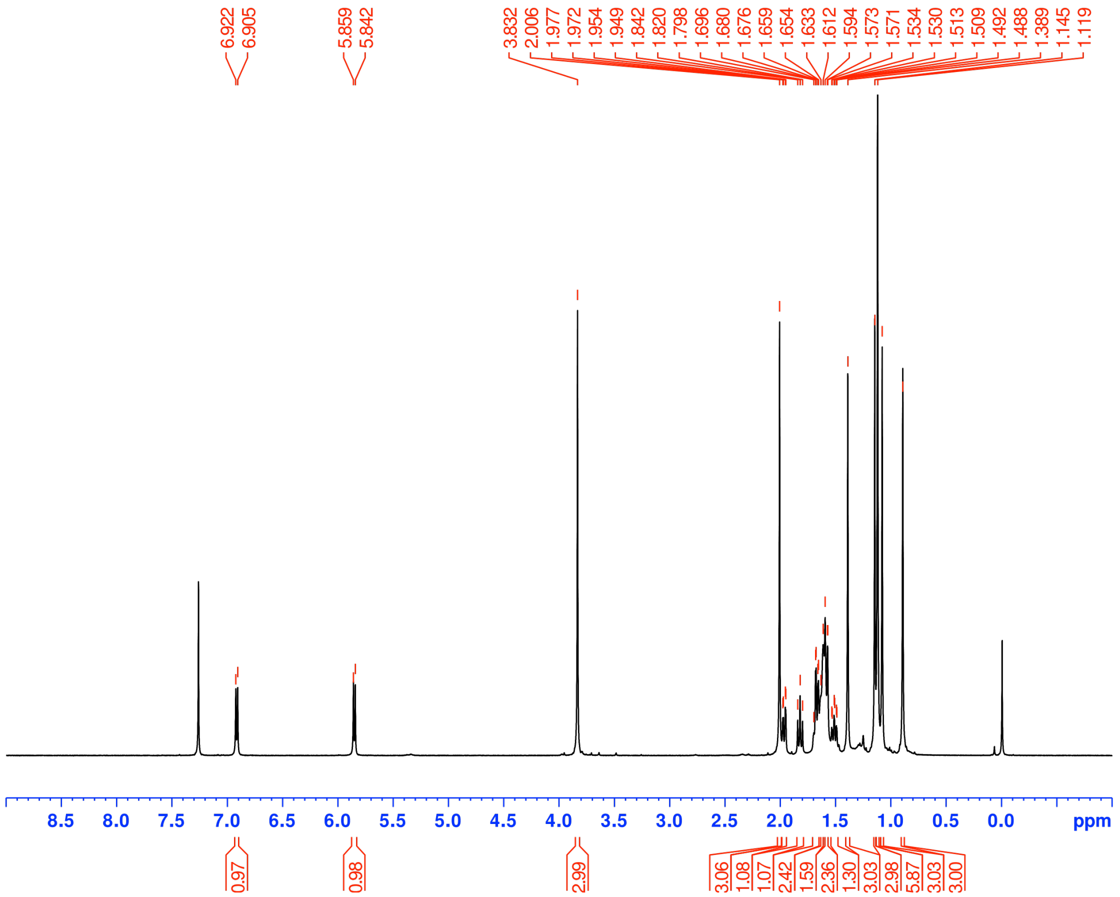

Figure S37. ¹H NMR spectrum of insuetusin A4 (**6**) in $CDCl_3$ at 600 MHz.

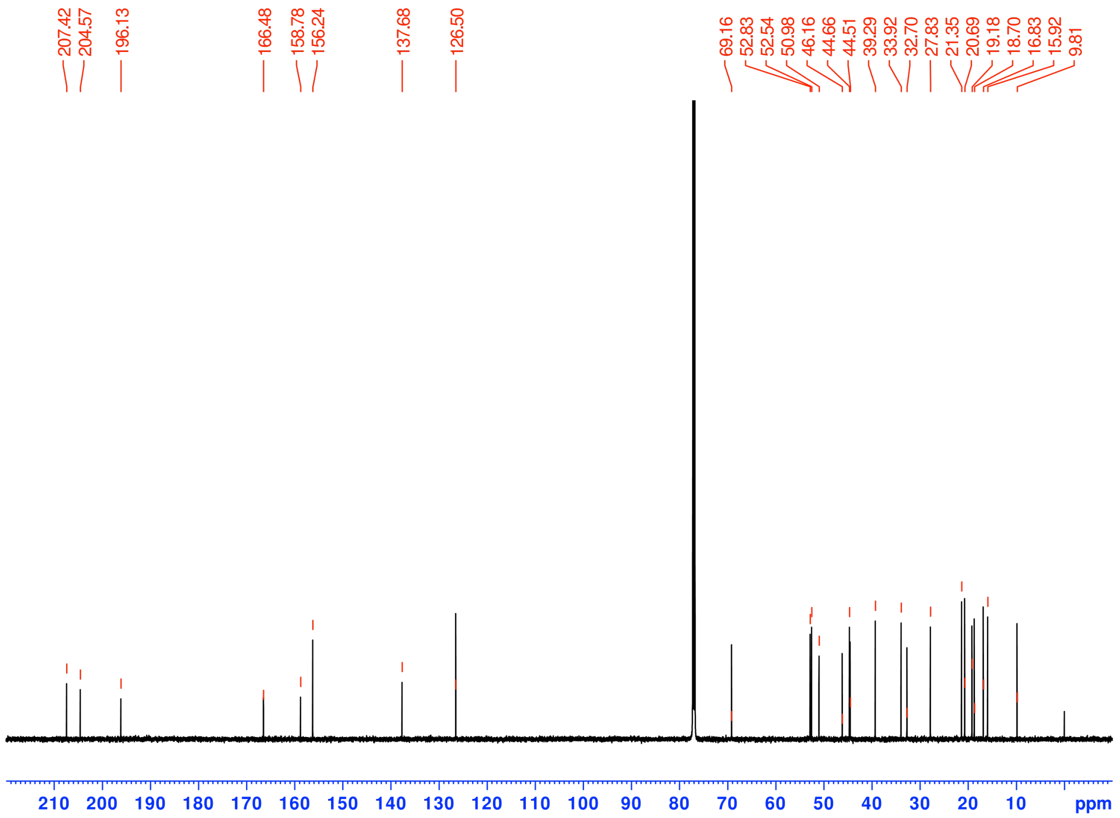

Figure S38. ¹³C NMR spectrum of insuetusin A4 (**6**) in $CDCl_3$ at 150 MHz.

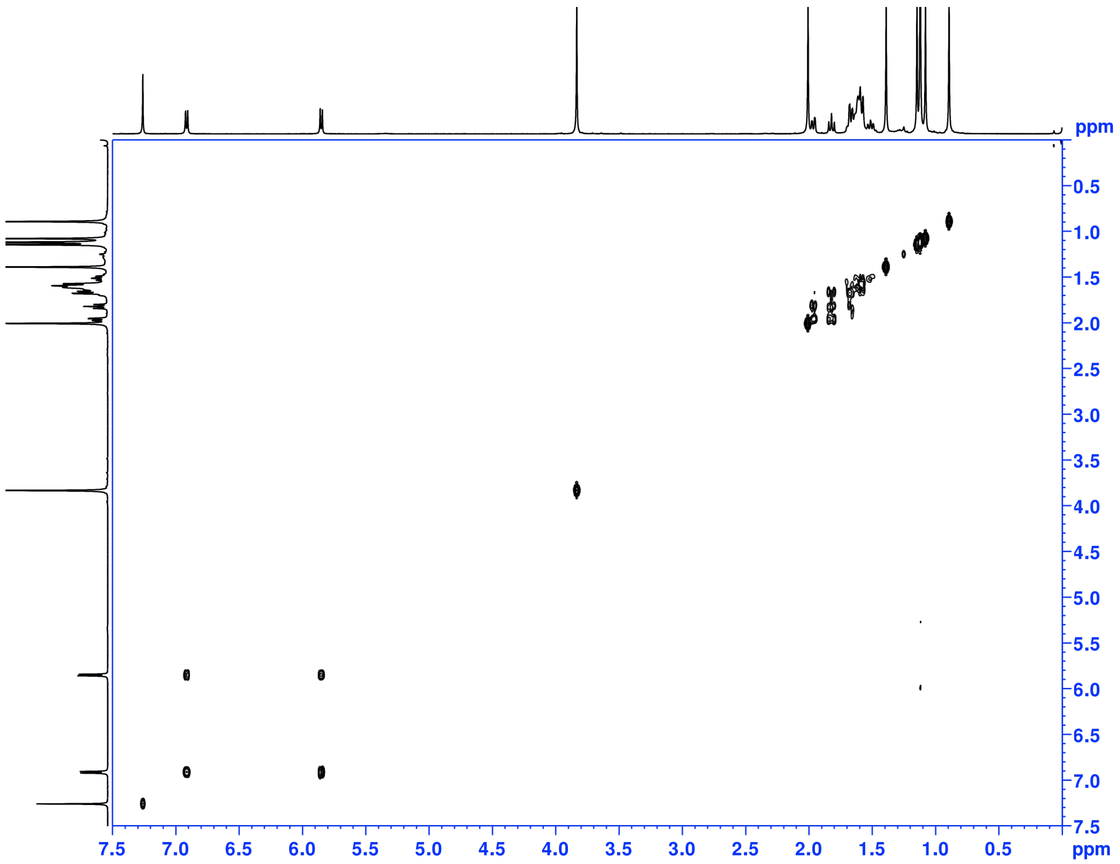

Figure S39. ^{1}H-^{1}H COSY spectrum of insuetusin A4 (**6**) in $CDCl_3$.

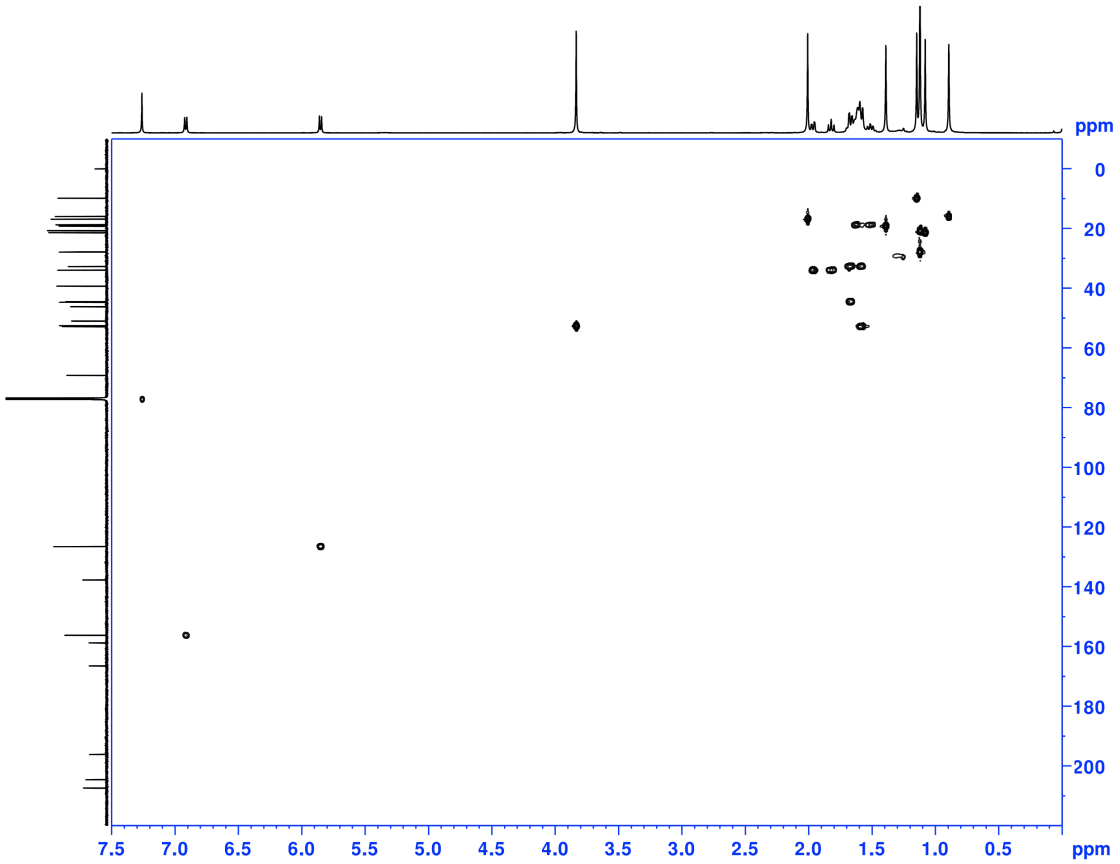

Figure S40. HSQC spectrum of insuetusin A4 (**6**) in $CDCl_3$.

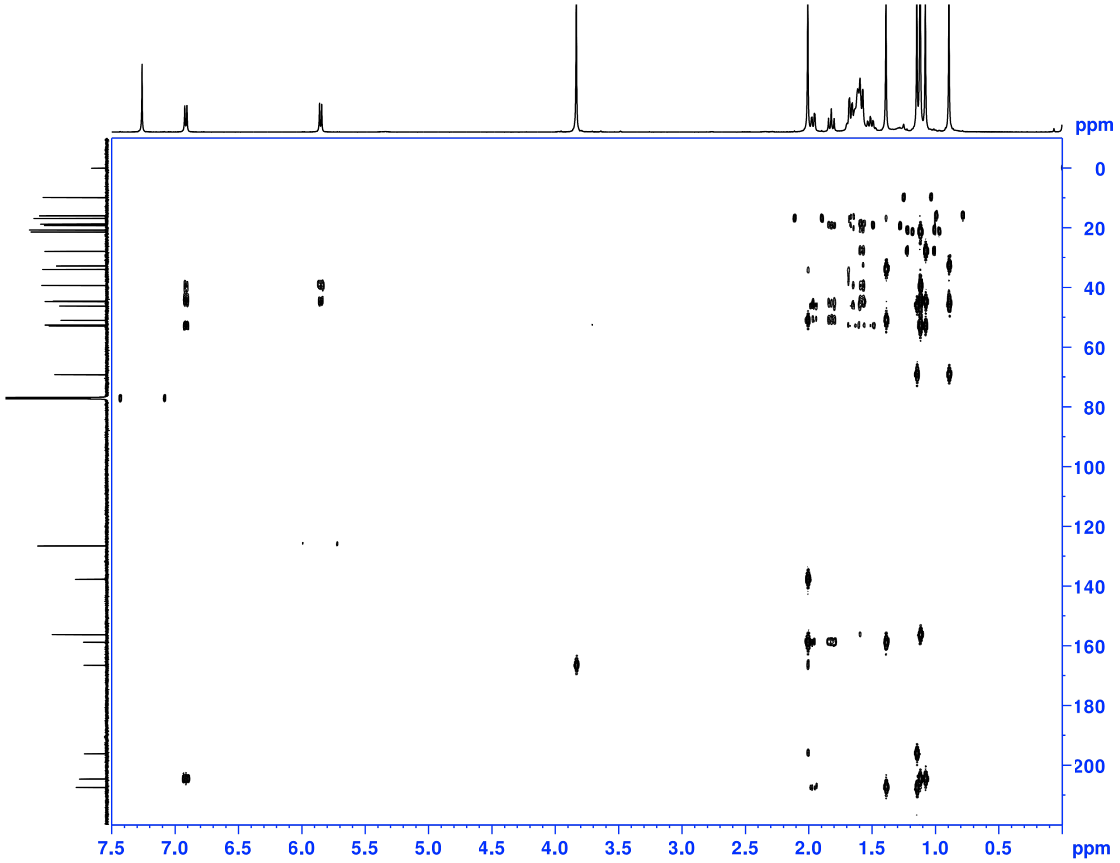

Figure S41. HMBC spectrum of insuetusin A4 (**6**) in $CDCl_3$.

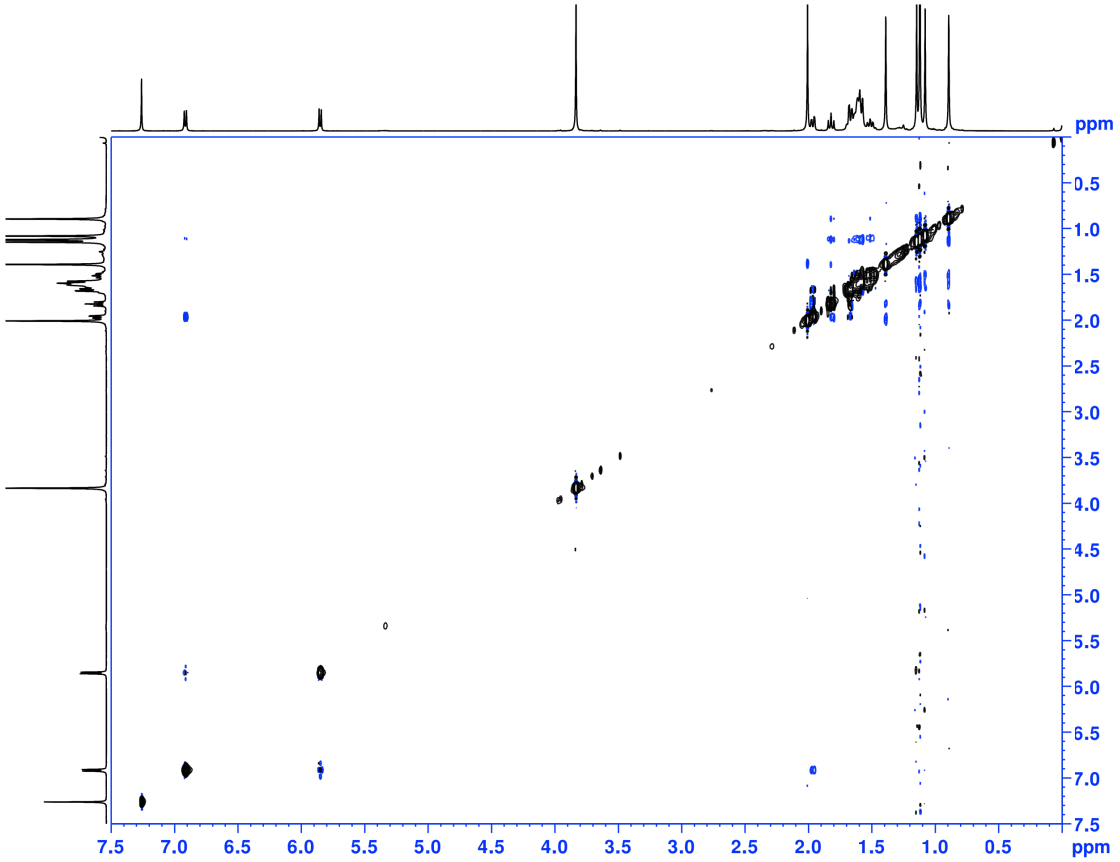

Figure S42. NOESY spectrum of insuetusin A4 (**6**) in $CDCl_3$.

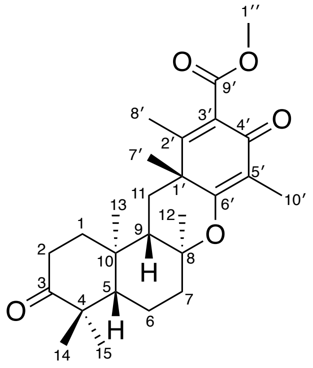

7

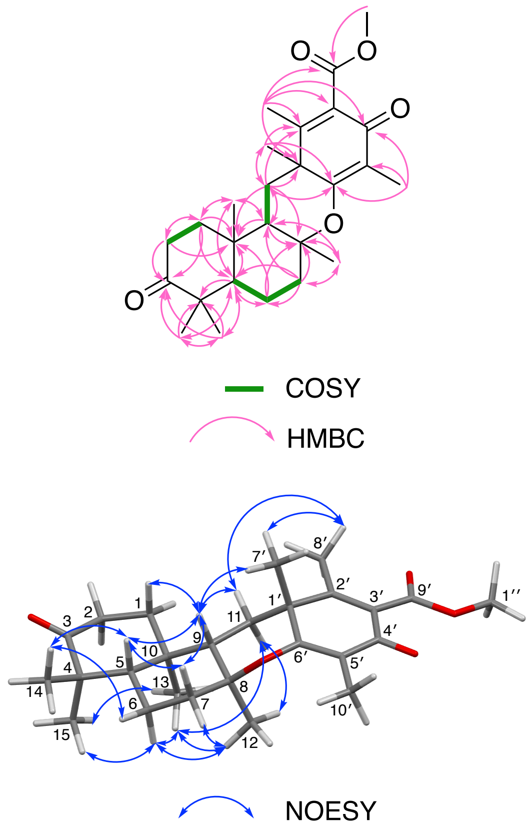

position	δ_C, type	δ_H, mult. (J in Hz)
1	37.7, CH_2	1.92 (α), ddd (13.1, 7.4, 4.5)
		1.56 (β), m
2	33.5, CH_2	2.54, m
3	216.2, C	
4	47.2, C	
5	54.5, CH	1.67, dd (12.4, 2.1)
6	20.9, CH_2	1.49 (α), qd (13.0, 2.3)
		1.77 (β), m
7	40.0, CH_2	2.07 (α), dt (12.8, 3.7)
		1.80 (β), m
8	83.7, C	
9	50.9, CH	1.97, dd (13.1, 2.8)
10	36.5, C	
11	28.9, CH_2	1.57 (α), t (13.1)
		1.77 (β), m
12	20.75, CH_3	1.17, s
13	15.4, CH_3	0.86, s
14	26.7, CH_3	1.14, s
15	20.9, CH_3	1.04, s
1′	41.6, C	
2′	156.9, C	
3′	132.1, C	
4′	183.6, C	
5′	120.6, C	
6′	171.0, C	
7′	27.2, CH_3	1.42, s
8′	15.3, CH_3	1.96, s
9′	167.9, C	
10′	7.9, CH_3	1.79, s
1′′	52.2, CH_3	3.87, s

^{1}H NMR: 600 MHz, ^{13}C NMR: 150 MHz (in $CDCl_3$)

Figure S43. NMR data of insuetusin B2 (**7**).

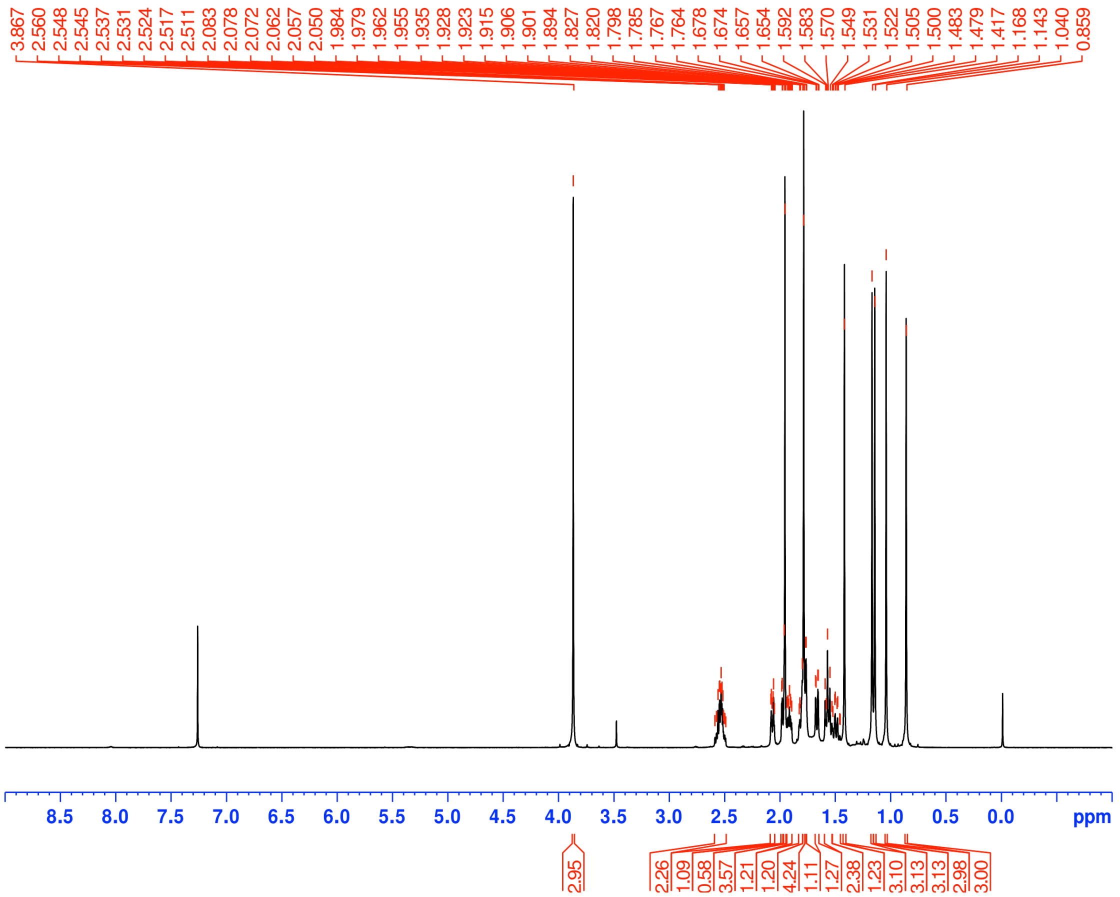

Figure S44. ^{1}H NMR spectrum of insuetusin B2 (**7**) in $CDCl_3$ at 600 MHz.

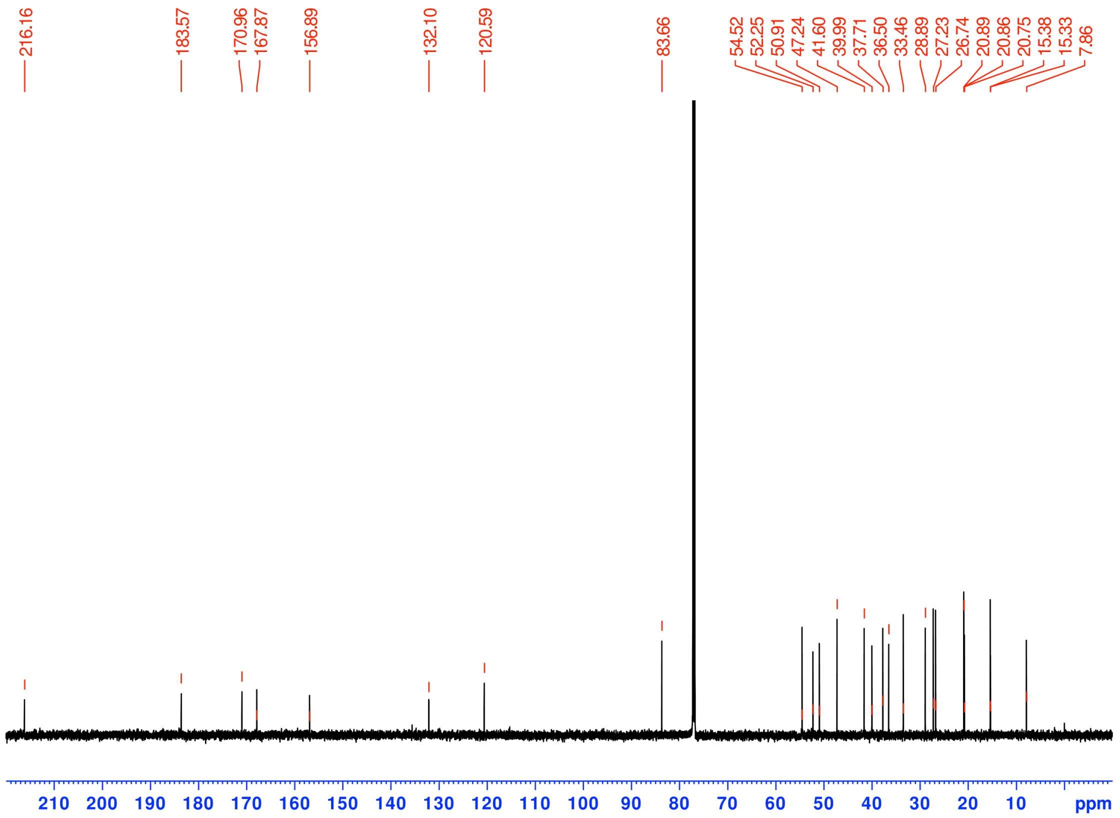

Figure S45. ^{13}C NMR spectrum of insuetusin B2 (**7**) in $CDCl_3$ at 150 MHz.

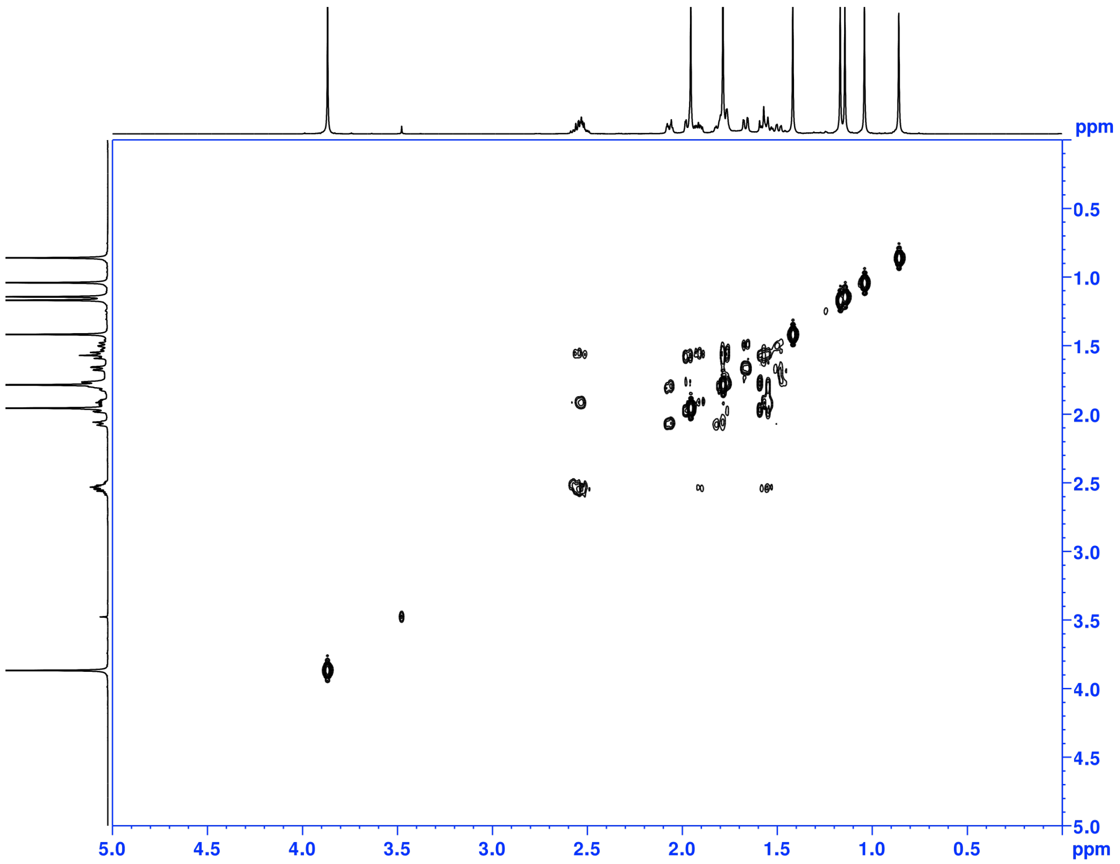

Figure S46. ^{1}H-^{1}H COSY spectrum of insuetusin B2 (**7**) in $CDCl_3$.

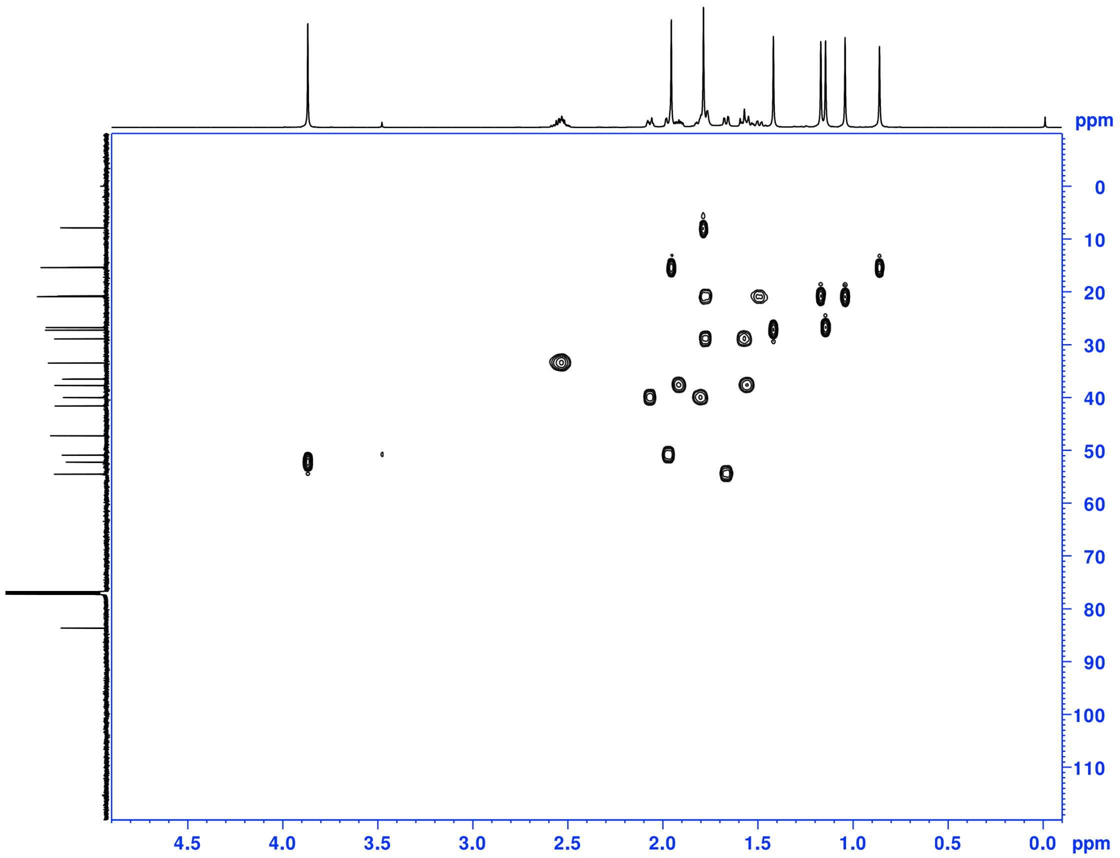

Figure S47. HSQC spectrum of insuetusin B2 (**7**) in $CDCl_3$.

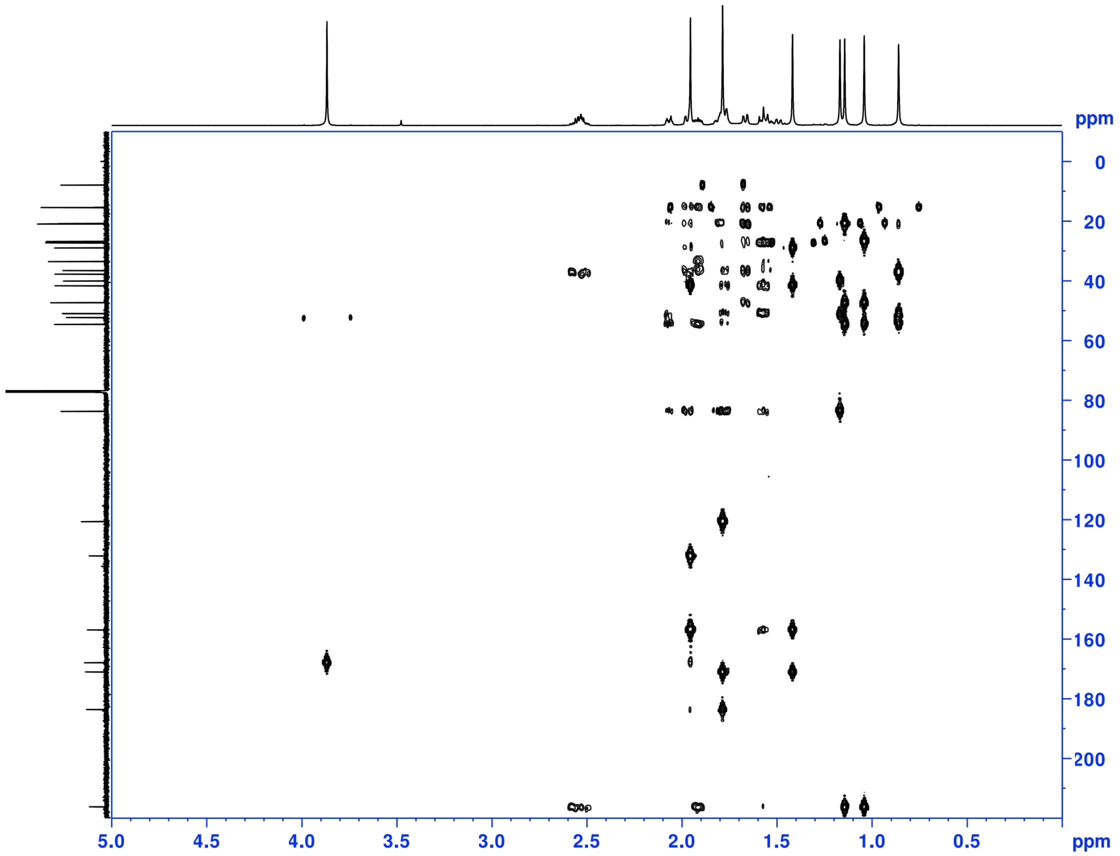

Figure S48. HMBC spectrum of insuetusin B2 (**7**) in $CDCl_3$.

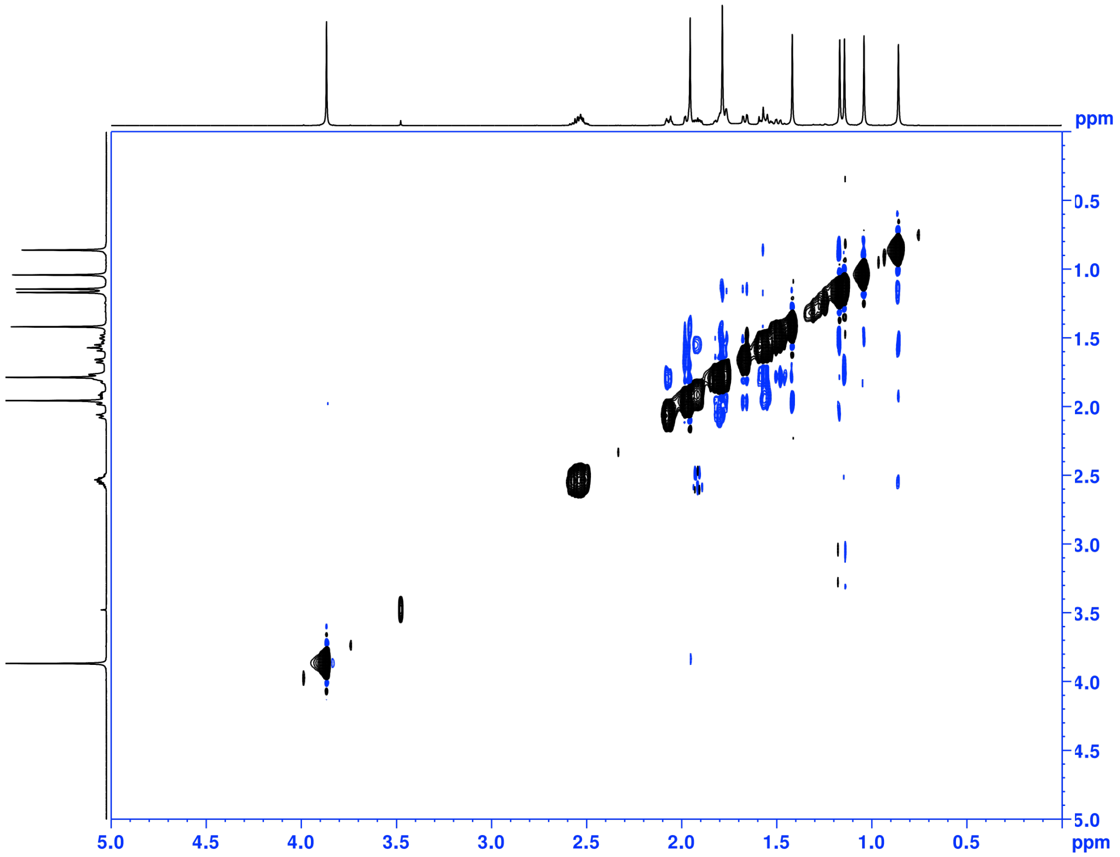

Figure S49. NOESY spectrum of insuetusin B2 (**7**) in $CDCl_3$.

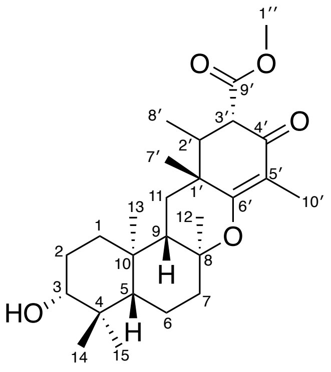

8

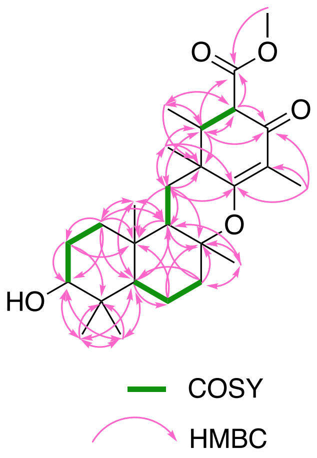

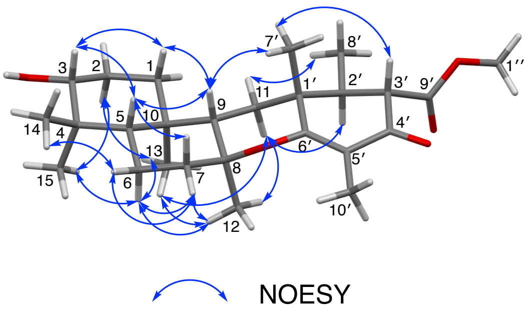

position	δ_C, type	δ_H, mult. (J in Hz)
1	37.1, CH_2	1.67 (α), m
		1.04 (β), m
2	27.0, CH_2	1.62 (α), qd (13.0, 3.3)
		1.72 (β), m
3	78.5, C	3.27, dd (11.5, 4.1)
4	38.9, C	
5	55.3, CH	1.02, dd (12.3, 2.1)
6	19.5, CH_2	1.39 (α), qd (13.8, 3.3)
		1.81 (β), dq (13.9, 3.2)
7	40.8, CH_2	2.02 (α), dt (12.4, 3.1)
		1.69 (β), m
8	82.6, C	
9	50.5, CH	1.72, dd (13.2, 3.0)
10	36.5, C	
11	31.0, CH_2	1.40 (α), t (13.2)
		1.65 (β), m
12	22.1, CH_3	1.20, s
13	15.6, CH_3	0.79, s
14	28.0, CH_3	1.03, s
15	15.3, CH_3	0.79, s
1′	37.6, C	
2′	40.9, CH	2.38, dq (13.0, 6.7)
3′	57.9, CH	3.27, d (12.9)
4′	193.7, C	
5′	117.9, C	
6′	175.6, C	
7′	19.9, CH_3	1.15, s
8′	12.5, CH_3	0.93, d (6.7)
9′	171.6, C	
10′	8.1, CH_3	1.66, s
1′′	52.1, CH_3	3.78, s

^{1}H NMR: 600 MHz, ^{13}C NMR: 150 MHz (in $CDCl_3$)

Figure S50. NMR data of insuetusin B3 (**8**).

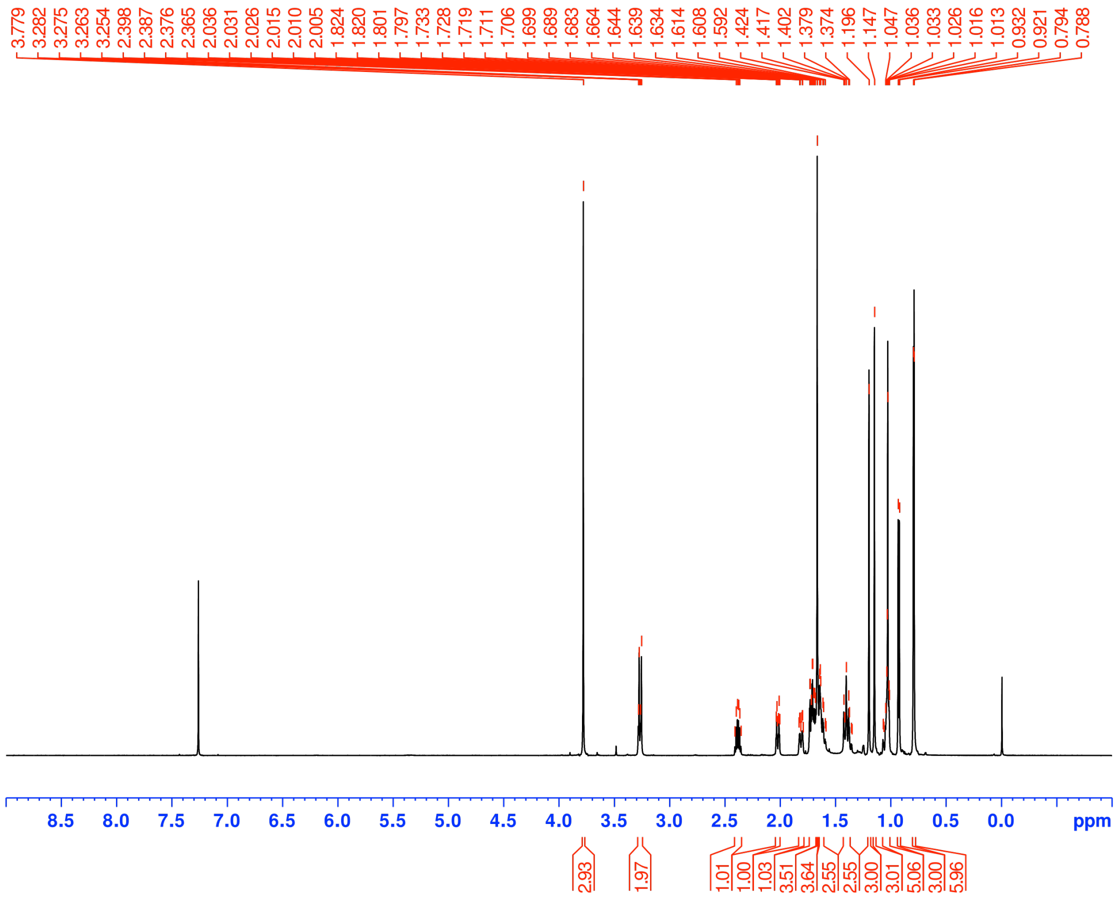

Figure S51. ^{1}H NMR spectrum of insuetusin B3 (**8**) in $CDCl_3$ at 600 MHz.

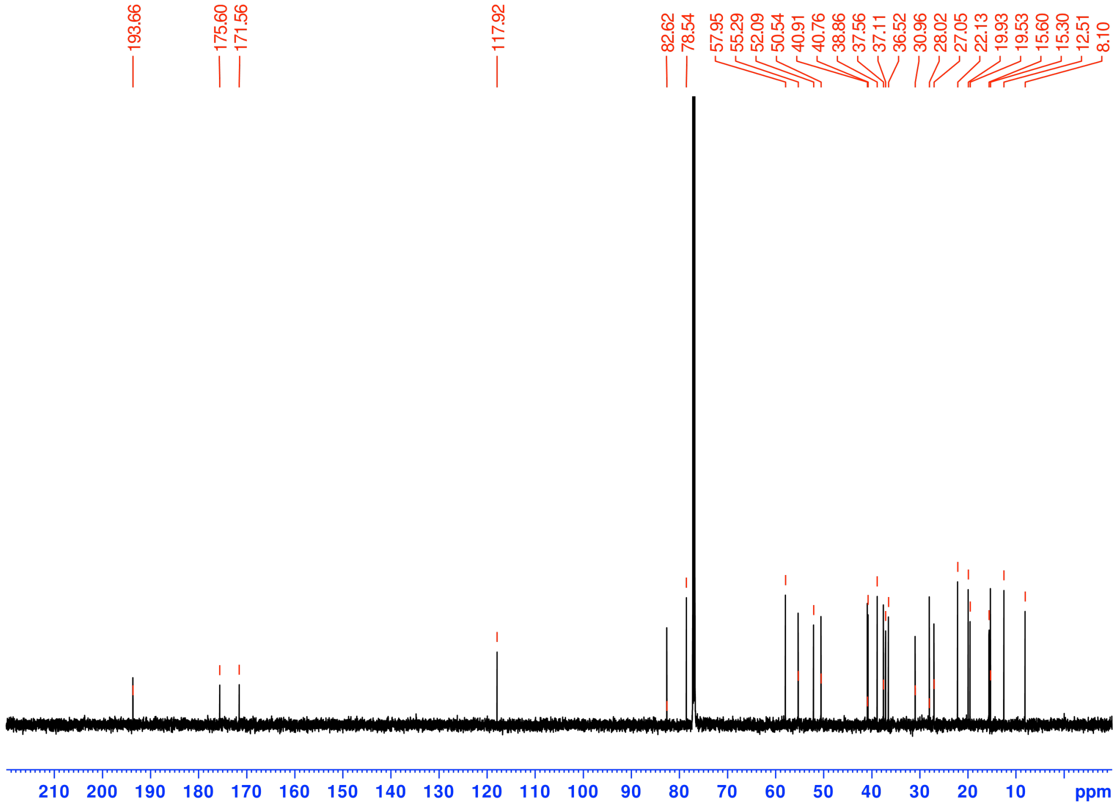

Figure S52. ^{13}C NMR spectrum of insuetusin B3 (**8**) in $CDCl_3$ at 150 MHz.

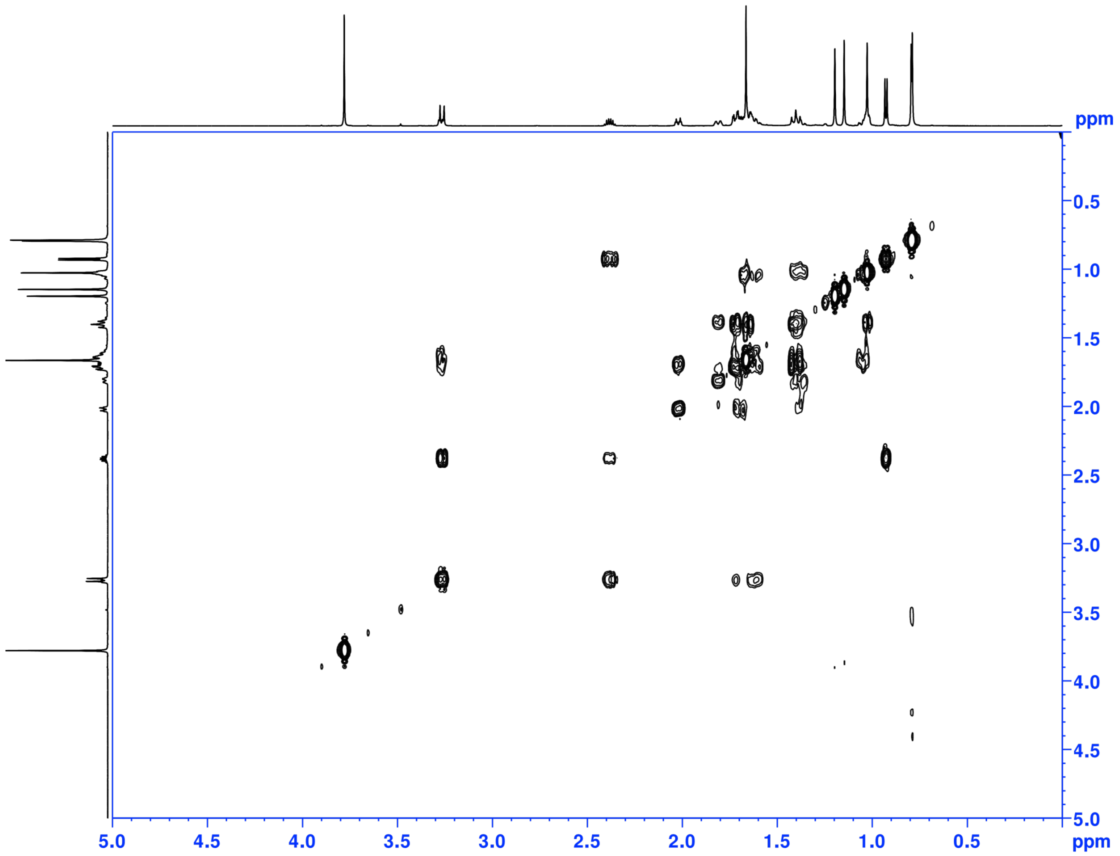

Figure S53. ^{1}H-^{1}H COSY spectrum of insuetusin B3 (**8**) in $CDCl_3$.

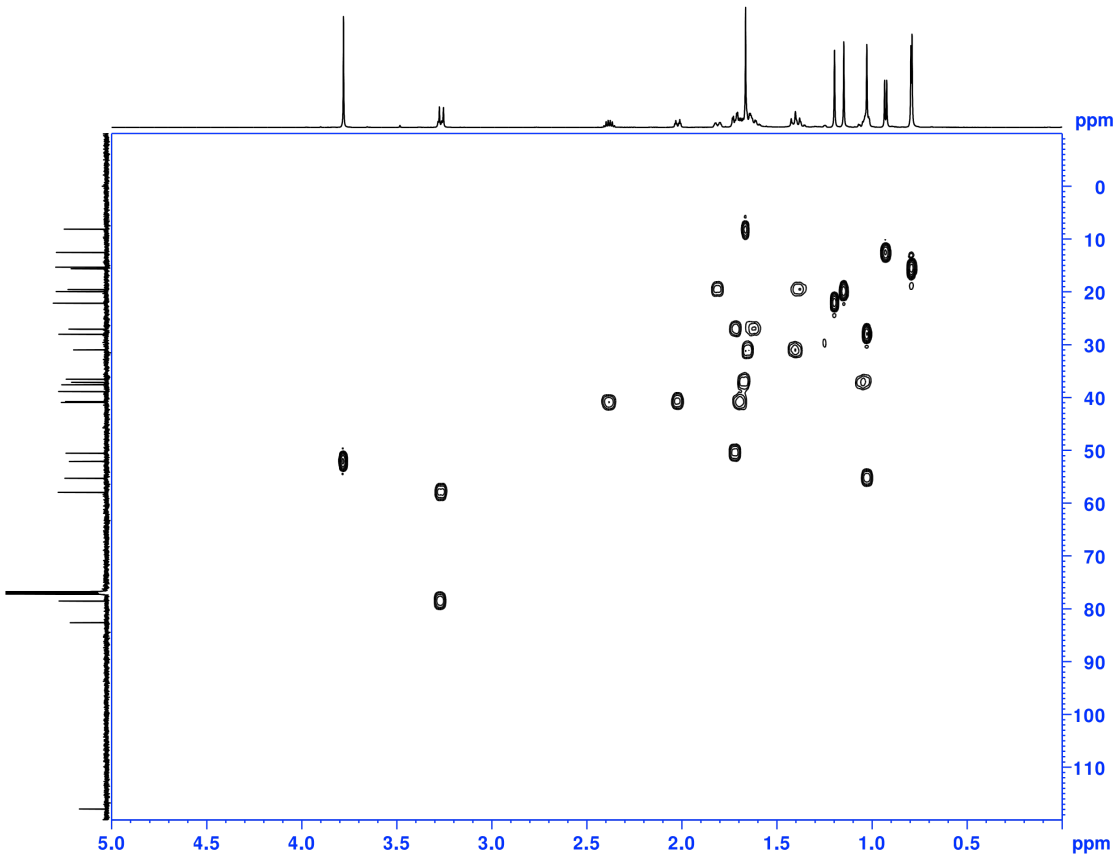

Figure S54. HSQC spectrum of insuetusin B3 (**8**) in $CDCl_3$.

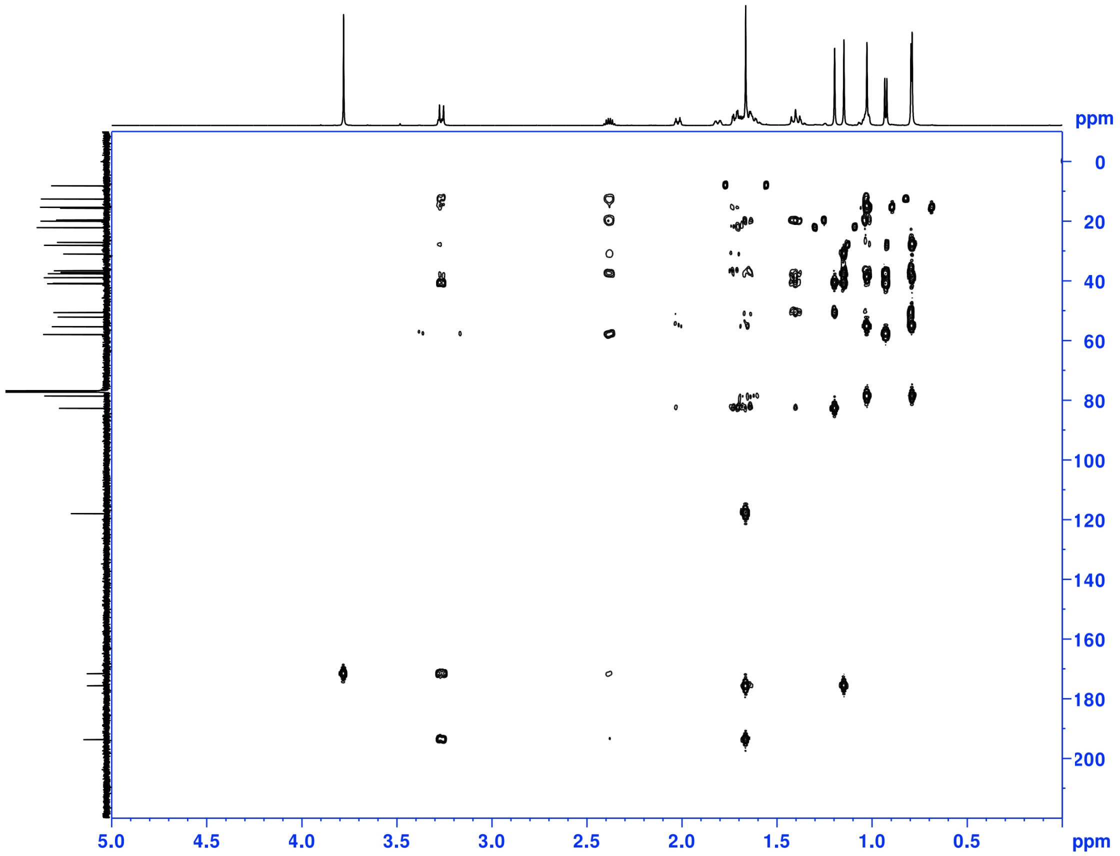

Figure S55. HMBC spectrum of insuetusin B3 (**8**) in $CDCl_3$.

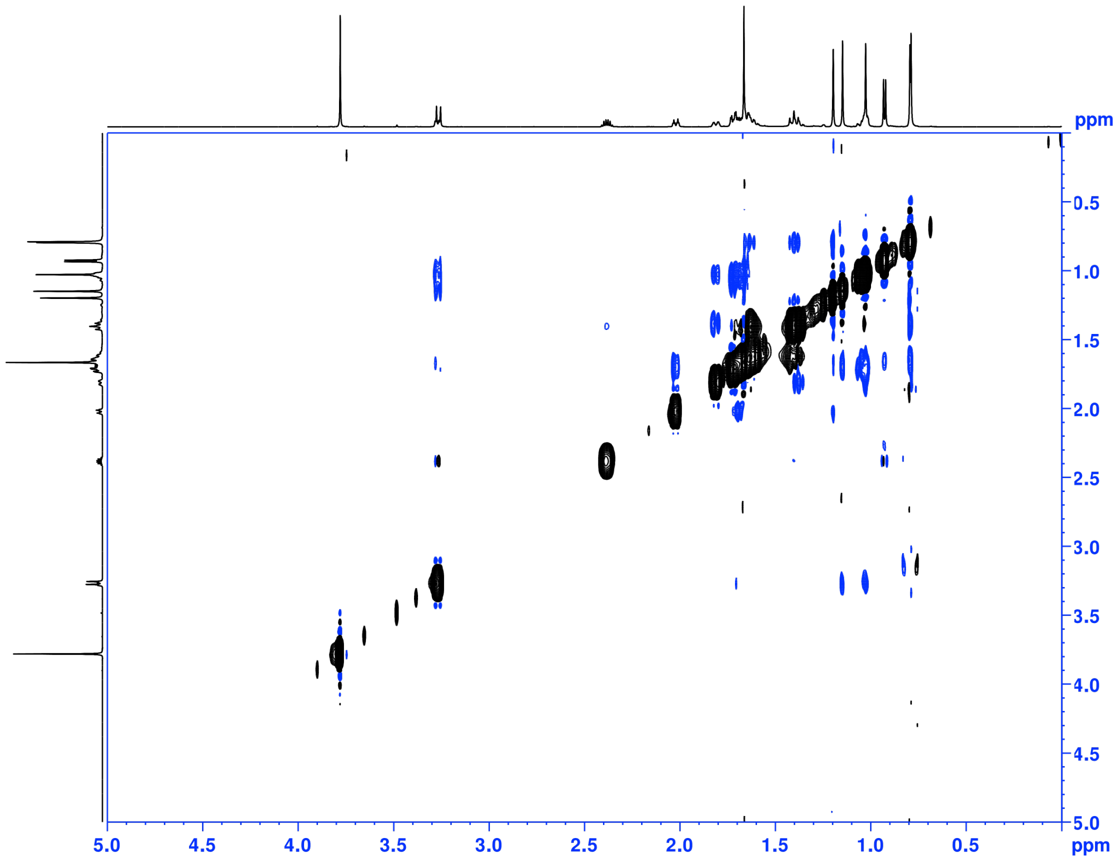

Figure S56. NOESY spectrum of insuetusin B3 (**8**) in $CDCl_3$.

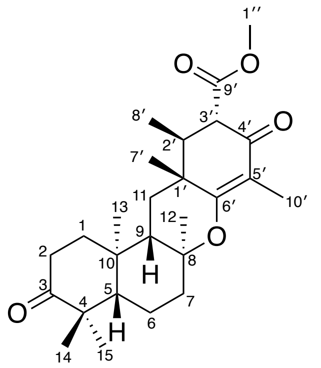

9

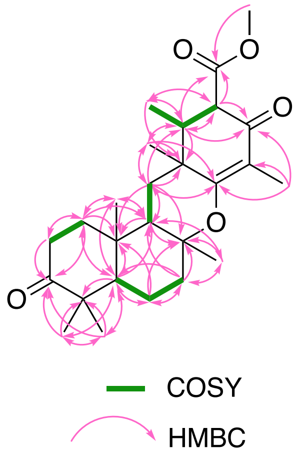

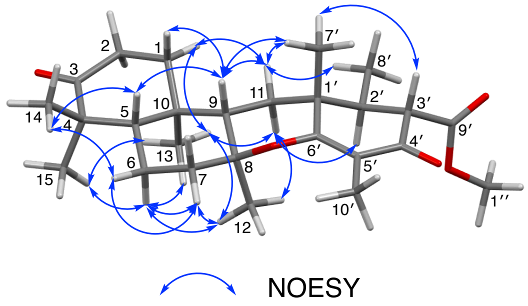

position	δ_C, type	δ_H, mult. (J in Hz)
1	37.6, CH_2	1.91 (α), ddd (13.2, 7.4, 4.4)
		1.51 (β), m
2	33.5, CH_2	2.53, m
3	216.4, C	
4	47.2, C	
5	54.5, CH	1.64, dd (12.0, 2.5)
6	20.8, CH_2	1.48 (α), m
		1.75 (β), m
7	40.0, CH_2	2.05 (α), dt (12.3, 3.2)
		1.74 (β), m
8	82.1, C	
9	50.0, CH	1.82, dd (13.3, 2.7)
10	36.3, C	
11	31.2, CH_2	1.46 (α), t (13.2)
		1.66 (β), m
12	21.7, CH_3	1.23, s
13	15.3, CH_3	0.89, s
14	26.8, CH_3	1.14, s
15	20.9, CH_3	1.04, s
1′	37.7, C	
2′	40.9, CH	2.40, dq (13.0, 6.7)
3′	57.9, CH	3.27, d (13.0)
4′	193.6, C	
5′	118.2, C	
6′	175.1, C	
7′	19.8, CH_3	1.16, s
8′	12.5, CH_3	0.94, d (6.7)
9′	171.5, C	
10′	8.1, CH_3	1.67, s
1′′	52.1, CH_3	3.78, s

^1H NMR: 600 MHz, ^{13}C NMR: 150 MHz (in $CDCl_3$)

Figure S57. NMR data of insuetusin B4 (**9**).

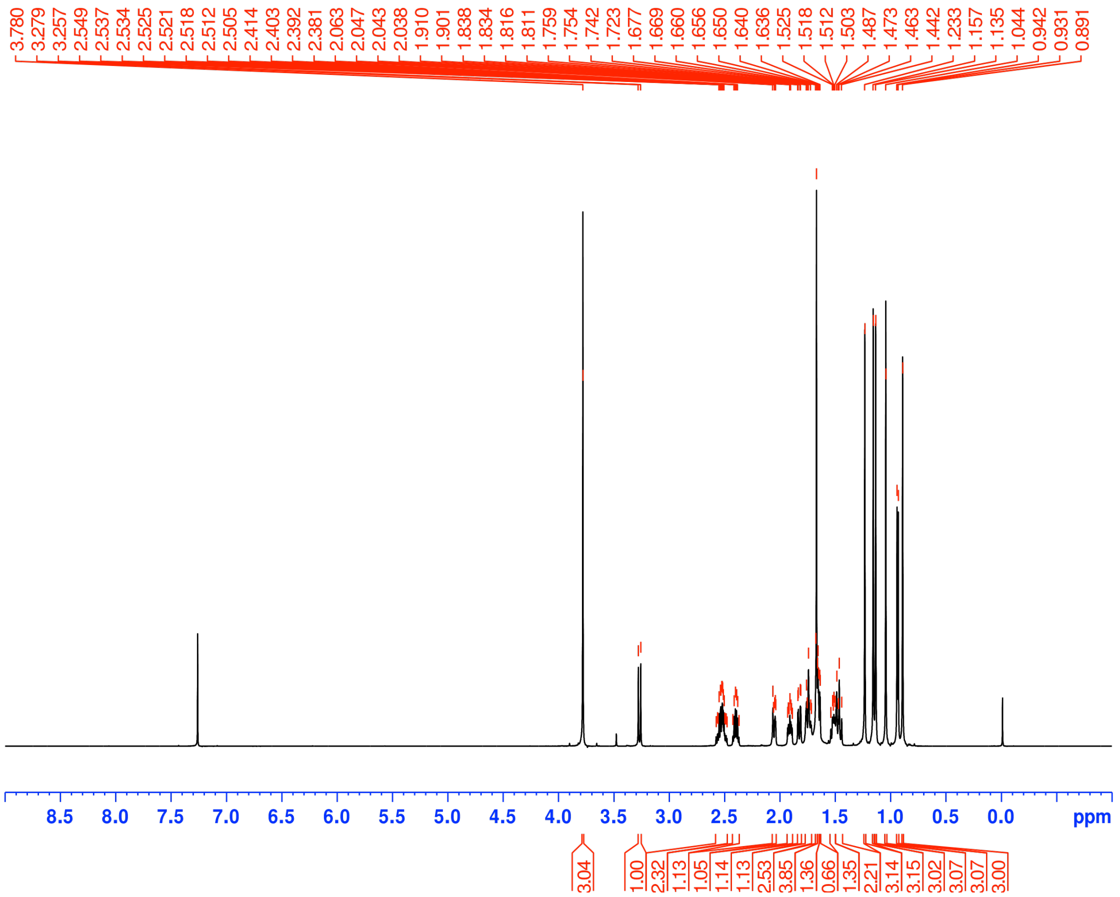

Figure S58. ^{1}H NMR spectrum of insuetusin B4 (**9**) in $CDCl_3$ at 600 MHz.

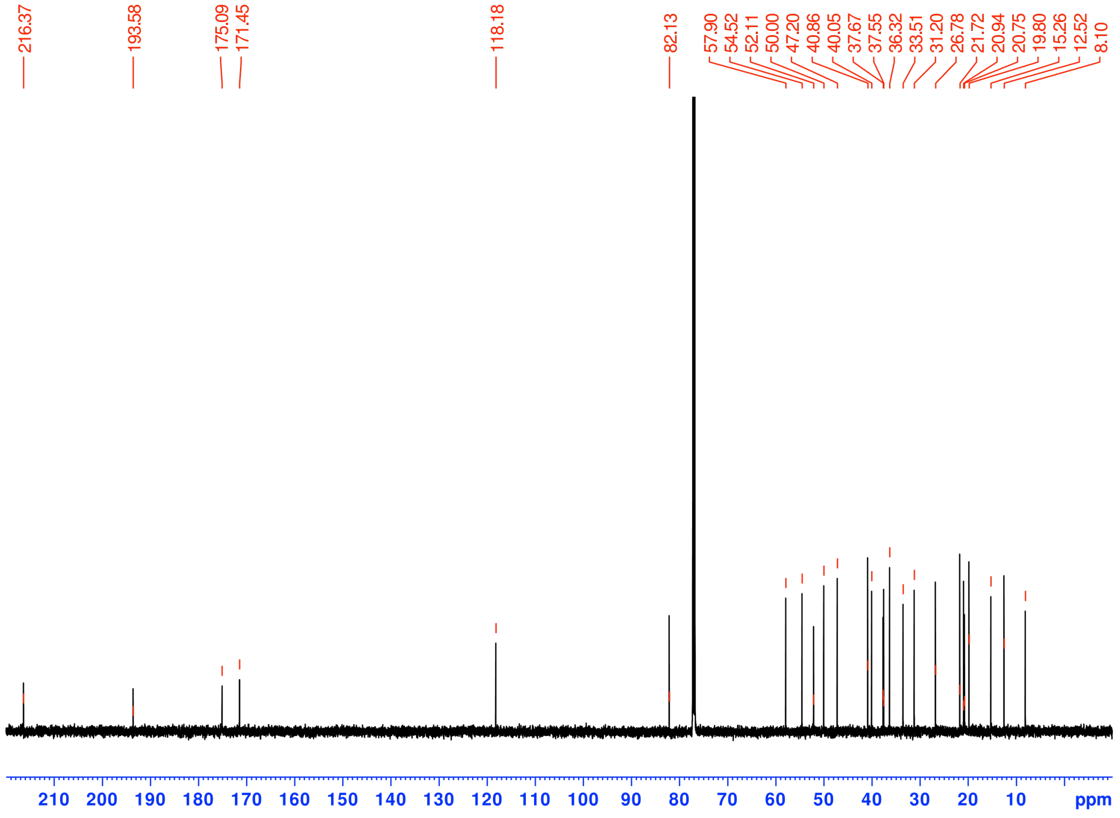

Figure S59. ^{13}C NMR spectrum of insuetusin B4 (**9**) in $CDCl_3$ at 150 MHz.

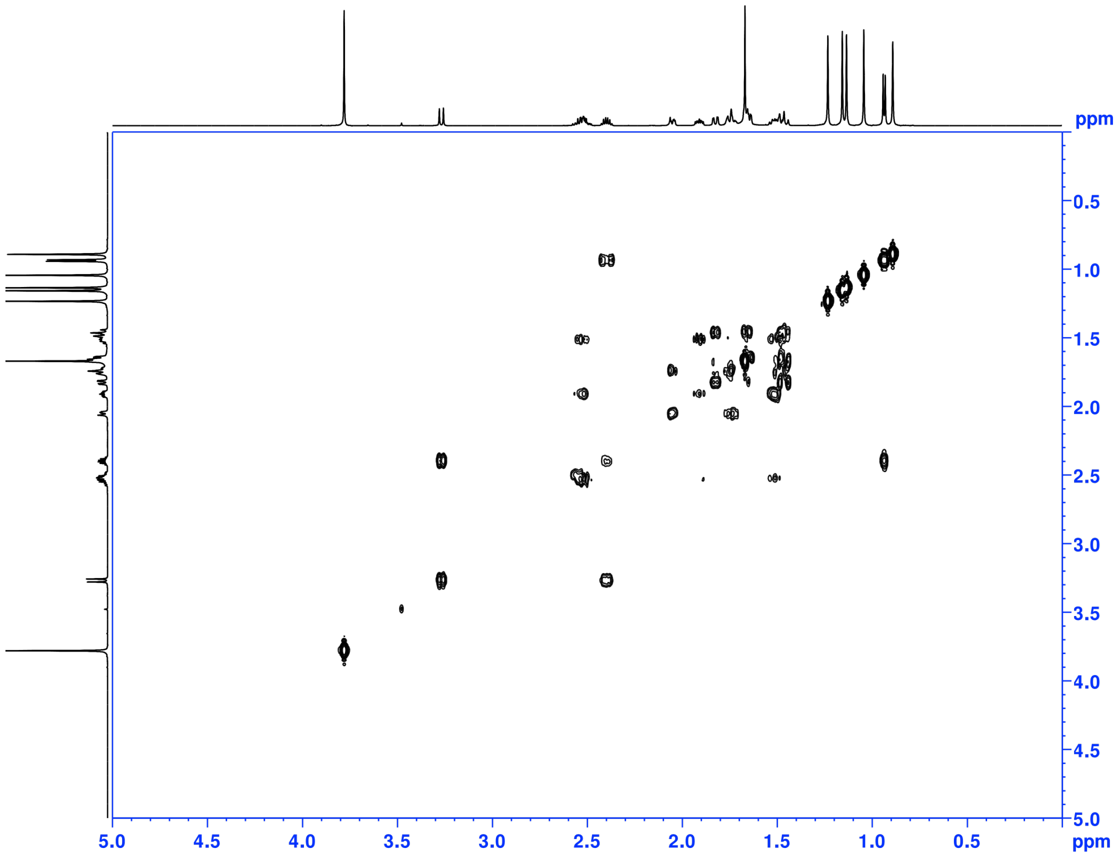

Figure S60. ^{1}H-^{1}H COSY spectrum of insuetusin B4 (**9**) in $CDCl_3$.

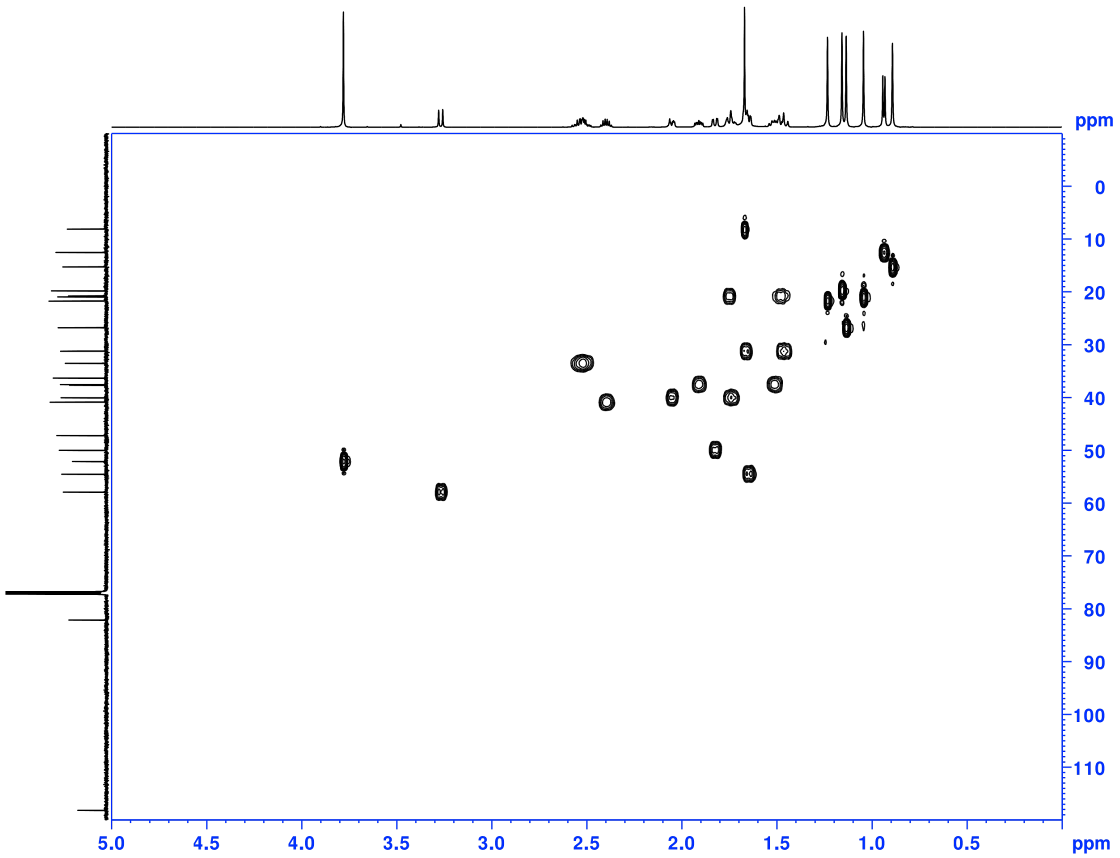

Figure S61. HSQC spectrum of insuetusin B4 (**9**) in $CDCl_3$.

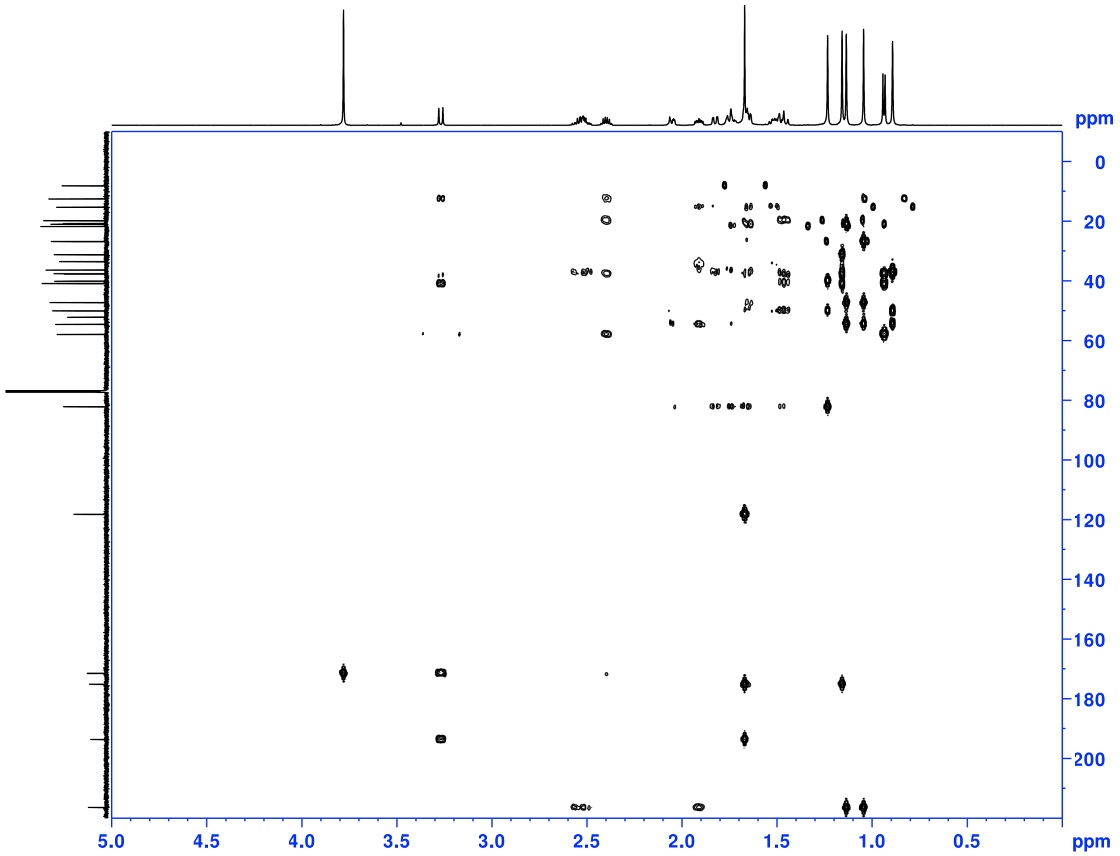

Figure S62. HMBC spectrum of insuetusin B4 (**9**) in $CDCl_3$.

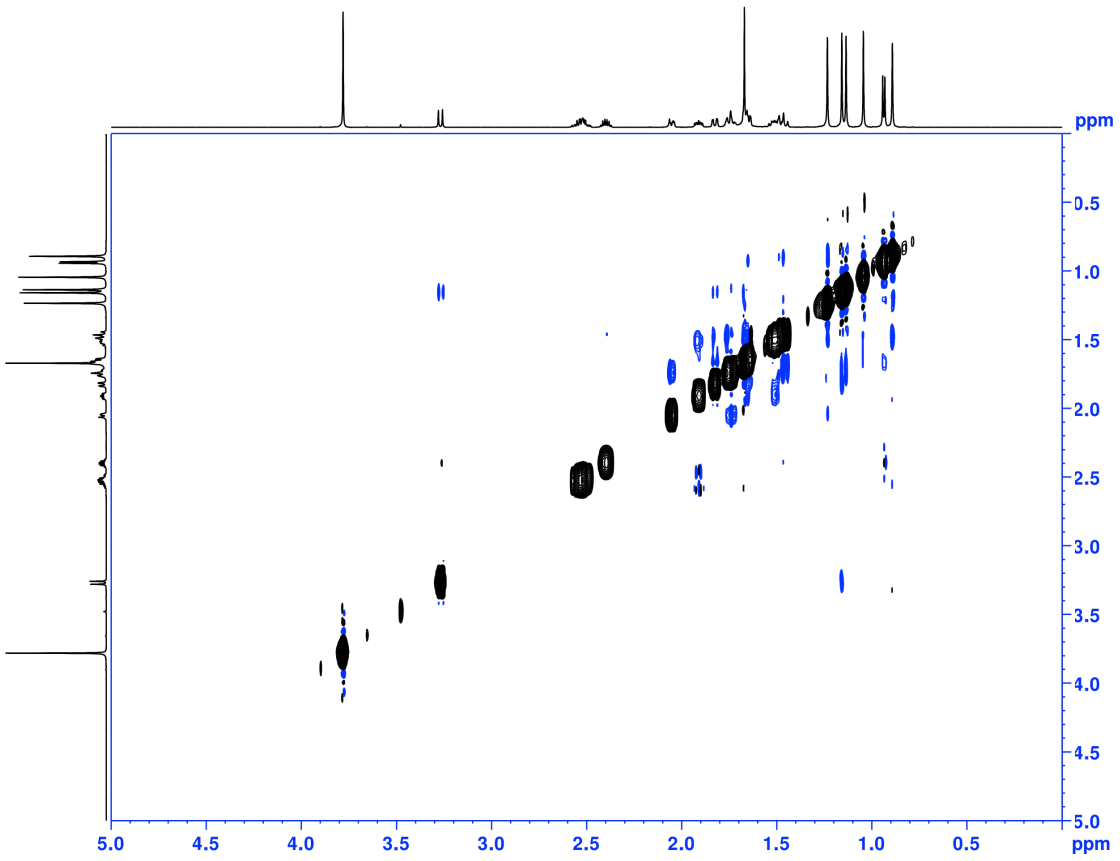

Figure S63. NOESY spectrum of insuetusin B4 (**9**) in $CDCl_3$.

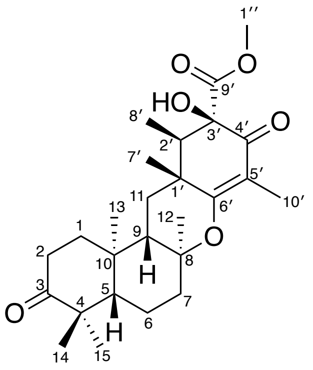

10

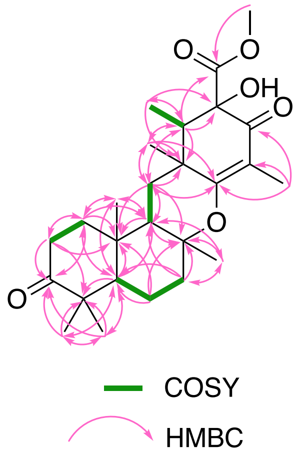

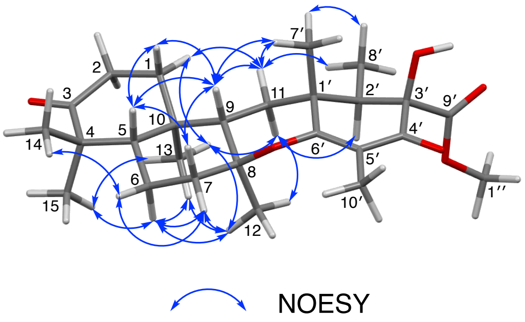

position	δC, type	δH, mult. (*J* in Hz)
1	37.5, CH_2	1.90 (α), m
		1.53 (β), m
2	33.5, CH_2	2.53, m
3	216.4, C	
4	47.2, C	
5	54.4, CH	1.67, dd (12.2, 2.8)
6	20.8, CH	1.48 (α), m
		1.75 (β), m
7	40.0, CH_2	2.07 (α), dt (13.4, 3.8)
		1.78 (β), m
8	82.7, C	
9	49.6, CH	1.88, dd (13.0, 2.9)
10	36.3, C	
11	31.8, CH_2	1.44 (α), t (13.1)
		1.66 (β), dd (13.0, 2.9)
12	21.9, CH_3	1.26, s
13	15.4, CH_3	0.89, s
14	26.9, CH_3	1.14, s
15	20.9, CH_3	1.04, s
1′	37.9, C	
2′	43.2, CH	2.47, q (7.0)
3′	80.0, C	
4′	194.1, C	
5′	116.8, C	
6′	176.8, C	
7′	22.9, CH_3	1.36, s
8′	8.2, CH_3	0.95, d (7.0)
9′	174.0, C	
10′	8.2, CH_3	1.72, s
1′′	53.4, CH_3	3.82, s

^1H NMR: 600 MHz, ^{13}C NMR: 150 MHz (in $CDCl_3$)

Figure S64. NMR data of insuetusin B5 (**10**).

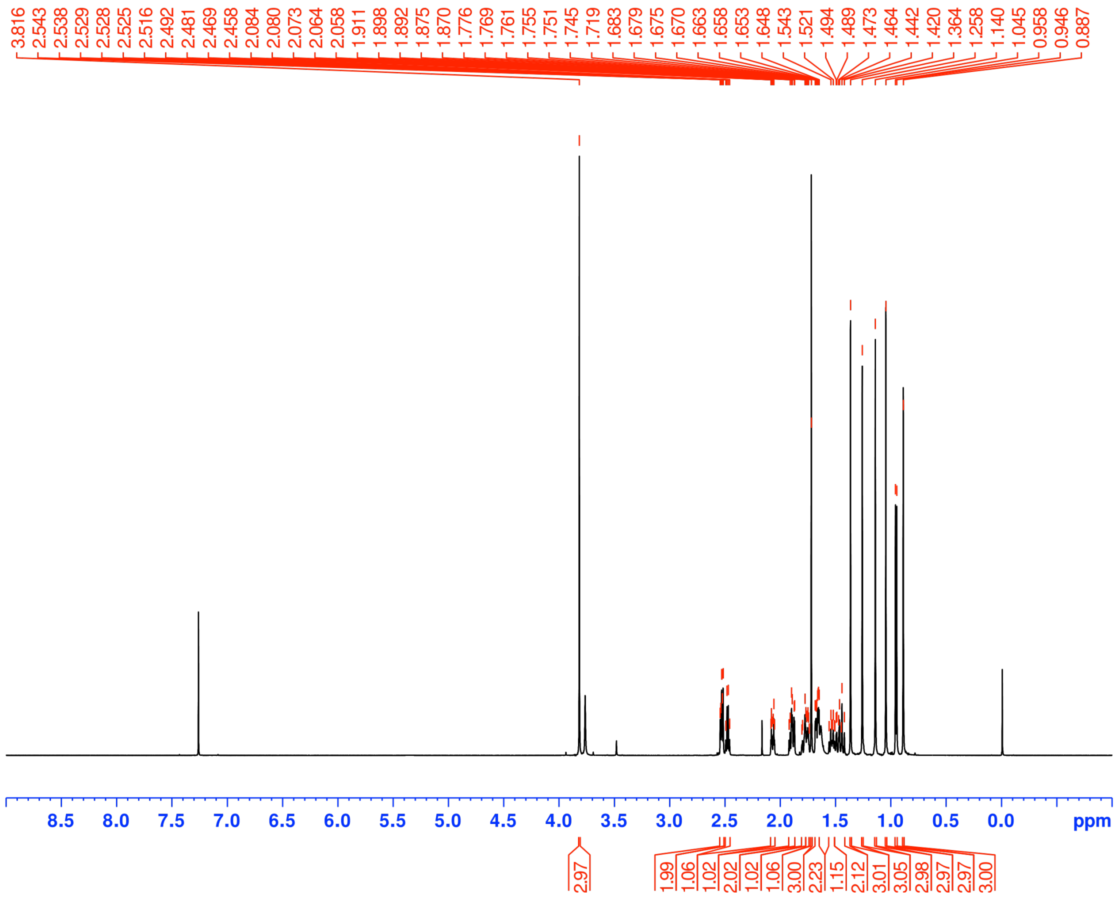

Figure S65. ^{1}H NMR spectrum of insuetusin B5 (**10**) in $CDCl_3$ at 600 MHz.

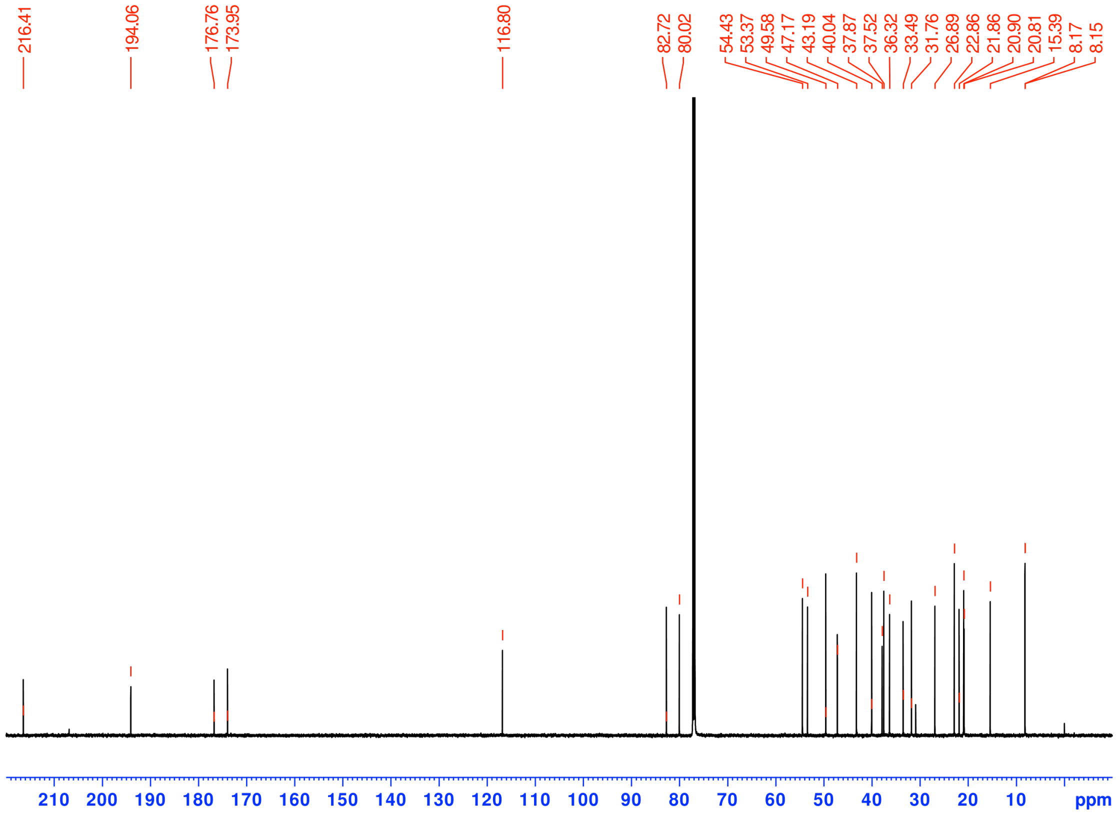

Figure S66. ^{13}C NMR spectrum of insuetusin B5 (**10**) in $CDCl_3$ at 150 MHz.

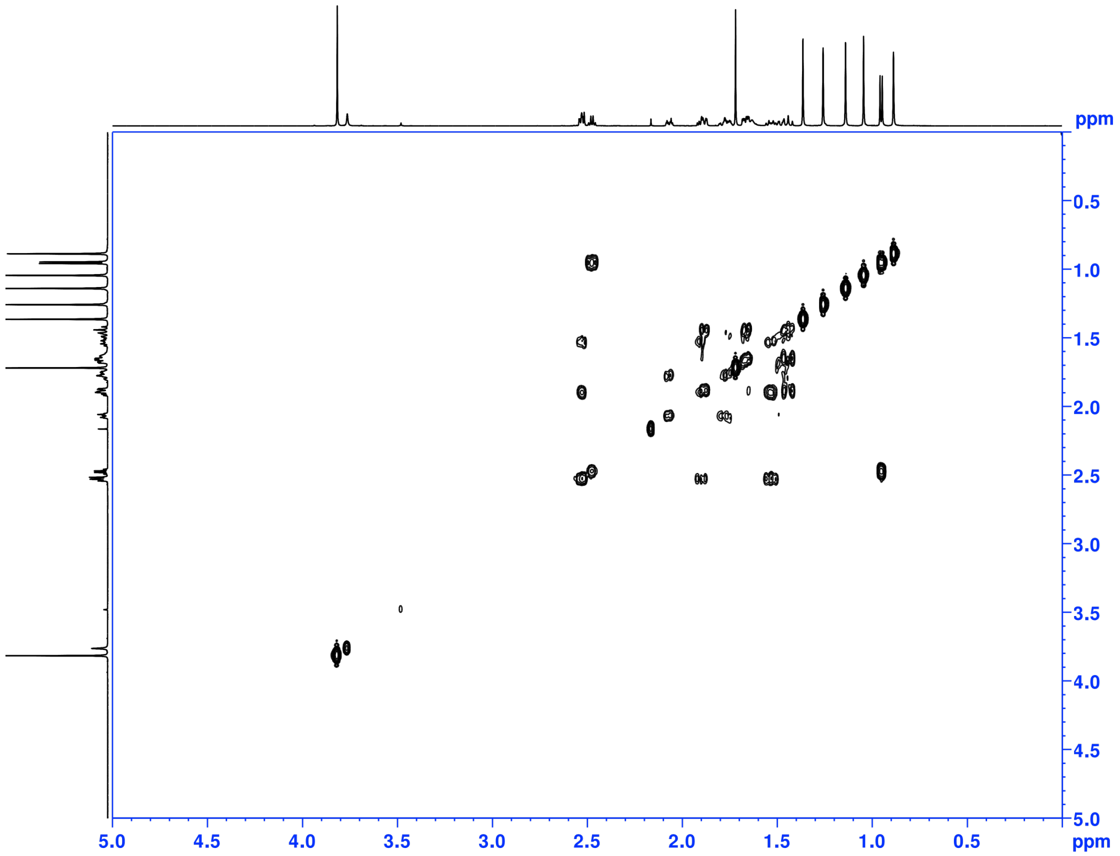

Figure S67. ^{1}H-^{1}H COSY spectrum of insuetusin B5 (**10**) in $CDCl_3$.

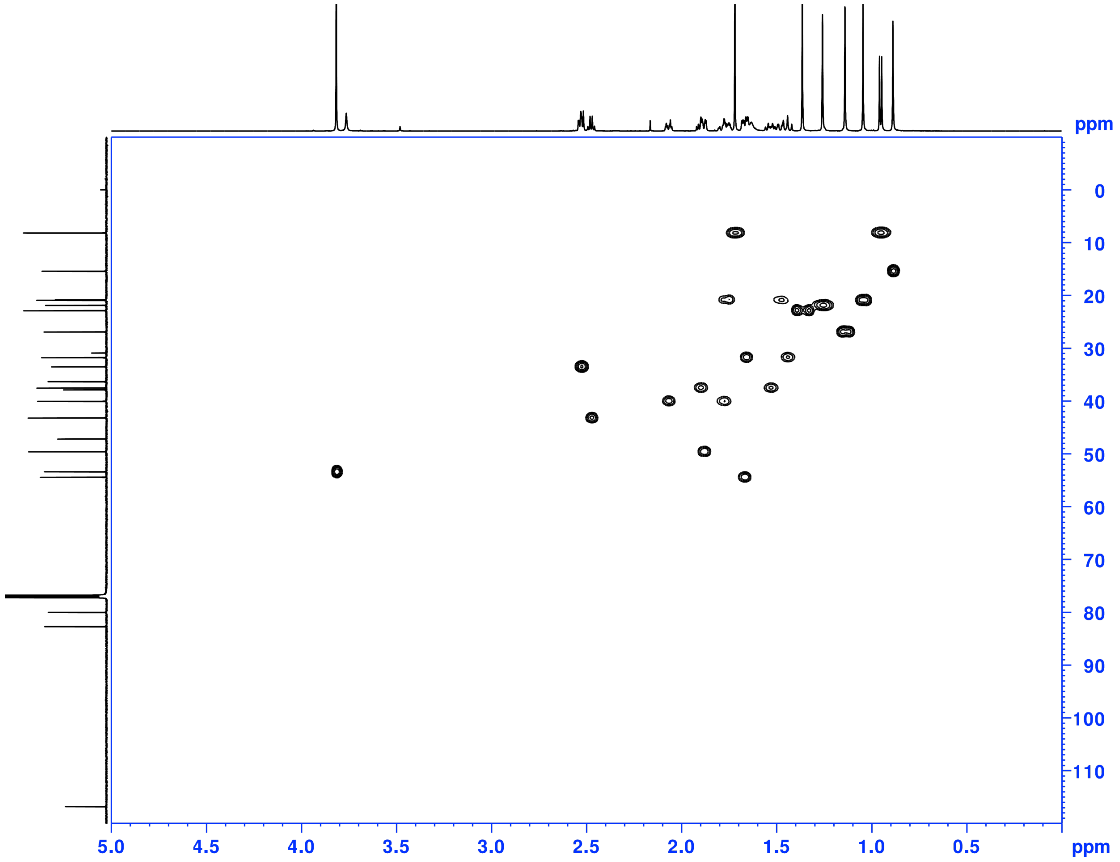

Figure S68. HSQC spectrum of insuetusin B5 (**10**) in $CDCl_3$.

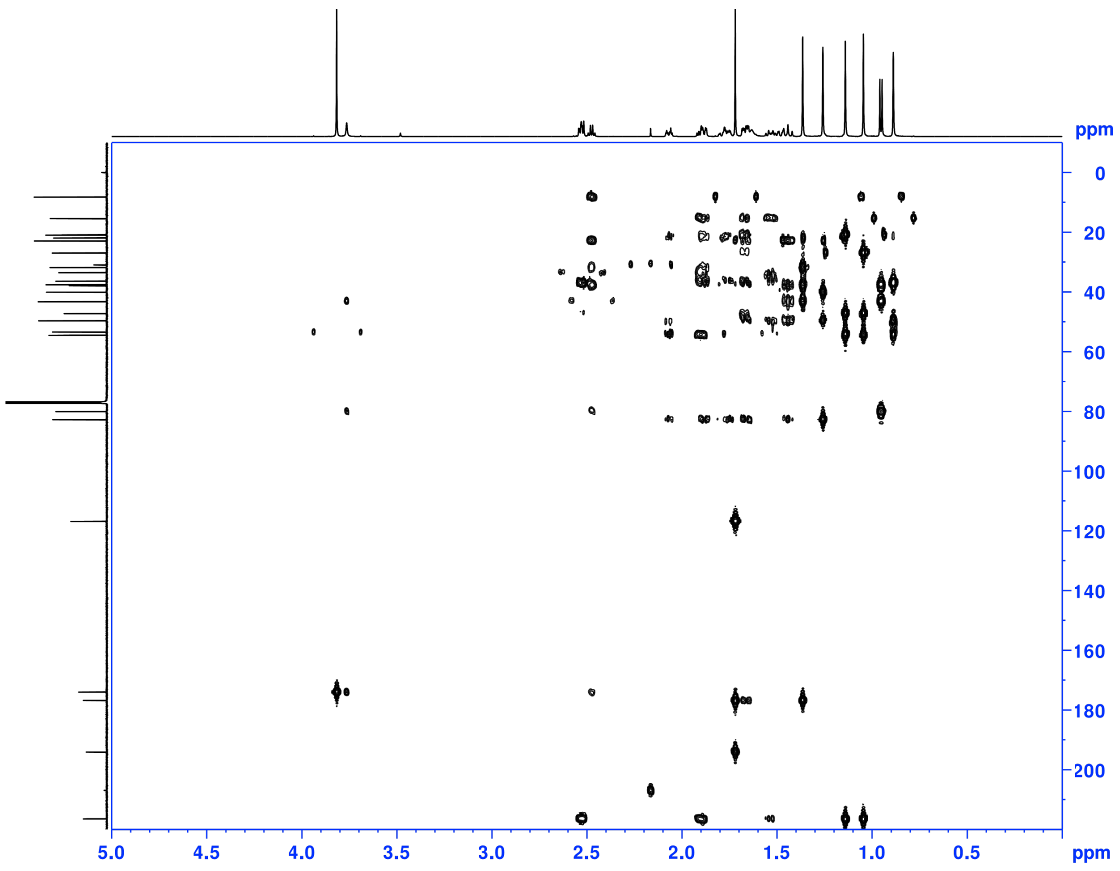

Figure S69. HMBC spectrum of insuetusin B5 (**10**) in $CDCl_3$.

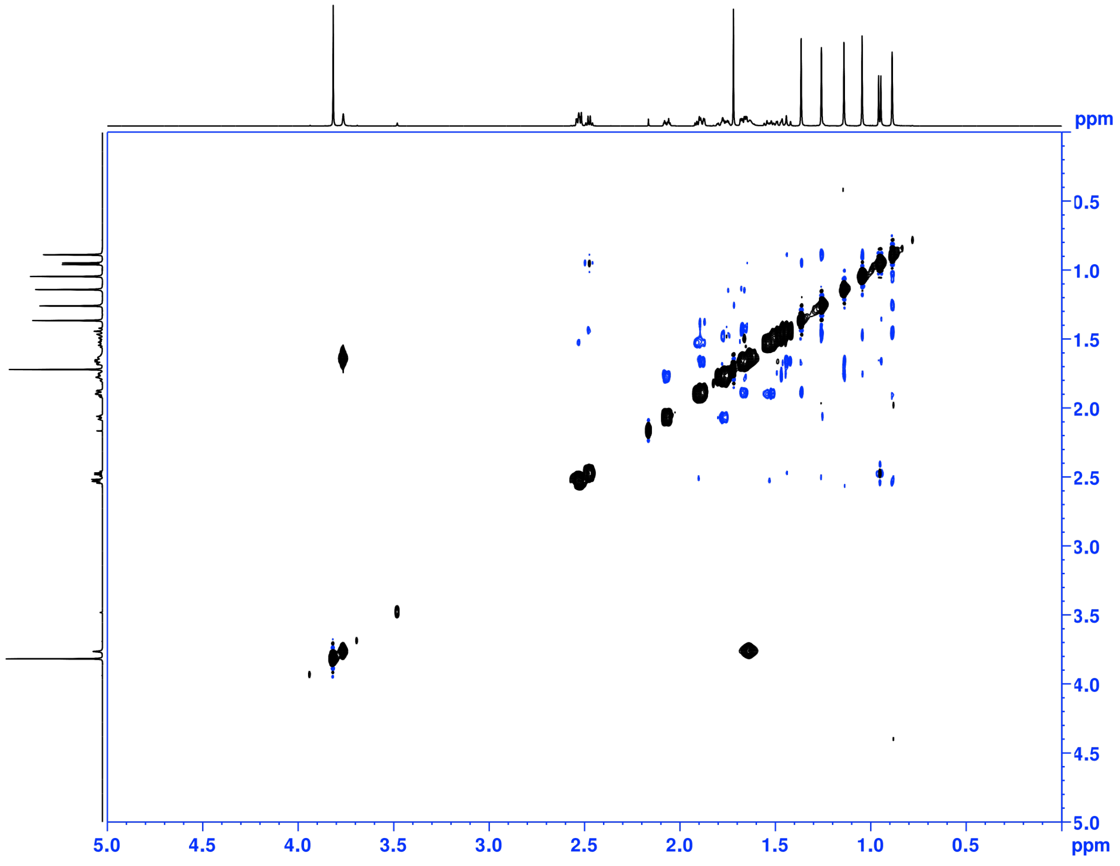

Figure S70. NOESY spectrum of insuetusin B5 (**10**) in $CDCl_3$.

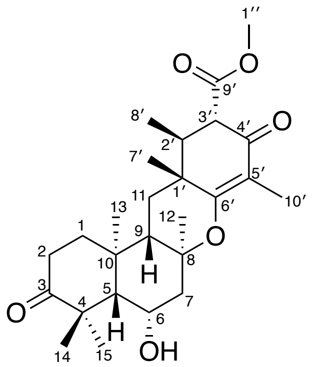

11

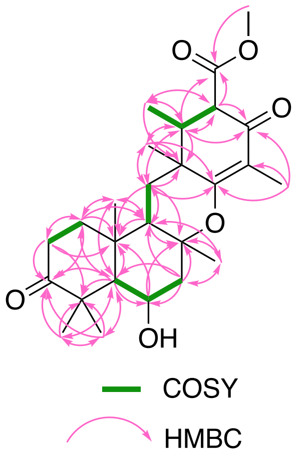

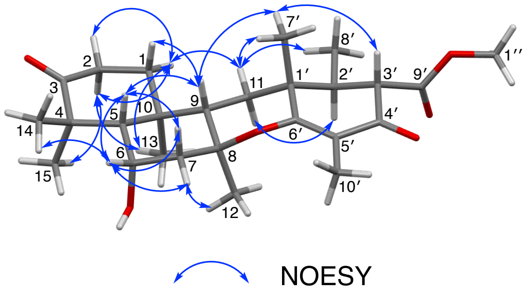

position	δ_C, type	δ_H, mult. (J in Hz)
1	40.1, CH_2	1.95 (α), m
		1.38 (β), m
2	33.9, CH_2	2.83 (α), td (14.6, 6.1)
		2.32 (β), brd (13.4)
3	215.1, C	
4	48.9, C	
5	56.7, CH	1.42, overlapped
6	68.7, CH	4.60, brs
7	48.4, CH_2	2.20 (α), dd (13.8, 2.1)
		1.95 (β), m
8	81.8, C	
9	50.8, CH	1.83, dd (12.9, 1.6)
10	36.7, C	
11	31.1, CH_2	1.60 (α), t (13.1)
		1.72 (β), dd (12.8, 1.8)
12	23.5, CH_3	1.50, s
13	16.2, CH_3	1.38, s
14	25.0, CH_3	1.21, s
15	23.6, CH_3	1.42, s
1′	37.7, C	
2′	40.8, CH	2.43, dq (13.2, 6.6)
3′	57.9, CH	3.27, d (13.0)
4′	193.6, C	
5′	118.5, C	
6′	174.9, C	
7′	19.9, CH_3	1.15, s
8′	12.5, CH_3	0.95, d (6.6)
9′	171.5, C	
10′	8.1, CH_3	1.67, s
1′′	52.1, CH_3	3.79, s

^{1}H NMR: 600 MHz, ^{13}C NMR: 150 MHz (in $CDCl_3$)

Figure S71. NMR data of insuetusin B6 (**11**).

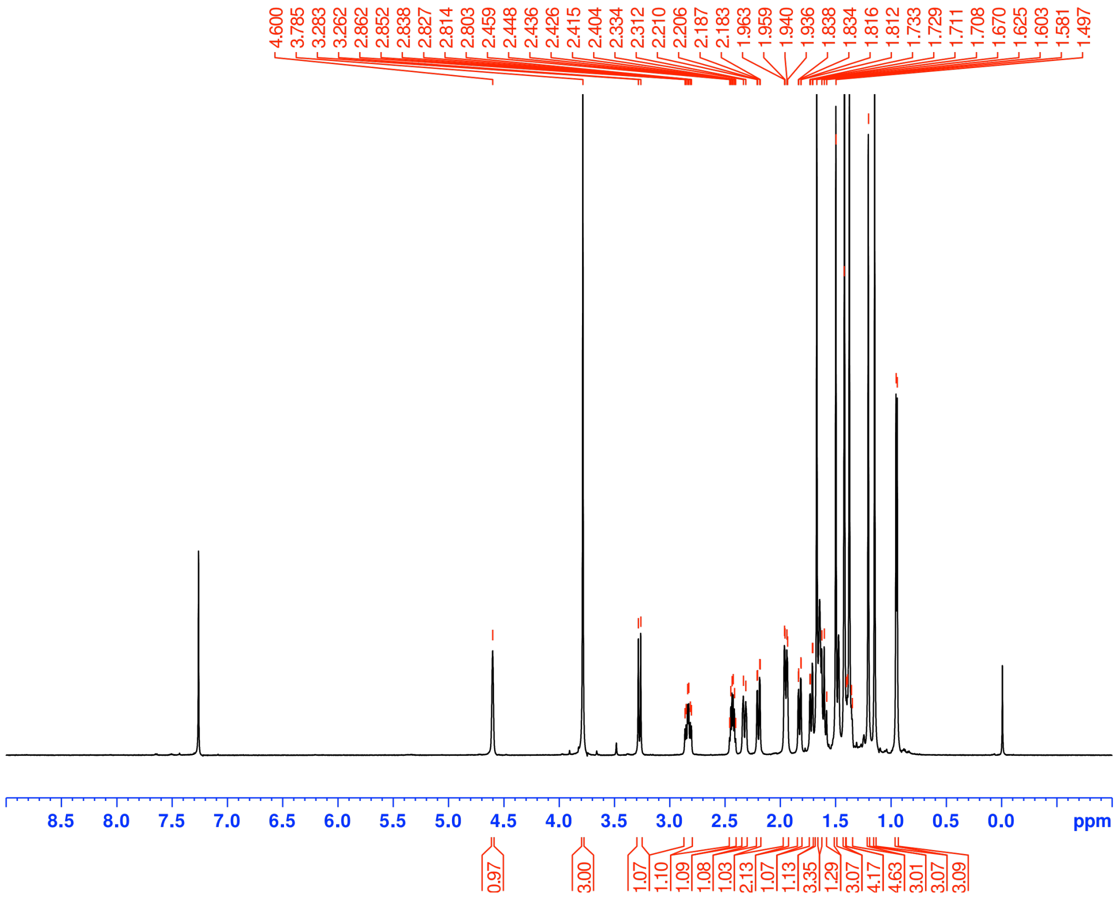

Figure S72. ^{1}H NMR spectrum of insuetusin B6 (**11**) in $CDCl_3$ at 600 MHz.

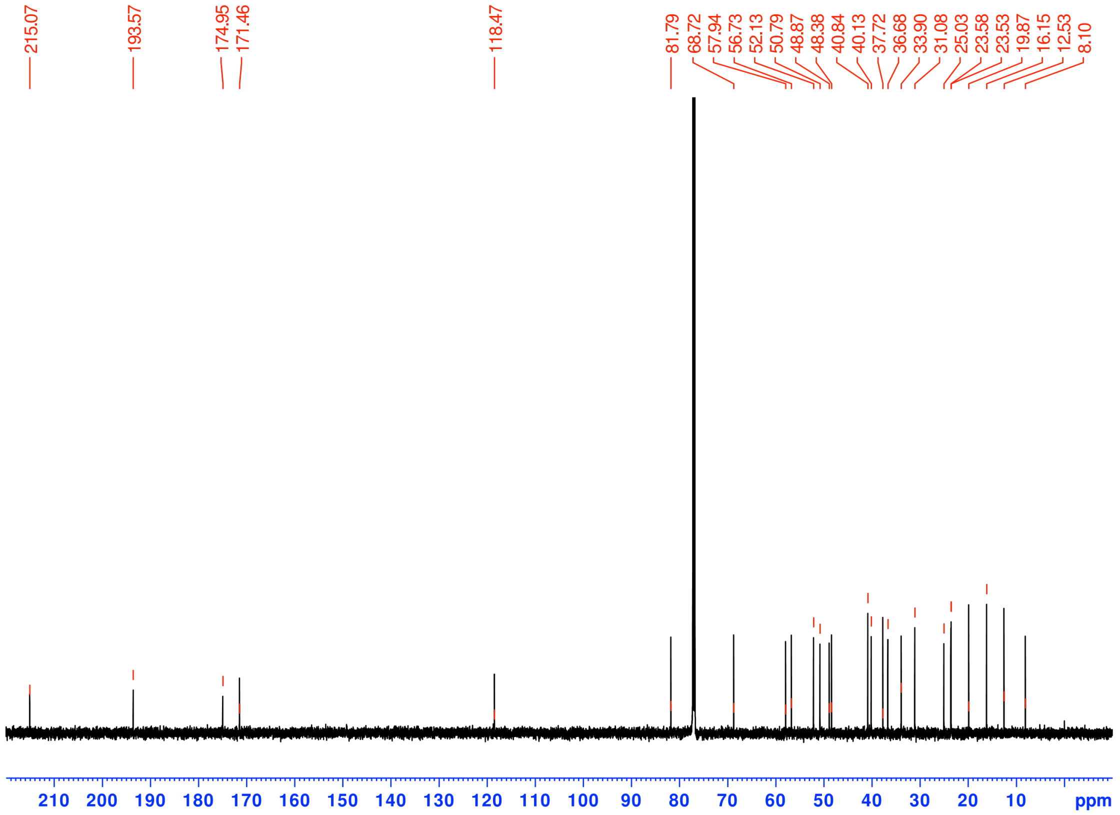

Figure S73. ^{13}C NMR spectrum of insuetusin B6 (**11**) in $CDCl_3$ at 150 MHz.

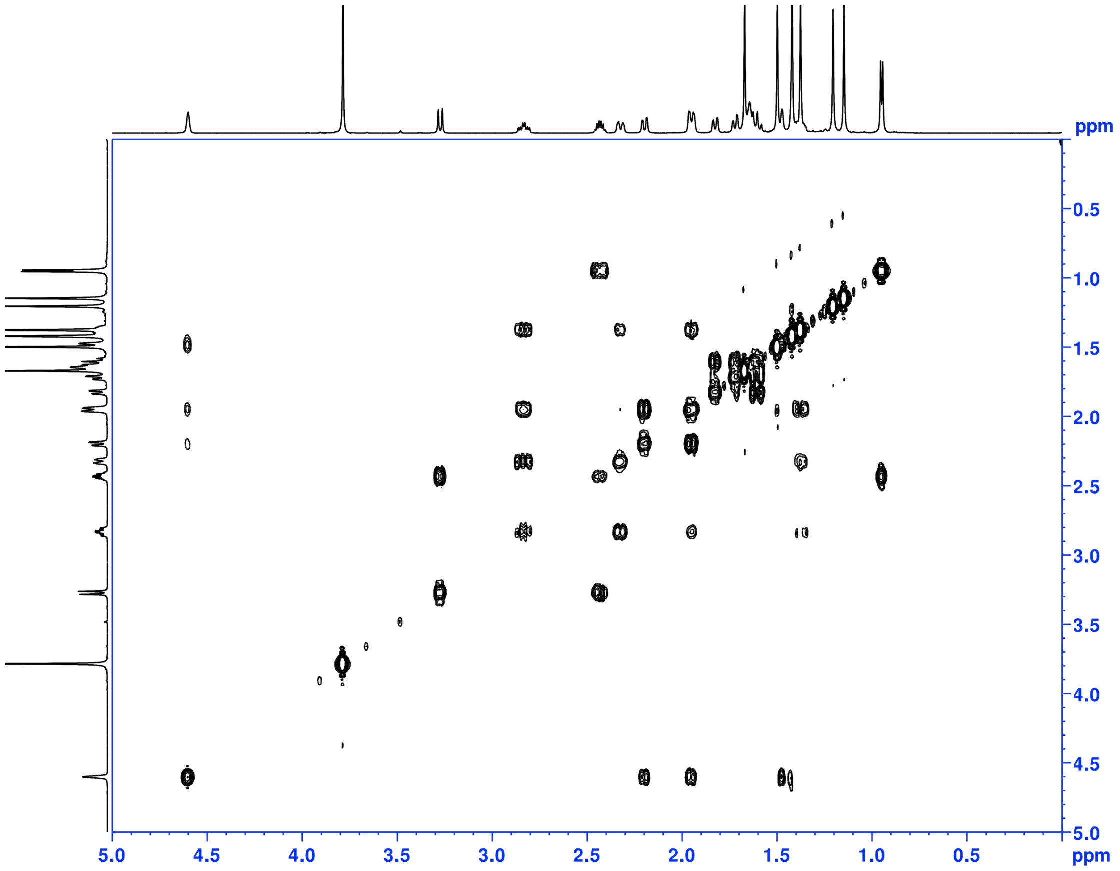

Figure S74. ^{1}H-^{1}H COSY spectrum of insuetusin B6 (**11**) in $CDCl_3$.

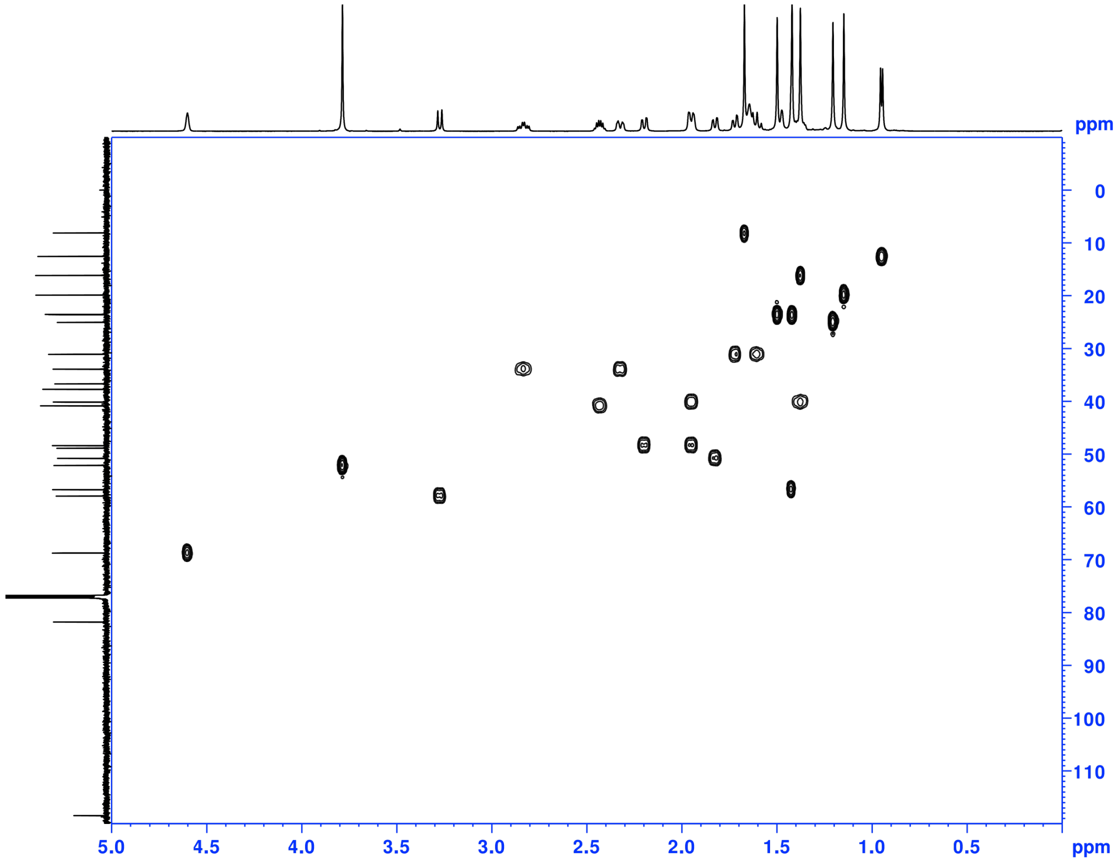

Figure S75. HSQC spectrum of insuetusin B6 (**11**) in $CDCl_3$.

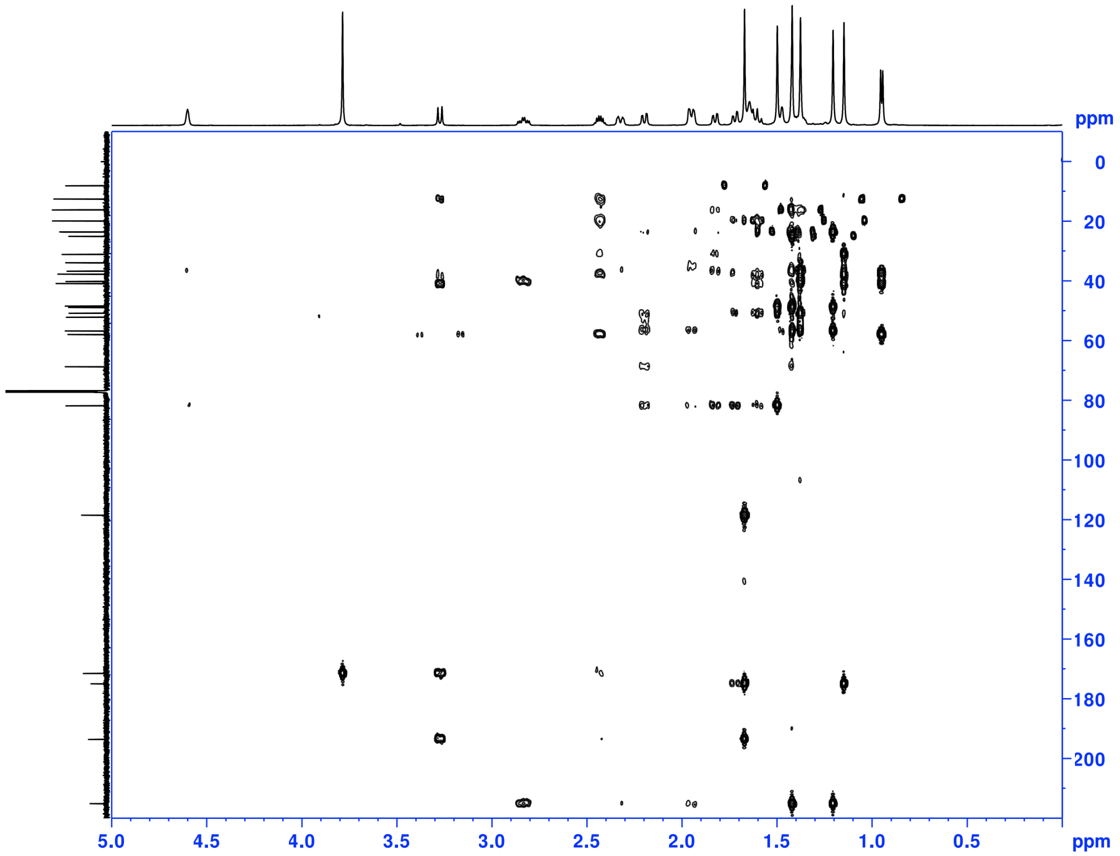

Figure S76. HMBC spectrum of insuetusin B6 (**11**) in $CDCl_3$.

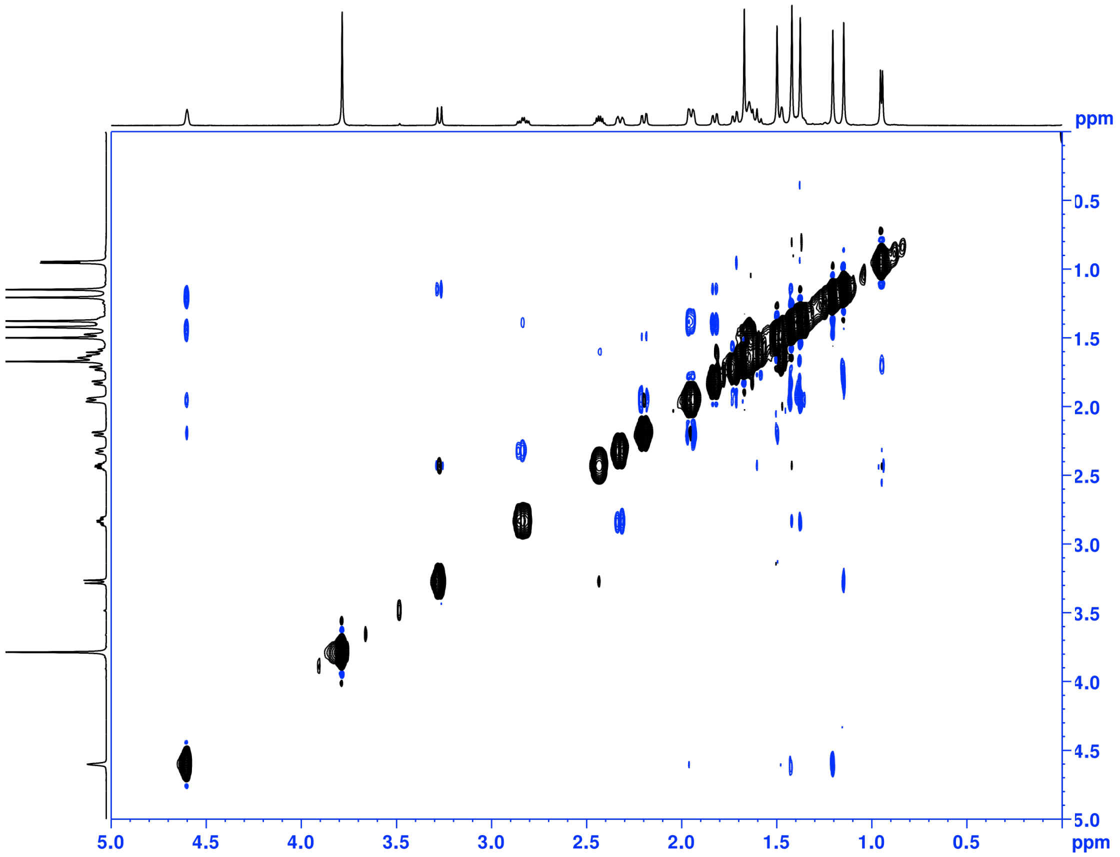

Figure S77. NOESY spectrum of insuetusin B6 (**11**) in $CDCl_3$.

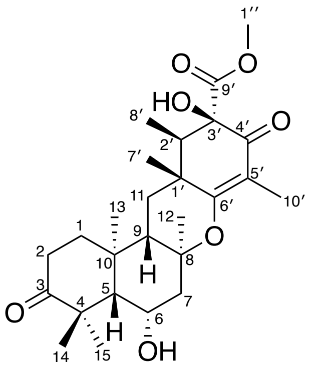

12

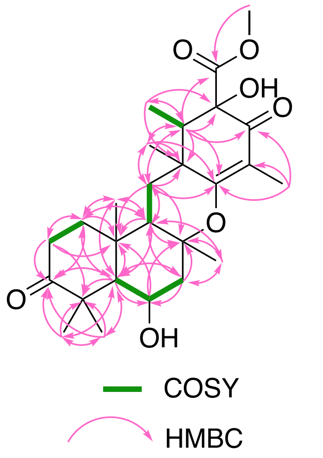

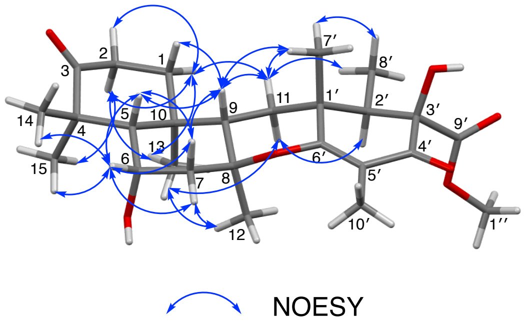

position	δ_C, type	δ_H, mult. (J in Hz)
1	40.1, CH_2	1.94 (α), ddd (13.0, 6.5, 2.7)
		1.39 (β), m
2	33.9, CH_2	2.82 (α), ddd (15.3, 13.9, 6.5)
		2.33 (β), ddd (15.3, 4.9, 2.7)
3	215.0, C	
4	48.8, C	
5	56.7, CH	1.44, d (2.1)
6	68.8, CH	4.61, q (2.2)
7	48.4, CH_2	2.21 (α), dd (14.0, 3.0)
		1.99 (β), dd (14.0, 3.0)
8	82.4, C	
9	50.4, CH	1.88, dd (13.1, 2.8)
10	36.7, C	
11	31.6, CH_2	1.58 (α), t (13.1)
		1.72 (β), dd (12.8, 3.0)
12	23.7, CH_3	1.52, s
13	16.3, CH_3	1.37, s
14	25.1, CH_3	1.21, s
15	23.6, CH_3	1.42, s
1′	37.9, C	
2′	43.2, CH	2.51, q (7.0)
3′	80.1, CH	3.27, d (13.0)
4′	194.0, C	
5′	117.1, C	
6′	176.6, C	
7′	23.0, CH_3	1.35, s
8′	8.2, CH_3	0.96, d (7.0)
9′	174.0, C	
10′	8.2, CH_3	1.72, s
1′′	53.4, CH_3	3.82, s

^{1}H NMR: 600 MHz, ^{13}C NMR: 150 MHz (in $CDCl_3$)

Figure S78. NMR data of insuetusin B7 (**12**).

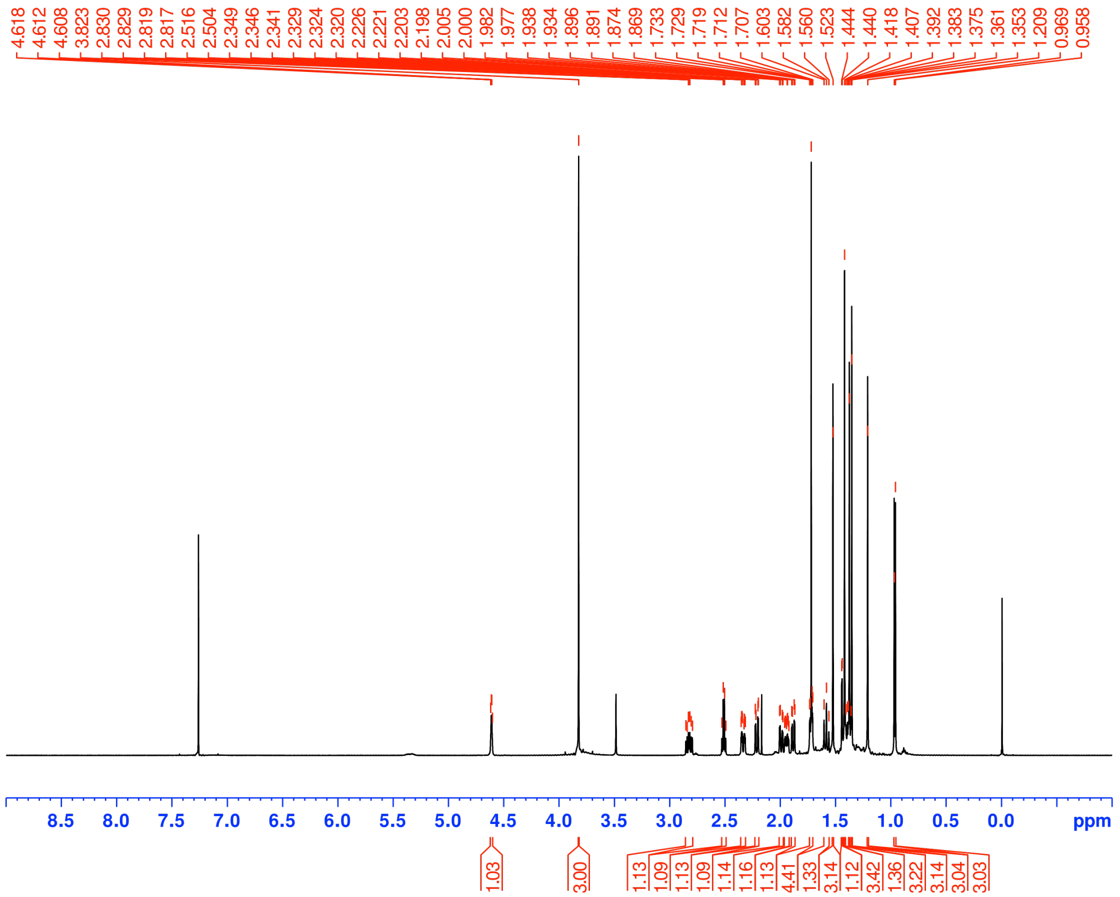

Figure S79. ^{1}H NMR spectrum of insuetusin B7 (**12**) in $CDCl_3$ at 600 MHz.

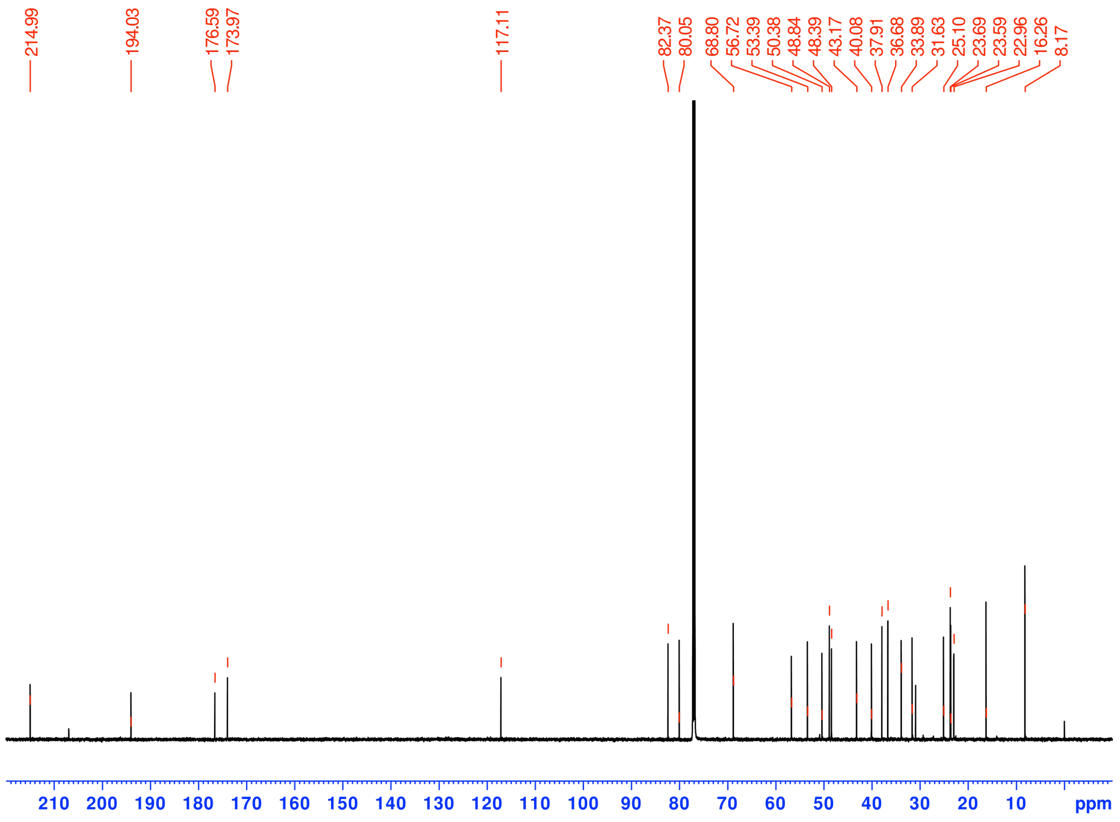

Figure S80. ^{13}C NMR spectrum of insuetusin B7 (**12**) in $CDCl_3$ at 150 MHz.

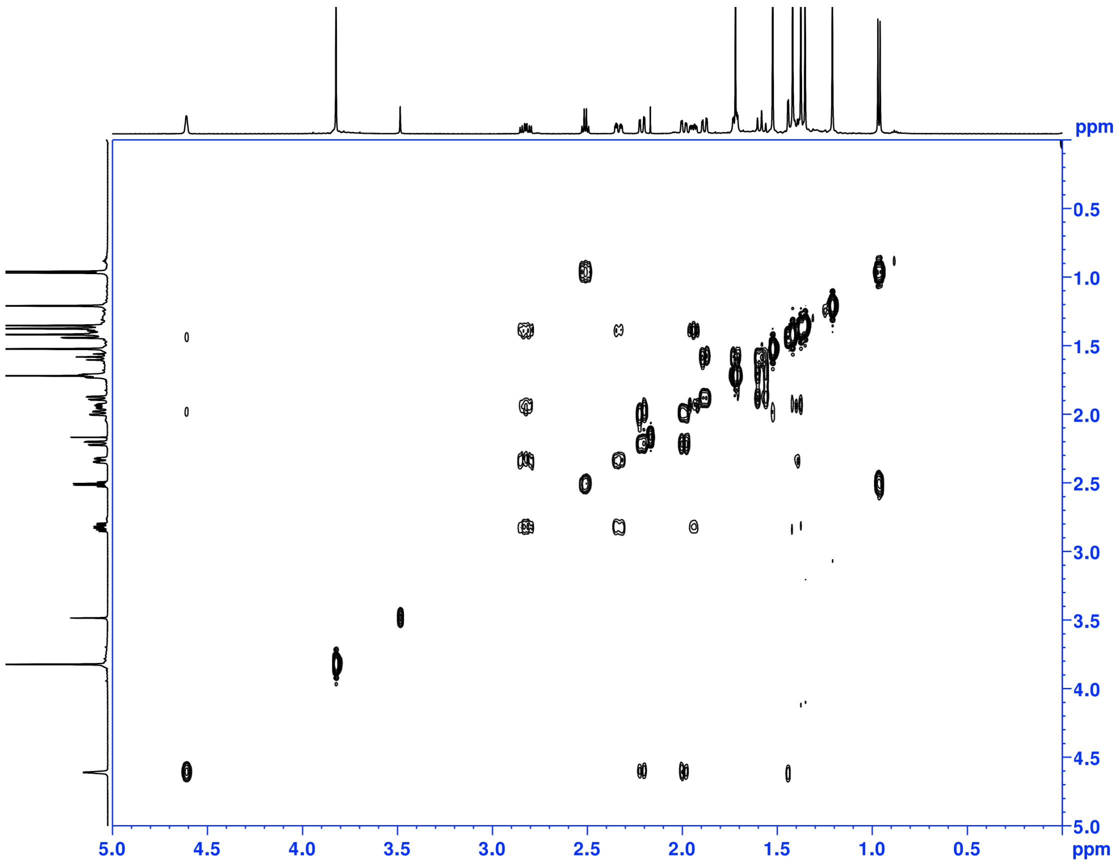

Figure S81. ^{1}H-^{1}H COSY spectrum of insuetusin B7 (**12**) in $CDCl_3$.

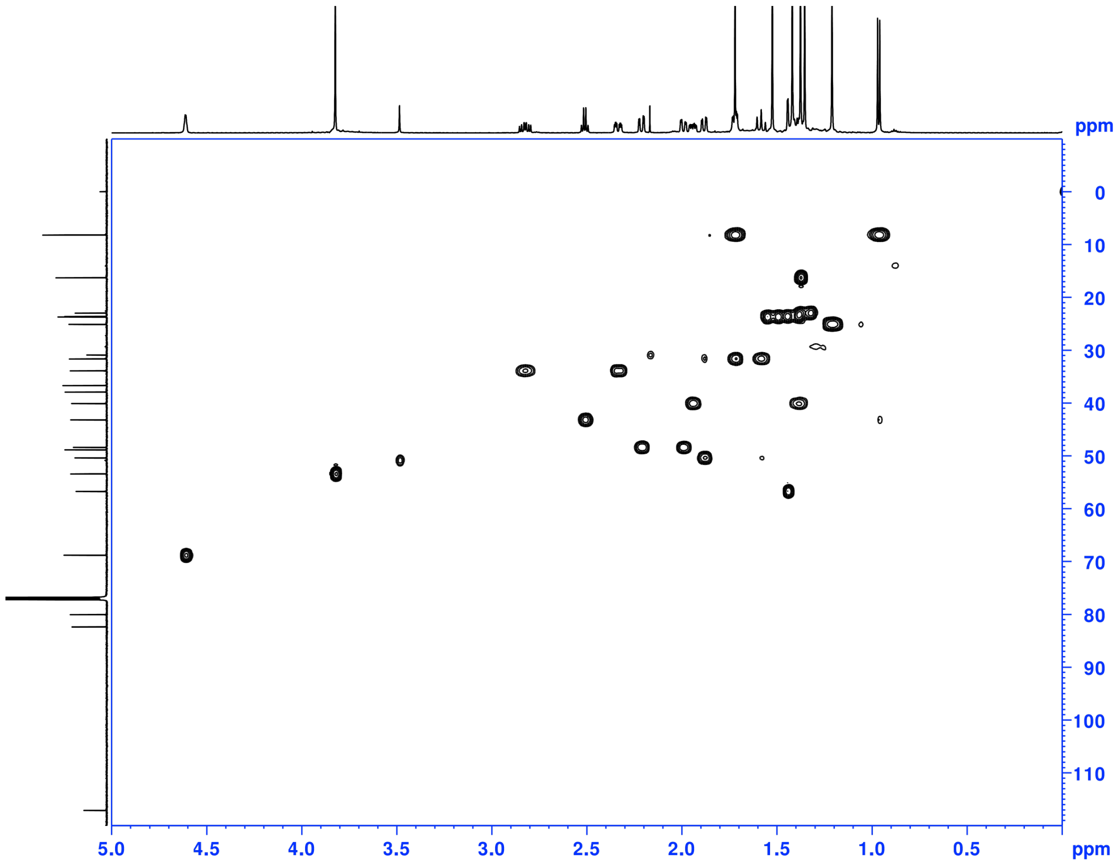

Figure S82. HSQC spectrum of insuetusin B7 (**12**) in $CDCl_3$.

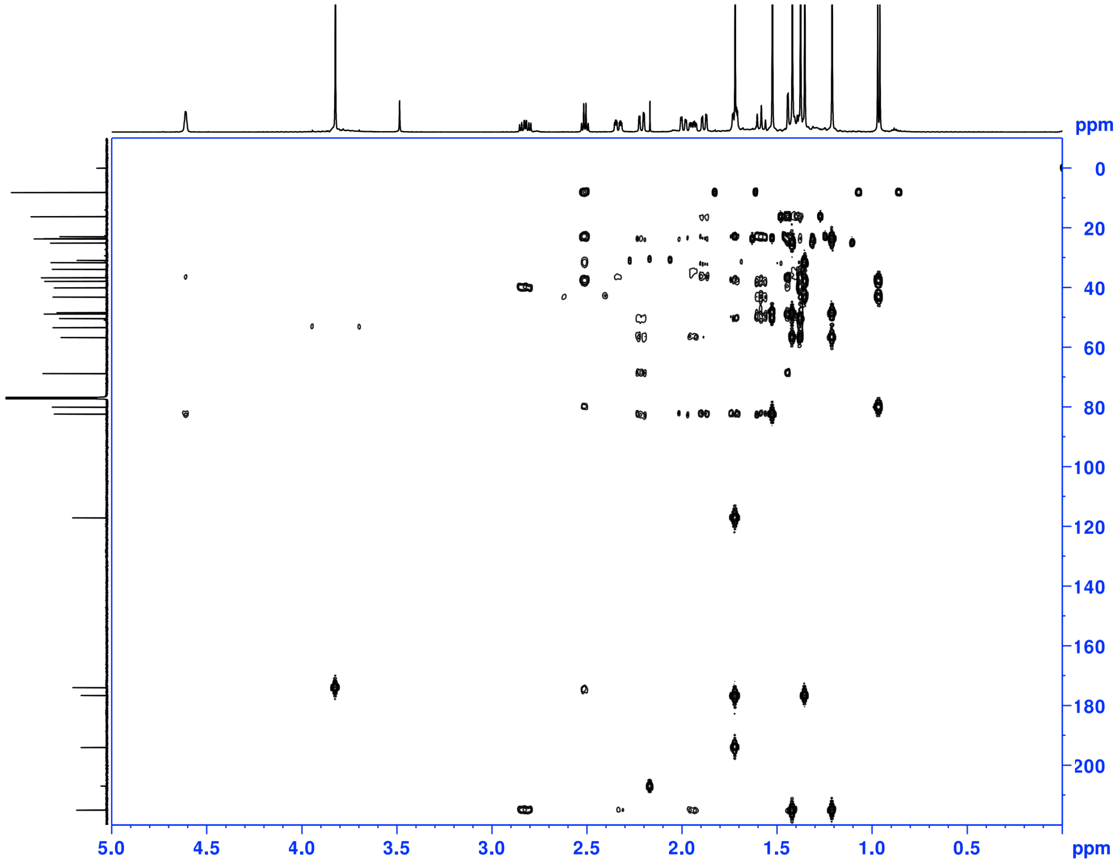

Figure S83. HMBC spectrum of insuetusin B7 (**12**) in $CDCl_3$.

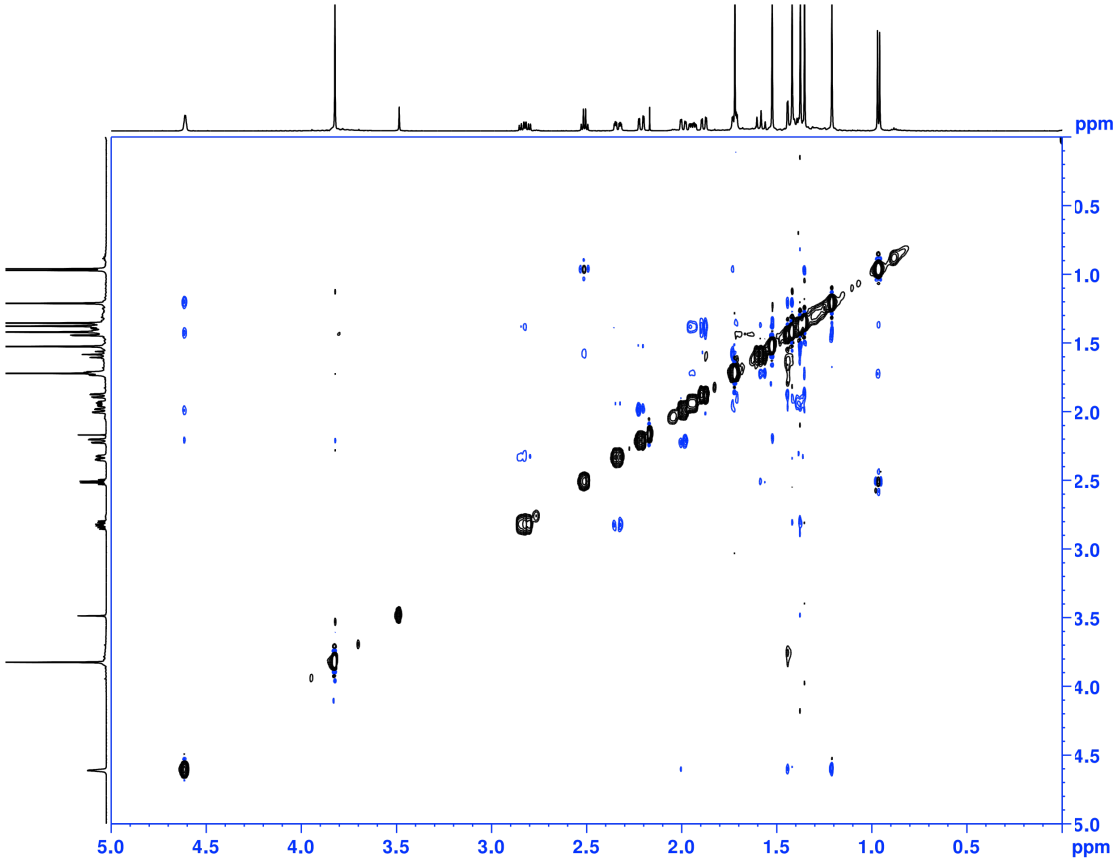

Figure S84. NOESY spectrum of insuetusin B7 (**12**) in $CDCl_3$.

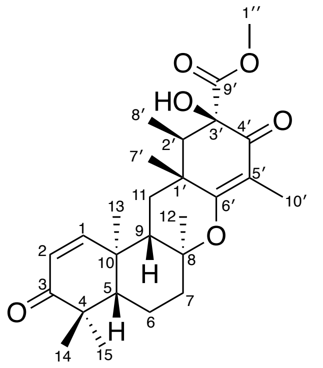

13

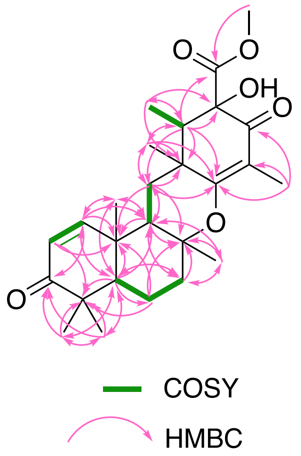

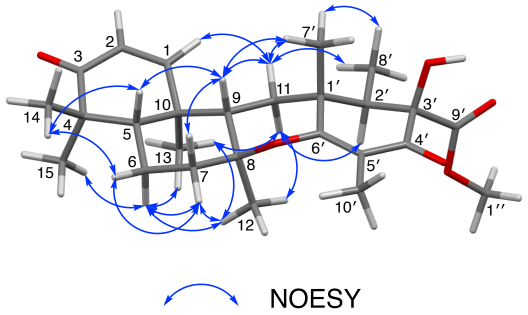

position	δ_C, type	δ_H, mult. (*J* in Hz)
1	155.8, CH	7.08, d (10.1)
2	126.6, CH	5.93, d (10.1)
3	204.4, C	
4	44.7, C	
5	53.4, CH	1.91, dd (13.0, 1.9)
6	20.2, CH_2	1.53 (α), m
		1.84 (β), m
7	40.1, CH_2	2.11 (α), dt (13.8, 4.5)
		1.82 (β), m
8	82.8, C	
9	45.0, CH	2.07, dd (13.3, 2.5)
10	39.0, C	
11	31.5, CH_2	1.55 (α), t (13.4)
		1.88 (β), dd (13.4, 2.4)
12	22.5, CH_3	1.30, s
13	18.9, CH_3	1.04, s
14	27.9, CH_3	1.20, s
15	21.3, CH_3	1.11, s
1′	38.1, C	
2′	43.2, CH	2.52, q (6.9)
3′	80.0, C	
4′	194.0, C	
5′	117.1, C	
6′	176.3, C	
7′	22.9, CH_3	1.40, s
8′	8.2, CH_3	1.01, d (6.9)
9′	173.9, C	
10′	8.2, CH_3	1.73, s
1′′	53.4, CH_3	3.83, s

^{1}H NMR: 600 MHz, ^{13}C NMR: 150 MHz (in $CDCl_3$)

Figure S85. NMR data of insuetusin B8 (**13**).

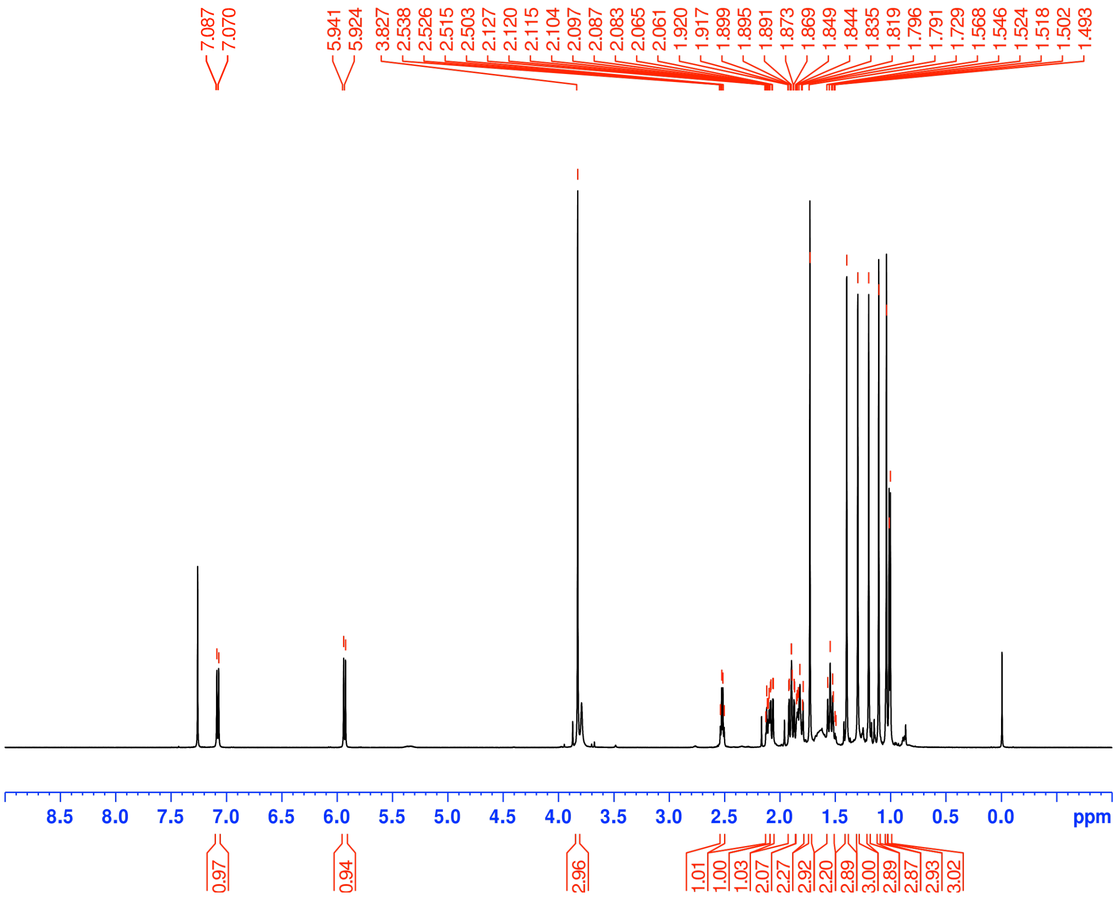

Figure S86. ^{1}H NMR spectrum of insuetusin B8 (**13**) in $CDCl_3$ at 600 MHz.

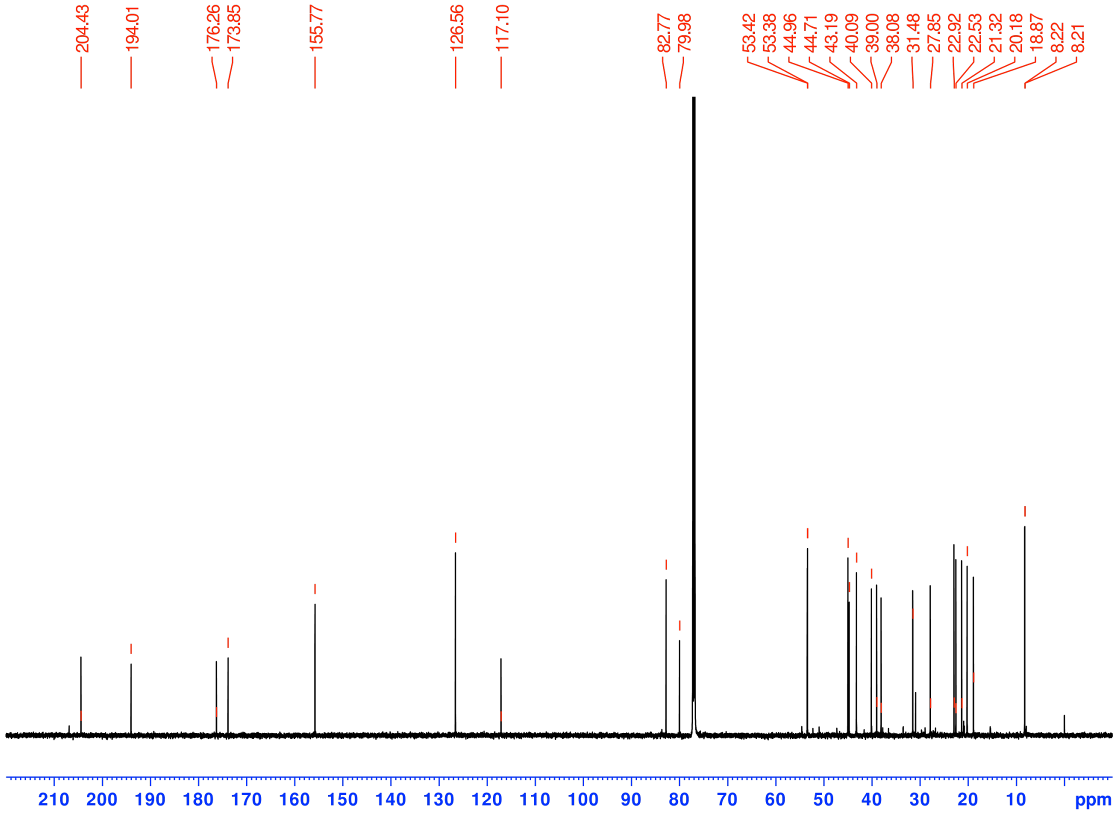

Figure S87. ^{13}C NMR spectrum of insuetusin B8 (**13**) in $CDCl_3$ at 150 MHz.

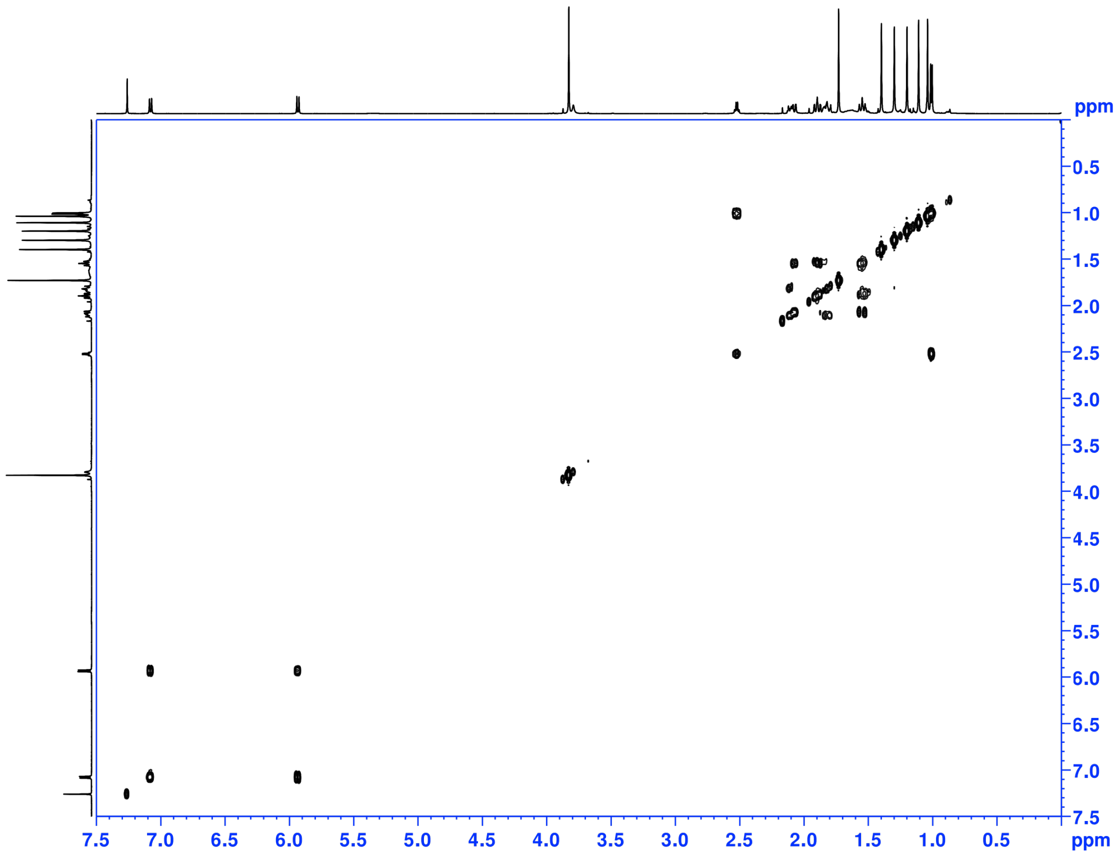

Figure S88. ^{1}H-^{1}H COSY spectrum of insuetusin B8 (**13**) in $CDCl_3$.

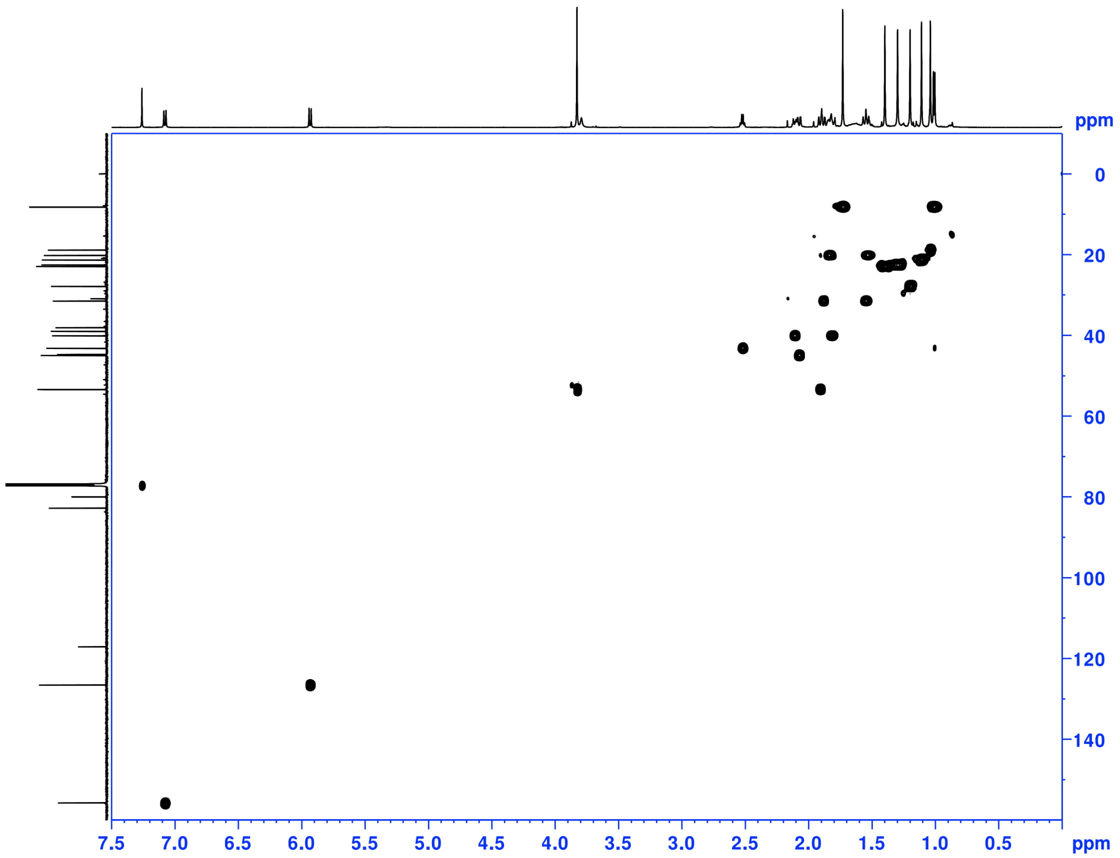

Figure S89. HSQC spectrum of insuetusin B8 (**13**) in $CDCl_3$.

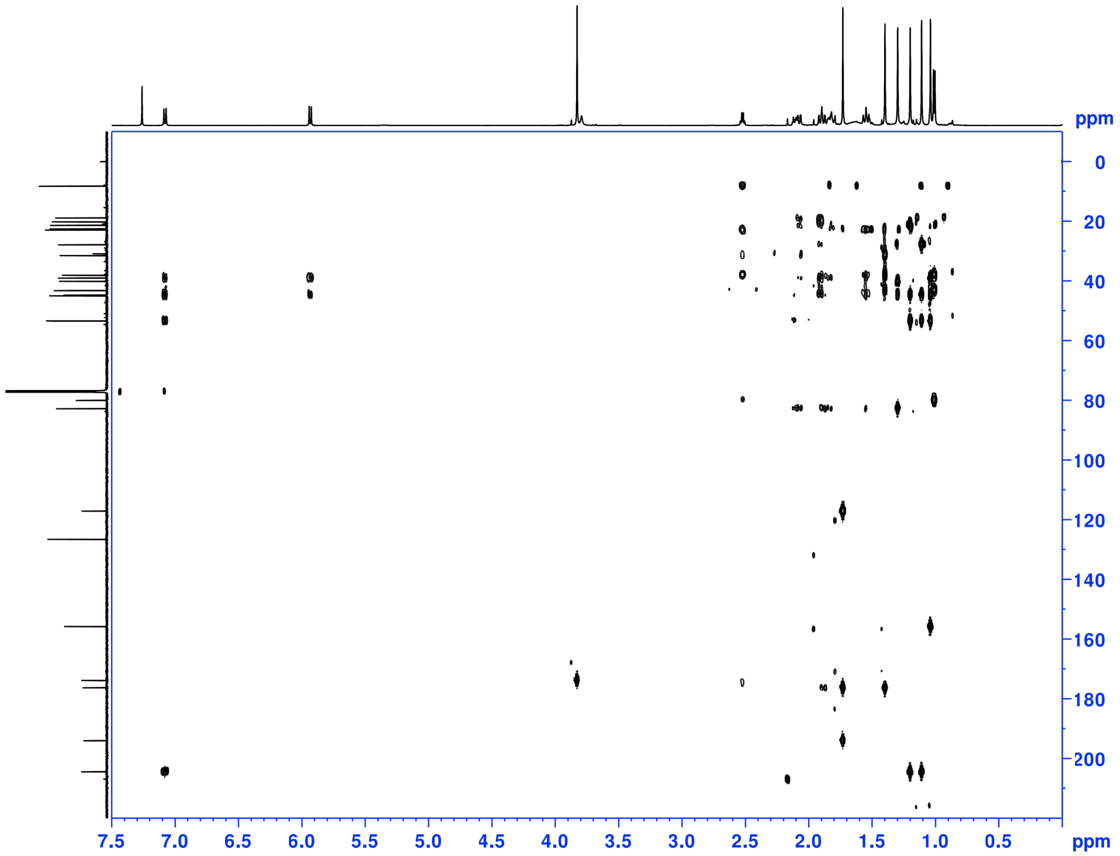

Figure S90. HMBC spectrum of insuetusin B8 (**13**) in $CDCl_3$.

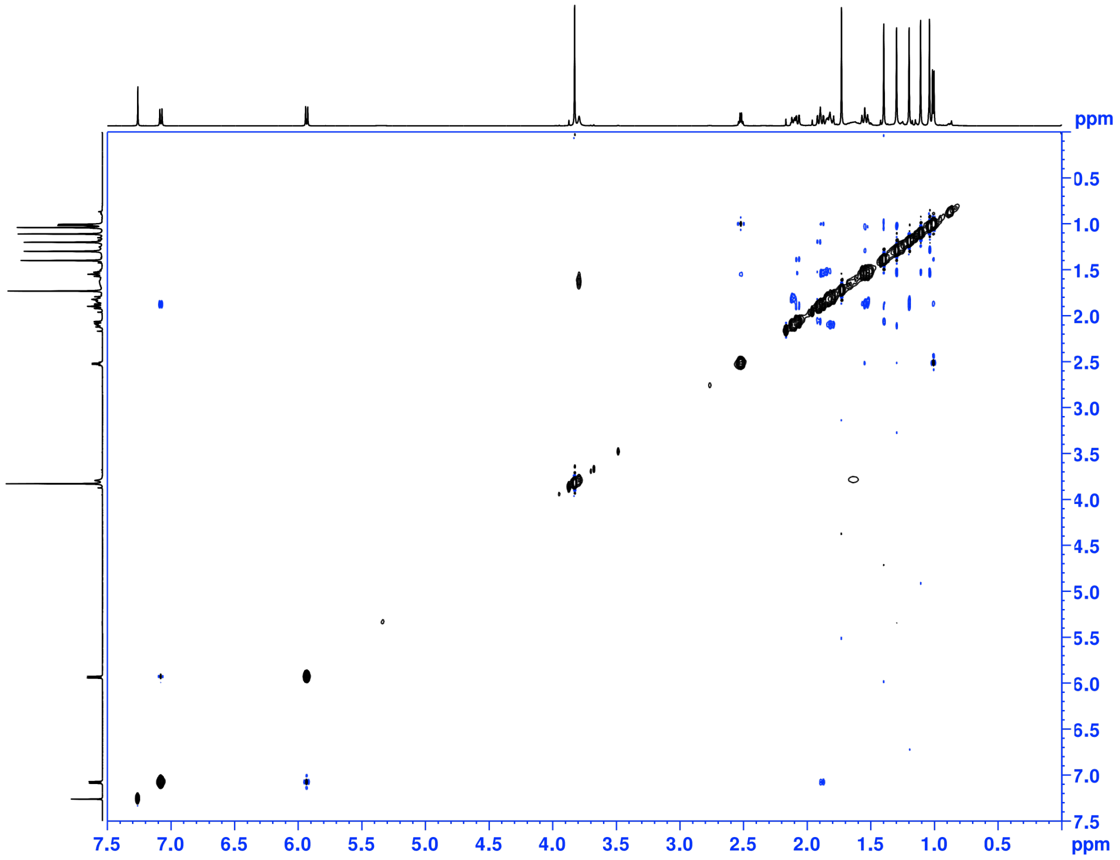

Figure S91. NOESY spectrum of insuetusin B8 (**13**) in $CDCl_3$.

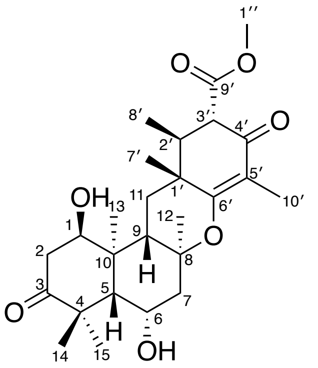

14

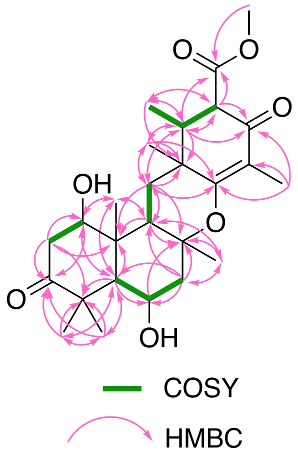

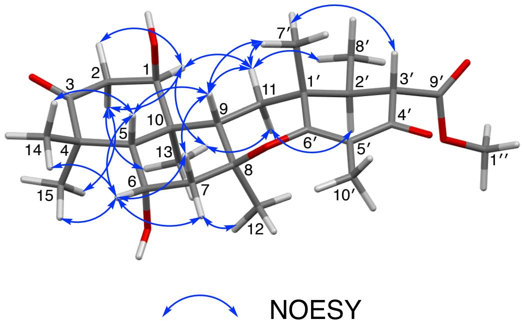

position	δC, type	δH, mult. (*J* in Hz)
1	73.6, CH	3.98, t (2.8)
2	42.8, CH_2	3.28 (α), dd (15.8, 3.0)
		2.33 (β), dd (15.5, 3.3)
3	213.7, C	
4	49.2, C	
5	49.0, CH	1.95, d (1.5)
6	69.2, CH_2	4.68, brt (2.8)
7	48.2, CH_2	2.17 (α), dd (14.0, 3.0)
		1.98 (β), dd (14.0, 3.0)
8	82.3, C	
9	42.5, CH	2.59, dd (13.0, 2.7)
10	40.8, C	
11	30.4, CH_2	1.56 (α), t (13.0)
		1.72 (β), dd (12.8, 2.6)
12	24.0, CH_3	1.52, s
13	16.7, CH_3	1.39, s
14	24.4, CH_3	1.22, s
15	23.4, CH_3	1.42, s
1′	37.9, C	
2′	40.9, CH	2.43, dq (13.0, 6.7)
3′	58.0, CH	3.28, d (12.9)
4′	193.6, C	
5′	118.2, C	
6′	175.4, C	
7′	19.7, CH_3	1.17, s
8′	12.6, CH_3	0.95, d (6.7)
9′	171.6, C	
10′	8.1, CH_3	1.68, s
1′′	52.1, CH_3	3.79, s

^{1}H NMR: 600 MHz, ^{13}C NMR: 150 MHz (in $CDCl_3$)

Figure S92. NMR data of insuetusin B9 (**14**).

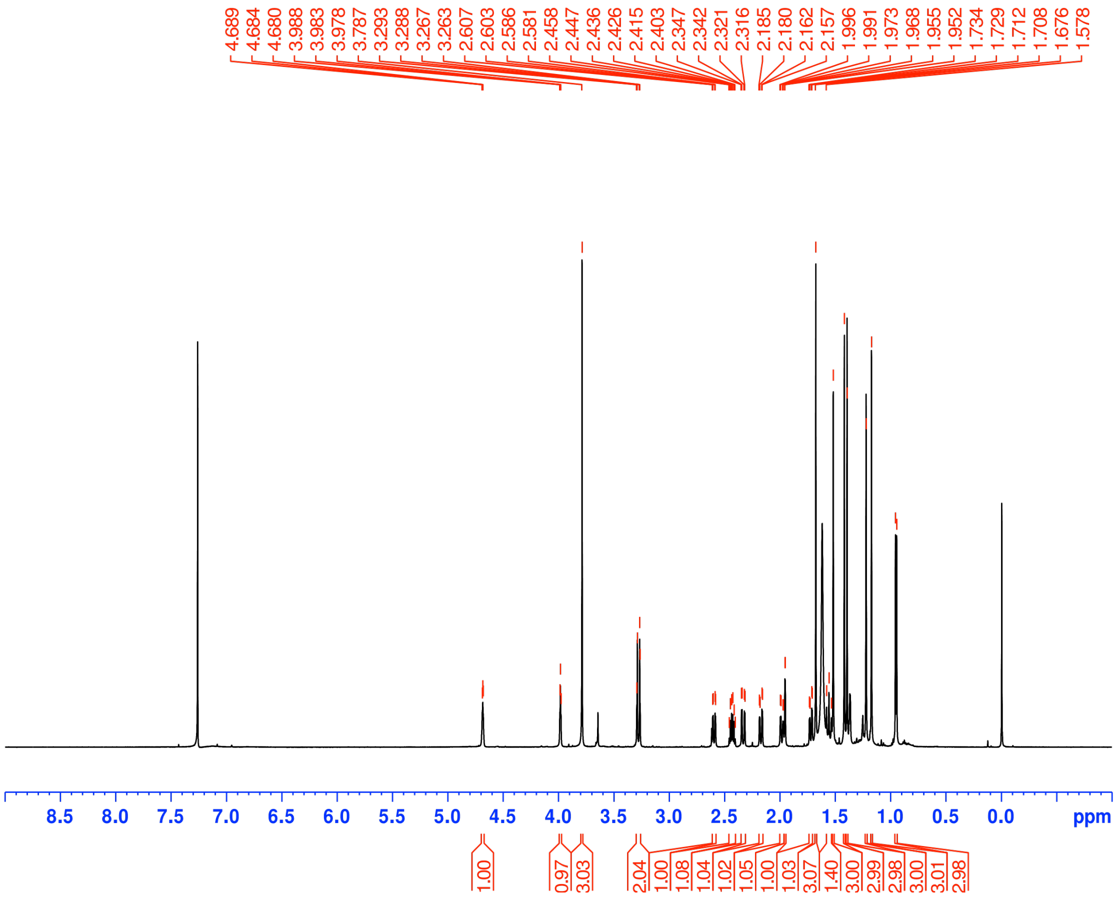

Figure S93. ^{1}H NMR spectrum of insuetusin B9 (**14**) in $CDCl_3$ at 600 MHz.

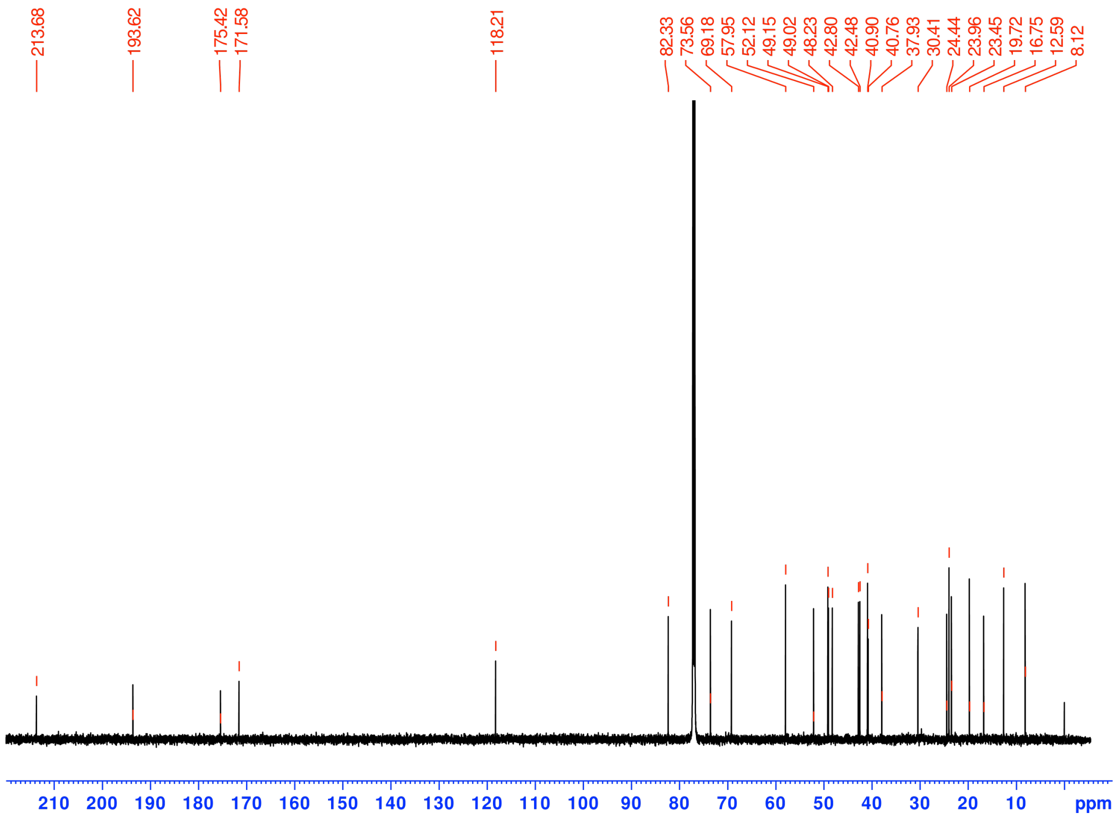

Figure S94. ^{13}C NMR spectrum of insuetusin B9 (**14**) in $CDCl_3$ at 150 MHz.

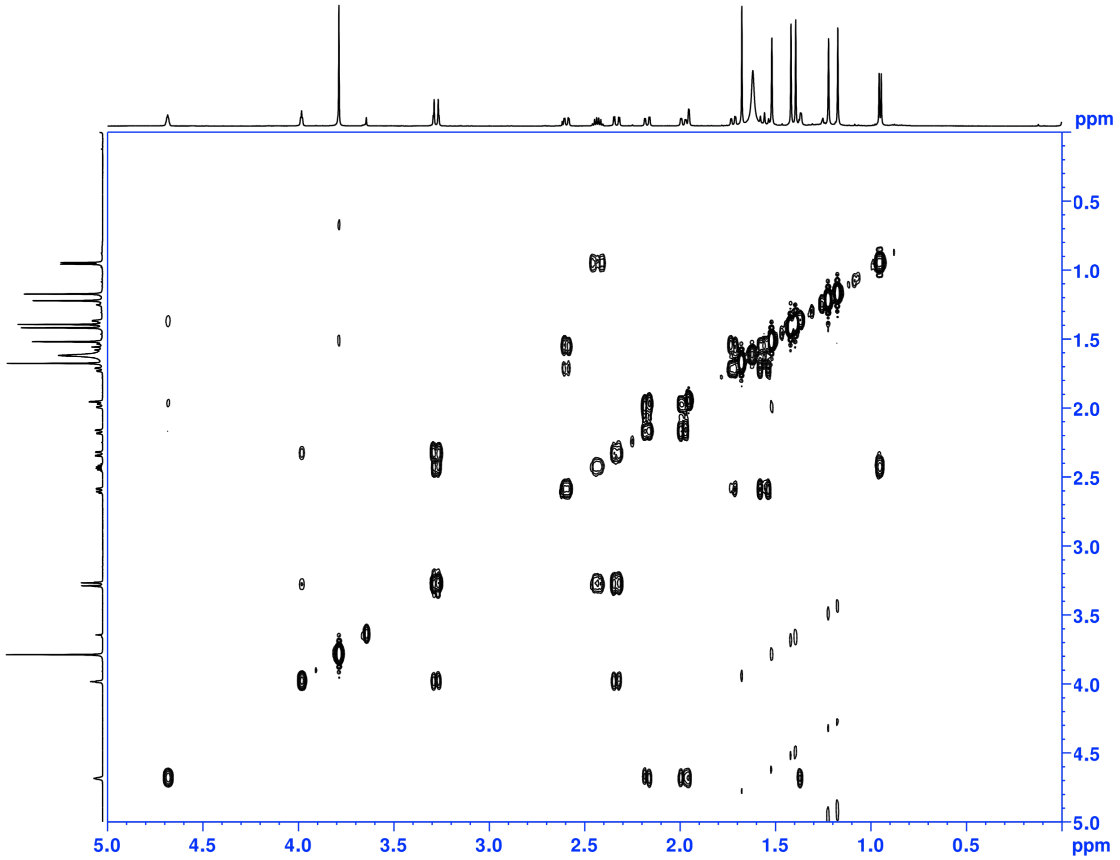

Figure S95. ^{1}H-^{1}H COSY spectrum of insuetusin B9 (**14**) in $CDCl_3$.

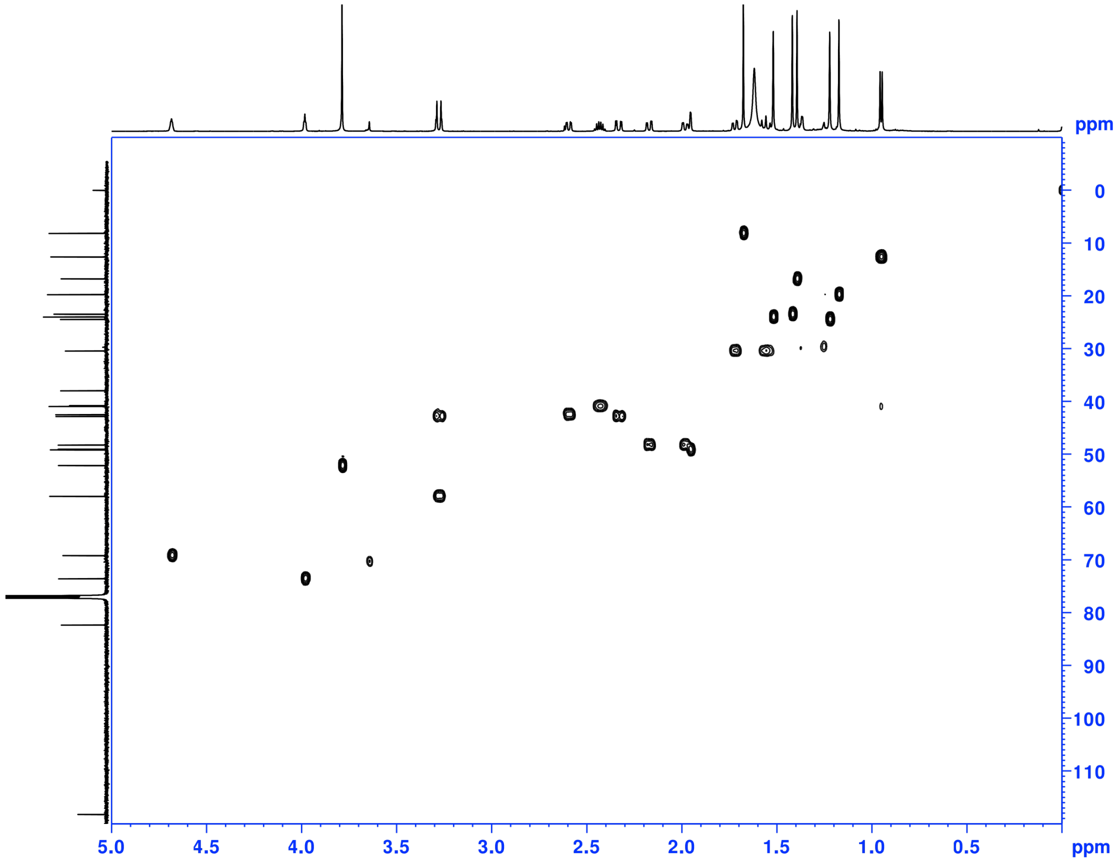

Figure S96. HSQC spectrum of insuetusin B9 (**14**) in $CDCl_3$.

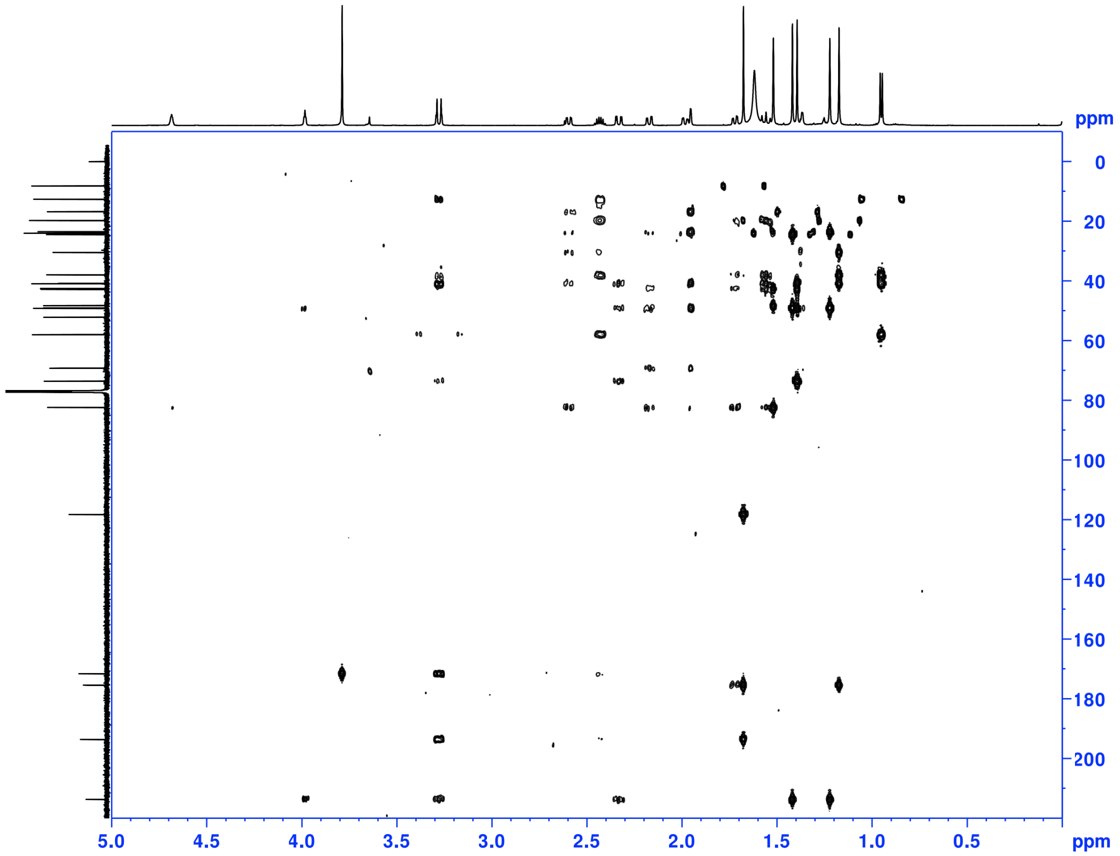

Figure S97. HMBC spectrum of insuetusin B9 (**14**) in $CDCl_3$.

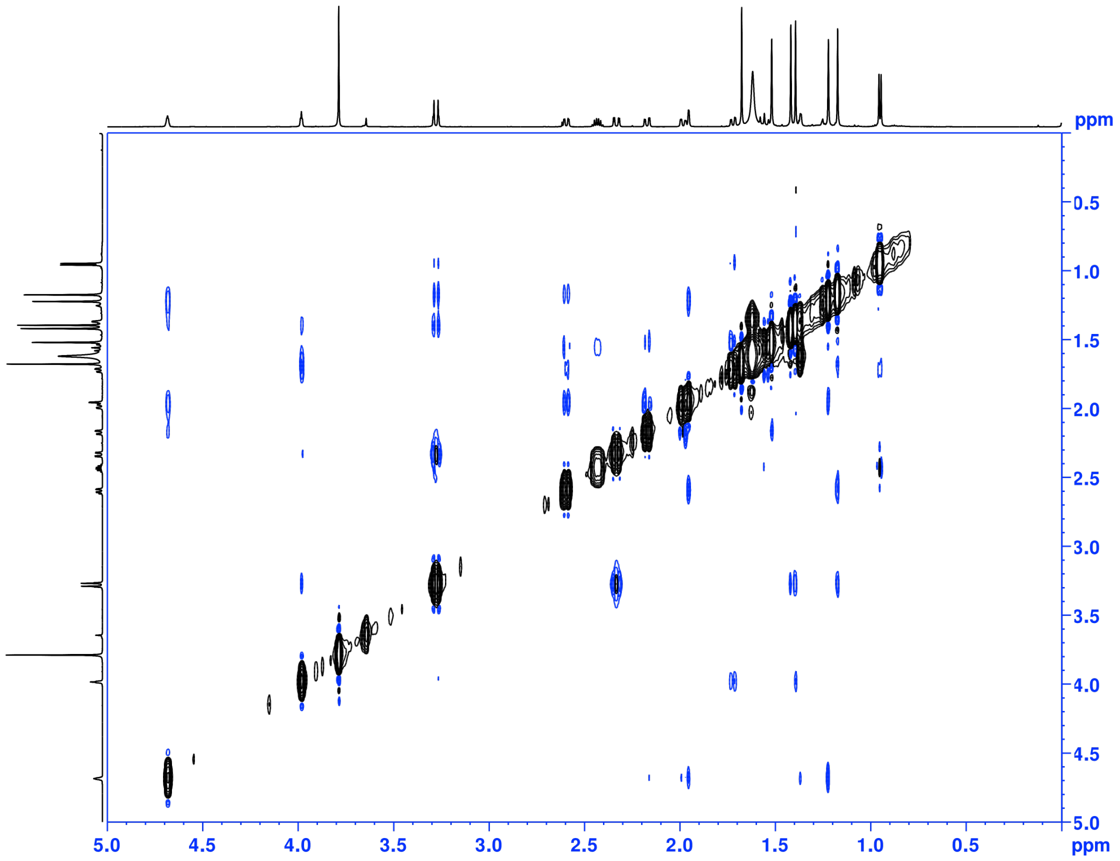

Figure S98. NOESY spectrum of insuetusin B9 (**14**) in $CDCl_3$.

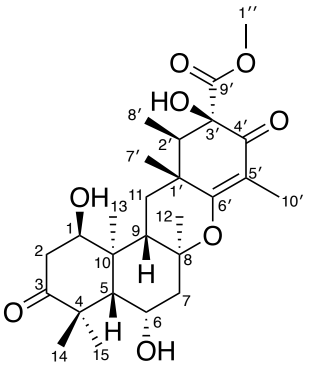

15

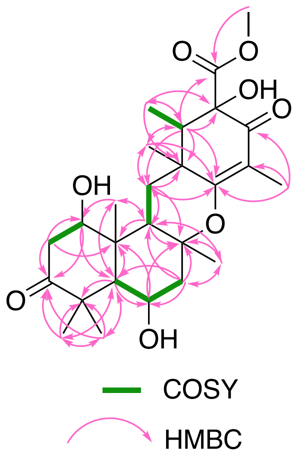

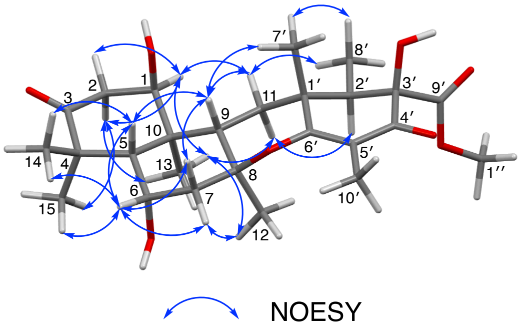

position	δ_C, type	δ_H, mult. (*J* in Hz)
1	73.5, CH	3.98, t (3.0)
2	42.8, CH_2	3.26 (α), dd (15.5, 3.0)
		2.34 (β), dd (15.5, 3.3)
3	213.6, C	
4	49.1, C	
5	49.0, CH	1.97, d (2.0)
6	69.2, CH_2	4.69, brs
7	48.2, CH_2	2.19 (α), dd (14.0, 3.0)
		2.02 (β), dd (13.9, 3.0)
8	82.9, C	
9	42.2, CH	2.64, dd (13.0, 2.8)
10	40.8, C	
11	31.0, CH_2	1.54 (α), t (12.8)
		1.72 (β), dd (11.8, 2.7)
12	24.1, CH_3	1.54, s
13	16.8, CH_3	1.39, s
14	24.5, CH_3	1.22, s
15	23.5, CH_3	1.41, s
1′	38.1, C	
2′	43.2, CH	2.51, q (7.0)
3′	80.1, C	
4′	194.1, C	
5′	116.9, C	
6′	177.1, C	
7′	22.9, CH_3	1.37, s
8′	8.2, CH_3	0.97, d (7.0)
9′	174.0, C	
10′	8.2, CH_3	1.72, s
1′′	53.4, CH_3	3.82, s

^{1}H NMR: 600 MHz, ^{13}C NMR: 150 MHz (in $CDCl_3$)

Figure S99. NMR data of insuetusin B10 (**15**).

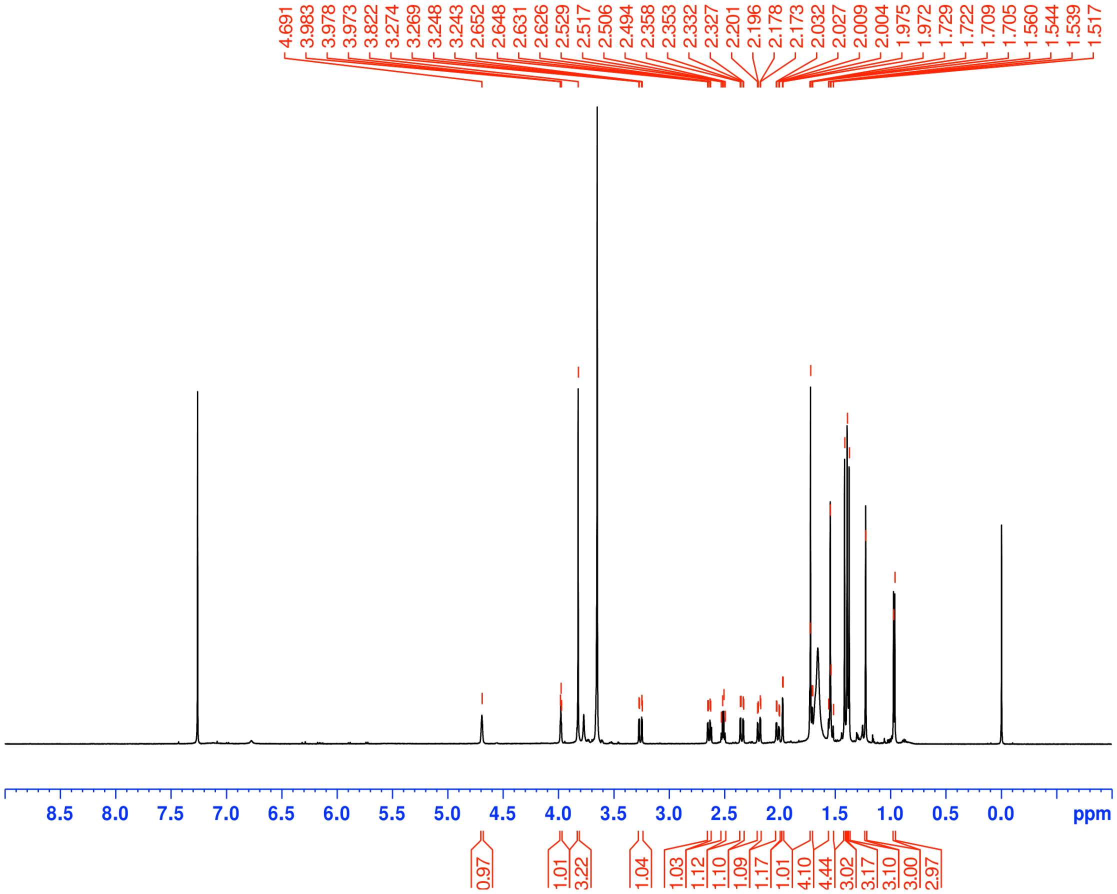

Figure S100. ^{1}H NMR spectrum of insuetusin B10 (**15**) in $CDCl_3$ at 600 MHz.

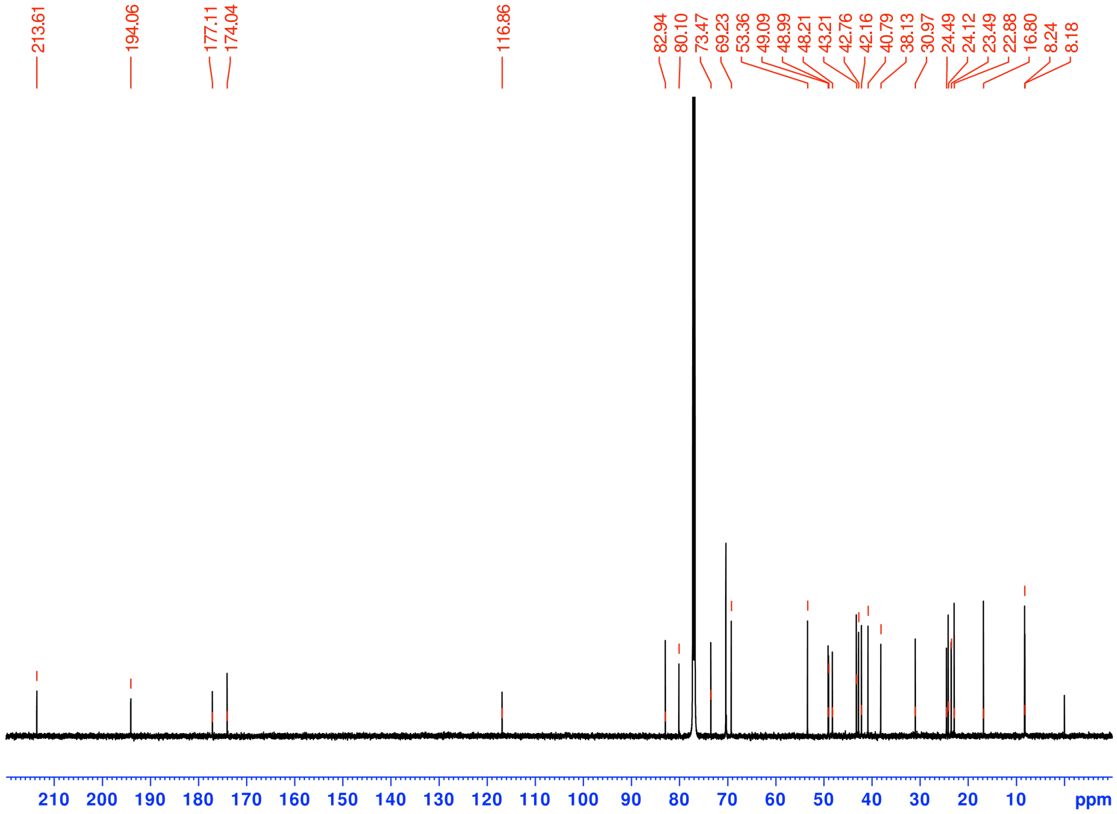

Figure S101. ^{13}C NMR spectrum of insuetusin B10 (**15**) in $CDCl_3$ at 150 MHz.

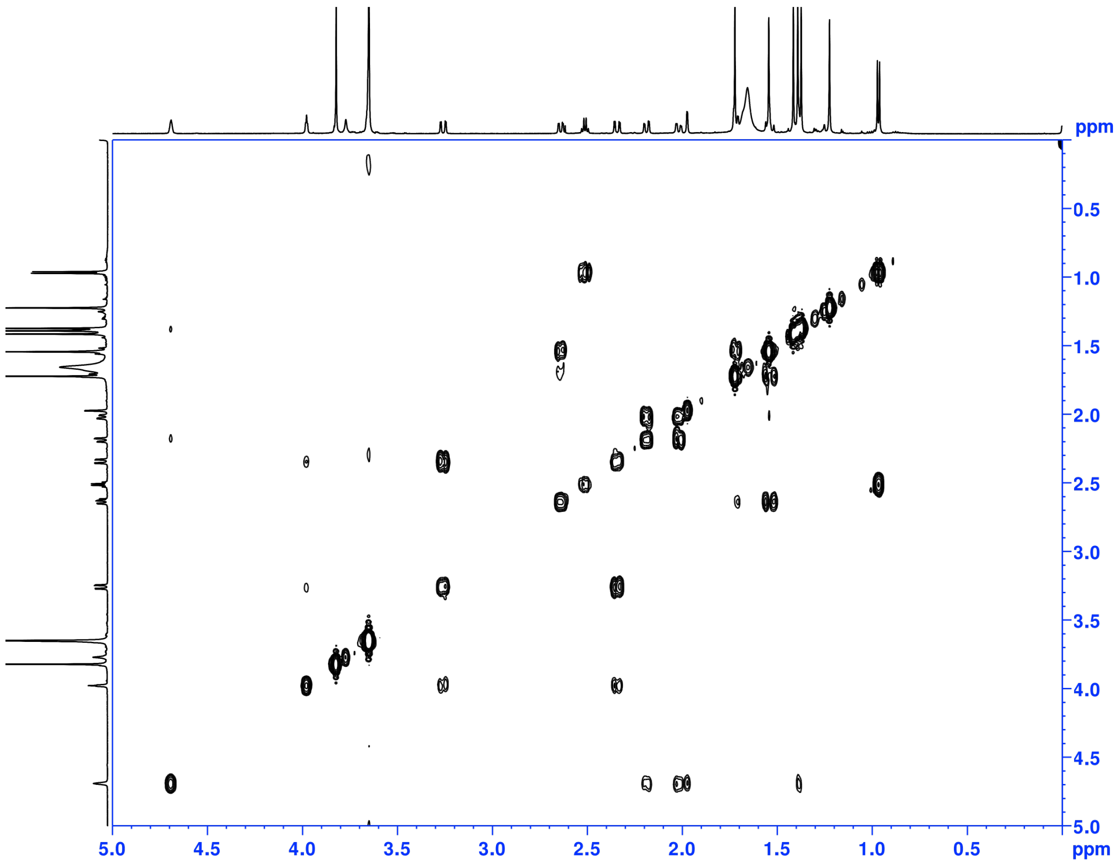

Figure S102. ^{1}H-^{1}H COSY spectrum of insuetusin B10 (**15**) in $CDCl_3$.

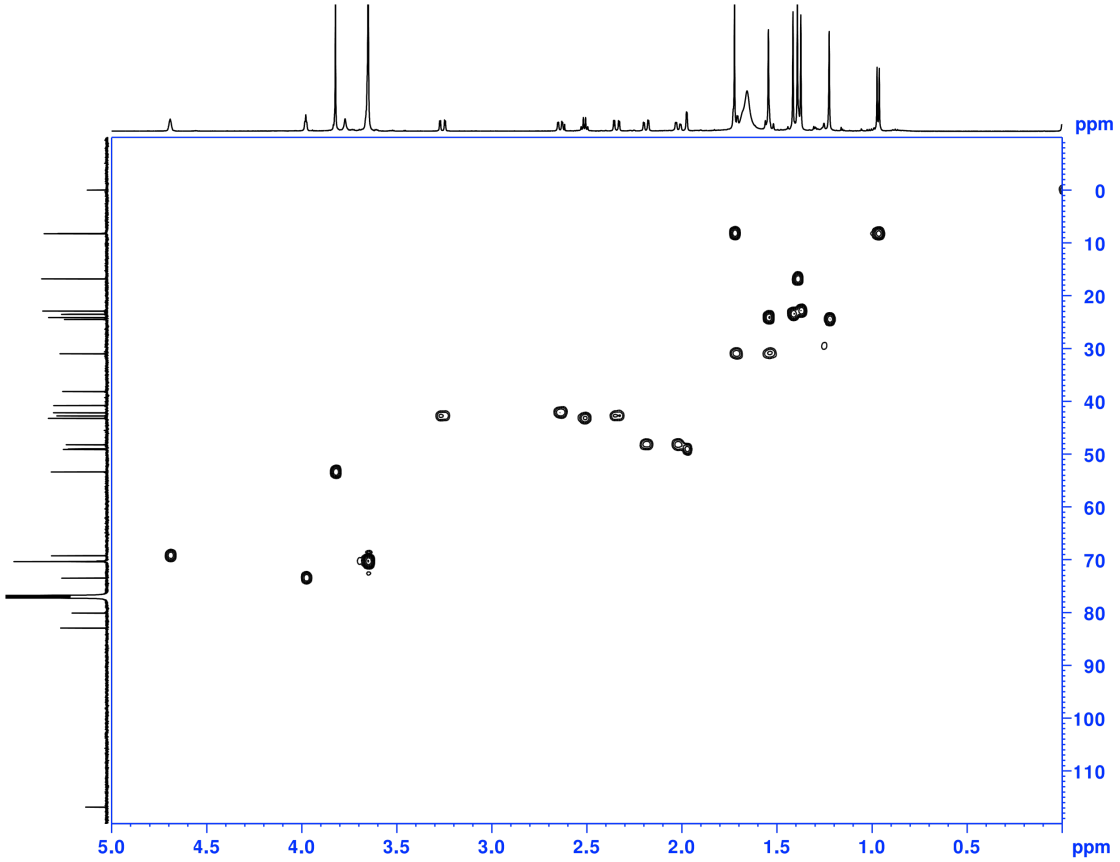

Figure S103. HSQC spectrum of insuetusin B10 (**15**) in $CDCl_3$.

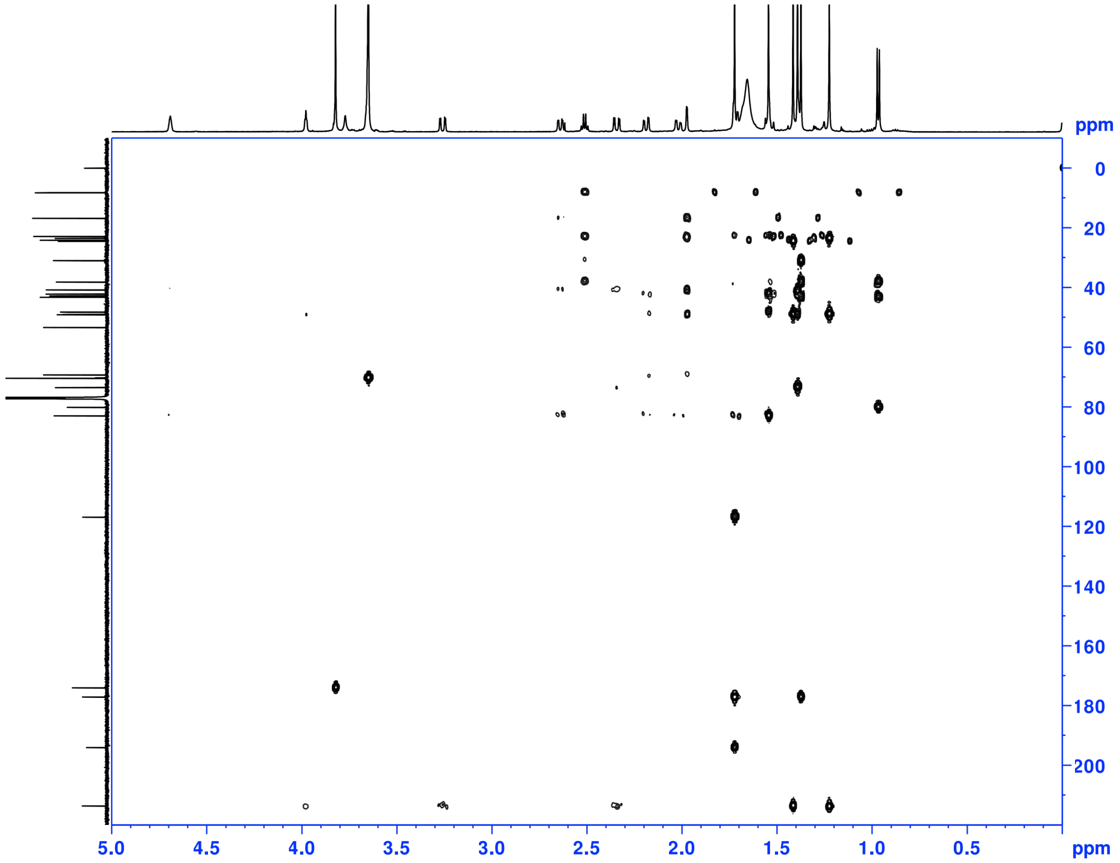

Figure S104. HMBC spectrum of insuetusin B10 (**15**) in $CDCl_3$.

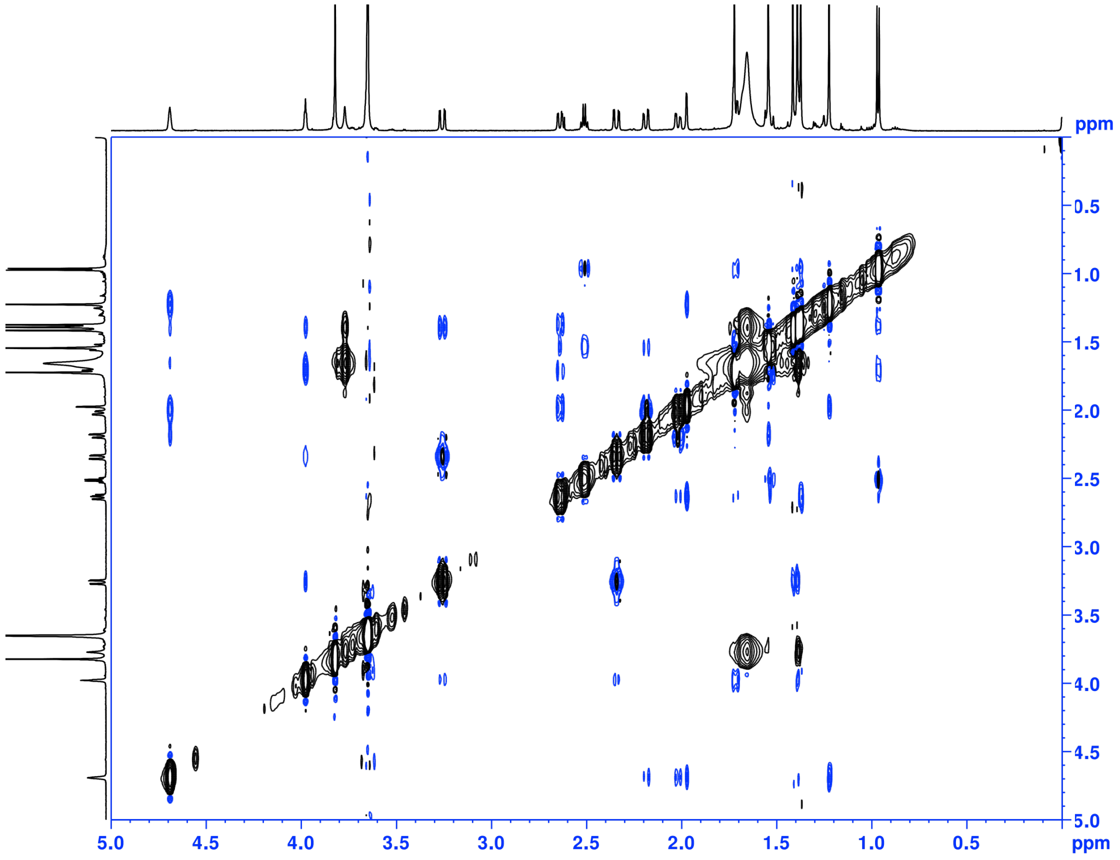

Figure S105. NOESY spectrum of insuetusin B10 (**15**) in $CDCl_3$.

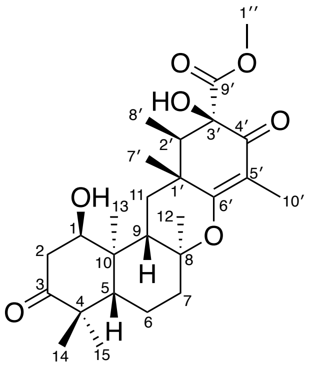

16

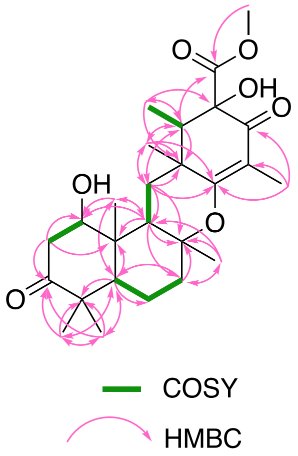

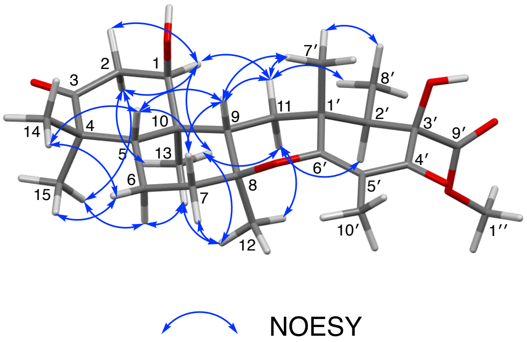

position	δ_C, type	δ_H, mult. (*J* in Hz)
1	72.2, CH	4.04, brs
2	43.2, CH_2	3.10 (α), dd (16.4, 3.8)
		2.41 (β), dd (16.4, 3.0)
3	214.3, C	
4	47.7, C	
5	47.3, CH	2.09, dd (12.6, 2.0)
6	20.1, CH_2	1.55 (α), m
		1.80 (β), m
7	39.9, CH_2	2.06 (α), dt (12.0, 3.0)
		1.81 (β), m
8	83.2, C	
9	41.8, CH	2.59, dd (13.0, 2.4)
10	40.7, C	
11	31.2, CH_2	1.43 (α), t (13.0)
		1.69 (β), dd (12.7, 2.4)
12	22.5, CH_3	1.30, s
13	15.4, CH_3	0.99, s
14	25.3, CH_3	1.16, s
15	21.3, CH_3	1.06, s
1′	38.1, C	
2′	43.3, CH	2.49, q (7.0)
3′	80.1, C	
4′	194.1, C	
5′	116.6, C	
6′	177.2, C	
7′	22.9, CH_3	1.39, s
8′	8.2, CH_3	0.96, d (7.0)
9′	174.0, C	
10′	8.2, CH_3	1.73, s
1′′	53.4, CH_3	3.82, s

^1H NMR: 600 MHz, ^{13}C NMR: 150 MHz (in $CDCl_3$)

Figure S106. NMR data of insuetusin B11 (**16**).

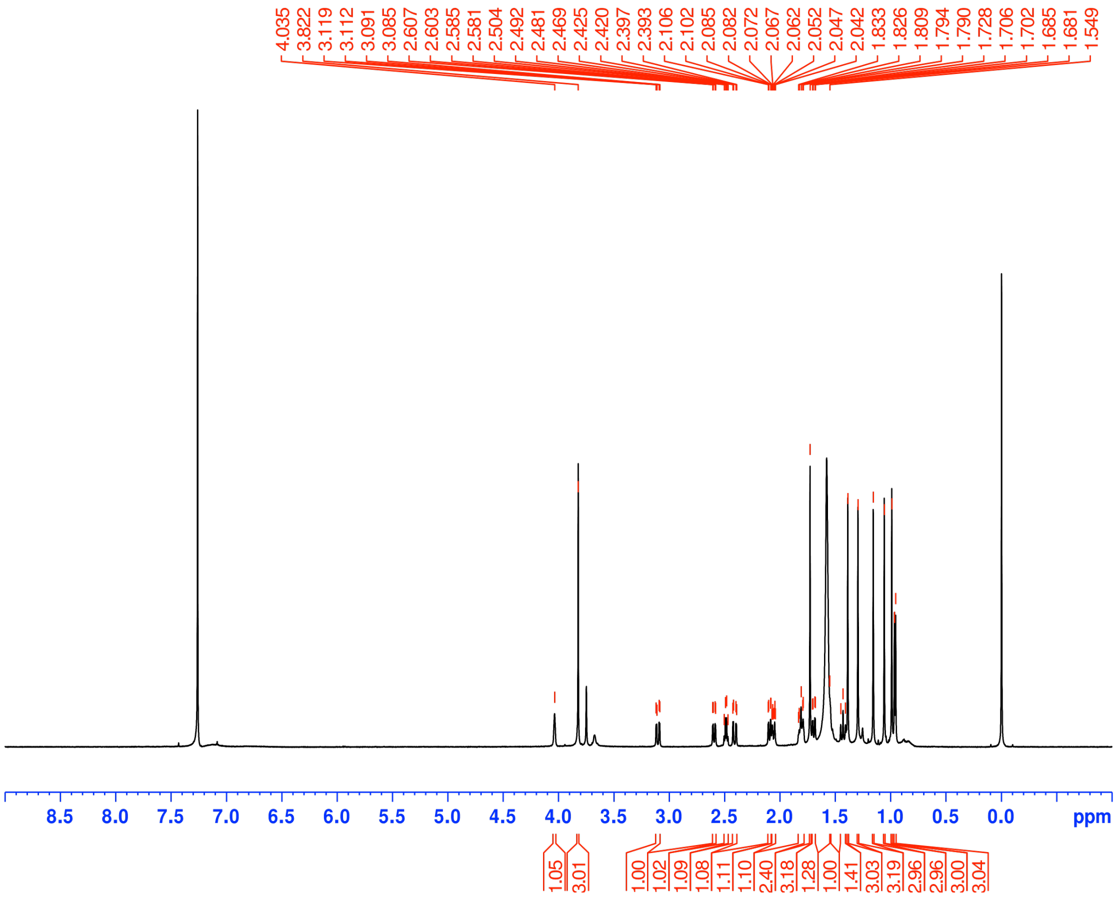

Figure S107. ^{1}H NMR spectrum of insuetusin B11 (**16**) in $CDCl_3$ at 600 MHz.

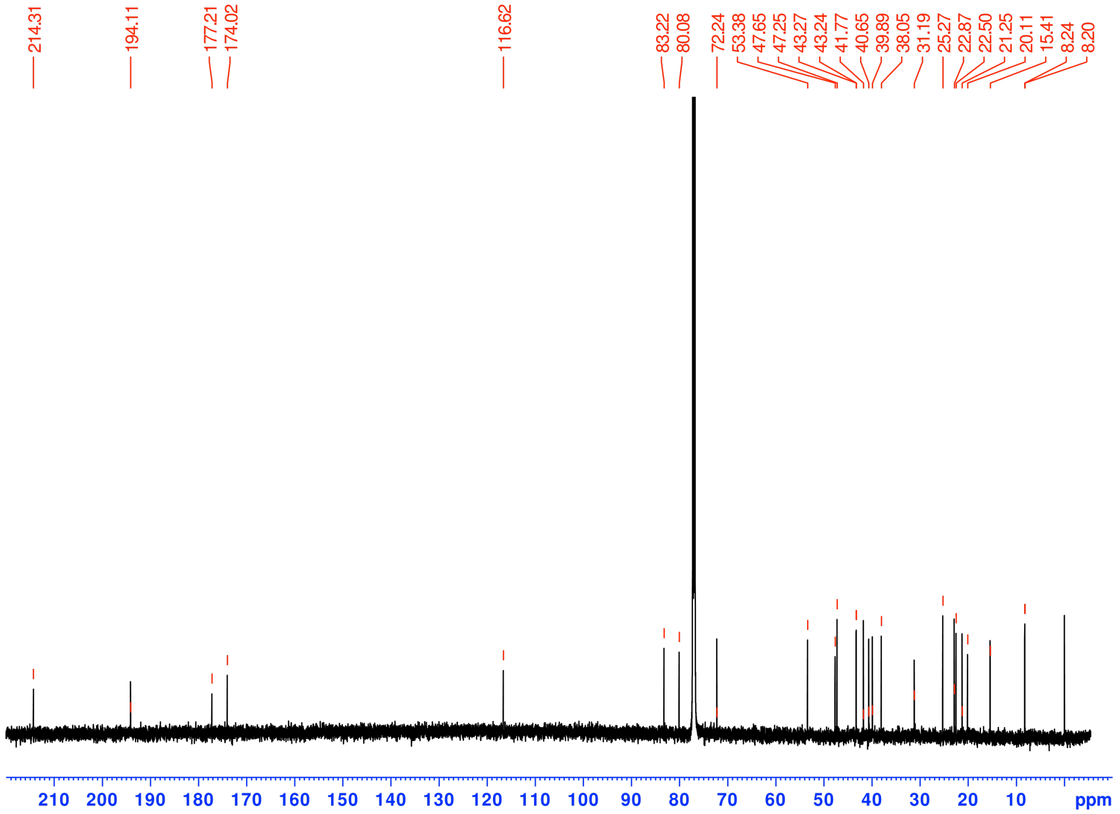

Figure S108. ^{13}C NMR spectrum of insuetusin B11 (**16**) in $CDCl_3$ at 150 MHz.

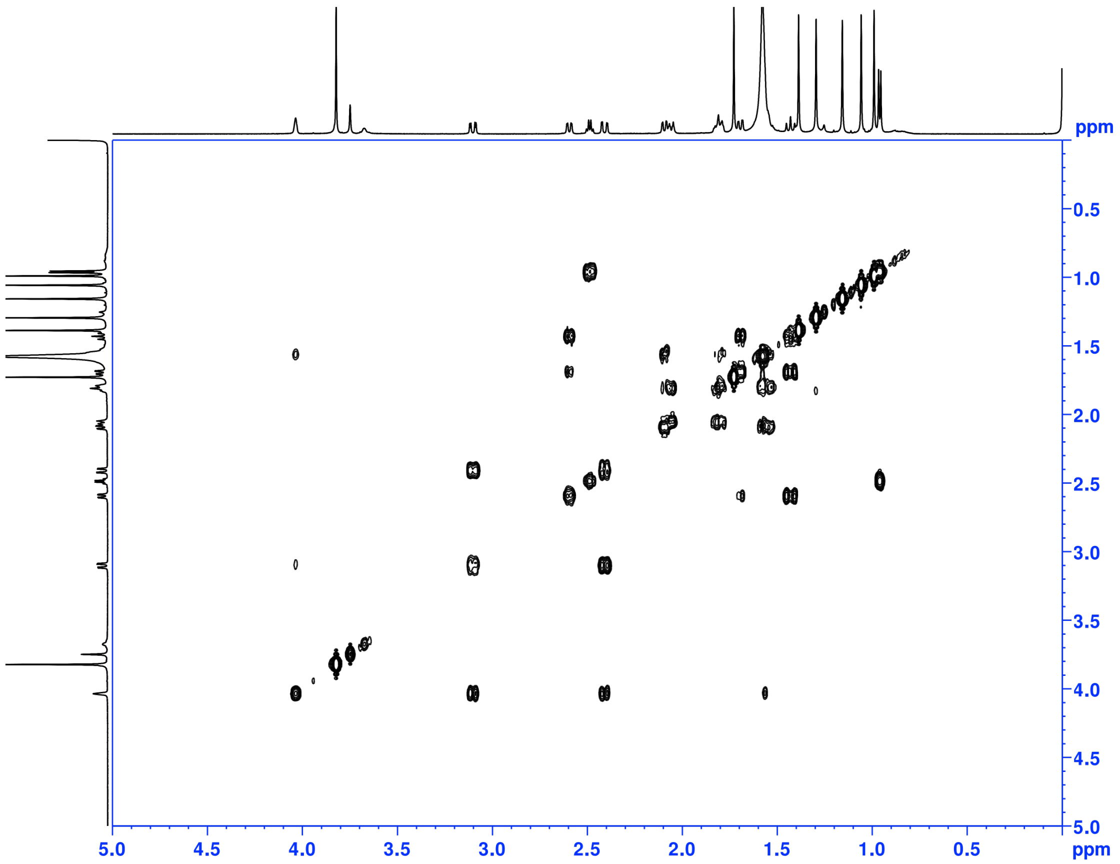

Figure S109. ^{1}H-^{1}H COSY spectrum of insuetusin B11 (**16**) in $CDCl_3$.

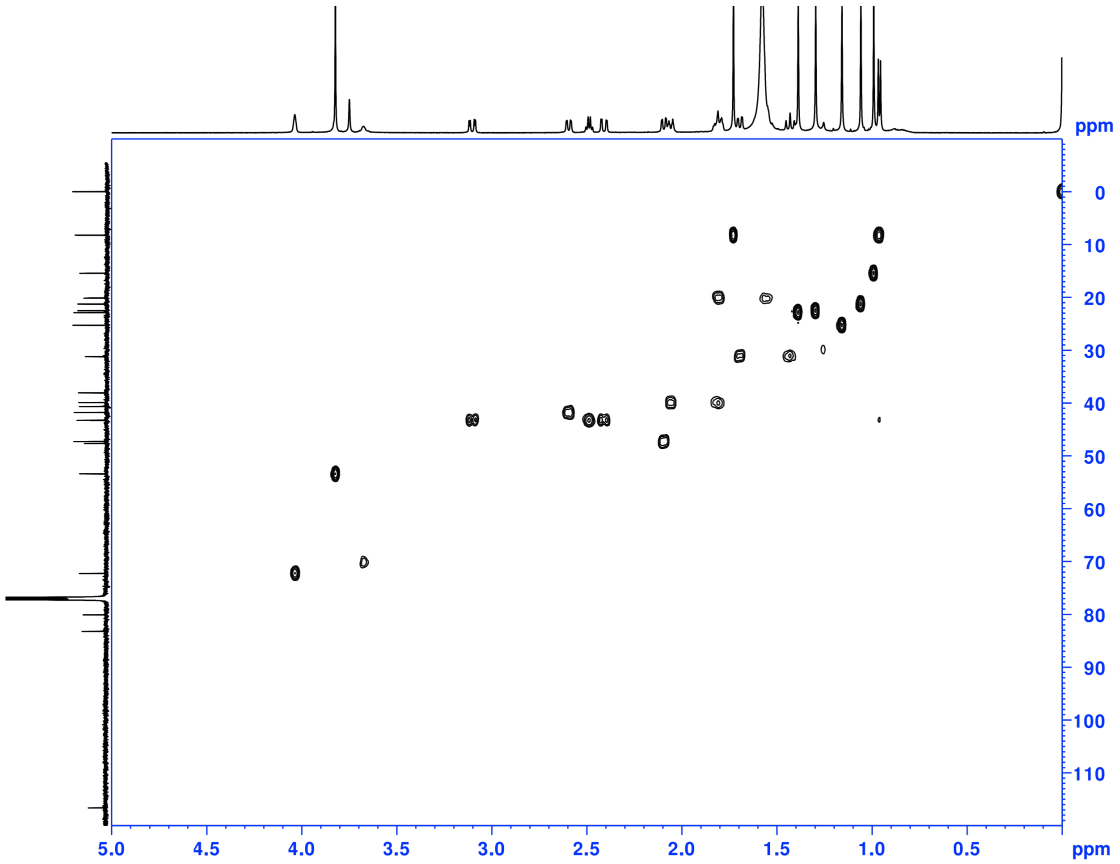

Figure S110. HSQC spectrum of insuetusin B11 (**16**) in $CDCl_3$.

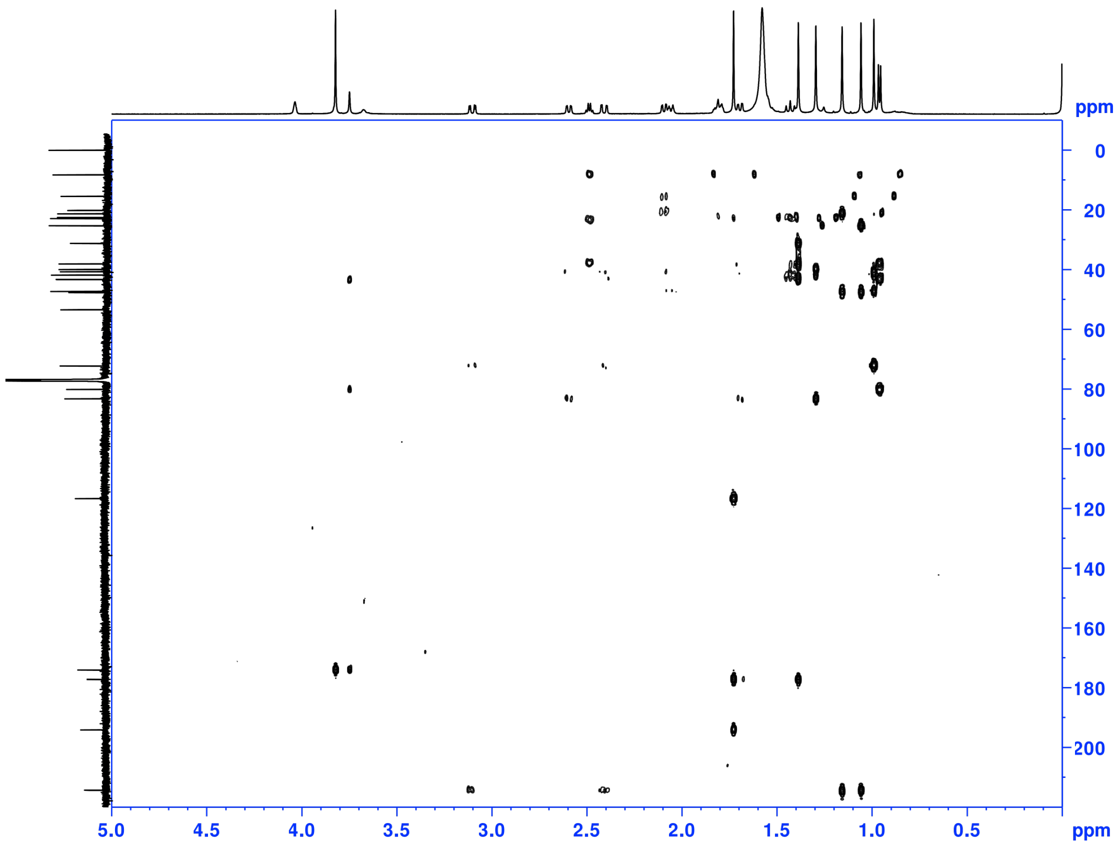

Figure S111. HMBC spectrum of insuetusin B11 (**16**) in $CDCl_3$.

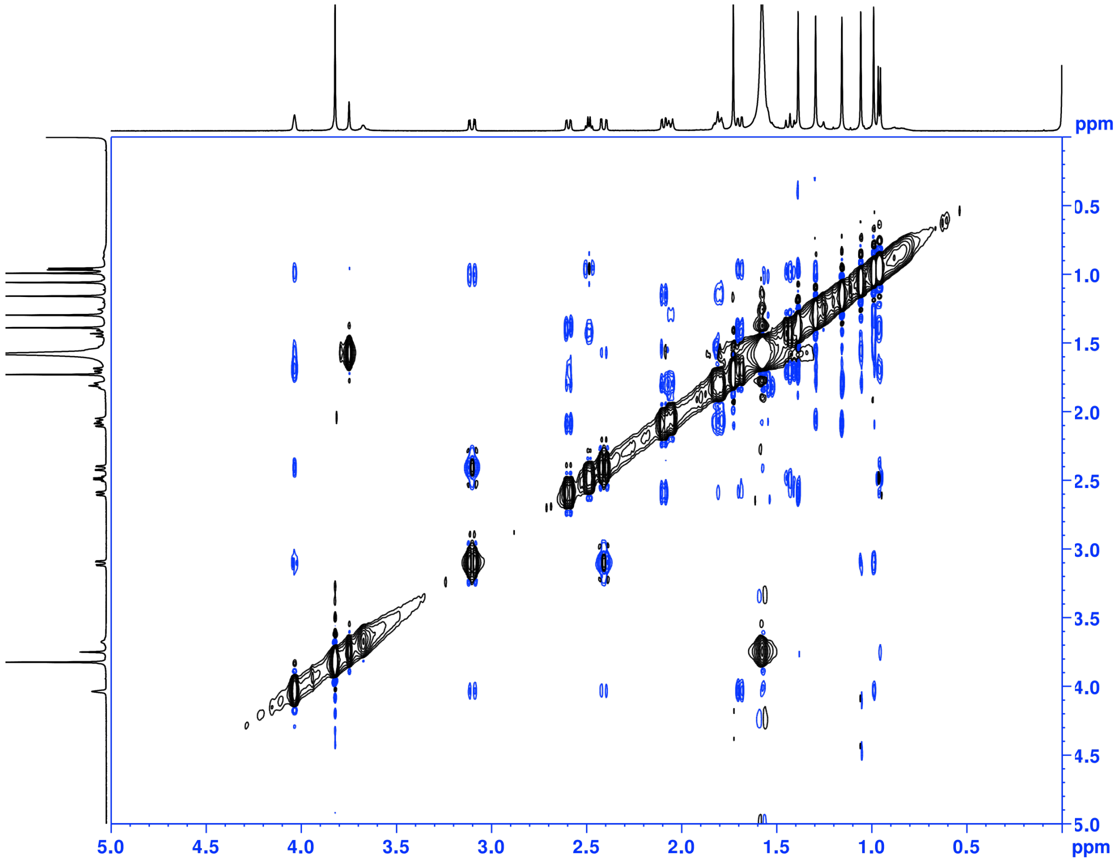

Figure S112. NOESY spectrum of insuetusin B11 (**16**) in $CDCl_3$.

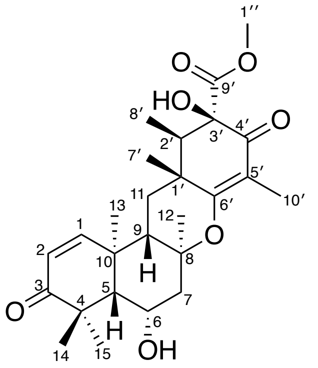

17

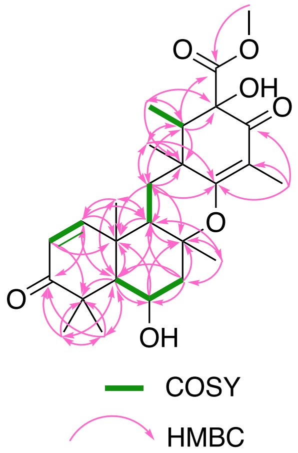

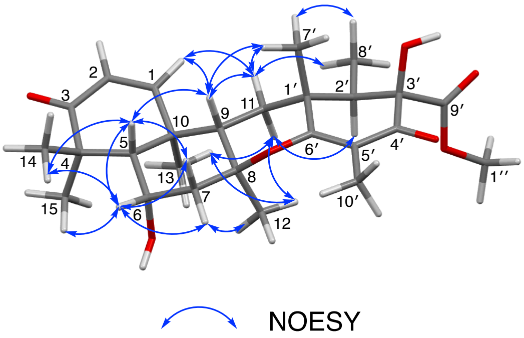

position	δ_C, type	δ_H, mult. (J in Hz)
1	155.2, CH	6.99, d (10.2)
2	125.1, CH	5.89, d (10.2)
3	204.2, C	
4	45.6, C	
5	54.3, CH	1.90, brs
6	68.1, CH	4.68, brs
7	47.9, CH_2	2.26 (α), dd (14.0, 2.4)
		2.03 (β), brd (12.0)
8	82.4, C	
9	45.9, CH	2.11, brd (11.4)
10	39.2, C	
11	31.4, CH_2	1.69 (α), t (13.3)
		1.93 (β), dd (13.0, 1.5)
12	24.3, CH_3	1.56, s
13	23.0, CH_3	1.39, s
14	26.2, CH_3	1.30, s
15	23.6, CH_3	1.41, s
1′	38.1, C	
2′	43.1, CH	2.56, q (6.9)
3′	80.0, C	
4′	194.0, C	
5′	117.4, C	
6′	176.1, C	
7′	18.5, CH_3	1.41, s
8′	8.3, CH_3	1.02, d (6.9)
9′	173.9, C	
10′	8.2, CH_3	1.73, s
1′′	53.5, CH_3	3.84, s

^1H NMR: 600 MHz, ^{13}C NMR: 150 MHz (in $CDCl_3$)

Figure S113. NMR data of insuetusin B12 (**17**).

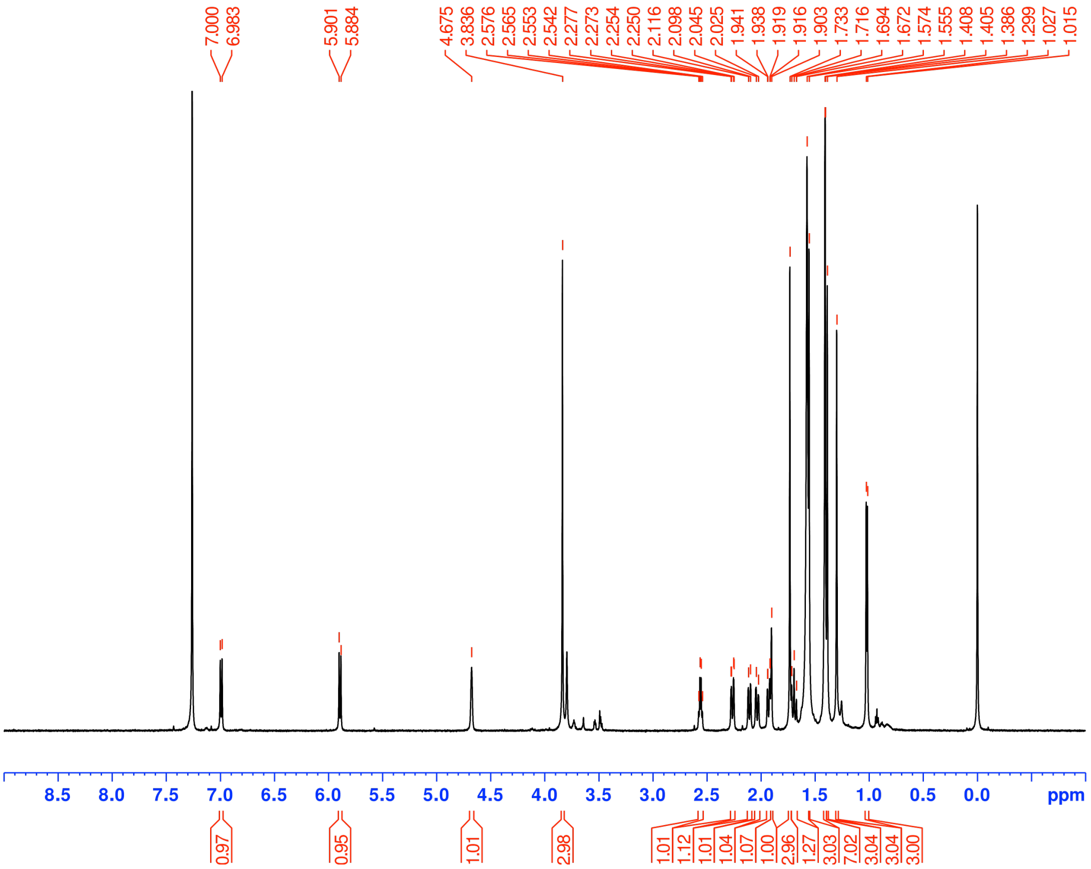

Figure S114. ^{1}H NMR spectrum of insuetusin B12 (**17**) in $CDCl_3$ at 600 MHz.

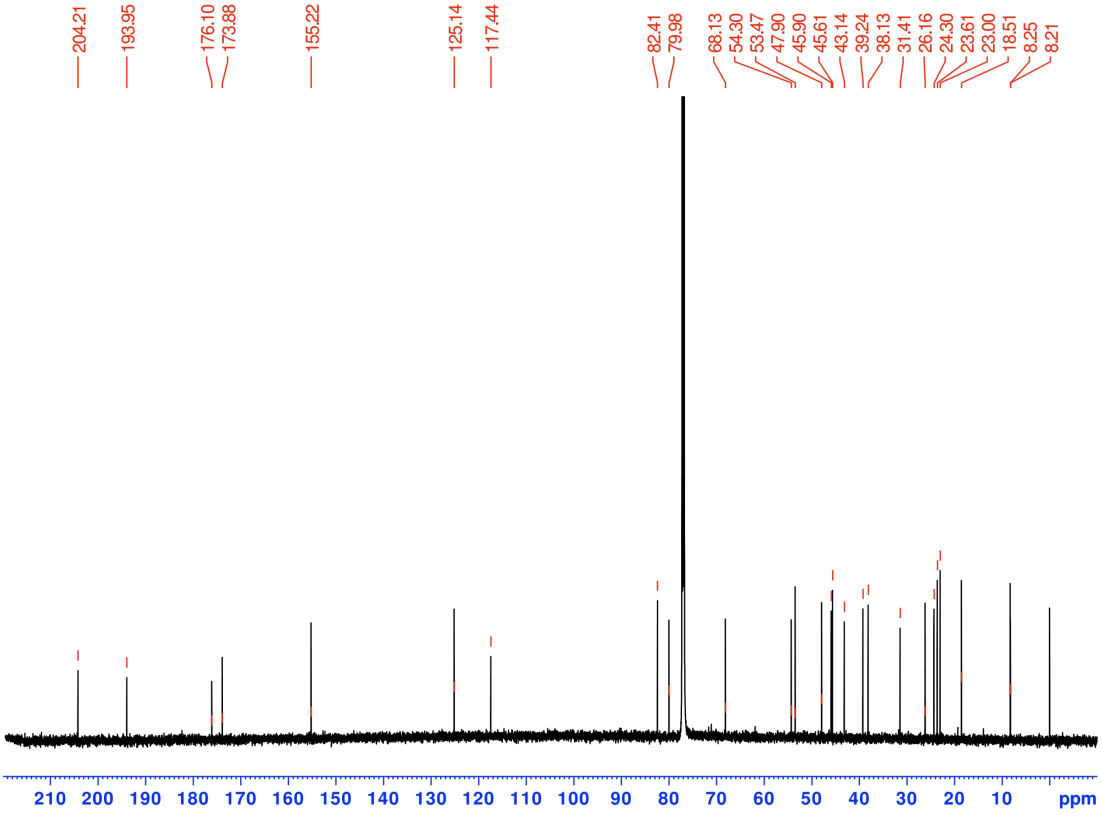

Figure S115. ^{13}C NMR spectrum of insuetusin B12 (**17**) in $CDCl_3$ at 150 MHz.

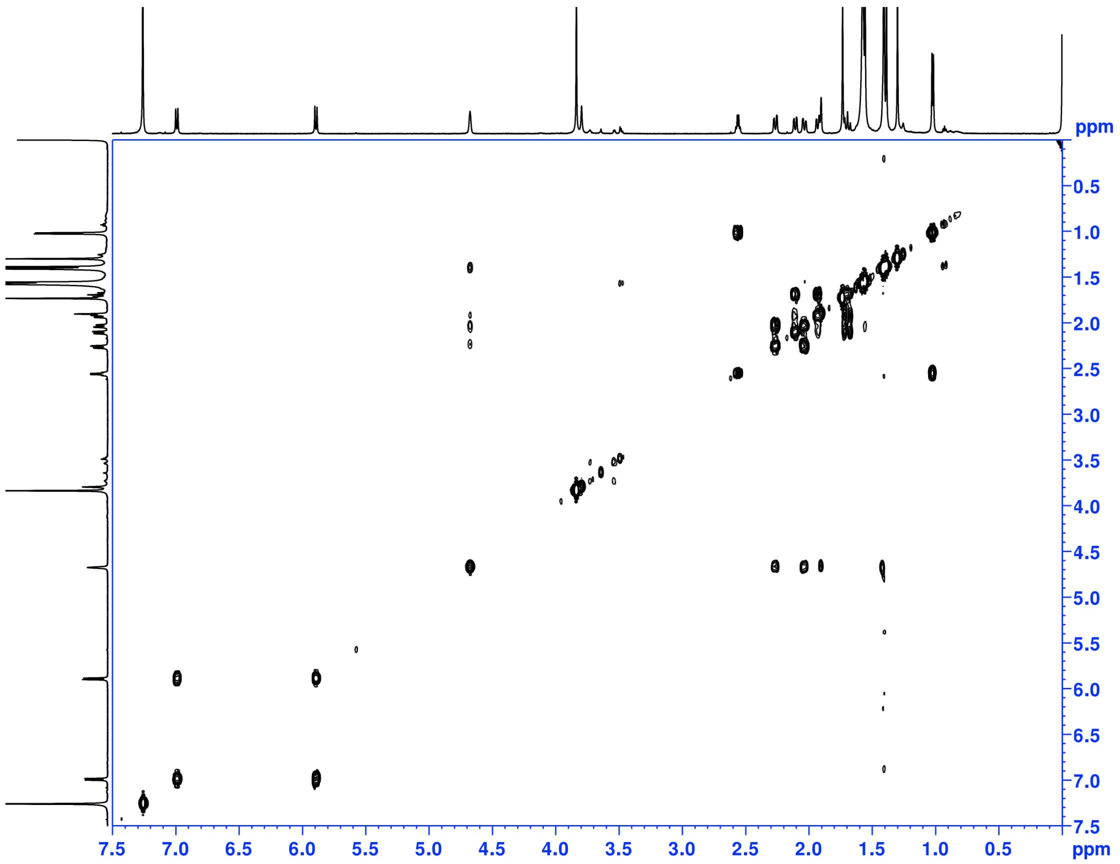

Figure S116. ^{1}H-^{1}H COSY spectrum of insuetusin B12 (**17**) in $CDCl_3$.

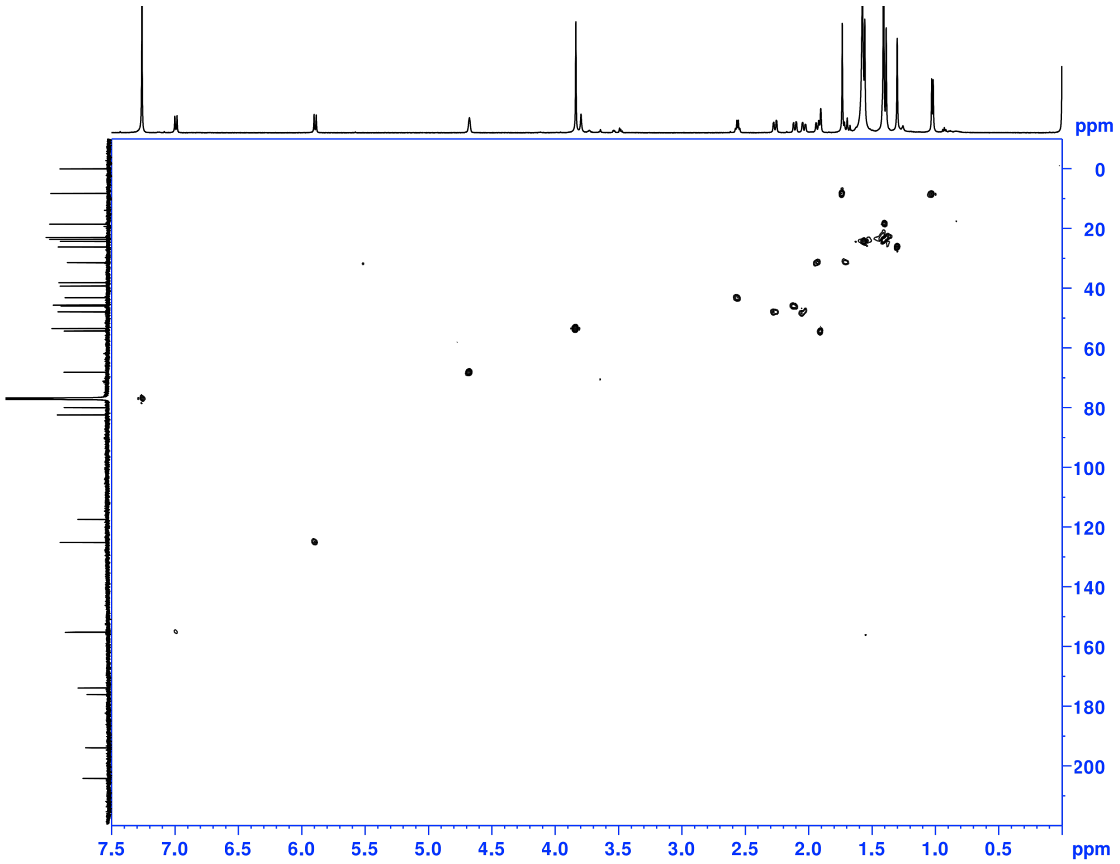

Figure S117. HSQC spectrum of insuetusin B12 (**17**) in $CDCl_3$.

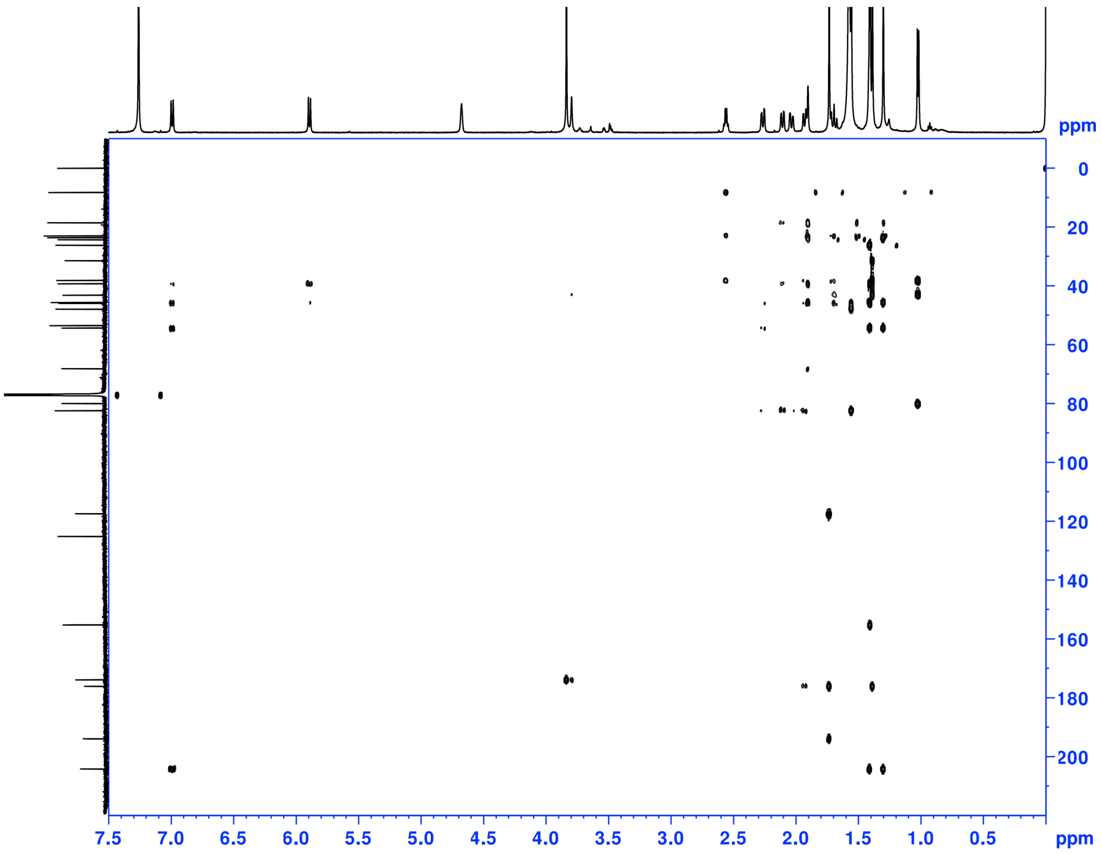

Figure S118. HMBC spectrum of insuetusin B12 (**17**) in $CDCl_3$.

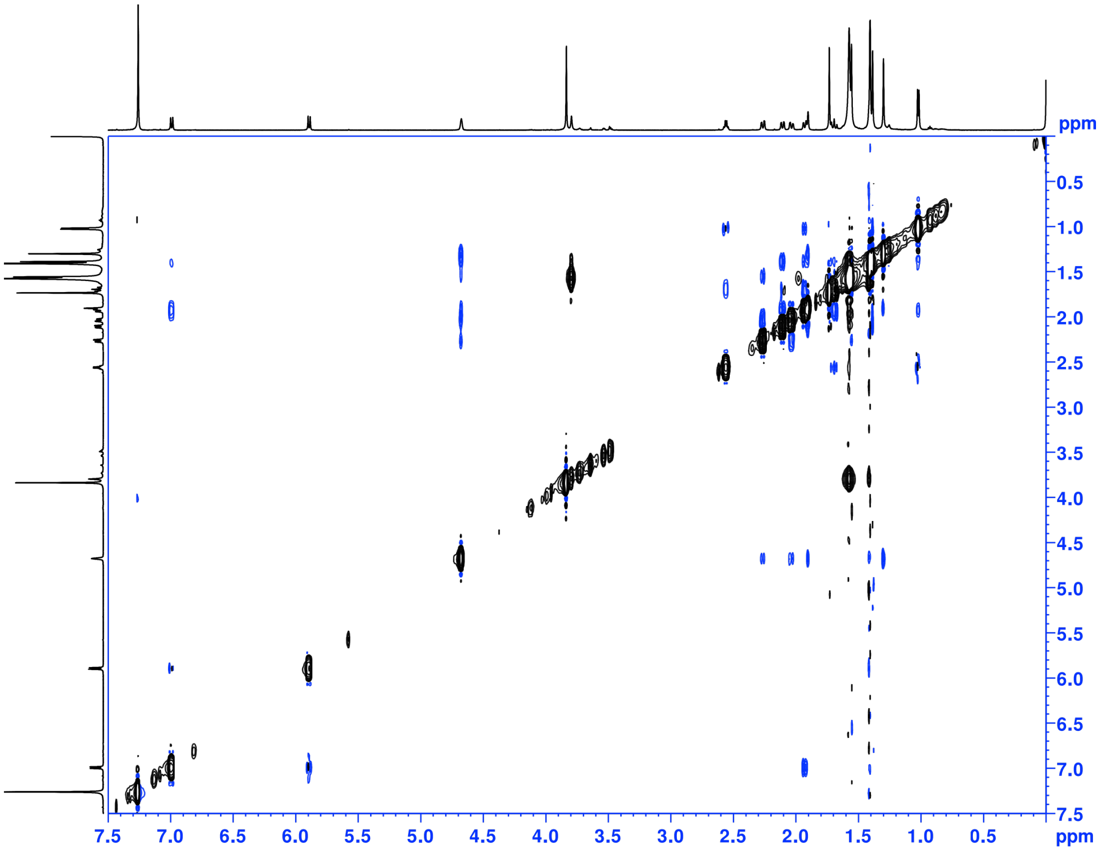

Figure S119. NOESY spectrum of insuetusin B12 (**17**) in $CDCl_3$.

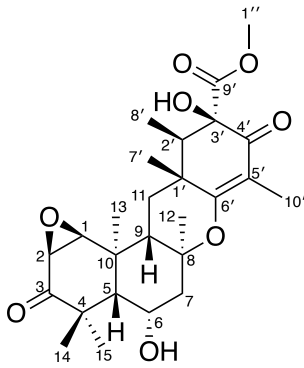

18

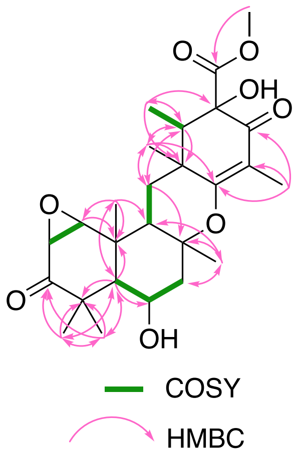

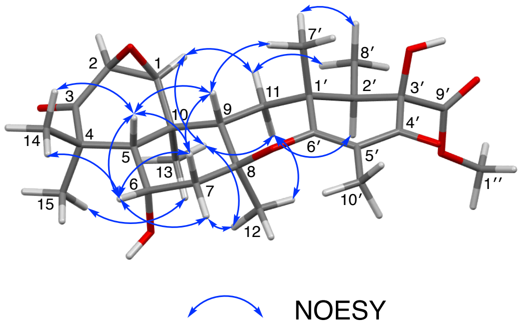

position	δ_C, type	δ_H, mult. (J in Hz)
1	62.1, CH	3.52, d (4.6)
2	56.2, CH	3.46, d (4.6)
3	210.6, C	
4	45.5, C	
5	48.1, CH	2.21, d (1.7)
6	67.4, CH	4.56, q (2.3)
7	47.4, CH_2	2.26 (α), dd (13.5, 3.1)
		2.03 (β), dd (13.5, 2.4)
8	82.4, C	
9	44.6, CH	2.41, dd (13.2, 2.7)
10	38.1, C	
11	32.0, CH_2	1.68 (α), m
		1.92 (β), dd (12.8, 2.8)
12	24.2, CH_3	1.54, s
13	15.2, CH_3	1.16, s
14	28.6, CH_3	1.23, s
15	23.3, CH_3	1.31, s
1′	38.2, C	
2′	43.1, CH	2.55, q (7.0)
3′	80.0, C	
4′	193.9, C	
5′	117.6, C	
6′	176.4, C	
7′	23.1, CH_3	1.44, s
8′	8.2, CH_3	1.00, d (7.0)
9′	173.9, C	
10′	8.2, CH_3	1.73, s
1′′	53.5, CH_3	3.83, s

^{1}H NMR: 600 MHz, ^{13}C NMR: 150 MHz (in $CDCl_3$)

Figure S120. NMR data of insuetusin B13 (**18**).

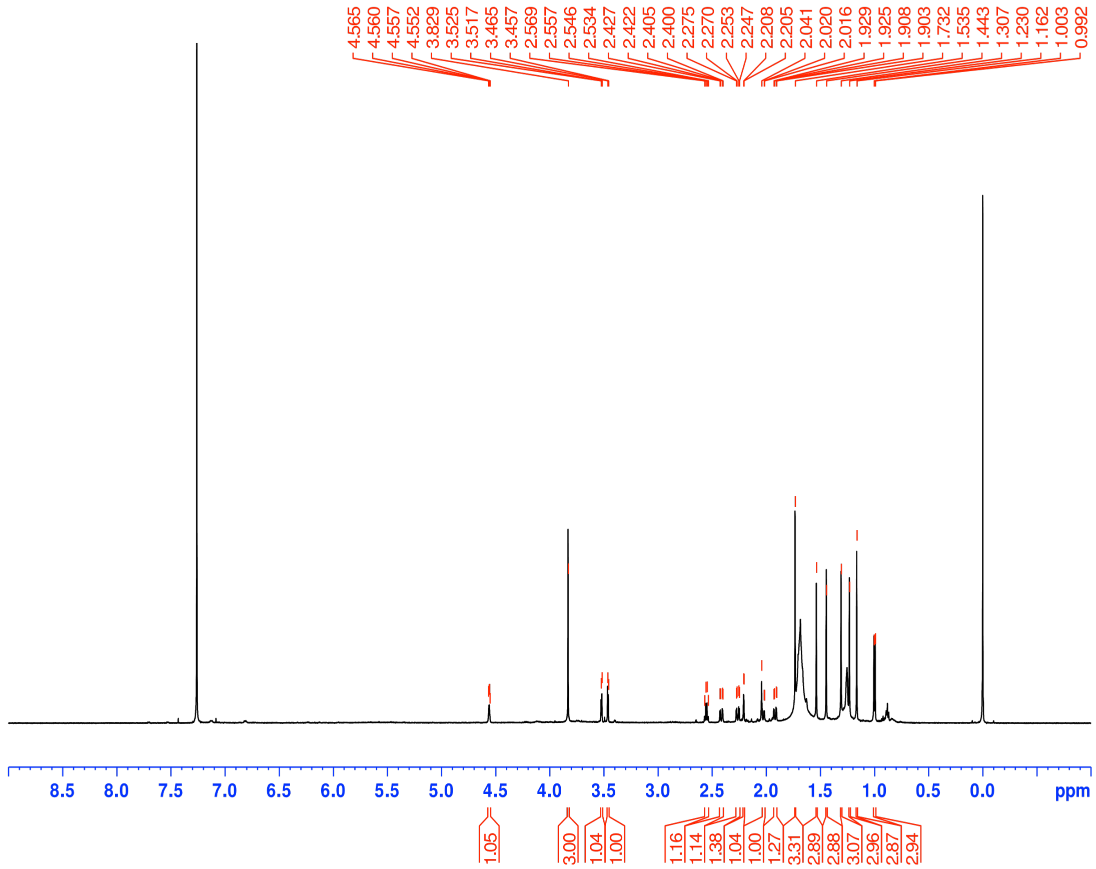

Figure S121. ^{1}H NMR spectrum of insuetusin B13 (**18**) in $CDCl_3$ at 600 MHz.

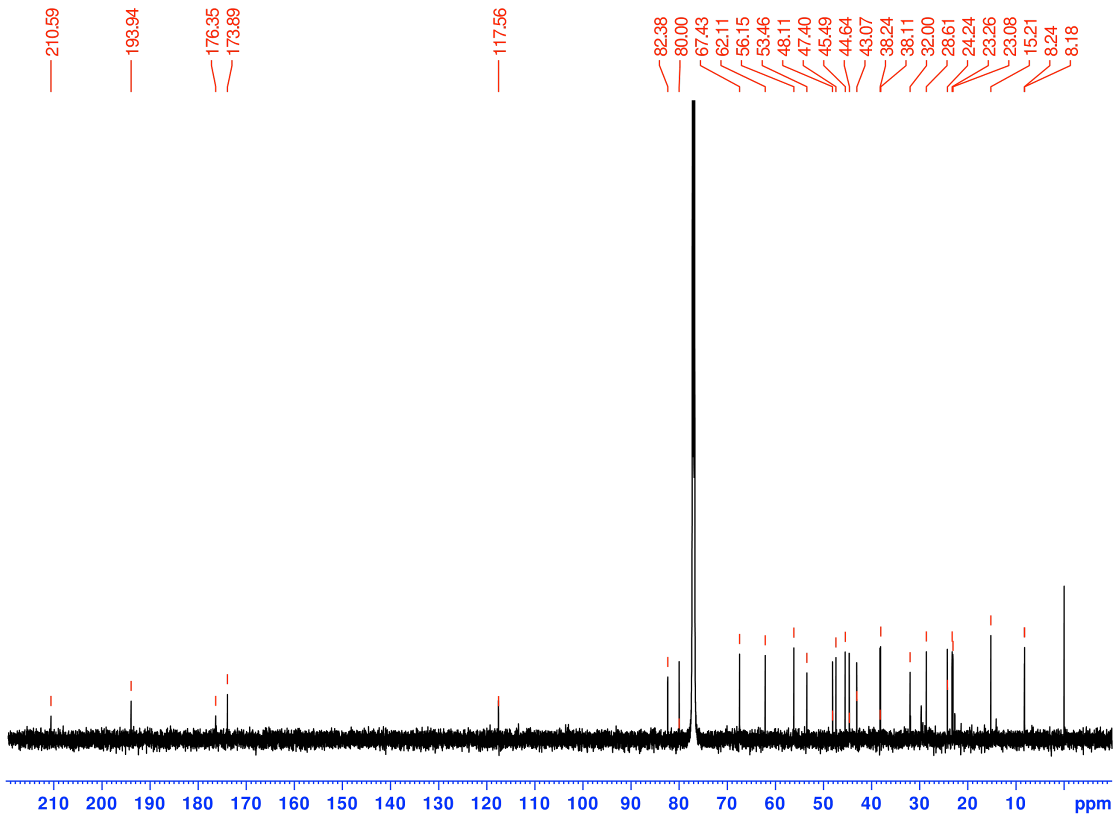

Figure S122. ^{13}C NMR spectrum of insuetusin B13 (**18**) in $CDCl_3$ at 150 MHz.

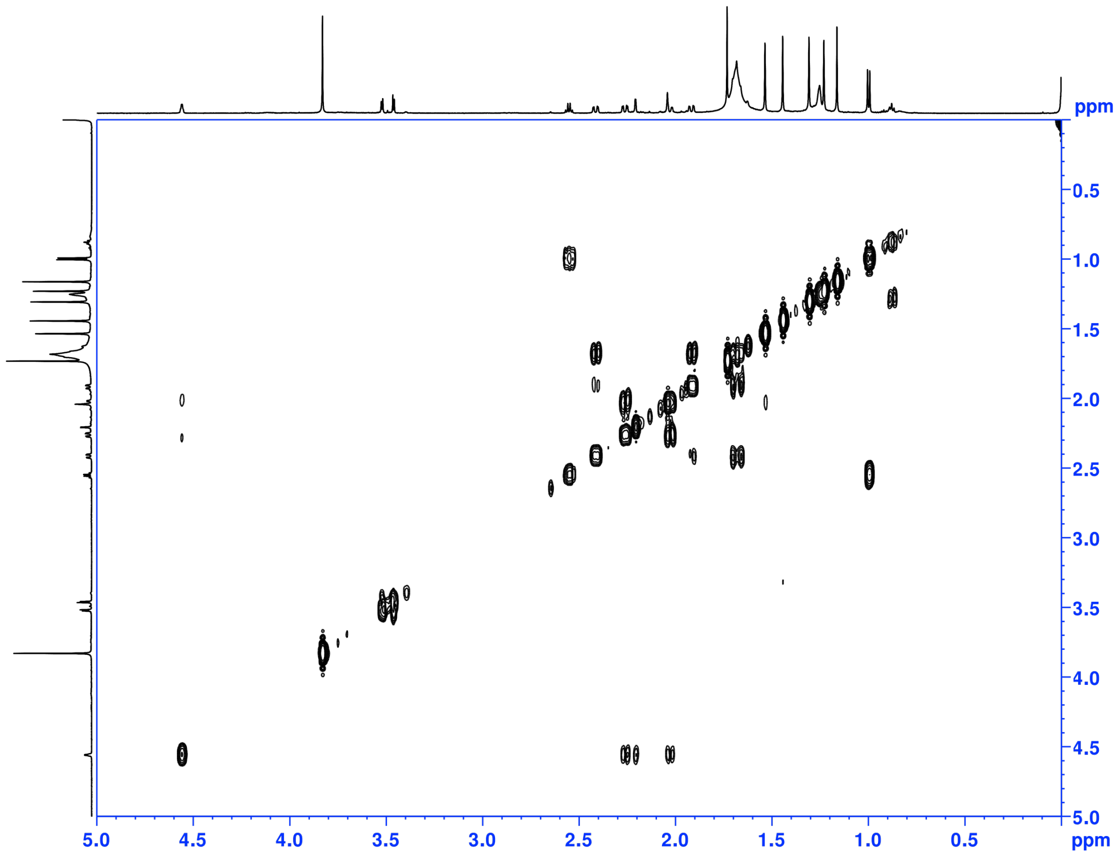

Figure S123. ^{1}H-^{1}H COSY spectrum of insuetusin B13 (**18**) in $CDCl_3$.

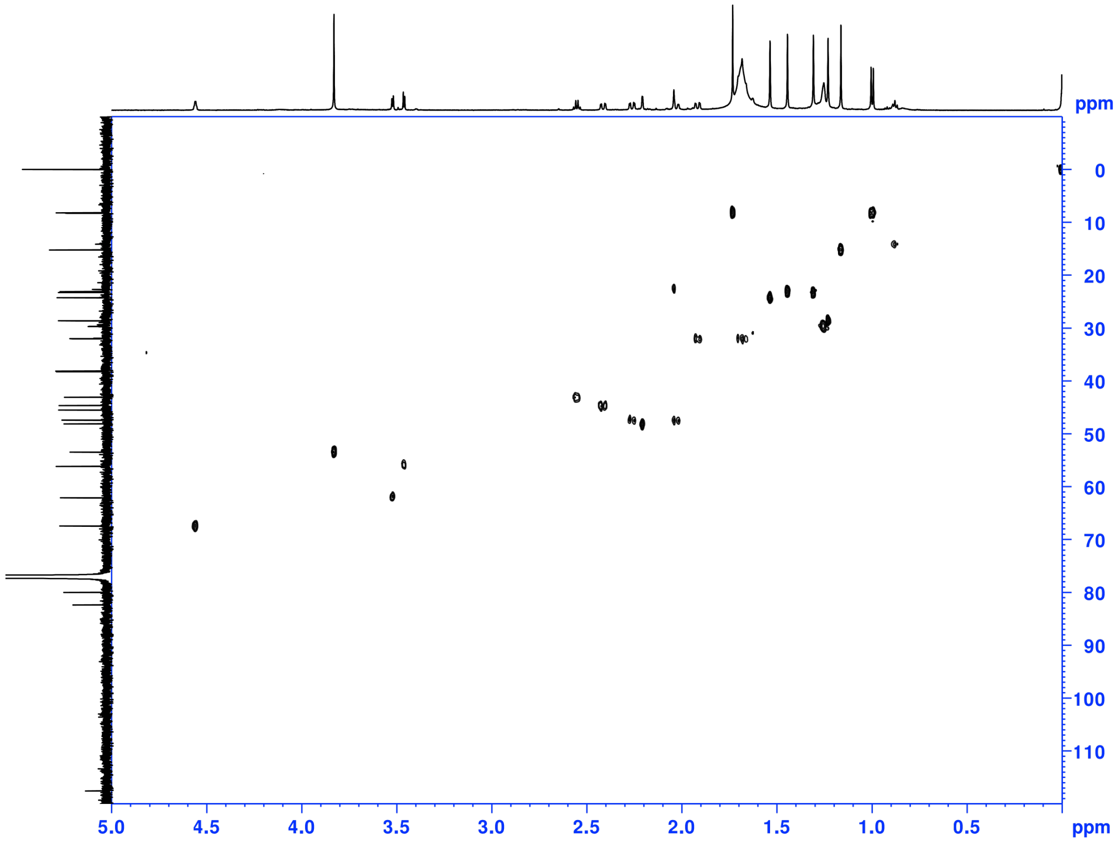

Figure S124. HSQC spectrum of insuetusin B13 (**18**) in $CDCl_3$.

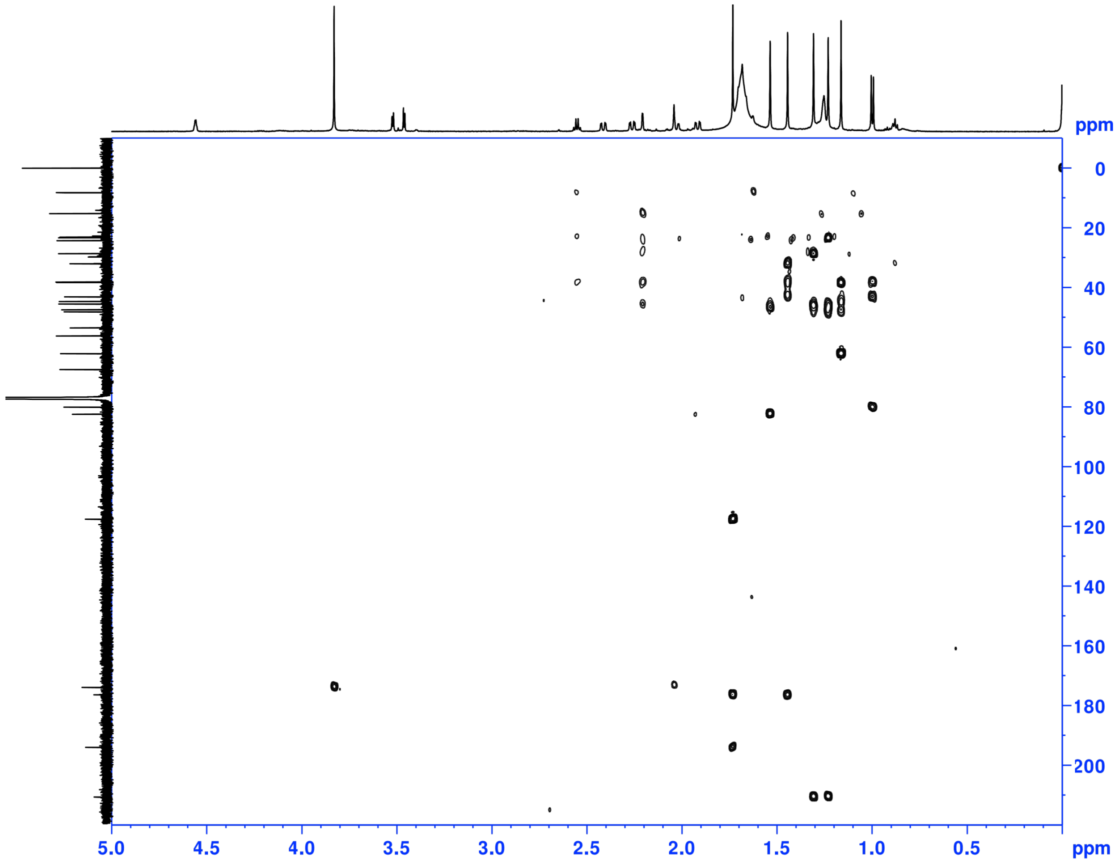

Figure S125. HMBC spectrum of insuetusin B13 (**18**) in $CDCl_3$.

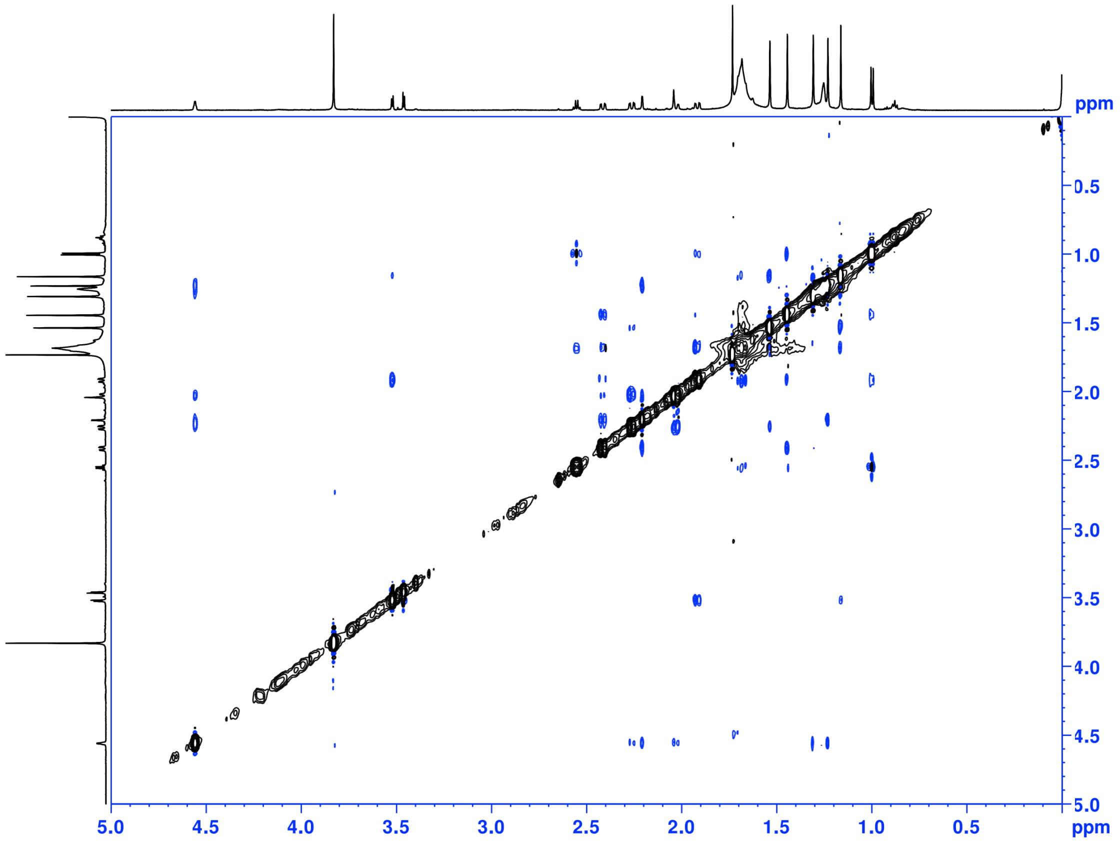

Figure S126. NOESY spectrum of insuetusin B13 (**18**) in $CDCl_3$.

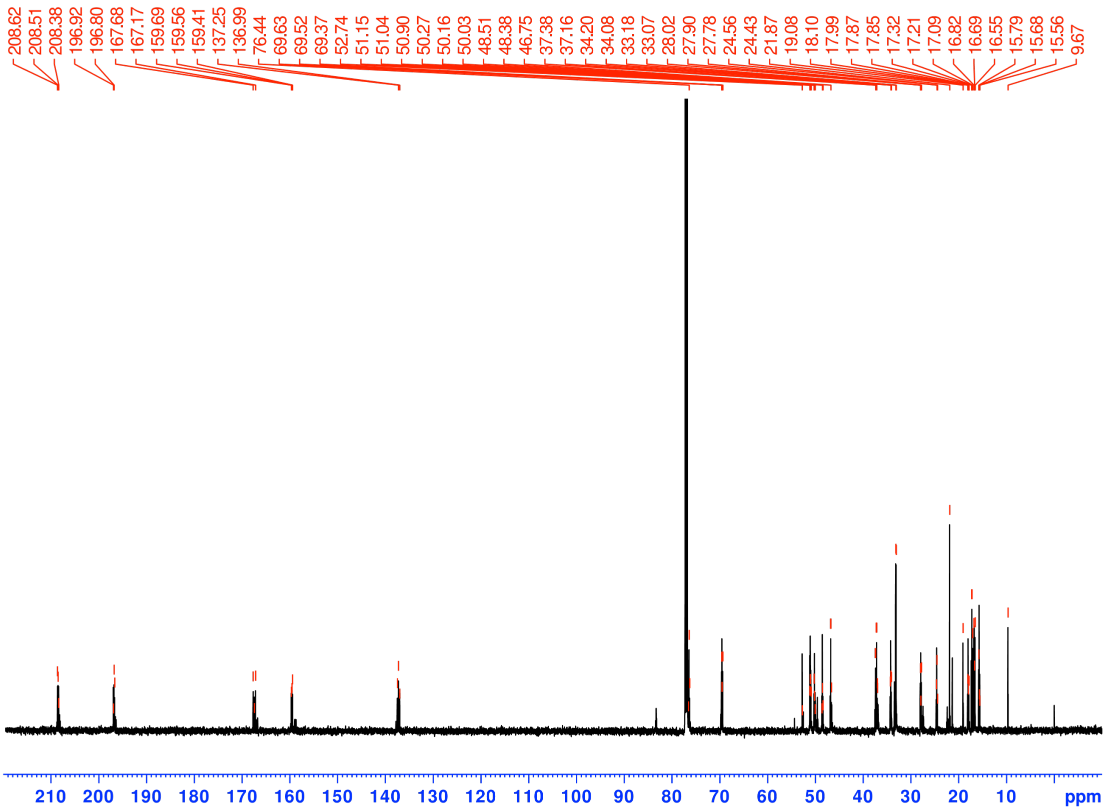

Figure S127. ^{13}C NMR spectrum of insuetusin A1 (**2**) labeled with sodium [1,2-$^{13}C_2$]acetate in $CDCl_3$ at 150 MHz.

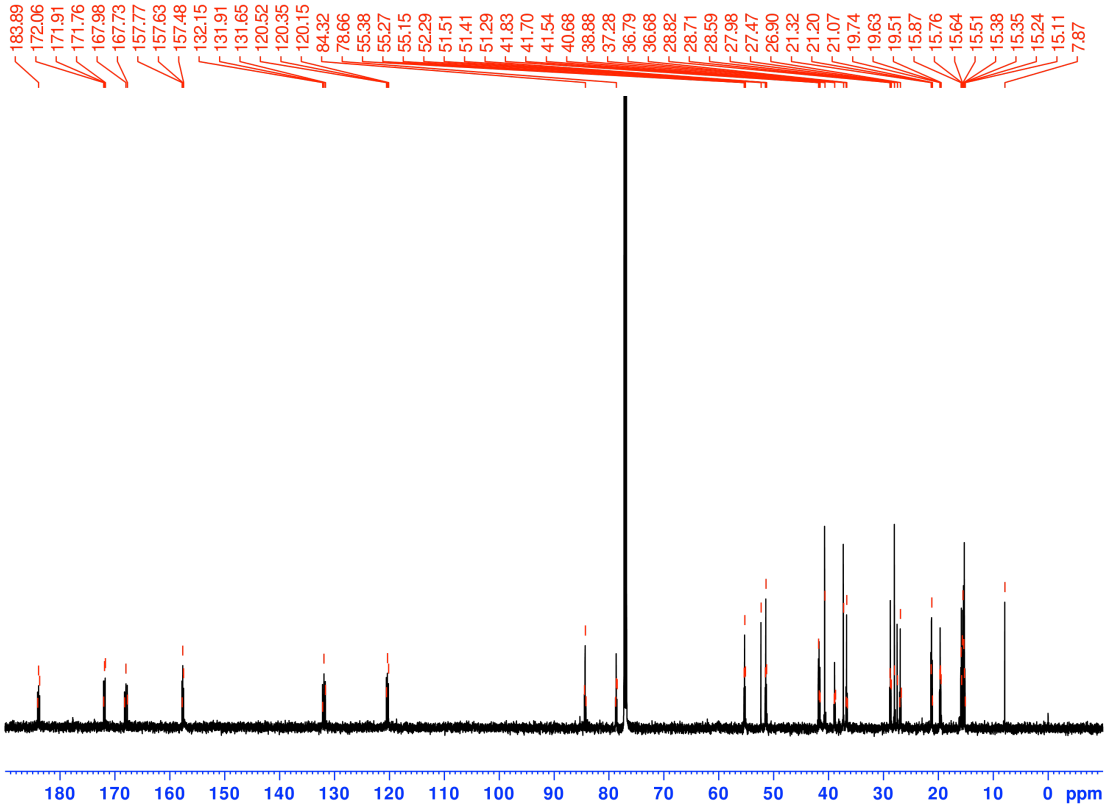

Figure S128. ^{13}C NMR spectrum of insuetusin B1 (**3**) labeled with sodium [1,2-$^{13}C_2$]acetate in $CDCl_3$ at 150 MHz.

X-Ray Crystallographic Data

Insuetusin A2 (4) (CCDC: 2174818)

ORTEP view of compound **4** with 50% probability of thermal ellipsoid.

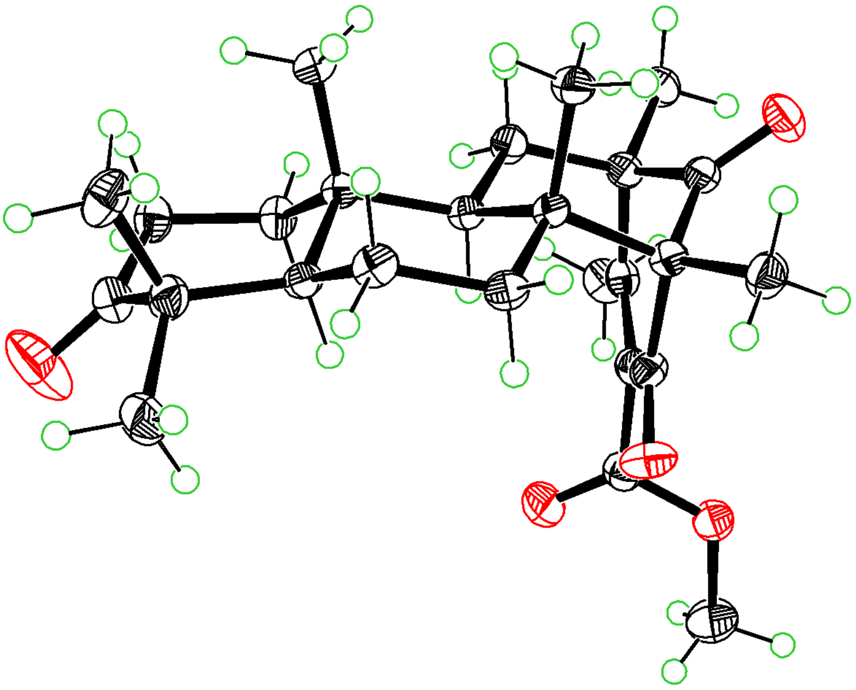

*Crystallographic data for insuetusin A2 (**4**).* $C_{26}H_{36}O_5$, M = 428.55, a = 8.2530(3) Å, b = 12.0228(4) Å, c = 23.2817(7) Å, α = 90°, β = 90°, γ = 90°, V = 2310.11(13) Å^3, T = 173(2) K, space group $P2_12_12_1$, Z = 4, μ(Cu Kα) = 0.672 mm^{-1}, 44 367 reflections measured, 4729 independent reflections (R_{int} = 0.0399). The final R_1 values were 0.0289 ($I > 2\sigma(I)$). The final $wR(F^2)$ values were 0.0796 ($I > 2\sigma(I)$). The final R_1 values were 0.0292 (all data). The final $wR(F^2)$ values were 0.0800 (all data). The goodness of fit on F^2 was 1.037. Flack parameter 0.01(3). The crystallographic information file (CIF) for this crystal structure was submitted to The Cambridge Crystallographic Data Centre (CCDC), under reference number 2174818.

Insuetusin A3 (5) (CCDC: 2174817)

ORTEP view of compound **5** with 50% probability of thermal ellipsoid.

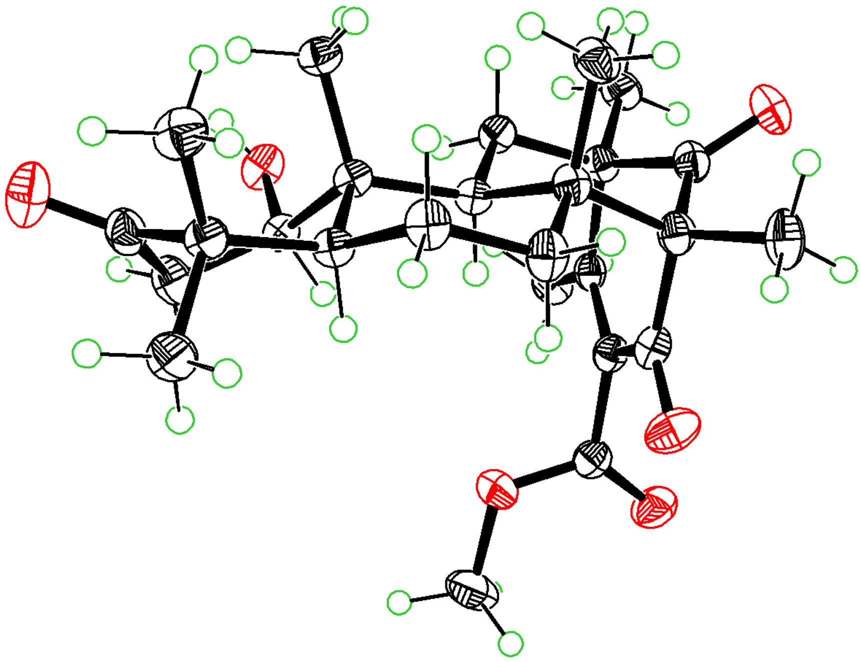

*Crystallographic data for insuetusin A3 (**5**).* $C_{26}H_{36}O_6$, $M = 444.55$, $a = 9.7178(3)$ Å, $b = 15.2909(5)$ Å, $c = 15.5463(5)$ Å, $\alpha = 90°$, $\beta = 90°$, $\gamma = 90°$, $V = 2310.09(13)$ Å^3, $T = 173(2)$ K, space group $P2_12_12_1$, $Z = 4$, μ(Cu Kα) = 0.725 mm^{-1}, 87 687 reflections measured, 4739 independent reflections ($R_{int} = 0.0579$). The final R_1 values were 0.0324 ($I > 2\sigma(I)$). The final $wR(F^2)$ values were 0.0890 ($I > 2\sigma(I)$). The final R_1 values were 0.0331 (all data). The final $wR(F^2)$ values were 0.0899 (all data). The goodness of fit on F^2 was 1.060. Flack parameter = 0.04(4). The crystallographic information file (CIF) for this crystal structure was submitted to The Cambridge Crystallographic Data Centre (CCDC), under reference number 2174817.

Insuetusin A4 (6) (CCDC: 2174819)

ORTEP view of compound **5** with 50% probability of thermal ellipsoid.

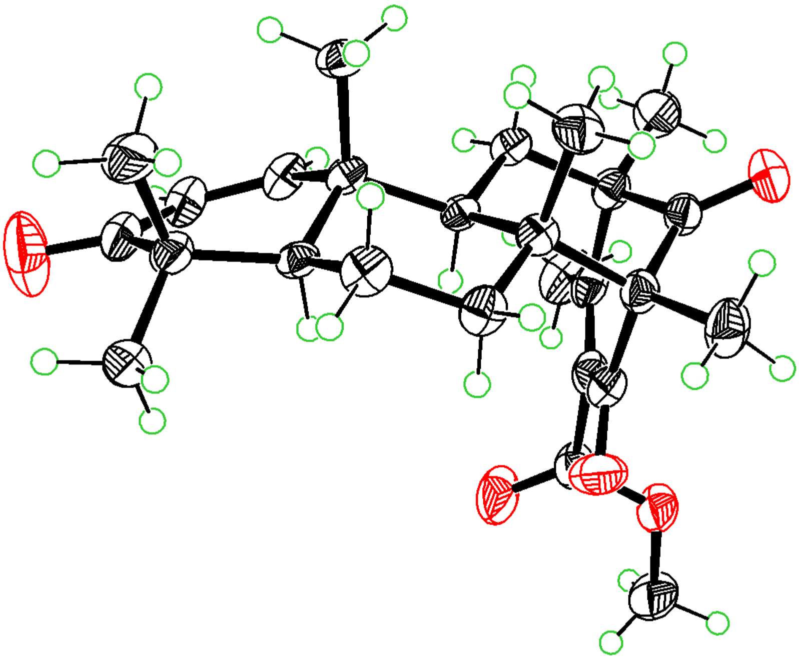

*Crystallographic data for insuetusin A4 (**6**).* $C_{26}H_{34}O_5$, M = 426.53, a = 8.3273(2) Å, b = 13.7746(3) Å, c = 19.8852(5) Å, α = 90°, β = 90°, γ = 90°, V = 2280.94(9) Å^3, T = 173(2) K, space group $P2_12_12_1$, Z = 4, μ(Cu Kα) = 0.680 mm^{-1}, 19 263 reflections measured, 4642 independent reflections (R_{int} = 0.0522). The final R_1 values were 0.0438 ($I > 2\sigma(I)$). The final $wR(F^2)$ values were 0.1188 ($I > 2\sigma(I)$). The final R_1 values were 0.0472 (all data). The final $wR(F^2)$ values were 0.1217 (all data). The goodness of fit on F^2 was 1.077. Flack parameter = 0.02(11). The crystallographic information file (CIF) for this crystal structure was submitted to The Cambridge Crystallographic Data Centre (CCDC), under reference number 2174819.

Insuetusin B3 (8) (CCDC: 2174822)

ORTEP view of compound **8** with 50% probability of thermal ellipsoid.

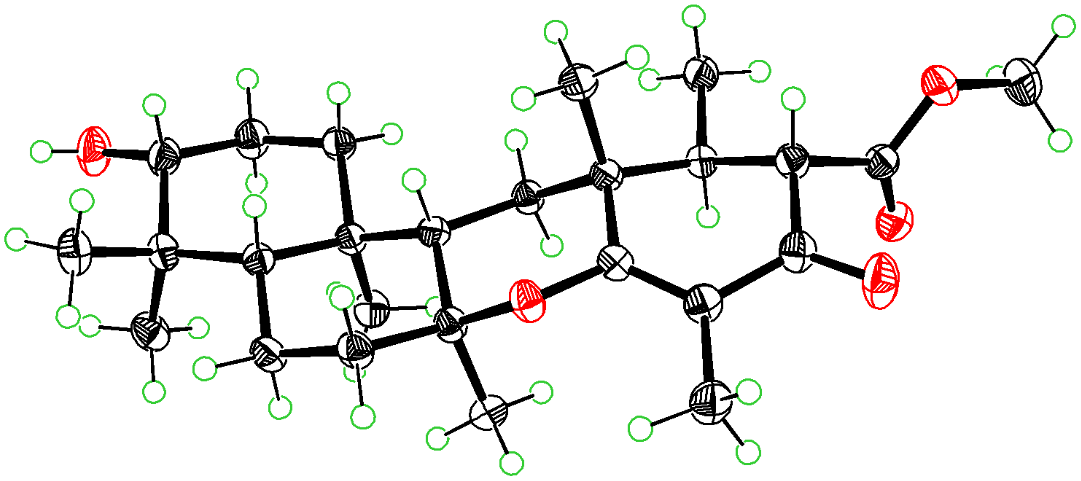

*Crystallographic data for insuetusin B3 (**8**).* $C_{26}H_{40}O_5$, $M = 432.58$, $a = 7.5943(3)$ Å, $b = 14.3966(5)$ Å, $c = 21.2500(8)$ Å, $\alpha = 90°$, $\beta = 90°$, $\gamma = 90°$, $V = 2323.31(15)$ Å^3, $T = 173(2)$ K, space group $P2_12_12_1$, $Z = 4$, μ(Cu Kα) = 0.669 mm^{-1}, 32 255 reflections measured, 4754 independent reflections ($R_{int} = 0.0519$). The final R_1 values were 0.0328 ($I > 2\sigma(I)$). The final $wR(F^2)$ values were 0.0855 ($I > 2\sigma(I)$). The final R_1 values were 0.0349 (all data). The final $wR(F^2)$ values were 0.0866 (all data). The goodness of fit on F^2 was 1.080. Flack parameter = 0.09(4). The crystallographic information file (CIF) for this crystal structure was submitted to The Cambridge Crystallographic Data Centre (CCDC), under reference number 2174822.

Insuetusin B6 (11) (CCDC: 2174820)

ORTEP view of compound **11** with 50% probability of thermal ellipsoid.

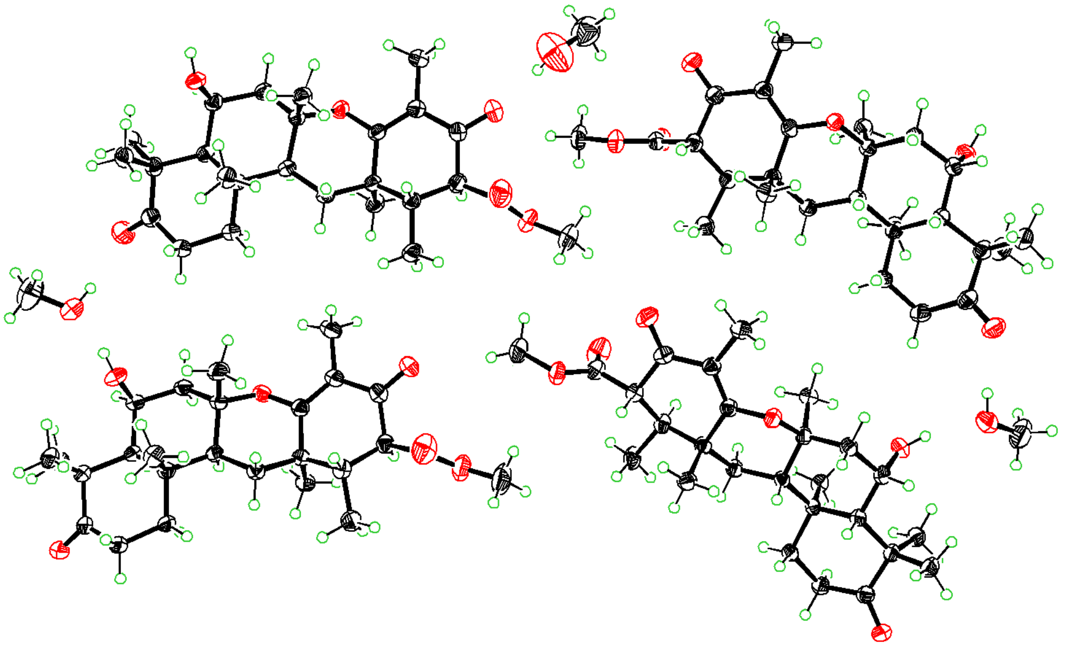

*Crystallographic data for insuetusin B6 (**11**).* $C_{26.75}H_{41}O_{6.75}$, M = 470.59, a = 10.0860(2) Å, b = 15.2512(3) Å, c = 17.1263(3) Å, α = 91.2110(10)°, β = 99.4940(10)°, γ = 98.3750(10)°, V = 2567.88(9) Å^3, T = 173(2) K, space group $P1$, Z = 4, μ(Cu Kα) = 0.698 mm^{-1}, 103 496 reflections measured, 20 178 independent reflections (R_{int} = 0.0421). The final R_1 values were 0.0360 ($I > 2\sigma(I)$). The final $wR(F^2)$ values were 0.0951 ($I > 2\sigma(I)$). The final R_1 values were 0.0376 (all data). The final $wR(F^2)$ values were 0.0966 (all data). The goodness of fit on F^2 was 1.080. Flack parameter = 0.00(3). The crystallographic information file (CIF) for this crystal structure was submitted to The Cambridge Crystallographic Data Centre (CCDC), under reference number 2174820.

Insuetusin B7 (12) (CCDC: 2174821)

ORTEP view of compound **12** with 50% probability of thermal ellipsoid.

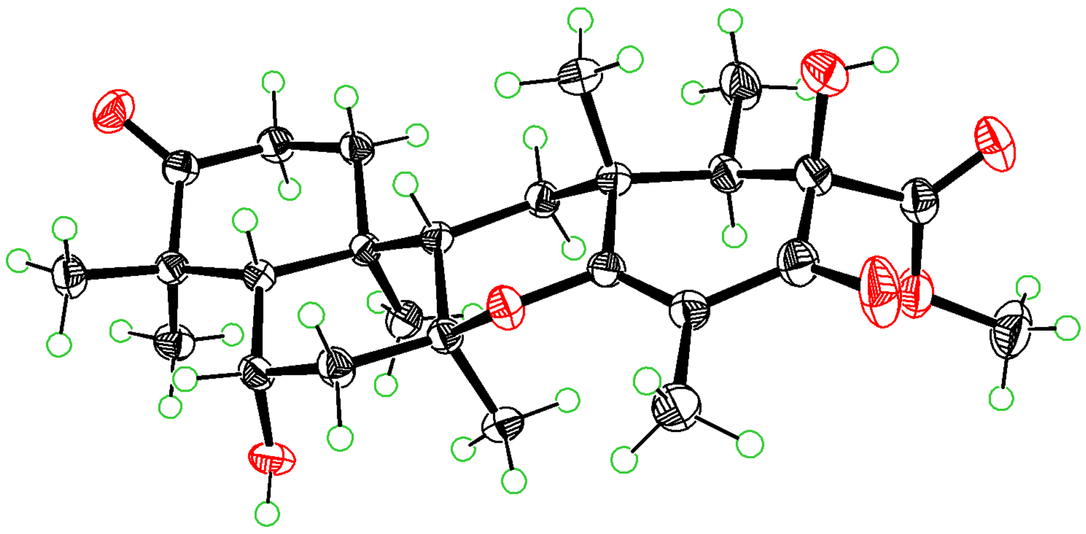

*Crystallographic data for insuetusin B7 (**12**).* $C_{26}H_{38}O_7$, M = 462.56, a = 8.2802(6) Å, b = 11.1074(8) Å, c = 13.0738(9) Å, α = 90°, β = 90.752(2)°, γ = 90°, V = 1202.31(15) Å^3, T = 213(2) K, space group $P2_1$, Z = 2, μ(Cu Kα) = 0.747 mm^{-1}, 46 472 reflections measured, 4907 independent reflections (R_{int} = 0.0484). The final R_1 values were 0.0283 ($I > 2\sigma(I)$). The final $wR(F^2)$ values were 0.0790 ($I > 2\sigma(I)$). The final R_1 values were 0.0285 (all data). The final $wR(F^2)$ values were 0.0792 (all data). The goodness of fit on F^2 was 1.036. Flack parameter = 0.03(3). The crystallographic information file (CIF) for this crystal structure was submitted to The Cambridge Crystallographic Data Centre (CCDC), under reference number 2174821.

Supplementary References

1. F. J. Jin, J. Maruyama, P. R. Juvvadi, M. Arioka and K. Kitamoto, *FEMS Microbiol. Lett.*, 2004, **239**, 79-85.

2. Q. Al Abdallah, W. Ge and J. R. Fortwendel, *mSphere*, 2017, **2**, e00446-00417.

3. Y. Matsuda, T. Bai, C. B. W. Phippen, C. S. Nødvig, I. Kjærbølling, T. C. Vesth, M. R. Andersen, U. H. Mortensen, C. H. Gotfredsen, I. Abe and T. O. Larsen, *Nat. Commun.*, 2018, **9**, 2587.

4. O. Yamada, S. Na Nan, T. Akao, M. Tominaga, H. Watanabe, T. Satoh, H. Enei and O. Akita, *J. Biosci. Bioeng.*, 2003, **95**, 82-88.

5. C. Liu, A. Minami, T. Ozaki, J. Wu, H. Kawagishi, J.-i. Maruyama and H. Oikawa, *J. Am. Chem. Soc.*, 2019, **141**, 15519-15523.

6. T. Fujii, H. Yamaoka, K. Gomi, K. Kitamoto and C. Kumagai, *Biosci. Biotechnol. Biochem.*, 1995, **59**, 1869-1874.

7. F. Jin, J. Maruyama, P. Juvvadi, M. Arioka and K. Kitamoto, *Biosci. Biotechnol. Biochem.*, 2004, **68**, 656-662.

8. T. Kubodera, N. Yamashita and A. Nishimura, *Biosci. Biotechnol. Biochem.*, 2002, **66**, 404-406.
